# Supplementary material for: A retrospective cohort study of valproate and infertility in men with epilepsy or bipolar disorder using international health data
Source: Nat Commun. 2025 Sep 15;16:8221. doi: 10.1038/s41467-025-63469-0 (PMC12436621; doi:10.1038/s41467-025-63469-0)
Supplement: Supplementary file 1 — Supplementary Information [file 41467_2025_63469_MOESM1_ESM.docx]

**Supplementary materials**

**Study title: A retrospective cohort study of valproate and infertility in men with epilepsy or bipolar disorder using international health data**

[Supplementary Table 1: Overview of human studies of male fertility on valproate 3](#_Toc194381469)

[Search strategy: 3](#_Toc194381470)

[Supplementary Table 1 3](#_Toc194381471)

[References 6](#_Toc194381472)

[Supplementary Table 2: Propensity score matching men with epilepsy or bipolar disorder exposed (cohort 1) and unexposed (cohort 2) to valproate – lifetime outcome assessment 7](#_Toc194381473)

[Supplementary Table 3: Propensity score matching men with epilepsy or bipolar disorder exposed (cohort 1) and unexposed (cohort 2) to valproate – 30-day outcome assessment 19](#_Toc194381474)

[Supplementary Table 4: Propensity score matching men with epilepsy or bipolar disorder exposed (cohort 1) and unexposed (cohort 2) to valproate – 60-day outcome assessment 31](#_Toc194381475)

[Supplementary Table 5: Propensity score matching men with epilepsy or bipolar disorder exposed (cohort 1) and unexposed (cohort 2) to valproate – 90-day outcome assessment 43](#_Toc194381476)

[Supplementary Table 6: Propensity score matching men with epilepsy or bipolar disorder exposed (cohort 1) and unexposed (cohort 2) to valproate – 180-day outcome assessment 55](#_Toc194381477)

[Supplementary Table 7: Propensity score matching men with epilepsy or bipolar disorder exposed (cohort 1) and unexposed (cohort 2) to valproate – 360-day outcome assessment 67](#_Toc194381478)

[Supplementary Table 8: Propensity score matching men with epilepsy or bipolar disorder exposed (cohort 1) and unexposed (cohort 2) to valproate – 2-year outcome assessment 79](#_Toc194381479)

[Supplementary Table 9: Propensity score matching men with epilepsy or bipolar disorder exposed (cohort 1) and unexposed (cohort 2) to valproate – 5-year outcome assessment 91](#_Toc194381480)

[Supplementary Table 10: Propensity score matching men with epilepsy or bipolar disorder exposed (cohort 1) and unexposed (cohort 2) to valproate – 10-year outcome assessment 103](#_Toc194381481)

[Supplementary Table 11: Propensity score matching men with epilepsy exposed (cohort 1) and unexposed (cohort 2) to valproate 115](#_Toc194381482)

[Supplementary Table 12: Propensity score matching men with bipolar disorder exposed (cohort 1) and unexposed (cohort 2) to valproate 127](#_Toc194381483)

[Supplementary Table 13: Propensity score matching men with epilepsy or bipolar disorder exposed (cohort 1) and unexposed (cohort 2) to valproate with key modifiable risk factors excluded 139](#_Toc194381484)

[Supplementary Table 14: Propensity score matching men with epilepsy or bipolar disorder exposed and unexposed to drugs known to cause infertility 149](#_Toc194381485)

[Supplementary Table 15: Serum valproate levels in men with epilepsy or bipolar disorder 160](#_Toc194381486)

[Supplementary Table 16: Serum valproate levels in men with epilepsy 161](#_Toc194381487)

[Supplementary Table 17: Serum valproate levels in men with bipolar disorder 162](#_Toc194381488)

[Supplementary Table 18: Serum valproate levels in men with epilepsy or bipolar disorder exposed and unexposed to valproate with key modifiable risk factors excluded 163](#_Toc194381489)

[Supplementary Table 19: Drugs known to cause male infertility 164](#_Toc194381490)

[Supplementary Table 20: Infertility outcomes in men with epilepsy and or bipolar disorder exposed and unexposed to drugs known to cause infertility 171](#_Toc194381491)

[Supplementary Figure 1: panel of survival plots showing men with epilepsy or bipolar disorder exposed and unexposed to drugs known to cause infertility 172](#_Toc194381492)

# Supplementary Table 1: Overview of human studies of male fertility on valproate

## Search strategy:

1. **MEDLINE (PubMed)** = (Valproic Acid[MeSH Terms] OR Valproate OR Valproic Acid OR Depakote OR VPA) AND (Infertility, Male[MeSH Terms] OR Spermatozoa[MeSH Terms] OR Testicular Diseases[MeSH Terms] OR Spermatogenesis[MeSH Terms] OR Reproductive Health[MeSH Terms] OR Male Fertility OR Sperm Quality OR Semen Parameters OR Sexual Function OR Testicular Function OR Testicular Volume) AND Humans[MeSH Terms]

Results = 141

1. **Scopus** = TITLE-ABS-KEY ( valproate OR "valproic acid" OR depakote OR vpa ) AND TITLE-ABS-KEY ( "male infertility" OR spermatozoa OR spermatogenesis OR "testicular disease" OR "testicular function" OR "reproductive health" OR "male fertility" OR "sperm quality" OR "semen parameters" OR "sexual function" ) AND ( LIMIT-TO ( DOCTYPE , "ar" ) OR LIMIT-TO ( DOCTYPE , "re" ) )

Results = 333

1. Bibliographies of relevant review articles found
2. A + B + C
3. Exclude irrelevant articles
4. D + E = Supplementary Table 1

## Supplementary Table 1

| Author, year, country | Study design | Exposure and inclusion | Control | Exclusion criteria | Findings | Author conclusions | Limitations | Level of evidence^1^ |
| --- | --- | --- | --- | --- | --- | --- | --- | --- |
| Guo 2021^2^ – China | Prospective cohort | **▪** 44 men with epilepsy on VPA (23 men) and OXC (21 men) monotherapy  **▪** These young men (age limit unspecified) were on 6 months of VPA or OXC | 30 age-matched healthy men | **▪** Use of hormones, antidepressants, or drugs to improve sexual function; (2) patients with liver or kidney dysfunction, thyroid disease, diabetes, infectious diseases, varicocele, cryptorchidism, a history of testicular surgery, Klinefelter syndrome, or urinary system diseases; (3) patients who had a history of long-term alcoholism, smoking, or exposure to toxic substances; and (4) patients with mental illness, intracranial occupying lesions, brain injury, or progressive degeneration of the nervous system. | VPA exposure pre 🡪 post treatment  **▪ 🡫 sperm concentration (×10^6^/mL):** 37.70 🡪 30.00 (*p* = 0.003)  **▪ 🡫 total number of sperm (×10^6^/ejaculation):** 130.37 🡪 97.29 (*p* = 0.012)  **▪ 🡫 sperm with forward movement (%):** 41.95 🡪 34.73 (*p* = 0.001)  **▪ 🡩 Prolactin** (*p* = 0.029)  **▪ No change:** Normal sperm %, IIEF-5, FSH, LH, testosterone, estradiol  Other results:  ▪ 🡫 Marriage and procreation rates in PWE vs. controls (p < 0.01)  ▪ 🡫 Sperm concentration, total sperm count, and IIEF-5 questionnaire in PWE vs. controls (p < 0:05)  ▪No change in sex hormone levels in PWE vs. controls | **▪** Sexual function and sperm quality were reduced in young males with epilepsy.  **▪** VPA may exert a negative effect on sexual function and sperm quality.  **▪** OXC has no harmful effect on sexual function and sperm quality in young males with epilepsy. | **▪** Small sample  **▪** Confounders like sex hormone disorders not accounted for  **▪** Large number of exclusions make sample less generalisable to real-world pool of patients  **▪** Lack of PWE untreated makes it difficult to differentiate between effect of ASMs vs. epilepsy on outcomes  **▪** Not a causal design: inferences about associations alone are permissible  **▪** The study of ASM monotherapy makes this less generalisable to a real-world epilepsy population, many of whom will be on ASM polytherapy | III |
| Markoula 2020^3^ – Greece | Prospective cohort | **▪** 17 infertile men with epilepsy on VPA monotherapy: 9 switched to LEV, 8 switched to LTG  **▪** Men (age limit unspecified) were on VPA over ≥2 years, seizure-free for 1-2 years, unable to conceive over 12 months. Female-factor infertility was not excluded. | n/a | **▪** Any other possible cause of male infertility, such as varicocele, chromosomal abnormalities and other chronic disease, as well as recognisable female factor infertility.  **▪** So as to focus on infertility “purely” related to reduction of sperm parameters, males with reported sexual dysfunction were excluded. This also limits generalisability. | Switch from VPA to LEV or LTG  **▪ 🡩 Total sperm motility (%):** 45.8 🡪 65.8 (*p* = <0.01)  **▪ 🡩 Non-progressive motility (%):** 18.4 🡪 25.5 (*p* = <0.02)  **▪ 🡫 Head defects (%):** 74.4 🡪 63.8 (*p* = <0.03)  **▪ 🡩 FSH**  (*p* = <0.03), **🡩 LH** (*p* = <0.03)  **▪ No change:** Semen volume, count, progressive motility, neck defects, tail defects, ERC, TZI  Other results:  **▪** Spontaneous pregnancies were reported in 3 of the patients' partners – one switched to LEV and two switched to LTG. All three were still on a valproate –a reducing regimen– taking a lower dose than at recruitment. | **▪** While not all of the results were significant, the overall trend showed that switching from VPA to LEV or LMG improved semen quantitative and quality characteristics in infertile men with epilepsy, with three successful conceptions recorded.  **▪** No association of the duration of treatment and the dose of VPA with the sperm parameters. | **▪** Small sample, unmatched  **▪**  Any potential effect of epilepsy *per se* on spermatozoa could not be explored. Epilepsy itself may have a direct effect on spermatogenesis, with untreated men with epilepsy having low seminal fluid volume, oligospermia and an increased number of abnormal spermatozoa.  **▪** The recruitment of patients with good seizure control was made in order to decrease the effect of epilepsy on spermatozoa on the one hand and to minimise the chance of seizure exacerbation after switching to LEV or LMG on the other. However, results less generalisable to a general epilepsy population, who may not be seizure-free.  **▪** Large number of exclusions make sample less generalisable to real-world pool of patients  **▪** Not a causal design: inferences about associations alone are permissible  **▪** The study of ASM monotherapy makes this less generalisable to a real-world epilepsy population, many of whom will be on ASM polytherapy  **▪** Most men did not conceive despite switching from valproate.  **▪** Where conception occurred, the men were still on valproate, making it difficult to confidently infer an association with infertility. | III |
| Ocek 2018 – Turkey^4^ | Case-control | **▪** 59 men with epilepsy: 30 on VPA, 29 on CBZ monotherapy  **▪** Men aged 18-56 years were on VPA or CBZ for ≥6 months | 30 healthy men | **▪** Endocrine disorder and hormone therapy, urogenital disorder or surgery (testicular surgery, varicocele, etc.), presence of a systemic disease that may affect sex hormone levels (such as atherosclerosis and DM), drug and alcohol abuse, and psychiatric problems | VPA exposure 🡪 control  **▪ 🡫 Sperm volume (mL):** 1.98 🡪 3.72 (*p* = <0.0001)  **▪ 🡫 Normal sperm (%):** 3.58 🡪 5.41 (*p* = <0.003)  **▪ 🡩 DHEAS** (*p* = <0.014)**, 🡫 free testosterone** (*p* = <0.038), **🡩 estradiol** (*p* = <0.0001)**, 🡫 BAT/BAE** (*p* = <0.0001)  **▪ 🡫** IIEF-5 (*p* = <0.0001)  **▪ No change:** Sperm concentration, motility, abnormal sperm heads, midpieces, tails, FSH, LH, prolactin, total testosterone, SHBG, BAT/LH  Other results  **▪No change:** VPA vs. CBZ in all parameters | ▪ Monotherapy with VPA or CBZ causes notable effects on reproductive endocrine functions, sperm parameters, and sexual function in male patients with epilepsy. | **▪** Small sample – unmatched, single-centre study  **▪** Comparison could not be conducted as they couldn’t obtain reliable pre-treatment data.  **▪** Unable to examine the psychiatric or psychosocial factors which can affect the sexual functions, especially sexual desire and general satisfaction.  **▪** Unable to examine mood state, differences in sexual hormones and their effect on sexual function, which are also all confounders.  **▪** Large number of exclusions make sample less generalisable to real-world pool of patients  **▪** Lack of PWE untreated makes it difficult to differentiate between effect of ASMs vs. epilepsy on outcomes  **▪** Not a causal design: inferences about associations alone are permissible  **▪** The study of ASM monotherapy makes this less generalisable to a real-world epilepsy population, many of whom will be on ASM polytherapy | IV |
| Hamed 2015 – Egypt^5^ | Case-control | **▪** 55 men with epilepsy on VPA (n=28) or VPA + CBZ (n=27)  **▪** Adult age men (limits unspecified, range 20-48) on VPA/VPA+CVZ for ≥2 years | 30 age- , BMI- and socioeconomic-status-matched healthy men | **▪** (1) a history of or examination suggesting other neurological, medical or surgical problems which can impair sexual function or sperm parameters (such as diabetes mellitus, coronary, renal, hepatic or genitourinary diseases and pelvic trauma, surgery or radiation therapy); (2) use of alcohol, excess tobacco, excess caffeine, recreational drugs or regular medications (such as antihypertensives, heart medications, antidepressants, tranquilizers, and sedatives) which my alter sperm parameters; and (3) use of nutritional therapies (such as carnitine, arginine, zinc, selenium and vitamin B12 supplements) which may improve sperm parameters. | VPA and VPA+CBZ exposure 🡪 control  **▪ 🡫 Mean free testosterone (pg/ml):** 12.56 🡪 25.55 (p < 0.01)  **▪ 🡫 Sperm concentration (million/ml):** 15.55 🡪 25.60 (p < 0.001)  **▪ 🡫 Sperm count (×10^6^):** 45.56 🡪 88.76 (p < 0.0001)  **▪ 🡫 Progressively motile sperm (×10^6^):** 25.80 🡪 75.58 (p < 0.0001)  **▪ 🡩 Non-progressively motile sperm (×10^6^):** 10.33 🡪 5.65 (p < 0.05)  **▪ 🡩 Immotile sperm (×10^6^):** 15.50 🡪 5.55 (p < 0.01)  **▪ 🡩 Abnormal forms (%):** 50.50 🡪 15.35 (p < 0.0001)  **▪ 🡫 Seminal total plasma carnitine** **(lmol/l)** (p < 0.01)  **▪ 🡫 Testicular volume (cm^3^):** 12.23 🡪 16.25 (right), 11.27 🡪 16.07 (left) (p < 0.01)  **▪ No change:** Prolactin, FSH, LH, free thyroxine, TSH, semen volume, liquefaction time, pus cells  VPA exposure 🡪 VPA+CBZ  **▪ 🡩** Sperm concentration, sperm count, progressively motile sperm, non-progressively motile sperm, immotile sperm, **🡫 abnormal forms**  **▪ No change:** free testosterone, semen volume, testicular volume  Other results  **▪** Significant correlations between: (1) duration of illness, sperm count and carnitine; (2) duration of ASM treatment, sperm count and carnitine; (3) VPA dose and sperm count, immotile sperm, abnormal forms, testicular volume and carnitine; (4) free testosterone and sperm count; and (5) carnitine and sperm count, immotile sperm and abnormal forms. | ▪ Chronic treatment with VPA, as a monotherapy or part of a polytherapy regimen, is associated with reduced sperm concentration, count and motility, increased abnormal sperm count and reduced testicular volume. | ▪ The findings of this study cannot exclude the role of epilepsy itself in sperm abnormalities.  **▪** Large number of exclusions make sample less generalisable to real-world pool of patients  **▪** Lack of PWE untreated makes it difficult to differentiate between effect of ASMs vs. epilepsy on outcomes  **▪** Not a causal design: inferences about associations alone are permissible | IV |
| Artama 2006^6^ – Finland | Retrospective cohort | **▪** PWE on CBZ overall (n = 2689), monotherapy (n = 2365); OXC overall (n = 832), monotherapy (n = 631); VPA overall (n = 1546), monotherapy (n = 1116)  **▪** PWE untreated (n = 2714–2785  **▪**Age-limits 15-49 years | 13 378–13 689 people without epilepsy | **▪** Persons with any children before the start of the follow-up | **▪ 🡫 Birth rate** in men and women with epilepsy vs. without epilepsy  **▪ 🡫 Birth rate** in PWE on ASMs vs. people without epilepsy in both sexes  **▪ 🡫 Birth rate** in untreated PWE vs. people without epilepsy in both sexes  **▪ 🡫 Birth rate** on CBZ and OXC vs. VPA in both sexes | ▪ Birth rate is decreased among PWE on ASMs, more so in men.  ▪ Among male patients with epilepsy, CBZ, OXC and VPA all were associated with low birth rate in relation to men without epilepsy.  ▪ However, only OXC was related to reduced birth rate amongst treated PWE compared with untreated PWE. | ▪ Unavailable information on semen quality or female fecundity, likely confounders  ▪ Unavailable information on intention to conceive and contraceptive use, also confounders  ▪ Institutionalised patients and patients who did not want reimbursement were not included, potentially limiting generalisability.  ▪ Evaluated the effect of ASM usage based on the information on ASM purchases – actual drug compliance could not be evaluated.  ▪ Unable to assess the time of conception, because the ‘intention to conceive’ was not available.  ▪ Did not have information on spontaneous or induced abortions – could not estimate the impact of these factors on decreased birth rate  ▪ Could not estimate the effect of psychosocial aspects on reproduction  ▪ As they did not have information on epilepsy type, they could not distinguish the effect of epilepsy and ASMs on fertility, that is, confounding by indication is possible.  **▪** Not a causal design: inferences about associations alone are permissible | III |
| Isojärvi 2004 – Finland^7^ | Case-control | **▪** 60 men with epilepsy 27 on VPA, 15 on CBZ, 18 on OXC monotherpy  **▪** Age-limits 18-45 years, no minimum treatment duration specified | 41 healthy men | **▪** Another illness or medication  **▪** Almost all of the patients treated with CBZ (15/18) or OXC (18/18) had cryptogenic partial epilepsy, whereas close to all of the VPA-treated men (27/29) had generalised epilepsy. Therefore, to reduce the number of confounding factors, only CBZ-treated men with partial epilepsy (n = 15), all OXC-treated men (n = 18), and VPA-treated men with generalised epilepsy (n = 27) were included in the analysis of the data. | VPA exposure 🡪 control  **▪ 🡫 Frequency of motile sperm (%):** 44 🡪 61 (p < 0.05)  **▪ 🡩 No. with <5% of sperm with normal morphology:*** 6 (24%) 🡪 1 (3%) (p < 0.01)  **▪ 🡩 No. with <50% of sperm motile, grades A + B:** 16 (64%) 🡪 14 (35%) (p < 0.05)  **▪ 🡩 No. with any sperm abnormality:** 20 (80%) 🡪 19 (40%) (p < 0.01)  **▪ 🡫 Testicular volume** (p = 0.01)  **▪ 🡩 Androstenedione** (p < 0.001)  **▪ No change:** Sperm concentration, frequency of morphologically normal sperm, dehydroepiandrosterone sulfate, testosterone, SHBG, FAI, LH, FSH, prolactin, inhibin-B  Other results  **▪** VPA-treated men with abnormal sperm had smaller testicular volumes (p = 0.003) than the control men, whereas the testicular volume of VPA-treated men with normal sperm was similar to control men.  **▪** No correlations between the duration of epilepsy or its treatment, the drug dose or serum concentration, any of the serum hormone concentrations or sex hormone binding globulin level, or free androgen index value and the sperm quality or testicular volume in any of the patient groups or in the control men. | ▪ CBZ, OXC, and VPA may be associated with abnormal sperm morphology in men with epilepsy.  ▪ However, partial or generalised epilepsy may also affect sperm morphology.  ▪ CBZ is also associated with low sperm concentration and poor motility of sperm in men with partial epilepsy and VPA with reduced motility of sperm in men with generalised epilepsy.  ▪ The number of men with normal sperm is low among VPA-treated men with generalised epilepsy.  ▪ VPA is also associated with reduced volume of the testicles, and the reduced testicular volume seems to correlate with abnormalities in sperm quality. | **▪** Small sample – exclusions limit generalisability to real-world pool of patients  **▪** Unmatched samples – confounders not accounted for  **▪** Lack of PWE untreated makes it difficult to differentiate between effect of ASMs vs. epilepsy on outcomes  **▪** Not a causal design: inferences about associations alone are permissible  **▪** The study of ASM monotherapy makes this less generalisable to a real-world epilepsy population, many of whom will be on ASM polytherapy | IV |
| Mikkonen 2004 – Finland^8^ | Case-control | **▪** 70 boys and young men with epilepsy: 25 on VPA, 28 on CBZ; 5 on LTG, 12 on OXC monotherapy  **▪** Age-limits 7-20 years, on CBZ, LTG, OXC, or VPA for >1 year | 70 healthy males matched for gender, age, and pubertal stage | **▪** No history of brain damage, mental retardation, steroid treatment for intractable epilepsy, or any illness possibly affecting hormonal functions. Mild asthma or allergic diseases were allowed. | VPA exposure 🡪 control  **▪ 🡩 Androstenedione at all pubertal stages** (*p* ≤ 0.02)  **▪ Independent predictors of elevated A level:** VPA (OR 117.5, 95% CI 12.5–1,103.7), and weight for height (OR 1.1, 95% CI 1.04–1.2)  **▪ No change:** Testicular volume, testosterone, DHEAS, SHBG, LH, FSH, inhibin-B | ▪ CBZ and VPA, but not LTG and OXC, are associated with changes in serum sex-hormone levels in boys and young men with epilepsy.  ▪ Testicular volume was not decreased in the VPA-treated young male subjects with epilepsy.  ▪ The clinical consequences of high serum A levels during pubertal development in boys taking VPA for epilepsy are unknown. The androgenic potency of A is relatively weak, and typical clinical signs of hyperandrogenism were not seen.  ▪ The long-term health consequences of these reproductive endocrine changes during pubertal development remain to be established. | **▪** Small sample – wide confidence intervals  **▪** Exclusions not fully defined  **▪** Lack of PWE untreated makes it difficult to differentiate between effect of ASMs vs. epilepsy on outcomes  **▪** Not a causal design: inferences about associations alone are permissible  **▪** The study of ASM monotherapy makes this less generalisable to a real-world epilepsy population, many of whom will be on ASM polytherapy | IV |
| Røste 2003 – Norway^9^ | Case-control | **▪** 36 men with epilepsy: 16 on VPA, 20 on CBZ monotherapy  **▪** Age-limits 20-40 years, on VPA or CBZ for >2 years | 90 healthy fertile men | **▪** No history of drug or alcohol abuse, steroid use, testicular surgery, varicocele or genitourinary disease, endocrine dysfunction, testicular abnormalities or infertility problems prior to their epilepsy and start of ASMs | VPA exposure 🡪 control  **▪ 🡫 Rapidly progressive motile sperm (%):** 24.56 🡪 35.21 (*p* = 0.005)  **▪ 🡩 Sperm head abnormalities (%):** 98 🡪 95.51 (*p* < 0.001)  **▪ 🡫 Sperm neck abnormalities (%):** 53.88 🡪 63.38 (*p* < 0.001)  **▪ 🡩 Sperm tail abnormalities (%):** 11.31🡪 7.06 (*p* < 0.006)  **▪ No change:** testicular volume, semen volume, sperm concentration, pH  Other results  **▪ 🡫 Testicular volume corrected for BMI (testicular size ÷ BMI mean):** VPA (mean 0.63) vs. CBZ (mean 0.74) – but not absolute testicular volumes (ml, VPA mean 17.3, CBZ mean 18.6)  **▪ No change VPA vs. CBZ groups:** semen parameters, sexual function, pregnancies fathered  **▪** PWE vs. controls had **🡫** rapidly progressive motile sperm (*p* < 0.001), **🡩** sperm head abnormalities (*p* < 0.001), **🡫** sperm neck abnormalities, and no change in remaining parameters | ▪ Men with epilepsy treated with either CBZ or VPA as monotherapy, had altered semen quality compared with controls regarding a few, but important semen parameters of sperm motility and morphology.  ▪ No significant differences between the patient groups regarding semen parameters, testicular size, fertility or reported libido/potency were observed.  ▪ Semen from VPA-treated patients had, as opposed to CBZ-treated, significantly increased amount of sperm-tail abnormalities compared with controls. | **▪** Small sample  **▪** Exclusions limit generalisability to real-world pool of patients  **▪** Not a causal design: inferences about associations alone are permissible  **▪** Lack of PWE untreated makes it difficult to differentiate between effect of ASMs vs. epilepsy on outcomes  **▪** Could not control for all relevant confounders, such as lifestyle (smoking, alcohol)  **▪** The study of ASM monotherapy makes this less generalisable to a real-world epilepsy population, many of whom will be on ASM polytherapy | IV |
| Rättyä 2001 – Finland^10^ | Case-control | **▪** 90 men with epilepsy on VPA (n = 21), CBZ (n = 40), or OXC (n = 29) monotherapy  **▪** Age-limits 18-50 years, no minimum treatment duration specified | 25 healthy men | **▪** Patients with diseases other than epilepsy, patients who were taking regular medication in addition to ASMs, or receiving polytherapy for epilepsy were excluded. | VPA exposure 🡪 control  **▪ 🡩 Androstenedione** (p < 0.001)  **▪ 🡫 FSH** (p < 0.05)  **▪ 🡫 Progesterone** (p < 0.05)  **▪ 🡩 Serum insulin** (p < 0.01)  **▪ No change:** testosterone, DHEAS, LH, progesterone, SHBG, IGF-I, IGFBP-1, IGFBP-3 | ▪ All ASMs studied were associated with noticeable changes in the reproductive endocrine function.  ▪ Increased serum androgen levels were found in close to 60% of the men taking VPA.  ▪ CBZ had an opposite effect on the androgen balance; serum levels of DHEAS were low, and SHBG concentrations were high.  ▪ 18% of men taking CBZ reported decreased libido, impaired potency, or both.  ▪ Low daily OXC doses did not have any effects on serum concentrations of reproductive hormones, but men taking high doses of OXC had increased serum testosterone, gonadotropin, and SHBG levels.  ▪ Serum insulin levels were high in all patient groups. | **▪** Small sample  **▪** Unmatched and no accounting for confounders  **▪** Lack of PWE untreated makes it difficult to differentiate between effect of ASMs vs. epilepsy on outcomes  **▪** Not a causal design: inferences about associations alone are permissible  **▪** The study of ASM monotherapy makes this less generalisable to a real-world epilepsy population, many of whom will be on ASM polytherapy | IV |
| Xiaotian 2013 – China^11^ | Case-control | **▪** Men with epilepsy: 32 on VPA monotherapy, 20 on LEV monotherapy or add-on  **▪** Age-limits 18-48 years, on VPA for >6 months | 30 health men | **▪** A history of drug or alcohol abuse, hormone therapy, testicular surgery, varicocele, urogenital disease, severe psychiatric disorder, hypothyroidism, hyperthyroidism, or disorders that might alter epilepsy or reproductive system function. In addition, patients with endocrine dysfunction, testicular deformity, or who had used antidepressants or drugs to improve erectile dysfunction were excluded from this study. | VPA exposure 🡪 control  **▪ 🡩 Frequency of morphologically abnormal sperm (%):** 69.20 🡪 16.00 (p < 0.01)  **▪ 🡫 Frequency of motile sperm (%):** 40.88 🡪 72.72 (p < 0.01)  **▪ 🡫 <25% of sperm in motile grade A:** 19.39 🡪 38.39 (p < 0.01)  **▪ 🡫 <50% of sperm in motile grades A+B:** 28.48 🡪 63.09 (p < 0.01)  **▪ 🡩 Frequency of morphologically abnormal sperm heads:** 61.46 🡪 11.80 (p < 0.01)  **▪ 🡩 Frequency of morphologically abnormal sperm midpieces:** 13.13 🡪 4.20 (p < 0.01)  **▪ 🡩 Frequency of morphologically abnormal sperm tails:** 12.13 🡪 2.67 (p < 0.01)  **▪ 🡫 LH** (p < 0.01)  **▪ 🡫 FSH** (p < 0.01)  **▪ 🡩 BAT/LH ratio** (p < 0.01)  **▪ 🡩 Prolactin** (p < 0.01)  **▪ 🡫 Total IIEF-5 scores** (p < 0.01)  **▪ No change:** Sperm concentration, testosterone, estradiol, BAT/BAE | ▪ Sperm motility rate in both LEV and VPA treatment groups was lower than in healthy controls.  ▪ The rate of abnormal sperm morphology was significantly higher in both LEV and VPA treatment groups than in the control group, especially with regard to sperm head deformity.  ▪ Sexual function scores were significantly lower in VPA and LEV groups than in the control group, suggesting sexual dysfunction in men with epilepsy.  ▪ LH and FSH levels were significantly lower in the VPA group than in the control, and BAT/LH ratio and the prolactin were significantly higher – regarded as adverse effects on reproductive endocrine function. | **▪** Small sample  ▪ The results of this study are based on comparisons between PWE and healthy controls, the effects of confounding factors related to the epilepsy itself may cause deviation of the study results in other populations.  **▪** Exclusions limit generalisability to real-world pool of patients  **▪** Unmatched and no accounting for confounders  **▪** Not a causal design: inferences about associations alone are permissible  **▪** The study of ASM monotherapy makes this less generalisable to a real-world epilepsy population, many of whom will be on ASM polytherapy | IV |
| Hayashi 2004 – Japan^12^ | Case report | **▪** 2 men with epilepsy on VPA  **▪** No age or minimum treatment limits | None | **▪** None | Case 1: VPA 🡪 PHT  **▪ 🡫 Sperm motility (%):** 6 🡪 64–74  **▪ 🡫 Sperm count (×10^6^):** 3 🡪 65–81  **▪** Infertility for 5 years 🡪 conceived child after 15 months  Case 2: VPA 🡪 PHT  **▪ 🡫 Sperm motility (%):** 5 🡪 65–77  **▪ 🡫 Sperm count (×10^6^):** 3 🡪 59–74  **▪** Infertility for 7 years 🡪 conceived child after 15 months | ▪ Suggest a possible association between oligoasthenozoospermia and VPA.  ▪ The fact that complete reversal of spermatic dysfunction followed by a successful conception was achieved after discontinuation of VPA suggests the drug was responsible for spermatic dysfunction in these individuals. | ▪ Case report – uncontrolled and potentially confounded, difficult to generalise or infer causality | V |
| Yerby 1999 – USA^13^ | Case report | **▪** 1 man with epilepsy on VPA monotherapy  **▪** 32 years old, on VPA for at least 2 years | None | **▪** None | VPA 🡪 felbamate  **▪ 🡫 Sperm count (per ml):** 144,430 🡪 16,900,000  **▪ 🡫 Sperm motility immediate:** none 🡪 50%  **▪ 🡫 Sperm motility 3-hour:** none 🡪 30%  **▪ 🡩 Abnormal forms:** 100% 🡪 72%  **▪ 🡫 Viability**: 10% 🡪 78%  **▪ One successful conception** on VPA + PHT  **▪ Unable to conceive subsequently on VPA** | ▪ This happy outcome may be simply fortuitous. It is clear that many persons are successfully treated with VPA and are not infertile.  ▪ However, the patient was unable to conceive while taking high doses of VPA (3,500 mg/day, with levels consistently >lo0 p,g/ml) and had marked reductions in sperm numbers and motility.  ▪ Within 2 months after discontinuation of VPA, a complete reversal of the spermatic dysfunction and a successful conception make it appear that VPA in this individual was responsible for spermatic dysfunction. | ▪ Case report – uncontrolled and potentially confounded, difficult to generalise or infer causality | V |
| Duncan 1999 – UK^14^ | Case-control | **▪** Men with epilepsy: 18 on VPA, 31 on CBZ, 21 on PHT, 48 on polytherapy, 32 untreated  **▪** Age-limits 18-65 years, no minimum treatment duration specified | 33 healthy controls | **▪** Not specified | VPA exposure 🡪 control  **▪ No change:** SHBG, total testosterone, free testosterone, free androgen index, dehydroepiandosterone sulphate, estradiol, androstenedione | Significant alteration in sex hormones relative to control occurred only in men with epilepsy taking CBZ, PHT, or polytherapy. | **▪** Small sample  **▪** Men had no other medical condition, limiting generalisability  **▪** Unmatched, and confounders such as lifestyle (alcohol, smoking), not accounted for  **▪** Not a causal design: inferences about associations alone are permissible | IV |
| Aldemir 2012 – Turkey^15^ | Cross-sectional | **▪** 39 men with bipolar disorder: (21 on lithium monotherapy and 18 on VPA monotherapy or VPA in combination with lithium) and 15 men with epilepsy on VPA monotherapy  **▪** Age-limits 18-50 years, on ≥3 months of lithium and/or VPA | None | **▪** A history of alcohol or substance use, testicular surgery or varicocele, endocrine or genitourinary diseases, pre-existing testicular abnormalities or infertility, epileptic seizures in the last month, or the use of any hormonal medication or psychotropic drugs other than lithium and VPA. | **▪ 🡩 Prolactin** in PWE on VPA vs. bipolar on lithium (p = 0.008)  **▪ 🡩 FSH** in PWE on VPA vs. bipolar on lithium (p = 0.043)  **▪ No change:** estradiol, LH, SHBG, free testosterone, free testosterone:LH ratio | Valproate did not have a negative effect on male reproductive hormones in the bipolar patients. The elevated prolactin and follicle-stimulating hormone levels observed in the epilepsy group should be attributed to epilepsy. | **▪** Small sample  **▪** Exclusions limit generalisability to real-world pool of patients  **▪** 1-month seizure-freedom less generalisable to a general epilepsy population, who may not be seizure-free over that period  **▪** Lack of PWE/bipolar disorder untreated makes it difficult to differentiate between effect of ASMs vs. disease on outcomes  **▪** The study of ASM monotherapy makes this less generalisable to a real-world epilepsy population, many of whom will be on ASM polytherapy  **▪** Unmatched, and confounders such as lifestyle (alcohol, smoking), not accounted for  **▪** Not a causal design: inferences about associations alone are permissible  **▪** 3-month minimum treatment period is short – less generalisable to long-term treatment | IV |
| Aldemir 2012 – Turkey^16^ | Cross-sectional | **▪** 6 men with bipolar disorder on lithium monotherapy  **▪** 5 men with bipolar disorder on VPA monotherapy or lithium and VPA,  **▪** 1 man with epilepsy on VPA monotherapy  **▪** Age-limits 18-50 years, on ≥3 months of lithium and/or VPA | None | Not specified | **▪ No change in sperm abnormality:** bipolar disorder on lithium vs. bipolar disorder on valproate  **▪ No change:** sperm count or motility in bipolar disorder on lithium only  **▪ 🡫 sperm count:** 2 men with bipolar disorder on valproate or lithium and valproate  **▪ 🡫 sperm motility:** 2 men with bipolar disorder on valproate or lithium and valproate  **▪ Impaired sperm morphology:** 3 men with bipolar disorder on lithium and 5 men with bipolar disorder on valproate or lithium and valproate  **▪ 🡫 sperm motility:** 1 man with epilepsy on VPA | **▪** Some alterations in semen parameters on valproate or lithium - results were not statistically significant | **▪** Small sample  **▪** Uncontrolled and unmatched – confounders not accounted for and difficult to disentangle whether any effect, if present, is related to valproate, lithium, bipolar disorder or epilepsy | IV |
| Kose-Ozlece 2015 – Turkey^17^ | Case report | **▪** 1 man with epilepsy on VPA monotherapy  **▪** 34 years old, on treatment for 6 years | None | **▪** None | VPA dose 500 mg 🡪 1000 mg 🡪 1500 mg 🡪 stopped VPA + commenced LTG  **▪ 🡫 Sperm count (×10^6^):** 87 🡪 2 🡪 5.1 🡪 91.2  **▪ 🡫 Sperm viability (%):** 55 🡪 20 🡪 20 🡪 70  **▪ 🡫 Progressive motility (%):** 25 🡪 5 🡪 5 🡪 35  **▪ 🡫 Total motility (%):** 42 🡪 10 🡪 10 🡪 60  **▪ 🡫 Normal morphology (%):** 8 🡪 1 🡪 0 🡪 28  **▪ 🡫 FSH**  **▪ 🡫 LH**  **▪ 🡩 dehydroepiandrosterone**  **▪ 🡩 testosterone**  **▪** Infertility for 3 years 🡪 conceived child 15 month after treatment modification | **▪** VPA-dependent impairments in the hormone and semen analysis parameters were reversible after the termination of treatment.  **▪** VPA cessation did not cause permanent hormonal deregulation and these side effects are dose dependent. | ▪ Case report – uncontrolled and potentially confounded, difficult to generalise or infer causality | V |
| Tallon 2021 – Ireland^18^ | Case report | **▪** 1 man with epilepsy on VPA and LTG  **▪** 34 years old, on treatment for 16 years | None | None | VPA and LTG 🡪 LTG and CLOB 🡪 VPA and LTG  **▪ 🡫 Sperm concentration (×10^6^/ml):** 9.5–13🡪 24🡪 40.5–56  **▪ 🡫 Sperm motility (%):** 27–32🡪 30🡪 42–50  **▪ 🡫 Normal morphology (%):** 1🡪 3🡪 2–4  **▪ No change:** Semen volume  **▪** Infertility for 6 months 🡪 conception occurred eight months following VPA cessation | **▪** Suggests VPA-induced infertility, that is dose-dependent, with a relationship between duration of treatment and progressive deterioration in sperm parameters, namely morphology. | ▪ Case report – uncontrolled and potentially confounded, difficult to generalise or infer causality | V |
| Curtis 1994 – USA^19^ | Case report | **▪** 1 man with epilepsy on VPA monotherapy  **▪** 39 years old, on treatment for 5 years | None | None | VPA 🡪 CBZ  **▪ 🡫 Sperm count (×10^6^/ml):** 18.4 🡪 157.5  **▪ 🡫 Sperm motility (%):** 4–8.4% 🡪 65%  **▪ 🡫 Morphology (%):** 4–5% 🡪 34% (oval sperm)  **▪** Infertility for 1 year 🡪 conception occurred sometime following VPA cessation | **▪** Spermatozoal function was impaired without effect on endocrine function. | ▪ Case report – uncontrolled and potentially confounded, difficult to generalise or infer causality | V |

**Abbreviations:** VPA = Valproic acid; LEV = Levetiracetam; IIEF-5 = Erectile Function-5; FSH = Follicle stimulating hormone; LH = Luteinising hormone; ERC = Excess residual cytoplasm; TZI = Teratozoospermia index; BAE = bioactive estrogen; BAT = bioactive testosterone; CBZ = carbamazepine, DHEAS = dehydroepiandrostenedione sulfate; SHBG = sex hormone-binding globulin; TSH = Thyroid stimulating hormone; ASM = antiseizure medication; FAI = 100 x testosterone/SHBG; ***** = Gravely abnormal sperm morphology; OR = Odds ratio; CI = Confidence interval; BMI – Body mass index; IGF-I = Insulin-like growth factor I, IGFBP-1 and -3 = insulin-like growth factor binding proteins 1 and 3; BAT = bioactive testosterone; BAE = bioactive estrogen; PHT = phenytoin; CLOB = clobazam

## References

1 OCEBM Levels of Evidence Working Group. The Oxford 2011 Levels of Evidence. (2011). <file:///Users/s1676656/Downloads/CEBM-Levels-of-Evidence-2.1.pdf>.

2 Guo, Y. *et al.* A Comparative Study of the Effects of Valproate and Oxcarbazepine on Sexual Function, Sperm Quality, and Sex Hormones in Males with Epilepsy. *Biomed Res Int* **2021**, 6624101, doi:10.1155/2021/6624101 (2021).

3 Markoula, S., Siarava, E., Kostoulas, C., Zikopoulos, A. & Georgiou, I. An open study of valproate in subfertile men with epilepsy. *Acta Neurol Scand* **142**, 317-322, doi:10.1111/ane.13311 (2020).

4 Ocek, L. *et al.* Evaluation of sex hormones and sperm parameters in male epileptic patients. *Acta Neurol Scand* **137**, 409-416, doi:10.1111/ane.12892 (2018).

5 Hamed, S. A. *et al.* Seminal fluid analysis and testicular volume in adults with epilepsy receiving valproate. *J Clin Neurosci* **22**, 508-512, doi:10.1016/j.jocn.2014.08.029 (2015).

6 Artama, M., Isojarvi, J. I. & Auvinen, A. Antiepileptic drug use and birth rate in patients with epilepsy--a population-based cohort study in Finland. *Hum Reprod* **21**, 2290-2295, doi:10.1093/humrep/del194 (2006).

7 Isojarvi, J. I. *et al.* Effect of epilepsy and antiepileptic drugs on male reproductive health. *Neurology* **62**, 247-253, doi:10.1212/01.wnl.0000098936.46730.64 (2004).

8 Mikkonen, K. *et al.* Serum androgen levels and testicular structure during pubertal maturation in male subjects with epilepsy. *Epilepsia* **45**, 769-776, doi:10.1111/j.0013-9580.2004.04604.x (2004).

9 Roste, L. S. *et al.* Alterations in semen parameters in men with epilepsy treated with valproate or carbamazepine monotherapy. *Eur J Neurol* **10**, 501-506, doi:10.1046/j.1468-1331.2003.00615.x (2003).

10 Rattya, J. *et al.* Reproductive effects of valproate, carbamazepine, and oxcarbazepine in men with epilepsy. *Neurology* **56**, 31-36, doi:10.1212/wnl.56.1.31 (2001).

11 Xiaotian, X. *et al.* Effects of antiepileptic drugs on reproductive endocrine function, sexual function and sperm parameters in Chinese Han men with epilepsy. *J Clin Neurosci* **20**, 1492-1497, doi:10.1016/j.jocn.2012.11.028 (2013).

12 Hayashi, T. *et al.* Improvement of oligoasthenozoospermia in epileptic patients on switching anti-epilepsy medication from sodium valproate to phenytoin. *Scand J Urol Nephrol* **39**, 431-432, doi:10.1080/00365590500252688 (2005).

13 Yerby, M. S. & McCoy, G. B. Male infertility: possible association with valproate exposure. *Epilepsia* **40**, 520-521, doi:10.1111/j.1528-1157.1999.tb00752.x (1999).

14 Duncan, S., Blacklaw, J., Beastall, G. H. & Brodie, M. J. Antiepileptic drug therapy and sexual function in men with epilepsy. *Epilepsia* **40**, 197-204, doi:10.1111/j.1528-1157.1999.tb02075.x (1999).

15 Aldemir, E. *et al.* Valproate-associated reproductive hormone abnormalities: do bipolar men have the same risk as epileptic men? *Turk Psikiyatri Derg* **23**, 223-227 (2012).

16 Aldemir, E. *et al.* Abnormal semen parameters in bipolar men treated with valproate. *Turk Psikiyatri Derg* **23**, 145-146 (2012).

17 Kose-Ozlece, H., Ilik, F., Cecen, K., Huseyinoglu, N. & Serim, A. Alterations in semen parameters in men with epilepsy treated with valproate. *Iran J Neurol* **14**, 164-167 (2015).

18 Tallon, E., O'Donovan, L. & Delanty, N. Reversible male infertility with valproate use: A review of the literature. *Epilepsy Behav Rep* **16**, 100446, doi:10.1016/j.ebr.2021.100446 (2021).

19 Curtis, V. L., Oelberg, D. G. & Willmore, L. J. Infertility secondary to valproate. *Journal of Epilepsy* **7**, 259-261, doi:<https://doi.org/10.1016/0896-6974(94)90053-1> (1994).

# Supplementary Table 2: Propensity score matching men with epilepsy or bipolar disorder exposed (cohort 1) and unexposed (cohort 2) to valproate – lifetime outcome assessment

Propensity score matching was performed on all listed characteristics. Characteristics of the cohorts before and after matching are summarized in the table below.

| **Cohort 1 and cohort 2 patient count before and after propensity score matching** | | | | | | | | | | | | |
| --- | --- | --- | --- | --- | --- | --- | --- | --- | --- | --- | --- | --- |
|  | | | Cohort | | | Patient count before matching | | | | Patient count after matching | | |
|  | | | 1 - Men with epilepsy or bipolar disorder exposed to valproate_v9 | | | 91,917 | | | | 78,971 | | |
|  | | | 2 - Men with epilepsy or bipolar disorder not exposed to valproate_v9 | | | 535,803 | | | | 78,971 | | |
| **Propensity score density function - Before and after matching (cohort 1 - purple, cohort 2 - green)** | | | | | | | | | | | | |
|  |  | | 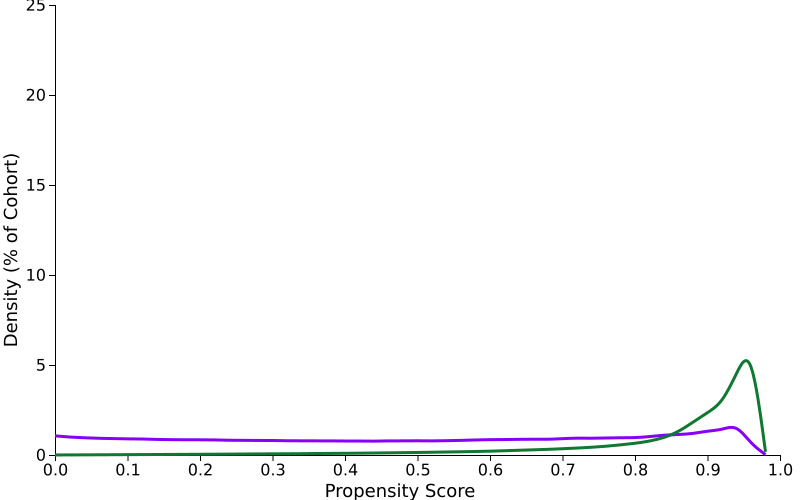 | | | | 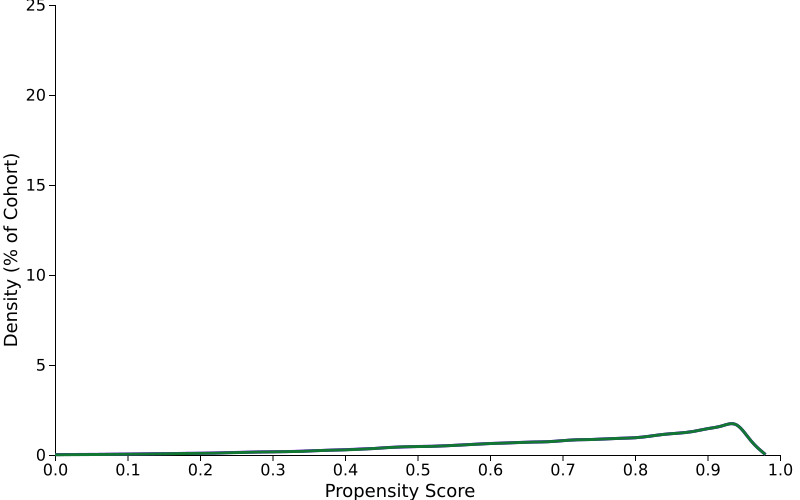 | | | | | |
| **Cohort 1 (N = 91,917) and cohort 2 (N = 535,803) characteristics before propensity score matching** | | | | | | | | | | | | |
|  | **Demographics** | | | | | | | | | | | |
|  |  | Cohort | | |  | Mean ± SD | | Patients | % of Cohort | | P-Value | SMD |
|  |  | 1 2 | | AI | Age at Index | 25.0 +/- 12.9 21.2 +/- 14.9 | | 89,409 514,650 | 100% 100% | | <0.001 | 0.277 |
|  |  | 1 2 | | 2106-3 | White |  | | 52,140 294,663 | 58.3% 57.3% | | <0.001 | 0.021 |
|  |  | 1 2 | | 1002-5 | American Indian or Alaska Native |  | | 357 1,784 | 0.4% 0.3% | | 0.014 | 0.009 |
|  |  | 1 2 | | UNK | Unknown Race |  | | 11,628 80,049 | 13.0% 15.6% | | <0.001 | 0.073 |
|  |  | 1 2 | | 2076-8 | Native Hawaiian or Other Pacific Islander |  | | 352 2,395 | 0.4% 0.5% | | 0.003 | 0.011 |
|  |  | 1 2 | | 2054-5 | Black or African American |  | | 16,497 88,712 | 18.5% 17.2% | | <0.001 | 0.032 |
|  |  | 1 2 | | 2131-1 | Other Race |  | | 4,772 30,672 | 5.3% 6.0% | | <0.001 | 0.027 |
|  |  | 1 2 | | 2028-9 | Asian |  | | 3,663 16,375 | 4.1% 3.2% | | <0.001 | 0.049 |
|  | **Diagnosis** | | | | | | | | | | | |
|  |  | Cohort | | |  | Mean ± SD | | Patients | % of Cohort | | P-Value | SMD |
|  |  | 1 2 | | F80-F89 | Pervasive and specific developmental disorders |  | | 14,309 47,247 | 16.0% 9.2% | | <0.001 | 0.207 |
|  |  | 1 2 | | F40-F48 | Anxiety, dissociative, stress-related, somatoform and other nonpsychotic mental disorders |  | | 25,097 89,873 | 28.1% 17.5% | | <0.001 | 0.255 |
|  |  | 1 2 | | F90-F98 | Behavioral and emotional disorders with onset usually occurring in childhood and adolescence |  | | 15,976 51,855 | 17.9% 10.1% | | <0.001 | 0.226 |
|  |  | 1 2 | | F20-F29 | Schizophrenia, schizotypal, delusional, and other non-mood psychotic disorders |  | | 14,270 27,326 | 16.0% 5.3% | | <0.001 | 0.351 |
|  |  | 1 2 | | F60-F69 | Disorders of adult personality and behavior |  | | 7,454 13,845 | 8.3% 2.7% | | <0.001 | 0.249 |
|  |  | 1 2 | | F70-F79 | Intellectual Disabilities |  | | 6,625 9,796 | 7.4% 1.9% | | <0.001 | 0.264 |
|  |  | 1 2 | | F01-F09 | Mental disorders due to known physiological conditions |  | | 4,714 8,596 | 5.3% 1.7% | | <0.001 | 0.198 |
|  |  | 1 2 | | F99-F99 | Unspecified mental disorder (F99) |  | | 2,556 4,891 | 2.9% 1.0% | | <0.001 | 0.140 |
|  |  | 1 2 | | E08-E13 | Diabetes mellitus |  | | 4,521 18,978 | 5.1% 3.7% | | <0.001 | 0.067 |
|  |  | 1 2 | | Q00-Q07 | Congenital malformations of the nervous system |  | | 3,239 10,059 | 3.6% 2.0% | | <0.001 | 0.101 |
|  |  | 1 2 | | Q60-Q64 | Congenital malformations of the urinary system |  | | 560 3,018 | 0.6% 0.6% | | 0.151 | 0.005 |
|  |  | 1 2 | | N45 | Orchitis and epididymitis |  | | 493 1,977 | 0.6% 0.4% | | <0.001 | 0.025 |
|  |  | 1 2 | | N43 | Hydrocele and spermatocele |  | | 575 2,846 | 0.6% 0.6% | | 0.001 | 0.012 |
|  |  | 1 2 | | N50.1 | Vascular disorders of male genital organs |  | | 44 152 | 0.0% 0.0% | | 0.003 | 0.010 |
|  |  | 1 2 | | N49 | Inflammatory disorders of male genital organs, not elsewhere classified |  | | 146 589 | 0.2% 0.1% | | <0.001 | 0.013 |
|  |  | 1 2 | | I86.1 | Scrotal varices |  | | 241 1,111 | 0.3% 0.2% | | 0.002 | 0.011 |
|  |  | 1 2 | | Q53 | Undescended and ectopic testicle |  | | 578 2,847 | 0.6% 0.6% | | 0.001 | 0.012 |
|  |  | 1 2 | | Q55 | Other congenital malformations of male genital organs |  | | 393 2,601 | 0.4% 0.5% | | 0.010 | 0.010 |
|  |  | 1 2 | | Q54 | Hypospadias |  | | 219 1,532 | 0.2% 0.3% | | 0.007 | 0.010 |
|  |  | 1 2 | | C60-C63 | Malignant neoplasms of male genital organs |  | | 98 593 | 0.1% 0.1% | | 0.647 | 0.002 |
|  |  | 1 2 | | N44.8 | Other noninflammatory disorders of the testis |  | | 251 727 | 0.3% 0.1% | | <0.001 | 0.030 |
|  |  | 1 2 | | N44.0 | Torsion of testis |  | | 62 318 | 0.1% 0.1% | | 0.406 | 0.003 |
|  |  | 1 2 | | N30-N39 | Other diseases of the urinary system |  | | 4,984 17,057 | 5.6% 3.3% | | <0.001 | 0.110 |
|  |  | 1 2 | | K40 | Inguinal hernia |  | | 910 5,081 | 1.0% 1.0% | | 0.395 | 0.003 |
|  |  | 1 2 | | E84 | Cystic fibrosis |  | | 74 350 | 0.1% 0.1% | | 0.124 | 0.005 |
|  |  | 1 2 | | S30-S39 | Injuries to the abdomen, lower back, lumbar spine, pelvis and external genitals |  | | 5,442 20,080 | 6.1% 3.9% | | <0.001 | 0.100 |
|  |  | 1 2 | | E23.0 | Hypopituitarism |  | | 392 1,533 | 0.4% 0.3% | | <0.001 | 0.023 |
|  |  | 1 2 | | R56 | Convulsions, not elsewhere classified |  | | 32,649 250,954 | 36.5% 48.8% | | <0.001 | 0.250 |
|  |  | 1 2 | | E34.5 | Androgen insensitivity syndrome |  | | 10 15 | 0.0% 0.0% | | <0.001 | 0.010 |
|  |  | 1 2 | | Q87.1 | Congenital malformation syndromes predominantly associated with short stature |  | | 94 371 | 0.1% 0.1% | | 0.001 | 0.011 |
|  |  | 1 2 | | F31 | Bipolar disorder |  | | 26,093 127,285 | 29.2% 24.7% | | <0.001 | 0.100 |
|  |  | 1 2 | | F32 | Depressive episode |  | | 15,420 51,702 | 17.2% 10.0% | | <0.001 | 0.211 |
|  |  | 1 2 | | F39 | Unspecified mood [affective] disorder |  | | 7,197 13,456 | 8.0% 2.6% | | <0.001 | 0.244 |
|  |  | 1 2 | | F33 | Major depressive disorder, recurrent |  | | 4,601 14,749 | 5.1% 2.9% | | <0.001 | 0.116 |
|  |  | 1 2 | | F34 | Persistent mood [affective] disorders |  | | 2,232 5,488 | 2.5% 1.1% | | <0.001 | 0.108 |
|  |  | 1 2 | | F30 | Manic episode |  | | 2,346 2,891 | 2.6% 0.6% | | <0.001 | 0.165 |
|  |  | 1 2 | | F50-F59 | Behavioral syndromes associated with physiological disturbances and physical factors |  | | 2,888 8,800 | 3.2% 1.7% | | <0.001 | 0.098 |
|  |  | 1 2 | | J00-J99 | Diseases of the respiratory system |  | | 29,838 152,303 | 33.4% 29.6% | | <0.001 | 0.081 |
|  |  | 1 2 | | I00-I99 | Diseases of the circulatory system |  | | 22,366 95,406 | 25.0% 18.5% | | <0.001 | 0.157 |
|  |  | 1 2 | | K00-K95 | Diseases of the digestive system |  | | 26,716 123,133 | 29.9% 23.9% | | <0.001 | 0.135 |
|  |  | 1 2 | | Q65-Q79 | Congenital malformations and deformations of the musculoskeletal system |  | | 2,911 15,214 | 3.3% 3.0% | | <0.001 | 0.017 |
|  |  | 1 2 | | N17-N19 | Acute kidney failure and chronic kidney disease |  | | 4,399 17,871 | 4.9% 3.5% | | <0.001 | 0.072 |
|  |  | 1 2 | | A50-A64 | Infections with a predominantly sexual mode of transmission |  | | 1,046 4,470 | 1.2% 0.9% | | <0.001 | 0.030 |
|  |  | 1 2 | | E03 | Other hypothyroidism |  | | 2,726 8,221 | 3.0% 1.6% | | <0.001 | 0.096 |
|  |  | 1 2 | | E05 | Thyrotoxicosis [hyperthyroidism] |  | | 381 1,146 | 0.4% 0.2% | | <0.001 | 0.036 |
|  |  | 1 2 | | E83.1 | Disorders of iron metabolism |  | | 101 583 | 0.1% 0.1% | | 0.979 | <0.001 |
|  |  | 1 2 | | Q99 | Other chromosome abnormalities, not elsewhere classified |  | | 1,077 2,907 | 1.2% 0.6% | | <0.001 | 0.068 |
|  |  | 1 2 | | Q93 | Monosomies and deletions from the autosomes, not elsewhere classified |  | | 489 1,601 | 0.5% 0.3% | | <0.001 | 0.036 |
|  |  | 1 2 | | Q90 | Down syndrome |  | | 337 1,776 | 0.4% 0.3% | | 0.137 | 0.005 |
|  |  | 1 2 | | Q92 | Other trisomies and partial trisomies of the autosomes, not elsewhere classified |  | | 198 540 | 0.2% 0.1% | | <0.001 | 0.029 |
|  |  | 1 2 | | Q98.4 | Klinefelter syndrome, unspecified |  | | 83 230 | 0.1% 0.0% | | <0.001 | 0.018 |
|  |  | 1 2 | | Q98.5 | Karyotype 47, XYY |  | | 31 94 | 0.0% 0.0% | | 0.002 | 0.010 |
|  |  | 1 2 | | Q98.7 | Male with sex chromosome mosaicism |  | | 31 59 | 0.0% 0.0% | | <0.001 | 0.015 |
|  |  | 1 2 | | Q98.8 | Other specified sex chromosome abnormalities, male phenotype |  | | 31 68 | 0.0% 0.0% | | <0.001 | 0.014 |
|  |  | 1 2 | | Q98.0 | Klinefelter syndrome karyotype 47, XXY |  | | 17 54 | 0.0% 0.0% | | 0.030 | 0.007 |
|  |  | 1 2 | | Q98.1 | Klinefelter syndrome, male with more than two X chromosomes |  | | 10 15 | 0.0% 0.0% | | <0.001 | 0.010 |
|  |  | 1 2 | | Q98.6 | Male with structurally abnormal sex chromosome |  | | 10 10 | 0.0% 0.0% | | <0.001 | 0.011 |
|  |  | 1 2 | | Q98.9 | Sex chromosome abnormality, male phenotype, unspecified |  | | 10 10 | 0.0% 0.0% | | <0.001 | 0.011 |
|  |  | 1 2 | | D35.2 | Benign neoplasm of pituitary gland |  | | 105 399 | 0.1% 0.1% | | <0.001 | 0.013 |
|  |  | 1 2 | | D35.3 | Benign neoplasm of craniopharyngeal duct |  | | 41 157 | 0.0% 0.0% | | 0.019 | 0.008 |
|  |  | 1 2 | | E40-E46 | Malnutrition |  | | 2,270 7,683 | 2.5% 1.5% | | <0.001 | 0.074 |
|  |  | 1 2 | | G40.9 | Epilepsy, unspecified |  | | 31,387 99,615 | 35.1% 19.4% | | <0.001 | 0.359 |
|  |  | 1 2 | | G40.3 | Generalized idiopathic epilepsy and epileptic syndromes |  | | 10,855 15,222 | 12.1% 3.0% | | <0.001 | 0.353 |
|  |  | 1 2 | | G40.4 | Other generalized epilepsy and epileptic syndromes |  | | 8,950 13,086 | 10.0% 2.5% | | <0.001 | 0.312 |
|  |  | 1 2 | | G40.2 | Localization-related (focal) (partial) symptomatic epilepsy and epileptic syndromes with complex partial seizures |  | | 8,191 19,536 | 9.2% 3.8% | | <0.001 | 0.219 |
|  |  | 1 2 | | G40.8 | Other epilepsy and recurrent seizures |  | | 6,655 17,481 | 7.4% 3.4% | | <0.001 | 0.179 |
|  |  | 1 2 | | G40.1 | Localization-related (focal) (partial) symptomatic epilepsy and epileptic syndromes with simple partial seizures |  | | 6,325 14,705 | 7.1% 2.9% | | <0.001 | 0.195 |
|  |  | 1 2 | | G40.A | Absence epileptic syndrome |  | | 3,238 5,177 | 3.6% 1.0% | | <0.001 | 0.175 |
|  |  | 1 2 | | G40.0 | Localization-related (focal) (partial) idiopathic epilepsy and epileptic syndromes with seizures of localized onset |  | | 2,083 4,866 | 2.3% 0.9% | | <0.001 | 0.109 |
|  |  | 1 2 | | G40.5 | Epileptic seizures related to external causes |  | | 1,249 2,624 | 1.4% 0.5% | | <0.001 | 0.091 |
|  |  | 1 2 | | G40.B | Juvenile myoclonic epilepsy [impulsive petit mal] |  | | 924 768 | 1.0% 0.1% | | <0.001 | 0.116 |
|  |  | 1 2 | | F17 | Nicotine dependence |  | | 17,264 65,657 | 19.3% 12.8% | | <0.001 | 0.179 |
|  |  | 1 2 | | F12 | Cannabis related disorders |  | | 11,484 29,898 | 12.8% 5.8% | | <0.001 | 0.244 |
|  |  | 1 2 | | F10 | Alcohol related disorders |  | | 9,242 39,107 | 10.3% 7.6% | | <0.001 | 0.096 |
|  |  | 1 2 | | F19 | Other psychoactive substance related disorders |  | | 7,852 23,131 | 8.8% 4.5% | | <0.001 | 0.173 |
|  |  | 1 2 | | F11 | Opioid related disorders |  | | 4,528 16,742 | 5.1% 3.3% | | <0.001 | 0.091 |
|  |  | 1 2 | | F14 | Cocaine related disorders |  | | 3,953 10,905 | 4.4% 2.1% | | <0.001 | 0.130 |
|  |  | 1 2 | | F15 | Other stimulant related disorders |  | | 3,848 10,586 | 4.3% 2.1% | | <0.001 | 0.128 |
|  |  | 1 2 | | F13 | Sedative, hypnotic, or anxiolytic related disorders |  | | 2,015 5,504 | 2.3% 1.1% | | <0.001 | 0.093 |
|  |  | 1 2 | | F18 | Inhalant related disorders |  | | 1,648 4,465 | 1.8% 0.9% | | <0.001 | 0.084 |
|  |  | 1 2 | | F16 | Hallucinogen related disorders |  | | 898 1,999 | 1.0% 0.4% | | <0.001 | 0.074 |
|  | **Procedure** | | | | | | | | | | | |
|  |  | Cohort | | |  | Mean ± SD | | Patients | % of Cohort | | P-Value | SMD |
|  |  | 1 2 | | 1010843 | Radiation Oncology Treatment |  | | 188 805 | 0.2% 0.2% | | <0.001 | 0.013 |
|  |  | 1 2 | | 1008061 | Surgical Procedures on the Urinary System |  | | 2,667 9,196 | 3.0% 1.8% | | <0.001 | 0.078 |
|  |  | 1 2 | | 1008011 | Repair initial inguinal hernia, age 5 years or older |  | | 129 455 | 0.1% 0.1% | | <0.001 | 0.016 |
|  |  | 1 2 | | 1008470 | Surgical Procedures on the Male Genital System |  | | 906 5,340 | 1.0% 1.0% | | 0.508 | 0.002 |
|  | **Medication** | | | | | | | | | | | |
|  |  | Cohort | | |  | Mean ± SD | | Patients | % of Cohort | | P-Value | SMD |
|  |  | 1 2 | | 25025 | finasteride |  | | 205 876 | 0.2% 0.2% | | <0.001 | 0.013 |
|  |  | 1 2 | | CN750 | LITHIUM SALTS |  | | 4,044 7,630 | 4.5% 1.5% | | <0.001 | 0.179 |
|  |  | 1 2 | | 6135 | ketoconazole |  | | 1,382 5,039 | 1.5% 1.0% | | <0.001 | 0.051 |
|  |  | 1 2 | | 10829 | trimethoprim |  | | 5,407 21,179 | 6.0% 4.1% | | <0.001 | 0.088 |
|  |  | 1 2 | | 7454 | nitrofurantoin |  | | 450 1,255 | 0.5% 0.2% | | <0.001 | 0.043 |
|  |  | 1 2 | | AM200 | ERYTHROMYCINS/MACROLIDES |  | | 8,294 32,697 | 9.3% 6.4% | | <0.001 | 0.109 |
|  |  | 1 2 | | AM300 | AMINOGLYCOSIDES |  | | 4,458 14,816 | 5.0% 2.9% | | <0.001 | 0.109 |
|  |  | 1 2 | | AN000 | ANTINEOPLASTICS |  | | 1,524 6,591 | 1.7% 1.3% | | <0.001 | 0.035 |
|  |  | 1 2 | | CN709 | ANTIPSYCHOTICS,OTHER |  | | 35,880 62,087 | 40.1% 12.1% | | <0.001 | 0.674 |
|  |  | 1 2 | | CN701 | PHENOTHIAZINE/RELATED ANTIPSYCHOTICS |  | | 2,919 2,243 | 3.3% 0.4% | | <0.001 | 0.211 |
|  |  | 1 2 | | CN609 | ANTIDEPRESSANTS,OTHER |  | | 27,030 65,048 | 30.2% 12.6% | | <0.001 | 0.439 |
|  |  | 1 2 | | CN601 | TRICYCLIC ANTIDEPRESSANTS |  | | 3,094 7,372 | 3.5% 1.4% | | <0.001 | 0.132 |
|  |  | 1 2 | | CV100 | BETA BLOCKERS/RELATED |  | | 10,059 27,905 | 11.3% 5.4% | | <0.001 | 0.212 |
|  |  | 1 2 | | CV200 | CALCIUM CHANNEL BLOCKERS |  | | 4,964 15,723 | 5.6% 3.1% | | <0.001 | 0.123 |
|  |  | 1 2 | | CV800 | ACE INHIBITORS |  | | 4,583 15,249 | 5.1% 3.0% | | <0.001 | 0.110 |
|  |  | 1 2 | | CV150 | ALPHA BLOCKERS/RELATED |  | | 3,651 8,098 | 4.1% 1.6% | | <0.001 | 0.152 |
|  |  | 1 2 | | CV490 | ANTIHYPERTENSIVES,OTHER |  | | 12,757 31,992 | 14.3% 6.2% | | <0.001 | 0.268 |
|  |  | 1 2 | | HS100 | ANDROGENS/ANABOLICS |  | | 557 2,411 | 0.6% 0.5% | | <0.001 | 0.021 |
|  |  | 1 2 | | HS800 | PROGESTINS |  | | 260 802 | 0.3% 0.2% | | <0.001 | 0.029 |
|  |  | 1 2 | | HS300 | ESTROGENS |  | | 163 740 | 0.2% 0.1% | | 0.006 | 0.010 |
|  |  | 1 2 | | 9997 | spironolactone |  | | 626 3,449 | 0.7% 0.7% | | 0.312 | 0.004 |
|  |  | 1 2 | | 3014 | cyproterone |  | | 10 10 | 0.0% 0.0% | | <0.001 | 0.011 |
|  |  | 1 2 | | 114477 | levetiracetam |  | | 21,265 63,597 | 23.8% 12.4% | | <0.001 | 0.300 |
|  |  | 1 2 | | 28439 | lamotrigine |  | | 8,041 22,765 | 9.0% 4.4% | | <0.001 | 0.183 |
|  |  | 1 2 | | 25480 | gabapentin |  | | 8,486 23,247 | 9.5% 4.5% | | <0.001 | 0.196 |
|  |  | 1 2 | | 38404 | topiramate |  | | 5,663 8,048 | 6.3% 1.6% | | <0.001 | 0.247 |
|  |  | 1 2 | | 32624 | oxcarbazepine |  | | 5,983 12,294 | 6.7% 2.4% | | <0.001 | 0.208 |
|  |  | 1 2 | | 623400 | lacosamide |  | | 4,315 5,144 | 4.8% 1.0% | | <0.001 | 0.229 |
|  |  | 1 2 | | 8183 | phenytoin |  | | 3,712 8,530 | 4.2% 1.7% | | <0.001 | 0.149 |
|  |  | 1 2 | | 21241 | clobazam |  | | 3,494 2,573 | 3.9% 0.5% | | <0.001 | 0.234 |
|  |  | 1 2 | | 2002 | carbamazepine |  | | 3,407 7,921 | 3.8% 1.5% | | <0.001 | 0.141 |
|  |  | 1 2 | | 39998 | zonisamide |  | | 3,105 3,548 | 3.5% 0.7% | | <0.001 | 0.196 |
|  |  | 1 2 | | 72236 | fosphenytoin |  | | 2,335 3,790 | 2.6% 0.7% | | <0.001 | 0.147 |
|  |  | 1 2 | | 4135 | ethosuximide |  | | 1,378 1,308 | 1.5% 0.3% | | <0.001 | 0.137 |
|  |  | 1 2 | | 69036 | rufinamide |  | | 632 365 | 0.7% 0.1% | | <0.001 | 0.102 |
|  |  | 1 2 | | 1739745 | brivaracetam |  | | 498 511 | 0.6% 0.1% | | <0.001 | 0.080 |
|  |  | 1 2 | | 14851 | vigabatrin |  | | 610 306 | 0.7% 0.1% | | <0.001 | 0.103 |
|  |  | 1 2 | | 24812 | felbamate |  | | 362 279 | 0.4% 0.1% | | <0.001 | 0.073 |
|  |  | 1 2 | | 1356552 | perampanel |  | | 425 378 | 0.5% 0.1% | | <0.001 | 0.077 |
|  |  | 1 2 | | 2045371 | cannabidiol |  | | 424 323 | 0.5% 0.1% | | <0.001 | 0.080 |
|  |  | 1 2 | | 1482502 | eslicarbazepine |  | | 208 370 | 0.2% 0.1% | | <0.001 | 0.041 |
|  |  | 1 2 | | 8691 | primidone |  | | 164 307 | 0.2% 0.1% | | <0.001 | 0.036 |
|  |  | 1 2 | | 2265690 | cenobamate |  | | 114 191 | 0.1% 0.0% | | <0.001 | 0.032 |
|  |  | 1 2 | | 31914 | tiagabine |  | | 46 60 | 0.1% 0.0% | | <0.001 | 0.022 |
|  |  | 1 2 | | 4328 | fenfluramine |  | | 32 13 | 0.0% 0.0% | | <0.001 | 0.024 |
|  |  | 1 2 | | 47858 | methsuximide |  | | 10 10 | 0.0% 0.0% | | <0.001 | 0.011 |
|  |  | 1 2 | | 2054968 | stiripentol |  | | 13 10 | 0.0% 0.0% | | <0.001 | 0.014 |
|  |  | 1 2 | | CN302 | BENZODIAZEPINE DERIVATIVE SEDATIVES/HYPNOTICS |  | | 49,315 132,873 | 55.2% 25.8% | | <0.001 | 0.626 |
|  |  | 1 2 | | CN309 | SEDATIVES/HYPNOTICS,OTHER |  | | 10,631 25,848 | 11.9% 5.0% | | <0.001 | 0.249 |
|  |  | 1 2 | | CN301 | BARBITURIC ACID DERIVATIVE SEDATIVES/HYPNOTICS |  | | 3,586 8,900 | 4.0% 1.7% | | <0.001 | 0.137 |
|  |  | 1 2 | | CN101 | OPIOID ANALGESICS |  | | 27,528 108,086 | 30.8% 21.0% | | <0.001 | 0.225 |
|  |  | 1 2 | | HS051 | GLUCOCORTICOIDS |  | | 22,564 96,982 | 25.2% 18.8% | | <0.001 | 0.155 |
|  | **Laboratory** | | | | | | | | | | | |
|  |  | Cohort | | |  | Mean ± SD | | Patients | % of Cohort | | P-Value | SMD |
|  |  | 1 2 | | 9083 | BMI | 25.7 +/- 7.1 25.4 +/- 7.5 | | 47,433 205,443 | 53.1% 39.9% | | <0.001 | 0.043 |
|  |  | 1 2 | |  | 0 - 0 kg/m2 |  | | 47,509 205,913 | 53.1% 40.0% | | <0.001 | 0.265 |
| **Cohort 1 (N = 78,971) and cohort 2 (N = 78,971) characteristics after propensity score matching** | | | | | | | | | | | | |
|  | **Demographics** | | | | | | | | | | | |
|  |  | Cohort | | |  | Mean ± SD | | Patients | % of Cohort | | P-Value | SMD |
|  |  | 1 2 | | AI | Age at Index | 25.2 +/- 12.8 26.5 +/- 13.4 | | 78,971 78,971 | 100% 100% | | <0.001 | 0.097 |
|  |  | 1 2 | | 2106-3 | White |  | | 45,987 46,632 | 58.2% 59.0% | | 0.001 | 0.017 |
|  |  | 1 2 | | 1002-5 | American Indian or Alaska Native |  | | 296 316 | 0.4% 0.4% | | 0.418 | 0.004 |
|  |  | 1 2 | | UNK | Unknown Race |  | | 10,597 10,036 | 13.4% 12.7% | | <0.001 | 0.021 |
|  |  | 1 2 | | 2076-8 | Native Hawaiian or Other Pacific Islander |  | | 317 329 | 0.4% 0.4% | | 0.636 | 0.002 |
|  |  | 1 2 | | 2054-5 | Black or African American |  | | 14,354 14,288 | 18.2% 18.1% | | 0.666 | 0.002 |
|  |  | 1 2 | | 2131-1 | Other Race |  | | 4,248 4,291 | 5.4% 5.4% | | 0.632 | 0.002 |
|  |  | 1 2 | | 2028-9 | Asian |  | | 3,172 3,079 | 4.0% 3.9% | | 0.230 | 0.006 |
|  | **Diagnosis** | | | | | | | | | | | |
|  |  | Cohort | | |  | Mean ± SD | | Patients | % of Cohort | | P-Value | SMD |
|  |  | 1 2 | | F80-F89 | Pervasive and specific developmental disorders |  | | 10,436 10,217 | 13.2% 12.9% | | 0.102 | 0.008 |
|  |  | 1 2 | | F40-F48 | Anxiety, dissociative, stress-related, somatoform and other nonpsychotic mental disorders |  | | 21,196 22,611 | 26.8% 28.6% | | <0.001 | 0.040 |
|  |  | 1 2 | | F90-F98 | Behavioral and emotional disorders with onset usually occurring in childhood and adolescence |  | | 12,590 12,965 | 15.9% 16.4% | | 0.010 | 0.013 |
|  |  | 1 2 | | F20-F29 | Schizophrenia, schizotypal, delusional, and other non-mood psychotic disorders |  | | 11,606 11,422 | 14.7% 14.5% | | 0.190 | 0.007 |
|  |  | 1 2 | | F60-F69 | Disorders of adult personality and behavior |  | | 5,449 5,436 | 6.9% 6.9% | | 0.897 | 0.001 |
|  |  | 1 2 | | F70-F79 | Intellectual Disabilities |  | | 4,216 4,048 | 5.3% 5.1% | | 0.058 | 0.010 |
|  |  | 1 2 | | F01-F09 | Mental disorders due to known physiological conditions |  | | 3,234 3,283 | 4.1% 4.2% | | 0.535 | 0.003 |
|  |  | 1 2 | | F99-F99 | Unspecified mental disorder (F99) |  | | 1,912 1,931 | 2.4% 2.4% | | 0.756 | 0.002 |
|  |  | 1 2 | | E08-E13 | Diabetes mellitus |  | | 3,887 4,209 | 4.9% 5.3% | | <0.001 | 0.018 |
|  |  | 1 2 | | Q00-Q07 | Congenital malformations of the nervous system |  | | 2,135 2,065 | 2.7% 2.6% | | 0.274 | 0.006 |
|  |  | 1 2 | | Q60-Q64 | Congenital malformations of the urinary system |  | | 438 450 | 0.6% 0.6% | | 0.686 | 0.002 |
|  |  | 1 2 | | N45 | Orchitis and epididymitis |  | | 420 460 | 0.5% 0.6% | | 0.176 | 0.007 |
|  |  | 1 2 | | N43 | Hydrocele and spermatocele |  | | 458 502 | 0.6% 0.6% | | 0.154 | 0.007 |
|  |  | 1 2 | | N50.1 | Vascular disorders of male genital organs |  | | 33 28 | 0.0% 0.0% | | 0.522 | 0.003 |
|  |  | 1 2 | | N49 | Inflammatory disorders of male genital organs, not elsewhere classified |  | | 122 134 | 0.2% 0.2% | | 0.453 | 0.004 |
|  |  | 1 2 | | I86.1 | Scrotal varices |  | | 208 229 | 0.3% 0.3% | | 0.314 | 0.005 |
|  |  | 1 2 | | Q53 | Undescended and ectopic testicle |  | | 416 395 | 0.5% 0.5% | | 0.460 | 0.004 |
|  |  | 1 2 | | Q55 | Other congenital malformations of male genital organs |  | | 275 266 | 0.3% 0.3% | | 0.698 | 0.002 |
|  |  | 1 2 | | Q54 | Hypospadias |  | | 174 168 | 0.2% 0.2% | | 0.745 | 0.002 |
|  |  | 1 2 | | C60-C63 | Malignant neoplasms of male genital organs |  | | 90 99 | 0.1% 0.1% | | 0.512 | 0.003 |
|  |  | 1 2 | | N44.8 | Other noninflammatory disorders of the testis |  | | 194 232 | 0.2% 0.3% | | 0.065 | 0.009 |
|  |  | 1 2 | | N44.0 | Torsion of testis |  | | 54 49 | 0.1% 0.1% | | 0.622 | 0.002 |
|  |  | 1 2 | | N30-N39 | Other diseases of the urinary system |  | | 3,879 3,970 | 4.9% 5.0% | | 0.292 | 0.005 |
|  |  | 1 2 | | K40 | Inguinal hernia |  | | 749 812 | 0.9% 1.0% | | 0.109 | 0.008 |
|  |  | 1 2 | | E84 | Cystic fibrosis |  | | 60 59 | 0.1% 0.1% | | 0.927 | <0.001 |
|  |  | 1 2 | | S30-S39 | Injuries to the abdomen, lower back, lumbar spine, pelvis and external genitals |  | | 4,508 4,846 | 5.7% 6.1% | | <0.001 | 0.018 |
|  |  | 1 2 | | E23.0 | Hypopituitarism |  | | 305 329 | 0.4% 0.4% | | 0.340 | 0.005 |
|  |  | 1 2 | | R56 | Convulsions, not elsewhere classified |  | | 26,098 23,866 | 33.0% 30.2% | | <0.001 | 0.061 |
|  |  | 1 2 | | E34.5 | Androgen insensitivity syndrome |  | | 10 10 | 0.0% 0.0% | | 1 | <0.001 |
|  |  | 1 2 | | Q87.1 | Congenital malformation syndromes predominantly associated with short stature |  | | 74 69 | 0.1% 0.1% | | 0.676 | 0.002 |
|  |  | 1 2 | | F31 | Bipolar disorder |  | | 23,977 26,033 | 30.4% 33.0% | | <0.001 | 0.056 |
|  |  | 1 2 | | F32 | Depressive episode |  | | 13,098 14,163 | 16.6% 17.9% | | <0.001 | 0.036 |
|  |  | 1 2 | | F39 | Unspecified mood [affective] disorder |  | | 5,610 5,816 | 7.1% 7.4% | | 0.045 | 0.010 |
|  |  | 1 2 | | F33 | Major depressive disorder, recurrent |  | | 3,973 4,361 | 5.0% 5.5% | | <0.001 | 0.022 |
|  |  | 1 2 | | F34 | Persistent mood [affective] disorders |  | | 1,791 1,924 | 2.3% 2.4% | | 0.027 | 0.011 |
|  |  | 1 2 | | F30 | Manic episode |  | | 1,728 1,627 | 2.2% 2.1% | | 0.078 | 0.009 |
|  |  | 1 2 | | F50-F59 | Behavioral syndromes associated with physiological disturbances and physical factors |  | | 2,272 2,369 | 2.9% 3.0% | | 0.148 | 0.007 |
|  |  | 1 2 | | J00-J99 | Diseases of the respiratory system |  | | 24,656 25,021 | 31.2% 31.7% | | 0.048 | 0.010 |
|  |  | 1 2 | | I00-I99 | Diseases of the circulatory system |  | | 18,529 19,686 | 23.5% 24.9% | | <0.001 | 0.034 |
|  |  | 1 2 | | K00-K95 | Diseases of the digestive system |  | | 21,945 22,919 | 27.8% 29.0% | | <0.001 | 0.027 |
|  |  | 1 2 | | Q65-Q79 | Congenital malformations and deformations of the musculoskeletal system |  | | 2,102 2,011 | 2.7% 2.5% | | 0.150 | 0.007 |
|  |  | 1 2 | | N17-N19 | Acute kidney failure and chronic kidney disease |  | | 3,616 3,925 | 4.6% 5.0% | | <0.001 | 0.018 |
|  |  | 1 2 | | A50-A64 | Infections with a predominantly sexual mode of transmission |  | | 906 1,033 | 1.1% 1.3% | | 0.004 | 0.015 |
|  |  | 1 2 | | E03 | Other hypothyroidism |  | | 2,140 2,174 | 2.7% 2.8% | | 0.600 | 0.003 |
|  |  | 1 2 | | E05 | Thyrotoxicosis [hyperthyroidism] |  | | 311 303 | 0.4% 0.4% | | 0.746 | 0.002 |
|  |  | 1 2 | | E83.1 | Disorders of iron metabolism |  | | 82 105 | 0.1% 0.1% | | 0.092 | 0.008 |
|  |  | 1 2 | | Q99 | Other chromosome abnormalities, not elsewhere classified |  | | 648 621 | 0.8% 0.8% | | 0.447 | 0.004 |
|  |  | 1 2 | | Q93 | Monosomies and deletions from the autosomes, not elsewhere classified |  | | 323 338 | 0.4% 0.4% | | 0.559 | 0.003 |
|  |  | 1 2 | | Q90 | Down syndrome |  | | 277 274 | 0.4% 0.3% | | 0.898 | 0.001 |
|  |  | 1 2 | | Q92 | Other trisomies and partial trisomies of the autosomes, not elsewhere classified |  | | 120 118 | 0.2% 0.1% | | 0.897 | 0.001 |
|  |  | 1 2 | | Q98.4 | Klinefelter syndrome, unspecified |  | | 64 73 | 0.1% 0.1% | | 0.442 | 0.004 |
|  |  | 1 2 | | Q98.5 | Karyotype 47, XYY |  | | 20 25 | 0.0% 0.0% | | 0.456 | 0.004 |
|  |  | 1 2 | | Q98.7 | Male with sex chromosome mosaicism |  | | 20 24 | 0.0% 0.0% | | 0.546 | 0.003 |
|  |  | 1 2 | | Q98.8 | Other specified sex chromosome abnormalities, male phenotype |  | | 20 25 | 0.0% 0.0% | | 0.456 | 0.004 |
|  |  | 1 2 | | Q98.0 | Klinefelter syndrome karyotype 47, XXY |  | | 12 16 | 0.0% 0.0% | | 0.450 | 0.004 |
|  |  | 1 2 | | Q98.1 | Klinefelter syndrome, male with more than two X chromosomes |  | | 10 10 | 0.0% 0.0% | | 1 | <0.001 |
|  |  | 1 2 | | Q98.6 | Male with structurally abnormal sex chromosome |  | | 10 0 | 0.0% 0% | | 0.002 | 0.016 |
|  |  | 1 2 | | Q98.9 | Sex chromosome abnormality, male phenotype, unspecified |  | | 10 10 | 0.0% 0.0% | | 1 | <0.001 |
|  |  | 1 2 | | D35.2 | Benign neoplasm of pituitary gland |  | | 76 86 | 0.1% 0.1% | | 0.432 | 0.004 |
|  |  | 1 2 | | D35.3 | Benign neoplasm of craniopharyngeal duct |  | | 32 39 | 0.0% 0.0% | | 0.406 | 0.004 |
|  |  | 1 2 | | E40-E46 | Malnutrition |  | | 1,717 1,782 | 2.2% 2.3% | | 0.266 | 0.006 |
|  |  | 1 2 | | G40.9 | Epilepsy, unspecified |  | | 23,946 22,369 | 30.3% 28.3% | | <0.001 | 0.044 |
|  |  | 1 2 | | G40.3 | Generalized idiopathic epilepsy and epileptic syndromes |  | | 6,685 6,367 | 8.5% 8.1% | | 0.004 | 0.015 |
|  |  | 1 2 | | G40.4 | Other generalized epilepsy and epileptic syndromes |  | | 5,131 4,825 | 6.5% 6.1% | | 0.002 | 0.016 |
|  |  | 1 2 | | G40.2 | Localization-related (focal) (partial) symptomatic epilepsy and epileptic syndromes with complex partial seizures |  | | 5,107 4,351 | 6.5% 5.5% | | <0.001 | 0.040 |
|  |  | 1 2 | | G40.8 | Other epilepsy and recurrent seizures |  | | 3,970 3,797 | 5.0% 4.8% | | 0.044 | 0.010 |
|  |  | 1 2 | | G40.1 | Localization-related (focal) (partial) symptomatic epilepsy and epileptic syndromes with simple partial seizures |  | | 3,651 3,278 | 4.6% 4.2% | | <0.001 | 0.023 |
|  |  | 1 2 | | G40.A | Absence epileptic syndrome |  | | 1,942 1,863 | 2.5% 2.4% | | 0.195 | 0.007 |
|  |  | 1 2 | | G40.0 | Localization-related (focal) (partial) idiopathic epilepsy and epileptic syndromes with seizures of localized onset |  | | 1,184 992 | 1.5% 1.3% | | <0.001 | 0.021 |
|  |  | 1 2 | | G40.5 | Epileptic seizures related to external causes |  | | 670 692 | 0.8% 0.9% | | 0.549 | 0.003 |
|  |  | 1 2 | | G40.B | Juvenile myoclonic epilepsy [impulsive petit mal] |  | | 561 501 | 0.7% 0.6% | | 0.065 | 0.009 |
|  |  | 1 2 | | F17 | Nicotine dependence |  | | 15,015 15,955 | 19.0% 20.2% | | <0.001 | 0.030 |
|  |  | 1 2 | | F12 | Cannabis related disorders |  | | 9,569 9,889 | 12.1% 12.5% | | 0.014 | 0.012 |
|  |  | 1 2 | | F10 | Alcohol related disorders |  | | 8,177 8,759 | 10.4% 11.1% | | <0.001 | 0.024 |
|  |  | 1 2 | | F19 | Other psychoactive substance related disorders |  | | 6,693 7,045 | 8.5% 8.9% | | 0.002 | 0.016 |
|  |  | 1 2 | | F11 | Opioid related disorders |  | | 3,944 4,307 | 5.0% 5.5% | | <0.001 | 0.021 |
|  |  | 1 2 | | F14 | Cocaine related disorders |  | | 3,341 3,501 | 4.2% 4.4% | | 0.048 | 0.010 |
|  |  | 1 2 | | F15 | Other stimulant related disorders |  | | 3,310 3,462 | 4.2% 4.4% | | 0.059 | 0.010 |
|  |  | 1 2 | | F13 | Sedative, hypnotic, or anxiolytic related disorders |  | | 1,672 1,738 | 2.1% 2.2% | | 0.253 | 0.006 |
|  |  | 1 2 | | F18 | Inhalant related disorders |  | | 1,340 1,425 | 1.7% 1.8% | | 0.103 | 0.008 |
|  |  | 1 2 | | F16 | Hallucinogen related disorders |  | | 739 773 | 0.9% 1.0% | | 0.380 | 0.004 |
|  | **Procedure** | | | | | | | | | | | |
|  |  | Cohort | | |  | Mean ± SD | | Patients | % of Cohort | | P-Value | SMD |
|  |  | 1 2 | | 1010843 | Radiation Oncology Treatment |  | | 151 186 | 0.2% 0.2% | | 0.056 | 0.010 |
|  |  | 1 2 | | 1008061 | Surgical Procedures on the Urinary System |  | | 2,048 2,121 | 2.6% 2.7% | | 0.252 | 0.006 |
|  |  | 1 2 | | 1008011 | Repair initial inguinal hernia, age 5 years or older |  | | 103 110 | 0.1% 0.1% | | 0.631 | 0.002 |
|  |  | 1 2 | | 1008470 | Surgical Procedures on the Male Genital System |  | | 712 722 | 0.9% 0.9% | | 0.791 | 0.001 |
|  | **Medication** | | | | | | | | | | | |
|  |  | Cohort | | |  | Mean ± SD | | Patients | % of Cohort | | P-Value | SMD |
|  |  | 1 2 | | 25025 | finasteride |  | | 185 211 | 0.2% 0.3% | | 0.191 | 0.007 |
|  |  | 1 2 | | CN750 | LITHIUM SALTS |  | | 3,317 3,366 | 4.2% 4.3% | | 0.540 | 0.003 |
|  |  | 1 2 | | 6135 | ketoconazole |  | | 1,102 1,175 | 1.4% 1.5% | | 0.123 | 0.008 |
|  |  | 1 2 | | 10829 | trimethoprim |  | | 4,408 4,702 | 5.6% 6.0% | | 0.002 | 0.016 |
|  |  | 1 2 | | 7454 | nitrofurantoin |  | | 352 366 | 0.4% 0.5% | | 0.601 | 0.003 |
|  |  | 1 2 | | AM200 | ERYTHROMYCINS/MACROLIDES |  | | 6,806 7,160 | 8.6% 9.1% | | 0.002 | 0.016 |
|  |  | 1 2 | | AM300 | AMINOGLYCOSIDES |  | | 3,468 3,659 | 4.4% 4.6% | | 0.021 | 0.012 |
|  |  | 1 2 | | AN000 | ANTINEOPLASTICS |  | | 1,254 1,394 | 1.6% 1.8% | | 0.006 | 0.014 |
|  |  | 1 2 | | CN709 | ANTIPSYCHOTICS,OTHER |  | | 30,165 31,298 | 38.2% 39.6% | | <0.001 | 0.029 |
|  |  | 1 2 | | CN701 | PHENOTHIAZINE/RELATED ANTIPSYCHOTICS |  | | 1,798 1,613 | 2.3% 2.0% | | 0.001 | 0.016 |
|  |  | 1 2 | | CN609 | ANTIDEPRESSANTS,OTHER |  | | 22,687 24,543 | 28.7% 31.1% | | <0.001 | 0.051 |
|  |  | 1 2 | | CN601 | TRICYCLIC ANTIDEPRESSANTS |  | | 2,522 2,756 | 3.2% 3.5% | | 0.001 | 0.016 |
|  |  | 1 2 | | CV100 | BETA BLOCKERS/RELATED |  | | 8,287 8,848 | 10.5% 11.2% | | <0.001 | 0.023 |
|  |  | 1 2 | | CV200 | CALCIUM CHANNEL BLOCKERS |  | | 4,173 4,502 | 5.3% 5.7% | | <0.001 | 0.018 |
|  |  | 1 2 | | CV800 | ACE INHIBITORS |  | | 3,852 4,227 | 4.9% 5.4% | | <0.001 | 0.022 |
|  |  | 1 2 | | CV150 | ALPHA BLOCKERS/RELATED |  | | 2,961 3,176 | 3.7% 4.0% | | 0.005 | 0.014 |
|  |  | 1 2 | | CV490 | ANTIHYPERTENSIVES,OTHER |  | | 10,176 10,849 | 12.9% 13.7% | | <0.001 | 0.025 |
|  |  | 1 2 | | HS100 | ANDROGENS/ANABOLICS |  | | 479 536 | 0.6% 0.7% | | 0.073 | 0.009 |
|  |  | 1 2 | | HS800 | PROGESTINS |  | | 210 225 | 0.3% 0.3% | | 0.471 | 0.004 |
|  |  | 1 2 | | HS300 | ESTROGENS |  | | 139 162 | 0.2% 0.2% | | 0.185 | 0.007 |
|  |  | 1 2 | | 9997 | spironolactone |  | | 552 601 | 0.7% 0.8% | | 0.148 | 0.007 |
|  |  | 1 2 | | 3014 | cyproterone |  | | 10 10 | 0.0% 0.0% | | 1 | <0.001 |
|  |  | 1 2 | | 114477 | levetiracetam |  | | 16,187 16,427 | 20.5% 20.8% | | 0.136 | 0.008 |
|  |  | 1 2 | | 28439 | lamotrigine |  | | 6,234 6,696 | 7.9% 8.5% | | <0.001 | 0.021 |
|  |  | 1 2 | | 25480 | gabapentin |  | | 7,174 7,769 | 9.1% 9.8% | | <0.001 | 0.026 |
|  |  | 1 2 | | 38404 | topiramate |  | | 3,670 3,810 | 4.6% 4.8% | | 0.097 | 0.008 |
|  |  | 1 2 | | 32624 | oxcarbazepine |  | | 4,212 4,239 | 5.3% 5.4% | | 0.763 | 0.002 |
|  |  | 1 2 | | 623400 | lacosamide |  | | 2,563 2,541 | 3.2% 3.2% | | 0.754 | 0.002 |
|  |  | 1 2 | | 8183 | phenytoin |  | | 2,671 2,805 | 3.4% 3.6% | | 0.065 | 0.009 |
|  |  | 1 2 | | 21241 | clobazam |  | | 1,783 1,750 | 2.3% 2.2% | | 0.574 | 0.003 |
|  |  | 1 2 | | 2002 | carbamazepine |  | | 2,531 2,582 | 3.2% 3.3% | | 0.468 | 0.004 |
|  |  | 1 2 | | 39998 | zonisamide |  | | 1,758 1,744 | 2.2% 2.2% | | 0.811 | 0.001 |
|  |  | 1 2 | | 72236 | fosphenytoin |  | | 1,323 1,250 | 1.7% 1.6% | | 0.147 | 0.007 |
|  |  | 1 2 | | 4135 | ethosuximide |  | | 868 800 | 1.1% 1.0% | | 0.094 | 0.008 |
|  |  | 1 2 | | 69036 | rufinamide |  | | 296 277 | 0.4% 0.4% | | 0.427 | 0.004 |
|  |  | 1 2 | | 1739745 | brivaracetam |  | | 262 248 | 0.3% 0.3% | | 0.535 | 0.003 |
|  |  | 1 2 | | 14851 | vigabatrin |  | | 256 242 | 0.3% 0.3% | | 0.530 | 0.003 |
|  |  | 1 2 | | 24812 | felbamate |  | | 201 191 | 0.3% 0.2% | | 0.613 | 0.003 |
|  |  | 1 2 | | 1356552 | perampanel |  | | 212 224 | 0.3% 0.3% | | 0.565 | 0.003 |
|  |  | 1 2 | | 2045371 | cannabidiol |  | | 181 190 | 0.2% 0.2% | | 0.640 | 0.002 |
|  |  | 1 2 | | 1482502 | eslicarbazepine |  | | 125 138 | 0.2% 0.2% | | 0.422 | 0.004 |
|  |  | 1 2 | | 8691 | primidone |  | | 122 129 | 0.2% 0.2% | | 0.658 | 0.002 |
|  |  | 1 2 | | 2265690 | cenobamate |  | | 60 64 | 0.1% 0.1% | | 0.719 | 0.002 |
|  |  | 1 2 | | 31914 | tiagabine |  | | 34 27 | 0.0% 0.0% | | 0.370 | 0.005 |
|  |  | 1 2 | | 4328 | fenfluramine |  | | 10 10 | 0.0% 0.0% | | 1 | <0.001 |
|  |  | 1 2 | | 47858 | methsuximide |  | | 10 10 | 0.0% 0.0% | | 1 | <0.001 |
|  |  | 1 2 | | 2054968 | stiripentol |  | | 10 10 | 0.0% 0.0% | | 1 | <0.001 |
|  |  | 1 2 | | CN302 | BENZODIAZEPINE DERIVATIVE SEDATIVES/HYPNOTICS |  | | 40,245 41,529 | 51.0% 52.6% | | <0.001 | 0.033 |
|  |  | 1 2 | | CN309 | SEDATIVES/HYPNOTICS,OTHER |  | | 8,289 8,608 | 10.5% 10.9% | | 0.009 | 0.013 |
|  |  | 1 2 | | CN301 | BARBITURIC ACID DERIVATIVE SEDATIVES/HYPNOTICS |  | | 2,422 2,444 | 3.1% 3.1% | | 0.749 | 0.002 |
|  |  | 1 2 | | CN101 | OPIOID ANALGESICS |  | | 23,042 25,058 | 29.2% 31.7% | | <0.001 | 0.055 |
|  |  | 1 2 | | HS051 | GLUCOCORTICOIDS |  | | 18,548 19,579 | 23.5% 24.8% | | <0.001 | 0.031 |
|  | **Laboratory** | | | | | | | | | | | |
|  |  | Cohort | | |  | Mean ± SD | | Patients | % of Cohort | | P-Value | SMD |
|  |  | 1 2 | | 9083 | BMI | 25.9 +/- 7.0 26.2 +/- 7.3 | | 40,479 43,143 | 51.3% 54.6% | | <0.001 | 0.045 |
|  |  | 1 2 | |  | 0 - 0 kg/m2 |  | | 40,550 43,230 | 51.3% 54.7% | | <0.001 | 0.068 |

**Abbreviations:** BMI = Body Mass Index; SD = Standard deviation; SMD = Standardised mean difference

**N.B.:** Uncorrected p-values were calculated using two-sided t-tests for continuous covariates and two-sided Z-tests for categorical covariates. These p-values reflect descriptive comparisons of baseline characteristics during propensity score matching. Correction for multiple comparisons is not appropriate in this context, as the aim is to assess covariate balance (reflected by a standardised mean difference of <0.1) rather than to test hypotheses. Exact p-values beyond three decimal places are not provided in the propensity score matching outputs generated by the TriNetX platform. Where p-values fall below this threshold, they are reported as <0.001.

# Supplementary Table 3: Propensity score matching men with epilepsy or bipolar disorder exposed (cohort 1) and unexposed (cohort 2) to valproate – 30-day outcome assessment

Propensity score matching was performed on all listed characteristics. Characteristics of the cohorts before and after matching are summarized in the table below.

| **Cohort 1 and cohort 2 patient count before and after propensity score matching** | | | | | | | | | | | | |
| --- | --- | --- | --- | --- | --- | --- | --- | --- | --- | --- | --- | --- |
|  | | | Cohort | | | Patient count before matching | | | | Patient count after matching | | |
|  | | | 1 - Men with epilepsy or bipolar disorder exposed to valproate_v9 | | | 90,571 | | | | 77,826 | | |
|  | | | 2 - Men with epilepsy or bipolar disorder not exposed to valproate_v9 | | | 524,095 | | | | 77,826 | | |
| **Propensity score density function - Before and after matching (cohort 1 - purple, cohort 2 - green)** | | | | | | | | | | | | |
|  |  | | 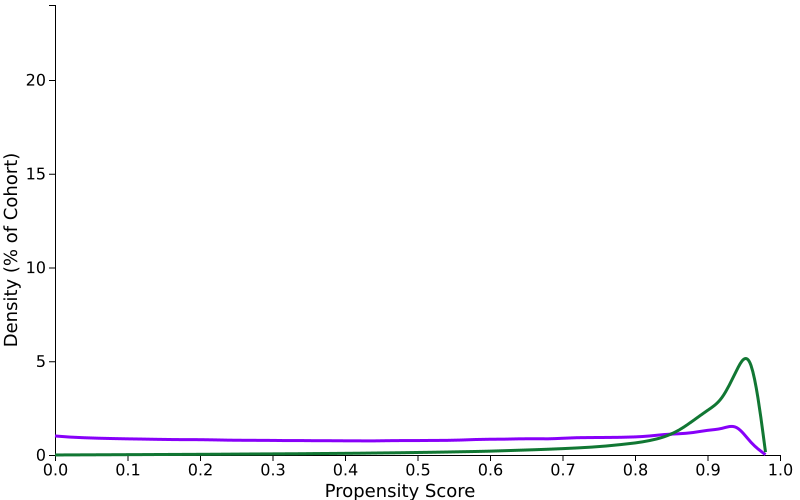 | | | | 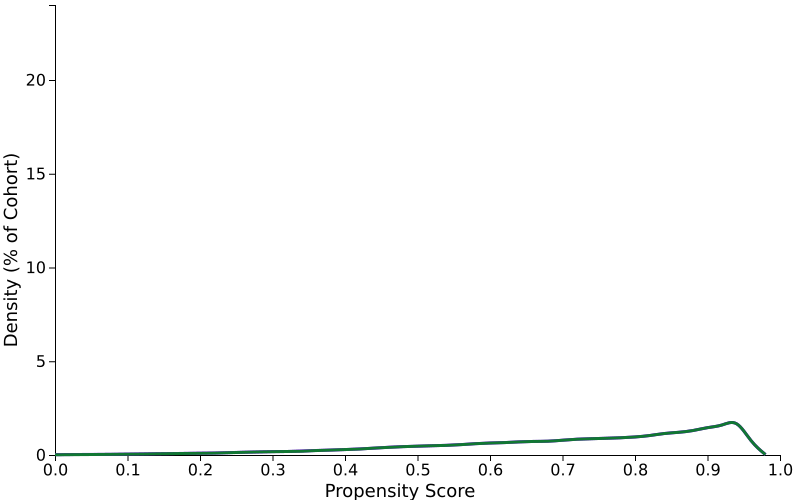 | | | | | |
| **Cohort 1 (N = 90,571) and cohort 2 (N = 524,095) characteristics before propensity score matching** | | | | | | | | | | | | |
|  | **Demographics** | | | | | | | | | | | |
|  |  | Cohort | | |  | Mean ± SD | | Patients | % of Cohort | | P-Value | SMD |
|  |  | 1 2 | | AI | Age at Index | 25.3 +/- 12.8 21.5 +/- 14.9 | | 88,063 503,020 | 100% 100% | | <0.001 | 0.268 |
|  |  | 1 2 | | 2106-3 | White |  | | 51,251 287,709 | 58.2% 57.2% | | <0.001 | 0.020 |
|  |  | 1 2 | | 1002-5 | American Indian or Alaska Native |  | | 349 1,743 | 0.4% 0.3% | | 0.022 | 0.008 |
|  |  | 1 2 | | UNK | Unknown Race |  | | 11,454 78,346 | 13.0% 15.6% | | <0.001 | 0.073 |
|  |  | 1 2 | | 2076-8 | Native Hawaiian or Other Pacific Islander |  | | 349 2,348 | 0.4% 0.5% | | 0.004 | 0.011 |
|  |  | 1 2 | | 2054-5 | Black or African American |  | | 16,344 87,044 | 18.6% 17.3% | | <0.001 | 0.033 |
|  |  | 1 2 | | 2131-1 | Other Race |  | | 4,670 29,664 | 5.3% 5.9% | | <0.001 | 0.026 |
|  |  | 1 2 | | 2028-9 | Asian |  | | 3,646 16,166 | 4.1% 3.2% | | <0.001 | 0.049 |
|  | **Diagnosis** | | | | | | | | | | | |
|  |  | Cohort | | |  | Mean ± SD | | Patients | % of Cohort | | P-Value | SMD |
|  |  | 1 2 | | F80-F89 | Pervasive and specific developmental disorders |  | | 13,953 45,580 | 15.8% 9.1% | | <0.001 | 0.207 |
|  |  | 1 2 | | F40-F48 | Anxiety, dissociative, stress-related, somatoform and other nonpsychotic mental disorders |  | | 24,938 89,250 | 28.3% 17.7% | | <0.001 | 0.253 |
|  |  | 1 2 | | F90-F98 | Behavioral and emotional disorders with onset usually occurring in childhood and adolescence |  | | 15,653 50,733 | 17.8% 10.1% | | <0.001 | 0.223 |
|  |  | 1 2 | | F20-F29 | Schizophrenia, schizotypal, delusional, and other non-mood psychotic disorders |  | | 14,250 27,284 | 16.2% 5.4% | | <0.001 | 0.352 |
|  |  | 1 2 | | F60-F69 | Disorders of adult personality and behavior |  | | 7,406 13,710 | 8.4% 2.7% | | <0.001 | 0.250 |
|  |  | 1 2 | | F70-F79 | Intellectual Disabilities |  | | 6,552 9,616 | 7.4% 1.9% | | <0.001 | 0.264 |
|  |  | 1 2 | | F01-F09 | Mental disorders due to known physiological conditions |  | | 4,694 8,511 | 5.3% 1.7% | | <0.001 | 0.199 |
|  |  | 1 2 | | F99-F99 | Unspecified mental disorder (F99) |  | | 2,550 4,878 | 2.9% 1.0% | | <0.001 | 0.140 |
|  |  | 1 2 | | E08-E13 | Diabetes mellitus |  | | 4,511 18,887 | 5.1% 3.8% | | <0.001 | 0.066 |
|  |  | 1 2 | | Q00-Q07 | Congenital malformations of the nervous system |  | | 3,161 9,599 | 3.6% 1.9% | | <0.001 | 0.103 |
|  |  | 1 2 | | Q60-Q64 | Congenital malformations of the urinary system |  | | 543 2,873 | 0.6% 0.6% | | 0.101 | 0.006 |
|  |  | 1 2 | | N45 | Orchitis and epididymitis |  | | 492 1,965 | 0.6% 0.4% | | <0.001 | 0.024 |
|  |  | 1 2 | | N43 | Hydrocele and spermatocele |  | | 571 2,775 | 0.6% 0.6% | | <0.001 | 0.013 |
|  |  | 1 2 | | N50.1 | Vascular disorders of male genital organs |  | | 44 151 | 0.0% 0.0% | | 0.003 | 0.010 |
|  |  | 1 2 | | N49 | Inflammatory disorders of male genital organs, not elsewhere classified |  | | 145 583 | 0.2% 0.1% | | <0.001 | 0.013 |
|  |  | 1 2 | | I86.1 | Scrotal varices |  | | 240 1,104 | 0.3% 0.2% | | 0.002 | 0.011 |
|  |  | 1 2 | | Q53 | Undescended and ectopic testicle |  | | 563 2,745 | 0.6% 0.5% | | 0.001 | 0.012 |
|  |  | 1 2 | | Q55 | Other congenital malformations of male genital organs |  | | 382 2,466 | 0.4% 0.5% | | 0.026 | 0.008 |
|  |  | 1 2 | | Q54 | Hypospadias |  | | 214 1,417 | 0.2% 0.3% | | 0.043 | 0.008 |
|  |  | 1 2 | | C60-C63 | Malignant neoplasms of male genital organs |  | | 98 591 | 0.1% 0.1% | | 0.619 | 0.002 |
|  |  | 1 2 | | N44.8 | Other noninflammatory disorders of the testis |  | | 250 718 | 0.3% 0.1% | | <0.001 | 0.031 |
|  |  | 1 2 | | N44.0 | Torsion of testis |  | | 62 312 | 0.1% 0.1% | | 0.362 | 0.003 |
|  |  | 1 2 | | N30-N39 | Other diseases of the urinary system |  | | 4,927 16,721 | 5.6% 3.3% | | <0.001 | 0.110 |
|  |  | 1 2 | | K40 | Inguinal hernia |  | | 900 4,936 | 1.0% 1.0% | | 0.260 | 0.004 |
|  |  | 1 2 | | E84 | Cystic fibrosis |  | | 74 343 | 0.1% 0.1% | | 0.102 | 0.006 |
|  |  | 1 2 | | S30-S39 | Injuries to the abdomen, lower back, lumbar spine, pelvis and external genitals |  | | 5,400 19,839 | 6.1% 3.9% | | <0.001 | 0.100 |
|  |  | 1 2 | | E23.0 | Hypopituitarism |  | | 370 1,430 | 0.4% 0.3% | | <0.001 | 0.023 |
|  |  | 1 2 | | R56 | Convulsions, not elsewhere classified |  | | 32,023 242,661 | 36.4% 48.2% | | <0.001 | 0.242 |
|  |  | 1 2 | | E34.5 | Androgen insensitivity syndrome |  | | 10 14 | 0.0% 0.0% | | <0.001 | 0.010 |
|  |  | 1 2 | | Q87.1 | Congenital malformation syndromes predominantly associated with short stature |  | | 91 347 | 0.1% 0.1% | | 0.001 | 0.012 |
|  |  | 1 2 | | F31 | Bipolar disorder |  | | 26,039 126,843 | 29.6% 25.2% | | <0.001 | 0.098 |
|  |  | 1 2 | | F32 | Depressive episode |  | | 15,375 51,561 | 17.5% 10.3% | | <0.001 | 0.210 |
|  |  | 1 2 | | F39 | Unspecified mood [affective] disorder |  | | 7,153 13,346 | 8.1% 2.7% | | <0.001 | 0.244 |
|  |  | 1 2 | | F33 | Major depressive disorder, recurrent |  | | 4,589 14,705 | 5.2% 2.9% | | <0.001 | 0.116 |
|  |  | 1 2 | | F34 | Persistent mood [affective] disorders |  | | 2,212 5,435 | 2.5% 1.1% | | <0.001 | 0.108 |
|  |  | 1 2 | | F30 | Manic episode |  | | 2,343 2,887 | 2.7% 0.6% | | <0.001 | 0.166 |
|  |  | 1 2 | | F50-F59 | Behavioral syndromes associated with physiological disturbances and physical factors |  | | 2,844 8,624 | 3.2% 1.7% | | <0.001 | 0.098 |
|  |  | 1 2 | | J00-J99 | Diseases of the respiratory system |  | | 29,365 147,431 | 33.3% 29.3% | | <0.001 | 0.087 |
|  |  | 1 2 | | I00-I99 | Diseases of the circulatory system |  | | 22,199 94,295 | 25.2% 18.7% | | <0.001 | 0.157 |
|  |  | 1 2 | | K00-K95 | Diseases of the digestive system |  | | 26,366 120,355 | 29.9% 23.9% | | <0.001 | 0.136 |
|  |  | 1 2 | | Q65-Q79 | Congenital malformations and deformations of the musculoskeletal system |  | | 2,823 14,326 | 3.2% 2.8% | | <0.001 | 0.021 |
|  |  | 1 2 | | N17-N19 | Acute kidney failure and chronic kidney disease |  | | 4,383 17,746 | 5.0% 3.5% | | <0.001 | 0.072 |
|  |  | 1 2 | | A50-A64 | Infections with a predominantly sexual mode of transmission |  | | 1,045 4,461 | 1.2% 0.9% | | <0.001 | 0.030 |
|  |  | 1 2 | | E03 | Other hypothyroidism |  | | 2,706 8,081 | 3.1% 1.6% | | <0.001 | 0.097 |
|  |  | 1 2 | | E05 | Thyrotoxicosis [hyperthyroidism] |  | | 380 1,139 | 0.4% 0.2% | | <0.001 | 0.036 |
|  |  | 1 2 | | E83.1 | Disorders of iron metabolism |  | | 100 577 | 0.1% 0.1% | | 0.926 | <0.001 |
|  |  | 1 2 | | Q99 | Other chromosome abnormalities, not elsewhere classified |  | | 1,021 2,710 | 1.2% 0.5% | | <0.001 | 0.068 |
|  |  | 1 2 | | Q93 | Monosomies and deletions from the autosomes, not elsewhere classified |  | | 465 1,479 | 0.5% 0.3% | | <0.001 | 0.037 |
|  |  | 1 2 | | Q90 | Down syndrome |  | | 329 1,697 | 0.4% 0.3% | | 0.090 | 0.006 |
|  |  | 1 2 | | Q92 | Other trisomies and partial trisomies of the autosomes, not elsewhere classified |  | | 182 491 | 0.2% 0.1% | | <0.001 | 0.028 |
|  |  | 1 2 | | Q98.4 | Klinefelter syndrome, unspecified |  | | 81 226 | 0.1% 0.0% | | <0.001 | 0.018 |
|  |  | 1 2 | | Q98.5 | Karyotype 47, XYY |  | | 28 84 | 0.0% 0.0% | | 0.003 | 0.010 |
|  |  | 1 2 | | Q98.7 | Male with sex chromosome mosaicism |  | | 28 49 | 0.0% 0.0% | | <0.001 | 0.015 |
|  |  | 1 2 | | Q98.8 | Other specified sex chromosome abnormalities, male phenotype |  | | 27 58 | 0.0% 0.0% | | <0.001 | 0.013 |
|  |  | 1 2 | | Q98.0 | Klinefelter syndrome karyotype 47, XXY |  | | 16 50 | 0.0% 0.0% | | 0.033 | 0.007 |
|  |  | 1 2 | | Q98.1 | Klinefelter syndrome, male with more than two X chromosomes |  | | 10 14 | 0.0% 0.0% | | <0.001 | 0.010 |
|  |  | 1 2 | | Q98.6 | Male with structurally abnormal sex chromosome |  | | 10 10 | 0.0% 0.0% | | <0.001 | 0.011 |
|  |  | 1 2 | | Q98.9 | Sex chromosome abnormality, male phenotype, unspecified |  | | 10 10 | 0.0% 0.0% | | <0.001 | 0.011 |
|  |  | 1 2 | | D35.2 | Benign neoplasm of pituitary gland |  | | 103 398 | 0.1% 0.1% | | <0.001 | 0.012 |
|  |  | 1 2 | | D35.3 | Benign neoplasm of craniopharyngeal duct |  | | 41 157 | 0.0% 0.0% | | 0.022 | 0.008 |
|  |  | 1 2 | | E40-E46 | Malnutrition |  | | 2,240 7,539 | 2.5% 1.5% | | <0.001 | 0.074 |
|  |  | 1 2 | | G40.9 | Epilepsy, unspecified |  | | 30,815 98,292 | 35.0% 19.5% | | <0.001 | 0.352 |
|  |  | 1 2 | | G40.3 | Generalized idiopathic epilepsy and epileptic syndromes |  | | 10,530 14,819 | 12.0% 2.9% | | <0.001 | 0.348 |
|  |  | 1 2 | | G40.4 | Other generalized epilepsy and epileptic syndromes |  | | 8,627 12,673 | 9.8% 2.5% | | <0.001 | 0.306 |
|  |  | 1 2 | | G40.2 | Localization-related (focal) (partial) symptomatic epilepsy and epileptic syndromes with complex partial seizures |  | | 8,047 19,181 | 9.1% 3.8% | | <0.001 | 0.218 |
|  |  | 1 2 | | G40.8 | Other epilepsy and recurrent seizures |  | | 6,362 16,593 | 7.2% 3.3% | | <0.001 | 0.177 |
|  |  | 1 2 | | G40.1 | Localization-related (focal) (partial) symptomatic epilepsy and epileptic syndromes with simple partial seizures |  | | 6,117 14,240 | 6.9% 2.8% | | <0.001 | 0.192 |
|  |  | 1 2 | | G40.A | Absence epileptic syndrome |  | | 3,070 4,963 | 3.5% 1.0% | | <0.001 | 0.170 |
|  |  | 1 2 | | G40.0 | Localization-related (focal) (partial) idiopathic epilepsy and epileptic syndromes with seizures of localized onset |  | | 1,952 4,698 | 2.2% 0.9% | | <0.001 | 0.103 |
|  |  | 1 2 | | G40.5 | Epileptic seizures related to external causes |  | | 1,227 2,565 | 1.4% 0.5% | | <0.001 | 0.091 |
|  |  | 1 2 | | G40.B | Juvenile myoclonic epilepsy [impulsive petit mal] |  | | 870 743 | 1.0% 0.1% | | <0.001 | 0.112 |
|  |  | 1 2 | | F17 | Nicotine dependence |  | | 17,253 65,626 | 19.6% 13.0% | | <0.001 | 0.178 |
|  |  | 1 2 | | F12 | Cannabis related disorders |  | | 11,478 29,852 | 13.0% 5.9% | | <0.001 | 0.244 |
|  |  | 1 2 | | F10 | Alcohol related disorders |  | | 9,240 39,090 | 10.5% 7.8% | | <0.001 | 0.095 |
|  |  | 1 2 | | F19 | Other psychoactive substance related disorders |  | | 7,848 23,085 | 8.9% 4.6% | | <0.001 | 0.173 |
|  |  | 1 2 | | F11 | Opioid related disorders |  | | 4,527 16,733 | 5.1% 3.3% | | <0.001 | 0.090 |
|  |  | 1 2 | | F14 | Cocaine related disorders |  | | 3,952 10,901 | 4.5% 2.2% | | <0.001 | 0.130 |
|  |  | 1 2 | | F15 | Other stimulant related disorders |  | | 3,847 10,577 | 4.4% 2.1% | | <0.001 | 0.128 |
|  |  | 1 2 | | F13 | Sedative, hypnotic, or anxiolytic related disorders |  | | 2,014 5,496 | 2.3% 1.1% | | <0.001 | 0.093 |
|  |  | 1 2 | | F18 | Inhalant related disorders |  | | 1,647 4,463 | 1.9% 0.9% | | <0.001 | 0.084 |
|  |  | 1 2 | | F16 | Hallucinogen related disorders |  | | 897 1,995 | 1.0% 0.4% | | <0.001 | 0.074 |
|  | **Procedure** | | | | | | | | | | | |
|  |  | Cohort | | |  | Mean ± SD | | Patients | % of Cohort | | P-Value | SMD |
|  |  | 1 2 | | 1010843 | Radiation Oncology Treatment |  | | 188 805 | 0.2% 0.2% | | <0.001 | 0.012 |
|  |  | 1 2 | | 1008061 | Surgical Procedures on the Urinary System |  | | 2,646 8,988 | 3.0% 1.8% | | <0.001 | 0.080 |
|  |  | 1 2 | | 1008011 | Repair initial inguinal hernia, age 5 years or older |  | | 129 447 | 0.1% 0.1% | | <0.001 | 0.017 |
|  |  | 1 2 | | 1008470 | Surgical Procedures on the Male Genital System |  | | 884 5,019 | 1.0% 1.0% | | 0.868 | 0.001 |
|  | **Medication** | | | | | | | | | | | |
|  |  | Cohort | | |  | Mean ± SD | | Patients | % of Cohort | | P-Value | SMD |
|  |  | 1 2 | | 25025 | finasteride |  | | 205 876 | 0.2% 0.2% | | <0.001 | 0.013 |
|  |  | 1 2 | | CN750 | LITHIUM SALTS |  | | 4,039 7,612 | 4.6% 1.5% | | <0.001 | 0.179 |
|  |  | 1 2 | | 6135 | ketoconazole |  | | 1,350 4,636 | 1.5% 0.9% | | <0.001 | 0.056 |
|  |  | 1 2 | | 10829 | trimethoprim |  | | 5,328 20,589 | 6.1% 4.1% | | <0.001 | 0.089 |
|  |  | 1 2 | | 7454 | nitrofurantoin |  | | 446 1,225 | 0.5% 0.2% | | <0.001 | 0.043 |
|  |  | 1 2 | | AM200 | ERYTHROMYCINS/MACROLIDES |  | | 8,185 32,010 | 9.3% 6.4% | | <0.001 | 0.109 |
|  |  | 1 2 | | AM300 | AMINOGLYCOSIDES |  | | 4,382 14,308 | 5.0% 2.8% | | <0.001 | 0.110 |
|  |  | 1 2 | | AN000 | ANTINEOPLASTICS |  | | 1,492 6,409 | 1.7% 1.3% | | <0.001 | 0.035 |
|  |  | 1 2 | | CN709 | ANTIPSYCHOTICS,OTHER |  | | 35,618 61,459 | 40.4% 12.2% | | <0.001 | 0.677 |
|  |  | 1 2 | | CN701 | PHENOTHIAZINE/RELATED ANTIPSYCHOTICS |  | | 2,909 2,219 | 3.3% 0.4% | | <0.001 | 0.212 |
|  |  | 1 2 | | CN609 | ANTIDEPRESSANTS,OTHER |  | | 26,814 64,337 | 30.4% 12.8% | | <0.001 | 0.439 |
|  |  | 1 2 | | CN601 | TRICYCLIC ANTIDEPRESSANTS |  | | 3,071 7,292 | 3.5% 1.4% | | <0.001 | 0.132 |
|  |  | 1 2 | | CV100 | BETA BLOCKERS/RELATED |  | | 10,034 27,750 | 11.4% 5.5% | | <0.001 | 0.212 |
|  |  | 1 2 | | CV200 | CALCIUM CHANNEL BLOCKERS |  | | 4,952 15,631 | 5.6% 3.1% | | <0.001 | 0.123 |
|  |  | 1 2 | | CV800 | ACE INHIBITORS |  | | 4,579 15,165 | 5.2% 3.0% | | <0.001 | 0.110 |
|  |  | 1 2 | | CV150 | ALPHA BLOCKERS/RELATED |  | | 3,645 8,075 | 4.1% 1.6% | | <0.001 | 0.152 |
|  |  | 1 2 | | CV490 | ANTIHYPERTENSIVES,OTHER |  | | 12,414 30,949 | 14.1% 6.2% | | <0.001 | 0.266 |
|  |  | 1 2 | | HS100 | ANDROGENS/ANABOLICS |  | | 556 2,385 | 0.6% 0.5% | | <0.001 | 0.021 |
|  |  | 1 2 | | HS800 | PROGESTINS |  | | 258 796 | 0.3% 0.2% | | <0.001 | 0.028 |
|  |  | 1 2 | | HS300 | ESTROGENS |  | | 163 738 | 0.2% 0.1% | | 0.007 | 0.009 |
|  |  | 1 2 | | 9997 | spironolactone |  | | 623 3,419 | 0.7% 0.7% | | 0.357 | 0.003 |
|  |  | 1 2 | | 3014 | cyproterone |  | | 10 10 | 0.0% 0.0% | | <0.001 | 0.011 |
|  |  | 1 2 | | 114477 | levetiracetam |  | | 20,755 62,239 | 23.6% 12.4% | | <0.001 | 0.295 |
|  |  | 1 2 | | 28439 | lamotrigine |  | | 7,907 22,560 | 9.0% 4.5% | | <0.001 | 0.180 |
|  |  | 1 2 | | 25480 | gabapentin |  | | 8,441 23,111 | 9.6% 4.6% | | <0.001 | 0.195 |
|  |  | 1 2 | | 38404 | topiramate |  | | 5,480 7,801 | 6.2% 1.6% | | <0.001 | 0.244 |
|  |  | 1 2 | | 32624 | oxcarbazepine |  | | 5,744 11,554 | 6.5% 2.3% | | <0.001 | 0.207 |
|  |  | 1 2 | | 623400 | lacosamide |  | | 4,248 5,093 | 4.8% 1.0% | | <0.001 | 0.228 |
|  |  | 1 2 | | 8183 | phenytoin |  | | 3,690 8,493 | 4.2% 1.7% | | <0.001 | 0.149 |
|  |  | 1 2 | | 21241 | clobazam |  | | 3,353 2,526 | 3.8% 0.5% | | <0.001 | 0.229 |
|  |  | 1 2 | | 2002 | carbamazepine |  | | 3,368 7,780 | 3.8% 1.5% | | <0.001 | 0.141 |
|  |  | 1 2 | | 39998 | zonisamide |  | | 2,921 3,382 | 3.3% 0.7% | | <0.001 | 0.190 |
|  |  | 1 2 | | 72236 | fosphenytoin |  | | 2,250 3,539 | 2.6% 0.7% | | <0.001 | 0.147 |
|  |  | 1 2 | | 4135 | ethosuximide |  | | 1,261 1,192 | 1.4% 0.2% | | <0.001 | 0.132 |
|  |  | 1 2 | | 69036 | rufinamide |  | | 627 356 | 0.7% 0.1% | | <0.001 | 0.103 |
|  |  | 1 2 | | 1739745 | brivaracetam |  | | 486 509 | 0.6% 0.1% | | <0.001 | 0.079 |
|  |  | 1 2 | | 14851 | vigabatrin |  | | 585 288 | 0.7% 0.1% | | <0.001 | 0.101 |
|  |  | 1 2 | | 24812 | felbamate |  | | 338 251 | 0.4% 0.0% | | <0.001 | 0.072 |
|  |  | 1 2 | | 1356552 | perampanel |  | | 415 378 | 0.5% 0.1% | | <0.001 | 0.076 |
|  |  | 1 2 | | 2045371 | cannabidiol |  | | 407 319 | 0.5% 0.1% | | <0.001 | 0.078 |
|  |  | 1 2 | | 1482502 | eslicarbazepine |  | | 204 365 | 0.2% 0.1% | | <0.001 | 0.041 |
|  |  | 1 2 | | 8691 | primidone |  | | 164 303 | 0.2% 0.1% | | <0.001 | 0.036 |
|  |  | 1 2 | | 2265690 | cenobamate |  | | 111 191 | 0.1% 0.0% | | <0.001 | 0.031 |
|  |  | 1 2 | | 31914 | tiagabine |  | | 46 58 | 0.1% 0.0% | | <0.001 | 0.023 |
|  |  | 1 2 | | 4328 | fenfluramine |  | | 32 13 | 0.0% 0.0% | | <0.001 | 0.024 |
|  |  | 1 2 | | 47858 | methsuximide |  | | 10 10 | 0.0% 0.0% | | <0.001 | 0.011 |
|  |  | 1 2 | | 2054968 | stiripentol |  | | 13 10 | 0.0% 0.0% | | <0.001 | 0.014 |
|  |  | 1 2 | | CN302 | BENZODIAZEPINE DERIVATIVE SEDATIVES/HYPNOTICS |  | | 48,423 128,955 | 55.0% 25.6% | | <0.001 | 0.627 |
|  |  | 1 2 | | CN309 | SEDATIVES/HYPNOTICS,OTHER |  | | 10,518 25,284 | 11.9% 5.0% | | <0.001 | 0.250 |
|  |  | 1 2 | | CN301 | BARBITURIC ACID DERIVATIVE SEDATIVES/HYPNOTICS |  | | 3,350 7,968 | 3.8% 1.6% | | <0.001 | 0.137 |
|  |  | 1 2 | | CN101 | OPIOID ANALGESICS |  | | 27,069 105,259 | 30.7% 20.9% | | <0.001 | 0.226 |
|  |  | 1 2 | | HS051 | GLUCOCORTICOIDS |  | | 22,195 94,122 | 25.2% 18.7% | | <0.001 | 0.157 |
|  | **Laboratory** | | | | | | | | | | | |
|  |  | Cohort | | |  | Mean ± SD | | Patients | % of Cohort | | P-Value | SMD |
|  |  | 1 2 | | 9083 | BMI | 25.9 +/- 7.0 25.6 +/- 7.5 | | 46,315 200,558 | 52.6% 39.9% | | <0.001 | 0.042 |
|  |  | 1 2 | |  | 0 - 0 kg/m2 |  | | 46,391 201,023 | 52.7% 40.0% | | <0.001 | 0.257 |
| **Cohort 1 (N = 77,826) and cohort 2 (N = 77,826) characteristics after propensity score matching** | | | | | | | | | | | | |
|  | **Demographics** | | | | | | | | | | | |
|  |  | Cohort | | |  | Mean ± SD | | Patients | % of Cohort | | P-Value | SMD |
|  |  | 1 2 | | AI | Age at Index | 25.5 +/- 12.7 26.6 +/- 13.3 | | 77,826 77,826 | 100% 100% | | <0.001 | 0.090 |
|  |  | 1 2 | | 2106-3 | White |  | | 45,215 45,757 | 58.1% 58.8% | | 0.005 | 0.014 |
|  |  | 1 2 | | 1002-5 | American Indian or Alaska Native |  | | 290 313 | 0.4% 0.4% | | 0.348 | 0.005 |
|  |  | 1 2 | | UNK | Unknown Race |  | | 10,415 9,970 | 13.4% 12.8% | | 0.001 | 0.017 |
|  |  | 1 2 | | 2076-8 | Native Hawaiian or Other Pacific Islander |  | | 311 295 | 0.4% 0.4% | | 0.515 | 0.003 |
|  |  | 1 2 | | 2054-5 | Black or African American |  | | 14,290 14,181 | 18.4% 18.2% | | 0.475 | 0.004 |
|  |  | 1 2 | | 2131-1 | Other Race |  | | 4,162 4,291 | 5.3% 5.5% | | 0.149 | 0.007 |
|  |  | 1 2 | | 2028-9 | Asian |  | | 3,143 3,019 | 4.0% 3.9% | | 0.107 | 0.008 |
|  | **Diagnosis** | | | | | | | | | | | |
|  |  | Cohort | | |  | Mean ± SD | | Patients | % of Cohort | | P-Value | SMD |
|  |  | 1 2 | | F80-F89 | Pervasive and specific developmental disorders |  | | 10,198 9,974 | 13.1% 12.8% | | 0.091 | 0.009 |
|  |  | 1 2 | | F40-F48 | Anxiety, dissociative, stress-related, somatoform and other nonpsychotic mental disorders |  | | 21,065 22,380 | 27.1% 28.8% | | <0.001 | 0.038 |
|  |  | 1 2 | | F90-F98 | Behavioral and emotional disorders with onset usually occurring in childhood and adolescence |  | | 12,415 12,767 | 16.0% 16.4% | | 0.015 | 0.012 |
|  |  | 1 2 | | F20-F29 | Schizophrenia, schizotypal, delusional, and other non-mood psychotic disorders |  | | 11,547 11,327 | 14.8% 14.6% | | 0.115 | 0.008 |
|  |  | 1 2 | | F60-F69 | Disorders of adult personality and behavior |  | | 5,400 5,338 | 6.9% 6.9% | | 0.535 | 0.003 |
|  |  | 1 2 | | F70-F79 | Intellectual Disabilities |  | | 4,192 3,957 | 5.4% 5.1% | | 0.007 | 0.014 |
|  |  | 1 2 | | F01-F09 | Mental disorders due to known physiological conditions |  | | 3,235 3,136 | 4.2% 4.0% | | 0.205 | 0.006 |
|  |  | 1 2 | | F99-F99 | Unspecified mental disorder (F99) |  | | 1,908 1,945 | 2.5% 2.5% | | 0.546 | 0.003 |
|  |  | 1 2 | | E08-E13 | Diabetes mellitus |  | | 3,888 4,134 | 5.0% 5.3% | | 0.005 | 0.014 |
|  |  | 1 2 | | Q00-Q07 | Congenital malformations of the nervous system |  | | 2,061 2,011 | 2.6% 2.6% | | 0.427 | 0.004 |
|  |  | 1 2 | | Q60-Q64 | Congenital malformations of the urinary system |  | | 421 447 | 0.5% 0.6% | | 0.376 | 0.004 |
|  |  | 1 2 | | N45 | Orchitis and epididymitis |  | | 420 467 | 0.5% 0.6% | | 0.114 | 0.008 |
|  |  | 1 2 | | N43 | Hydrocele and spermatocele |  | | 455 490 | 0.6% 0.6% | | 0.253 | 0.006 |
|  |  | 1 2 | | N50.1 | Vascular disorders of male genital organs |  | | 36 35 | 0.0% 0.0% | | 0.906 | 0.001 |
|  |  | 1 2 | | N49 | Inflammatory disorders of male genital organs, not elsewhere classified |  | | 125 136 | 0.2% 0.2% | | 0.496 | 0.003 |
|  |  | 1 2 | | I86.1 | Scrotal varices |  | | 206 207 | 0.3% 0.3% | | 0.961 | <0.001 |
|  |  | 1 2 | | Q53 | Undescended and ectopic testicle |  | | 400 399 | 0.5% 0.5% | | 0.972 | <0.001 |
|  |  | 1 2 | | Q55 | Other congenital malformations of male genital organs |  | | 278 262 | 0.4% 0.3% | | 0.490 | 0.003 |
|  |  | 1 2 | | Q54 | Hypospadias |  | | 171 160 | 0.2% 0.2% | | 0.545 | 0.003 |
|  |  | 1 2 | | C60-C63 | Malignant neoplasms of male genital organs |  | | 90 90 | 0.1% 0.1% | | 1 | <0.001 |
|  |  | 1 2 | | N44.8 | Other noninflammatory disorders of the testis |  | | 208 227 | 0.3% 0.3% | | 0.362 | 0.005 |
|  |  | 1 2 | | N44.0 | Torsion of testis |  | | 55 60 | 0.1% 0.1% | | 0.641 | 0.002 |
|  |  | 1 2 | | N30-N39 | Other diseases of the urinary system |  | | 3,813 3,862 | 4.9% 5.0% | | 0.566 | 0.003 |
|  |  | 1 2 | | K40 | Inguinal hernia |  | | 723 786 | 0.9% 1.0% | | 0.103 | 0.008 |
|  |  | 1 2 | | E84 | Cystic fibrosis |  | | 58 68 | 0.1% 0.1% | | 0.373 | 0.005 |
|  |  | 1 2 | | S30-S39 | Injuries to the abdomen, lower back, lumbar spine, pelvis and external genitals |  | | 4,489 4,807 | 5.8% 6.2% | | 0.001 | 0.017 |
|  |  | 1 2 | | E23.0 | Hypopituitarism |  | | 283 286 | 0.4% 0.4% | | 0.900 | 0.001 |
|  |  | 1 2 | | R56 | Convulsions, not elsewhere classified |  | | 25,667 23,781 | 33.0% 30.6% | | <0.001 | 0.052 |
|  |  | 1 2 | | E34.5 | Androgen insensitivity syndrome |  | | 10 10 | 0.0% 0.0% | | 1 | <0.001 |
|  |  | 1 2 | | Q87.1 | Congenital malformation syndromes predominantly associated with short stature |  | | 69 66 | 0.1% 0.1% | | 0.796 | 0.001 |
|  |  | 1 2 | | F31 | Bipolar disorder |  | | 23,875 25,558 | 30.7% 32.8% | | <0.001 | 0.046 |
|  |  | 1 2 | | F32 | Depressive episode |  | | 13,038 13,916 | 16.8% 17.9% | | <0.001 | 0.030 |
|  |  | 1 2 | | F39 | Unspecified mood [affective] disorder |  | | 5,556 5,687 | 7.1% 7.3% | | 0.200 | 0.007 |
|  |  | 1 2 | | F33 | Major depressive disorder, recurrent |  | | 3,993 4,179 | 5.1% 5.4% | | 0.035 | 0.011 |
|  |  | 1 2 | | F34 | Persistent mood [affective] disorders |  | | 1,742 1,862 | 2.2% 2.4% | | 0.043 | 0.010 |
|  |  | 1 2 | | F30 | Manic episode |  | | 1,731 1,604 | 2.2% 2.1% | | 0.026 | 0.011 |
|  |  | 1 2 | | F50-F59 | Behavioral syndromes associated with physiological disturbances and physical factors |  | | 2,251 2,308 | 2.9% 3.0% | | 0.392 | 0.004 |
|  |  | 1 2 | | J00-J99 | Diseases of the respiratory system |  | | 24,237 24,466 | 31.1% 31.4% | | 0.211 | 0.006 |
|  |  | 1 2 | | I00-I99 | Diseases of the circulatory system |  | | 18,387 19,176 | 23.6% 24.6% | | <0.001 | 0.024 |
|  |  | 1 2 | | K00-K95 | Diseases of the digestive system |  | | 21,681 22,456 | 27.9% 28.9% | | <0.001 | 0.022 |
|  |  | 1 2 | | Q65-Q79 | Congenital malformations and deformations of the musculoskeletal system |  | | 2,044 1,948 | 2.6% 2.5% | | 0.124 | 0.008 |
|  |  | 1 2 | | N17-N19 | Acute kidney failure and chronic kidney disease |  | | 3,596 3,711 | 4.6% 4.8% | | 0.168 | 0.007 |
|  |  | 1 2 | | A50-A64 | Infections with a predominantly sexual mode of transmission |  | | 910 992 | 1.2% 1.3% | | 0.059 | 0.010 |
|  |  | 1 2 | | E03 | Other hypothyroidism |  | | 2,106 2,202 | 2.7% 2.8% | | 0.138 | 0.008 |
|  |  | 1 2 | | E05 | Thyrotoxicosis [hyperthyroidism] |  | | 303 304 | 0.4% 0.4% | | 0.968 | <0.001 |
|  |  | 1 2 | | E83.1 | Disorders of iron metabolism |  | | 81 80 | 0.1% 0.1% | | 0.937 | <0.001 |
|  |  | 1 2 | | Q99 | Other chromosome abnormalities, not elsewhere classified |  | | 616 650 | 0.8% 0.8% | | 0.337 | 0.005 |
|  |  | 1 2 | | Q93 | Monosomies and deletions from the autosomes, not elsewhere classified |  | | 316 313 | 0.4% 0.4% | | 0.905 | 0.001 |
|  |  | 1 2 | | Q90 | Down syndrome |  | | 264 313 | 0.3% 0.4% | | 0.041 | 0.010 |
|  |  | 1 2 | | Q92 | Other trisomies and partial trisomies of the autosomes, not elsewhere classified |  | | 111 115 | 0.1% 0.1% | | 0.790 | 0.001 |
|  |  | 1 2 | | Q98.4 | Klinefelter syndrome, unspecified |  | | 60 64 | 0.1% 0.1% | | 0.719 | 0.002 |
|  |  | 1 2 | | Q98.5 | Karyotype 47, XYY |  | | 19 23 | 0.0% 0.0% | | 0.537 | 0.003 |
|  |  | 1 2 | | Q98.7 | Male with sex chromosome mosaicism |  | | 18 22 | 0.0% 0.0% | | 0.527 | 0.003 |
|  |  | 1 2 | | Q98.8 | Other specified sex chromosome abnormalities, male phenotype |  | | 17 23 | 0.0% 0.0% | | 0.343 | 0.005 |
|  |  | 1 2 | | Q98.0 | Klinefelter syndrome karyotype 47, XXY |  | | 12 10 | 0.0% 0.0% | | 0.670 | 0.002 |
|  |  | 1 2 | | Q98.1 | Klinefelter syndrome, male with more than two X chromosomes |  | | 10 10 | 0.0% 0.0% | | 1 | <0.001 |
|  |  | 1 2 | | Q98.6 | Male with structurally abnormal sex chromosome |  | | 10 10 | 0.0% 0.0% | | 1 | <0.001 |
|  |  | 1 2 | | Q98.9 | Sex chromosome abnormality, male phenotype, unspecified |  | | 0 10 | 0% 0.0% | | 0.002 | 0.016 |
|  |  | 1 2 | | D35.2 | Benign neoplasm of pituitary gland |  | | 80 82 | 0.1% 0.1% | | 0.875 | 0.001 |
|  |  | 1 2 | | D35.3 | Benign neoplasm of craniopharyngeal duct |  | | 34 37 | 0.0% 0.0% | | 0.722 | 0.002 |
|  |  | 1 2 | | E40-E46 | Malnutrition |  | | 1,685 1,776 | 2.2% 2.3% | | 0.118 | 0.008 |
|  |  | 1 2 | | G40.9 | Epilepsy, unspecified |  | | 23,587 22,056 | 30.3% 28.3% | | <0.001 | 0.043 |
|  |  | 1 2 | | G40.3 | Generalized idiopathic epilepsy and epileptic syndromes |  | | 6,488 6,057 | 8.3% 7.8% | | <0.001 | 0.020 |
|  |  | 1 2 | | G40.4 | Other generalized epilepsy and epileptic syndromes |  | | 4,989 4,726 | 6.4% 6.1% | | 0.006 | 0.014 |
|  |  | 1 2 | | G40.2 | Localization-related (focal) (partial) symptomatic epilepsy and epileptic syndromes with complex partial seizures |  | | 5,031 4,348 | 6.5% 5.6% | | <0.001 | 0.037 |
|  |  | 1 2 | | G40.8 | Other epilepsy and recurrent seizures |  | | 3,843 3,706 | 4.9% 4.8% | | 0.106 | 0.008 |
|  |  | 1 2 | | G40.1 | Localization-related (focal) (partial) symptomatic epilepsy and epileptic syndromes with simple partial seizures |  | | 3,560 3,210 | 4.6% 4.1% | | <0.001 | 0.022 |
|  |  | 1 2 | | G40.A | Absence epileptic syndrome |  | | 1,866 1,774 | 2.4% 2.3% | | 0.123 | 0.008 |
|  |  | 1 2 | | G40.0 | Localization-related (focal) (partial) idiopathic epilepsy and epileptic syndromes with seizures of localized onset |  | | 1,130 1,009 | 1.5% 1.3% | | 0.008 | 0.013 |
|  |  | 1 2 | | G40.5 | Epileptic seizures related to external causes |  | | 675 796 | 0.9% 1.0% | | 0.002 | 0.016 |
|  |  | 1 2 | | G40.B | Juvenile myoclonic epilepsy [impulsive petit mal] |  | | 533 517 | 0.7% 0.7% | | 0.620 | 0.003 |
|  |  | 1 2 | | F17 | Nicotine dependence |  | | 14,967 15,864 | 19.2% 20.4% | | <0.001 | 0.029 |
|  |  | 1 2 | | F12 | Cannabis related disorders |  | | 9,542 9,825 | 12.3% 12.6% | | 0.030 | 0.011 |
|  |  | 1 2 | | F10 | Alcohol related disorders |  | | 8,132 8,640 | 10.4% 11.1% | | <0.001 | 0.021 |
|  |  | 1 2 | | F19 | Other psychoactive substance related disorders |  | | 6,653 6,917 | 8.5% 8.9% | | 0.018 | 0.012 |
|  |  | 1 2 | | F11 | Opioid related disorders |  | | 3,941 4,189 | 5.1% 5.4% | | 0.005 | 0.014 |
|  |  | 1 2 | | F14 | Cocaine related disorders |  | | 3,330 3,472 | 4.3% 4.5% | | 0.078 | 0.009 |
|  |  | 1 2 | | F15 | Other stimulant related disorders |  | | 3,277 3,427 | 4.2% 4.4% | | 0.061 | 0.009 |
|  |  | 1 2 | | F13 | Sedative, hypnotic, or anxiolytic related disorders |  | | 1,665 1,737 | 2.1% 2.2% | | 0.212 | 0.006 |
|  |  | 1 2 | | F18 | Inhalant related disorders |  | | 1,330 1,423 | 1.7% 1.8% | | 0.074 | 0.009 |
|  |  | 1 2 | | F16 | Hallucinogen related disorders |  | | 746 778 | 1.0% 1.0% | | 0.410 | 0.004 |
|  | **Procedure** | | | | | | | | | | | |
|  |  | Cohort | | |  | Mean ± SD | | Patients | % of Cohort | | P-Value | SMD |
|  |  | 1 2 | | 1010843 | Radiation Oncology Treatment |  | | 149 174 | 0.2% 0.2% | | 0.164 | 0.007 |
|  |  | 1 2 | | 1008061 | Surgical Procedures on the Urinary System |  | | 2,027 2,048 | 2.6% 2.6% | | 0.739 | 0.002 |
|  |  | 1 2 | | 1008011 | Repair initial inguinal hernia, age 5 years or older |  | | 103 113 | 0.1% 0.1% | | 0.496 | 0.003 |
|  |  | 1 2 | | 1008470 | Surgical Procedures on the Male Genital System |  | | 698 685 | 0.9% 0.9% | | 0.725 | 0.002 |
|  | **Medication** | | | | | | | | | | | |
|  |  | Cohort | | |  | Mean ± SD | | Patients | % of Cohort | | P-Value | SMD |
|  |  | 1 2 | | 25025 | finasteride |  | | 183 214 | 0.2% 0.3% | | 0.119 | 0.008 |
|  |  | 1 2 | | CN750 | LITHIUM SALTS |  | | 3,293 3,352 | 4.2% 4.3% | | 0.459 | 0.004 |
|  |  | 1 2 | | 6135 | ketoconazole |  | | 1,067 1,134 | 1.4% 1.5% | | 0.150 | 0.007 |
|  |  | 1 2 | | 10829 | trimethoprim |  | | 4,315 4,601 | 5.5% 5.9% | | 0.002 | 0.016 |
|  |  | 1 2 | | 7454 | nitrofurantoin |  | | 340 367 | 0.4% 0.5% | | 0.309 | 0.005 |
|  |  | 1 2 | | AM200 | ERYTHROMYCINS/MACROLIDES |  | | 6,690 7,080 | 8.6% 9.1% | | <0.001 | 0.018 |
|  |  | 1 2 | | AM300 | AMINOGLYCOSIDES |  | | 3,415 3,550 | 4.4% 4.6% | | 0.098 | 0.008 |
|  |  | 1 2 | | AN000 | ANTINEOPLASTICS |  | | 1,236 1,343 | 1.6% 1.7% | | 0.034 | 0.011 |
|  |  | 1 2 | | CN709 | ANTIPSYCHOTICS,OTHER |  | | 29,950 31,013 | 38.5% 39.8% | | <0.001 | 0.028 |
|  |  | 1 2 | | CN701 | PHENOTHIAZINE/RELATED ANTIPSYCHOTICS |  | | 1,754 1,603 | 2.3% 2.1% | | 0.008 | 0.013 |
|  |  | 1 2 | | CN609 | ANTIDEPRESSANTS,OTHER |  | | 22,490 23,976 | 28.9% 30.8% | | <0.001 | 0.042 |
|  |  | 1 2 | | CN601 | TRICYCLIC ANTIDEPRESSANTS |  | | 2,525 2,677 | 3.2% 3.4% | | 0.032 | 0.011 |
|  |  | 1 2 | | CV100 | BETA BLOCKERS/RELATED |  | | 8,254 8,825 | 10.6% 11.3% | | <0.001 | 0.023 |
|  |  | 1 2 | | CV200 | CALCIUM CHANNEL BLOCKERS |  | | 4,154 4,384 | 5.3% 5.6% | | 0.010 | 0.013 |
|  |  | 1 2 | | CV800 | ACE INHIBITORS |  | | 3,828 4,071 | 4.9% 5.2% | | 0.005 | 0.014 |
|  |  | 1 2 | | CV150 | ALPHA BLOCKERS/RELATED |  | | 2,944 3,147 | 3.8% 4.0% | | 0.008 | 0.013 |
|  |  | 1 2 | | CV490 | ANTIHYPERTENSIVES,OTHER |  | | 9,940 10,401 | 12.8% 13.4% | | 0.001 | 0.018 |
|  |  | 1 2 | | HS100 | ANDROGENS/ANABOLICS |  | | 483 534 | 0.6% 0.7% | | 0.109 | 0.008 |
|  |  | 1 2 | | HS800 | PROGESTINS |  | | 213 243 | 0.3% 0.3% | | 0.159 | 0.007 |
|  |  | 1 2 | | HS300 | ESTROGENS |  | | 144 150 | 0.2% 0.2% | | 0.726 | 0.002 |
|  |  | 1 2 | | 9997 | spironolactone |  | | 547 582 | 0.7% 0.7% | | 0.296 | 0.005 |
|  |  | 1 2 | | 3014 | cyproterone |  | | 10 10 | 0.0% 0.0% | | 1 | <0.001 |
|  |  | 1 2 | | 114477 | levetiracetam |  | | 15,851 16,204 | 20.4% 20.8% | | 0.027 | 0.011 |
|  |  | 1 2 | | 28439 | lamotrigine |  | | 6,120 6,507 | 7.9% 8.4% | | <0.001 | 0.018 |
|  |  | 1 2 | | 25480 | gabapentin |  | | 7,111 7,623 | 9.1% 9.8% | | <0.001 | 0.022 |
|  |  | 1 2 | | 38404 | topiramate |  | | 3,569 3,604 | 4.6% 4.6% | | 0.672 | 0.002 |
|  |  | 1 2 | | 32624 | oxcarbazepine |  | | 4,025 4,106 | 5.2% 5.3% | | 0.356 | 0.005 |
|  |  | 1 2 | | 623400 | lacosamide |  | | 2,516 2,523 | 3.2% 3.2% | | 0.920 | 0.001 |
|  |  | 1 2 | | 8183 | phenytoin |  | | 2,641 2,811 | 3.4% 3.6% | | 0.019 | 0.012 |
|  |  | 1 2 | | 21241 | clobazam |  | | 1,748 1,719 | 2.2% 2.2% | | 0.618 | 0.003 |
|  |  | 1 2 | | 2002 | carbamazepine |  | | 2,474 2,608 | 3.2% 3.4% | | 0.056 | 0.010 |
|  |  | 1 2 | | 39998 | zonisamide |  | | 1,676 1,665 | 2.2% 2.1% | | 0.847 | 0.001 |
|  |  | 1 2 | | 72236 | fosphenytoin |  | | 1,261 1,205 | 1.6% 1.5% | | 0.256 | 0.006 |
|  |  | 1 2 | | 4135 | ethosuximide |  | | 808 740 | 1.0% 1.0% | | 0.082 | 0.009 |
|  |  | 1 2 | | 69036 | rufinamide |  | | 291 273 | 0.4% 0.4% | | 0.448 | 0.004 |
|  |  | 1 2 | | 1739745 | brivaracetam |  | | 256 242 | 0.3% 0.3% | | 0.530 | 0.003 |
|  |  | 1 2 | | 14851 | vigabatrin |  | | 240 247 | 0.3% 0.3% | | 0.751 | 0.002 |
|  |  | 1 2 | | 24812 | felbamate |  | | 184 179 | 0.2% 0.2% | | 0.793 | 0.001 |
|  |  | 1 2 | | 1356552 | perampanel |  | | 209 227 | 0.3% 0.3% | | 0.388 | 0.004 |
|  |  | 1 2 | | 2045371 | cannabidiol |  | | 179 183 | 0.2% 0.2% | | 0.833 | 0.001 |
|  |  | 1 2 | | 1482502 | eslicarbazepine |  | | 126 134 | 0.2% 0.2% | | 0.620 | 0.003 |
|  |  | 1 2 | | 8691 | primidone |  | | 124 127 | 0.2% 0.2% | | 0.850 | 0.001 |
|  |  | 1 2 | | 2265690 | cenobamate |  | | 68 67 | 0.1% 0.1% | | 0.931 | <0.001 |
|  |  | 1 2 | | 31914 | tiagabine |  | | 27 31 | 0.0% 0.0% | | 0.599 | 0.003 |
|  |  | 1 2 | | 4328 | fenfluramine |  | | 13 10 | 0.0% 0.0% | | 0.532 | 0.003 |
|  |  | 1 2 | | 47858 | methsuximide |  | | 10 10 | 0.0% 0.0% | | 1 | <0.001 |
|  |  | 1 2 | | 2054968 | stiripentol |  | | 10 10 | 0.0% 0.0% | | 1 | <0.001 |
|  |  | 1 2 | | CN302 | BENZODIAZEPINE DERIVATIVE SEDATIVES/HYPNOTICS |  | | 39,551 40,976 | 50.8% 52.7% | | <0.001 | 0.037 |
|  |  | 1 2 | | CN309 | SEDATIVES/HYPNOTICS,OTHER |  | | 8,235 8,521 | 10.6% 10.9% | | 0.019 | 0.012 |
|  |  | 1 2 | | CN301 | BARBITURIC ACID DERIVATIVE SEDATIVES/HYPNOTICS |  | | 2,248 2,304 | 2.9% 3.0% | | 0.400 | 0.004 |
|  |  | 1 2 | | CN101 | OPIOID ANALGESICS |  | | 22,662 24,697 | 29.1% 31.7% | | <0.001 | 0.057 |
|  |  | 1 2 | | HS051 | GLUCOCORTICOIDS |  | | 18,215 19,318 | 23.4% 24.8% | | <0.001 | 0.033 |
|  | **Laboratory** | | | | | | | | | | | |
|  |  | Cohort | | |  | Mean ± SD | | Patients | % of Cohort | | P-Value | SMD |
|  |  | 1 2 | | 9083 | BMI | 26.0 +/- 7.0 26.3 +/- 7.2 | | 39,641 42,096 | 50.9% 54.1% | | <0.001 | 0.043 |
|  |  | 1 2 | |  | 0 - 0 kg/m2 |  | | 39,714 42,172 | 51.0% 54.2% | | <0.001 | 0.063 |

**Abbreviations:** BMI = Body Mass Index; SD = Standard deviation; SMD = Standardised mean difference

**N.B.:** Uncorrected p-values were calculated using two-sided t-tests for continuous covariates and two-sided Z-tests for categorical covariates. These p-values reflect descriptive comparisons of baseline characteristics during propensity score matching. Correction for multiple comparisons is not appropriate in this context, as the aim is to assess covariate balance (reflected by a standardised mean difference of <0.1) rather than to test hypotheses. Exact p-values beyond three decimal places are not provided in the propensity score matching outputs generated by the TriNetX platform. Where p-values fall below this threshold, they are reported as <0.001.

# Supplementary Table 4: Propensity score matching men with epilepsy or bipolar disorder exposed (cohort 1) and unexposed (cohort 2) to valproate – 60-day outcome assessment

Propensity score matching was performed on all listed characteristics. Characteristics of the cohorts before and after matching are summarized in the table below.

| **Cohort 1 and cohort 2 patient count before and after propensity score matching** | | | | | | | | | | | | |
| --- | --- | --- | --- | --- | --- | --- | --- | --- | --- | --- | --- | --- |
|  | | | Cohort | | | Patient count before matching | | | | Patient count after matching | | |
|  | | | 1 - Men with epilepsy or bipolar disorder exposed to valproate_v9 | | | 89,759 | | | | 77,099 | | |
|  | | | 2 - Men with epilepsy or bipolar disorder not exposed to valproate_v9 | | | 514,623 | | | | 77,099 | | |
| **Propensity score density function - Before and after matching (cohort 1 - purple, cohort 2 - green)** | | | | | | | | | | | | |
|  |  | | 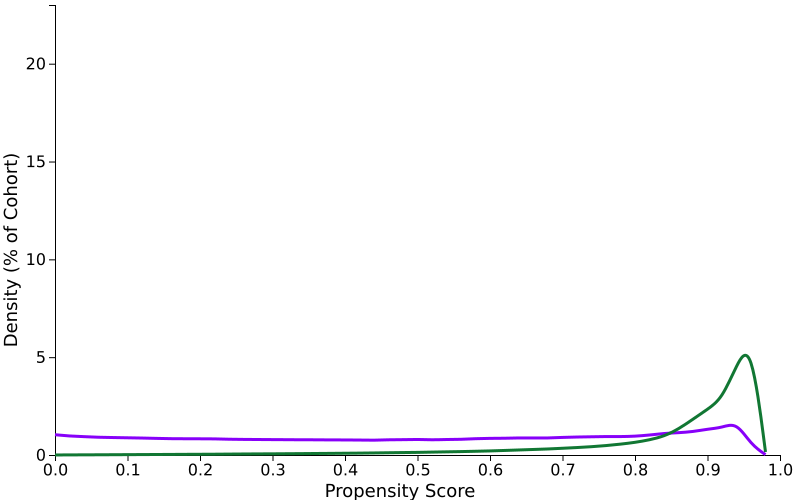 | | | | 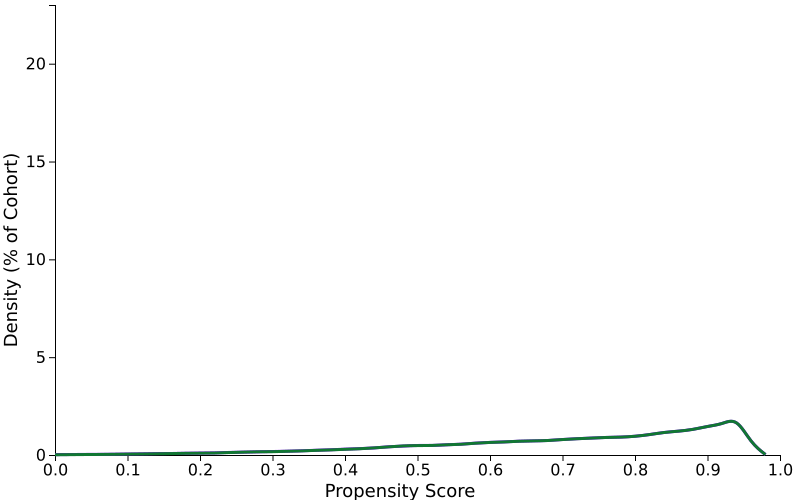 | | | | | |
| **Cohort 1 (N = 89,759) and cohort 2 (N = 514,623) characteristics before propensity score matching** | | | | | | | | | | | | |
|  | **Demographics** | | | | | | | | | | | |
|  |  | Cohort | | |  | Mean ± SD | | Patients | % of Cohort | | P-Value | SMD |
|  |  | 1 2 | | AI | Age at Index | 24.9 +/- 12.9 21.0 +/- 14.9 | | 87,251 493,641 | 100% 100% | | <0.001 | 0.283 |
|  |  | 1 2 | | 2106-3 | White |  | | 50,983 282,710 | 58.4% 57.3% | | <0.001 | 0.024 |
|  |  | 1 2 | | 1002-5 | American Indian or Alaska Native |  | | 351 1,739 | 0.4% 0.4% | | 0.023 | 0.008 |
|  |  | 1 2 | | UNK | Unknown Race |  | | 11,552 78,500 | 13.2% 15.9% | | <0.001 | 0.076 |
|  |  | 1 2 | | 2076-8 | Native Hawaiian or Other Pacific Islander |  | | 341 2,297 | 0.4% 0.5% | | 0.003 | 0.011 |
|  |  | 1 2 | | 2054-5 | Black or African American |  | | 15,816 83,023 | 18.1% 16.8% | | <0.001 | 0.034 |
|  |  | 1 2 | | 2131-1 | Other Race |  | | 4,585 29,432 | 5.3% 6.0% | | <0.001 | 0.031 |
|  |  | 1 2 | | 2028-9 | Asian |  | | 3,623 15,940 | 4.2% 3.2% | | <0.001 | 0.049 |
|  | **Diagnosis** | | | | | | | | | | | |
|  |  | Cohort | | |  | Mean ± SD | | Patients | % of Cohort | | P-Value | SMD |
|  |  | 1 2 | | F80-F89 | Pervasive and specific developmental disorders |  | | 14,101 45,978 | 16.2% 9.3% | | <0.001 | 0.206 |
|  |  | 1 2 | | F40-F48 | Anxiety, dissociative, stress-related, somatoform and other nonpsychotic mental disorders |  | | 24,360 85,265 | 27.9% 17.3% | | <0.001 | 0.257 |
|  |  | 1 2 | | F90-F98 | Behavioral and emotional disorders with onset usually occurring in childhood and adolescence |  | | 15,687 49,699 | 18.0% 10.1% | | <0.001 | 0.229 |
|  |  | 1 2 | | F20-F29 | Schizophrenia, schizotypal, delusional, and other non-mood psychotic disorders |  | | 13,777 25,909 | 15.8% 5.2% | | <0.001 | 0.349 |
|  |  | 1 2 | | F60-F69 | Disorders of adult personality and behavior |  | | 7,312 13,396 | 8.4% 2.7% | | <0.001 | 0.249 |
|  |  | 1 2 | | F70-F79 | Intellectual Disabilities |  | | 6,463 9,365 | 7.4% 1.9% | | <0.001 | 0.264 |
|  |  | 1 2 | | F01-F09 | Mental disorders due to known physiological conditions |  | | 4,595 8,245 | 5.3% 1.7% | | <0.001 | 0.197 |
|  |  | 1 2 | | F99-F99 | Unspecified mental disorder (F99) |  | | 2,421 4,597 | 2.8% 0.9% | | <0.001 | 0.137 |
|  |  | 1 2 | | E08-E13 | Diabetes mellitus |  | | 4,329 17,769 | 5.0% 3.6% | | <0.001 | 0.067 |
|  |  | 1 2 | | Q00-Q07 | Congenital malformations of the nervous system |  | | 3,204 9,860 | 3.7% 2.0% | | <0.001 | 0.101 |
|  |  | 1 2 | | Q60-Q64 | Congenital malformations of the urinary system |  | | 549 2,912 | 0.6% 0.6% | | 0.164 | 0.005 |
|  |  | 1 2 | | N45 | Orchitis and epididymitis |  | | 463 1,798 | 0.5% 0.4% | | <0.001 | 0.025 |
|  |  | 1 2 | | N43 | Hydrocele and spermatocele |  | | 559 2,731 | 0.6% 0.6% | | 0.002 | 0.011 |
|  |  | 1 2 | | N50.1 | Vascular disorders of male genital organs |  | | 44 148 | 0.1% 0.0% | | 0.002 | 0.010 |
|  |  | 1 2 | | N49 | Inflammatory disorders of male genital organs, not elsewhere classified |  | | 138 545 | 0.2% 0.1% | | <0.001 | 0.013 |
|  |  | 1 2 | | I86.1 | Scrotal varices |  | | 238 1,039 | 0.3% 0.2% | | <0.001 | 0.013 |
|  |  | 1 2 | | Q53 | Undescended and ectopic testicle |  | | 576 2,806 | 0.7% 0.6% | | 0.001 | 0.012 |
|  |  | 1 2 | | Q55 | Other congenital malformations of male genital organs |  | | 391 2,556 | 0.4% 0.5% | | 0.008 | 0.010 |
|  |  | 1 2 | | Q54 | Hypospadias |  | | 218 1,511 | 0.2% 0.3% | | 0.005 | 0.011 |
|  |  | 1 2 | | C60-C63 | Malignant neoplasms of male genital organs |  | | 91 543 | 0.1% 0.1% | | 0.638 | 0.002 |
|  |  | 1 2 | | N44.8 | Other noninflammatory disorders of the testis |  | | 248 720 | 0.3% 0.1% | | <0.001 | 0.030 |
|  |  | 1 2 | | N44.0 | Torsion of testis |  | | 60 299 | 0.1% 0.1% | | 0.369 | 0.003 |
|  |  | 1 2 | | N30-N39 | Other diseases of the urinary system |  | | 4,759 15,978 | 5.5% 3.2% | | <0.001 | 0.109 |
|  |  | 1 2 | | K40 | Inguinal hernia |  | | 888 4,898 | 1.0% 1.0% | | 0.484 | 0.003 |
|  |  | 1 2 | | E84 | Cystic fibrosis |  | | 73 334 | 0.1% 0.1% | | 0.100 | 0.006 |
|  |  | 1 2 | | S30-S39 | Injuries to the abdomen, lower back, lumbar spine, pelvis and external genitals |  | | 5,220 18,766 | 6.0% 3.8% | | <0.001 | 0.101 |
|  |  | 1 2 | | E23.0 | Hypopituitarism |  | | 387 1,489 | 0.4% 0.3% | | <0.001 | 0.023 |
|  |  | 1 2 | | R56 | Convulsions, not elsewhere classified |  | | 31,822 241,632 | 36.5% 48.9% | | <0.001 | 0.254 |
|  |  | 1 2 | | E34.5 | Androgen insensitivity syndrome |  | | 10 15 | 0.0% 0.0% | | <0.001 | 0.010 |
|  |  | 1 2 | | Q87.1 | Congenital malformation syndromes predominantly associated with short stature |  | | 93 357 | 0.1% 0.1% | | 0.001 | 0.011 |
|  |  | 1 2 | | F31 | Bipolar disorder |  | | 25,397 120,525 | 29.1% 24.4% | | <0.001 | 0.106 |
|  |  | 1 2 | | F32 | Depressive episode |  | | 14,868 48,728 | 17.0% 9.9% | | <0.001 | 0.211 |
|  |  | 1 2 | | F39 | Unspecified mood [affective] disorder |  | | 7,021 12,842 | 8.0% 2.6% | | <0.001 | 0.244 |
|  |  | 1 2 | | F33 | Major depressive disorder, recurrent |  | | 4,475 13,891 | 5.1% 2.8% | | <0.001 | 0.119 |
|  |  | 1 2 | | F34 | Persistent mood [affective] disorders |  | | 2,200 5,289 | 2.5% 1.1% | | <0.001 | 0.109 |
|  |  | 1 2 | | F30 | Manic episode |  | | 2,234 2,721 | 2.6% 0.6% | | <0.001 | 0.163 |
|  |  | 1 2 | | F50-F59 | Behavioral syndromes associated with physiological disturbances and physical factors |  | | 2,740 8,198 | 3.1% 1.7% | | <0.001 | 0.097 |
|  |  | 1 2 | | J00-J99 | Diseases of the respiratory system |  | | 28,901 144,380 | 33.1% 29.2% | | <0.001 | 0.084 |
|  |  | 1 2 | | I00-I99 | Diseases of the circulatory system |  | | 21,722 90,926 | 24.9% 18.4% | | <0.001 | 0.158 |
|  |  | 1 2 | | K00-K95 | Diseases of the digestive system |  | | 25,937 117,080 | 29.7% 23.7% | | <0.001 | 0.136 |
|  |  | 1 2 | | Q65-Q79 | Congenital malformations and deformations of the musculoskeletal system |  | | 2,886 14,947 | 3.3% 3.0% | | <0.001 | 0.016 |
|  |  | 1 2 | | N17-N19 | Acute kidney failure and chronic kidney disease |  | | 4,195 16,793 | 4.8% 3.4% | | <0.001 | 0.071 |
|  |  | 1 2 | | A50-A64 | Infections with a predominantly sexual mode of transmission |  | | 997 4,138 | 1.1% 0.8% | | <0.001 | 0.031 |
|  |  | 1 2 | | E03 | Other hypothyroidism |  | | 2,620 7,795 | 3.0% 1.6% | | <0.001 | 0.095 |
|  |  | 1 2 | | E05 | Thyrotoxicosis [hyperthyroidism] |  | | 372 1,094 | 0.4% 0.2% | | <0.001 | 0.036 |
|  |  | 1 2 | | E83.1 | Disorders of iron metabolism |  | | 97 550 | 0.1% 0.1% | | 0.984 | <0.001 |
|  |  | 1 2 | | Q99 | Other chromosome abnormalities, not elsewhere classified |  | | 1,067 2,854 | 1.2% 0.6% | | <0.001 | 0.068 |
|  |  | 1 2 | | Q93 | Monosomies and deletions from the autosomes, not elsewhere classified |  | | 484 1,575 | 0.6% 0.3% | | <0.001 | 0.036 |
|  |  | 1 2 | | Q90 | Down syndrome |  | | 325 1,721 | 0.4% 0.3% | | 0.273 | 0.004 |
|  |  | 1 2 | | Q92 | Other trisomies and partial trisomies of the autosomes, not elsewhere classified |  | | 196 535 | 0.2% 0.1% | | <0.001 | 0.029 |
|  |  | 1 2 | | Q98.4 | Klinefelter syndrome, unspecified |  | | 82 224 | 0.1% 0.0% | | <0.001 | 0.018 |
|  |  | 1 2 | | Q98.5 | Karyotype 47, XYY |  | | 31 94 | 0.0% 0.0% | | 0.002 | 0.010 |
|  |  | 1 2 | | Q98.7 | Male with sex chromosome mosaicism |  | | 31 59 | 0.0% 0.0% | | <0.001 | 0.015 |
|  |  | 1 2 | | Q98.8 | Other specified sex chromosome abnormalities, male phenotype |  | | 31 67 | 0.0% 0.0% | | <0.001 | 0.014 |
|  |  | 1 2 | | Q98.0 | Klinefelter syndrome karyotype 47, XXY |  | | 16 50 | 0.0% 0.0% | | 0.036 | 0.007 |
|  |  | 1 2 | | Q98.1 | Klinefelter syndrome, male with more than two X chromosomes |  | | 10 15 | 0.0% 0.0% | | <0.001 | 0.010 |
|  |  | 1 2 | | Q98.6 | Male with structurally abnormal sex chromosome |  | | 10 10 | 0.0% 0.0% | | <0.001 | 0.011 |
|  |  | 1 2 | | Q98.9 | Sex chromosome abnormality, male phenotype, unspecified |  | | 10 10 | 0.0% 0.0% | | <0.001 | 0.011 |
|  |  | 1 2 | | D35.2 | Benign neoplasm of pituitary gland |  | | 97 370 | 0.1% 0.1% | | 0.001 | 0.012 |
|  |  | 1 2 | | D35.3 | Benign neoplasm of craniopharyngeal duct |  | | 39 145 | 0.0% 0.0% | | 0.019 | 0.008 |
|  |  | 1 2 | | E40-E46 | Malnutrition |  | | 2,198 7,403 | 2.5% 1.5% | | <0.001 | 0.073 |
|  |  | 1 2 | | G40.9 | Epilepsy, unspecified |  | | 30,535 96,038 | 35.0% 19.5% | | <0.001 | 0.355 |
|  |  | 1 2 | | G40.3 | Generalized idiopathic epilepsy and epileptic syndromes |  | | 10,630 14,789 | 12.2% 3.0% | | <0.001 | 0.352 |
|  |  | 1 2 | | G40.4 | Other generalized epilepsy and epileptic syndromes |  | | 8,770 12,704 | 10.1% 2.6% | | <0.001 | 0.311 |
|  |  | 1 2 | | G40.2 | Localization-related (focal) (partial) symptomatic epilepsy and epileptic syndromes with complex partial seizures |  | | 7,965 18,800 | 9.1% 3.8% | | <0.001 | 0.218 |
|  |  | 1 2 | | G40.8 | Other epilepsy and recurrent seizures |  | | 6,548 17,140 | 7.5% 3.5% | | <0.001 | 0.178 |
|  |  | 1 2 | | G40.1 | Localization-related (focal) (partial) symptomatic epilepsy and epileptic syndromes with simple partial seizures |  | | 6,206 14,299 | 7.1% 2.9% | | <0.001 | 0.194 |
|  |  | 1 2 | | G40.A | Absence epileptic syndrome |  | | 3,214 5,043 | 3.7% 1.0% | | <0.001 | 0.176 |
|  |  | 1 2 | | G40.0 | Localization-related (focal) (partial) idiopathic epilepsy and epileptic syndromes with seizures of localized onset |  | | 2,044 4,642 | 2.3% 0.9% | | <0.001 | 0.111 |
|  |  | 1 2 | | G40.5 | Epileptic seizures related to external causes |  | | 1,228 2,587 | 1.4% 0.5% | | <0.001 | 0.090 |
|  |  | 1 2 | | G40.B | Juvenile myoclonic epilepsy [impulsive petit mal] |  | | 885 711 | 1.0% 0.1% | | <0.001 | 0.115 |
|  |  | 1 2 | | F17 | Nicotine dependence |  | | 16,842 62,898 | 19.3% 12.7% | | <0.001 | 0.180 |
|  |  | 1 2 | | F12 | Cannabis related disorders |  | | 11,167 28,562 | 12.8% 5.8% | | <0.001 | 0.243 |
|  |  | 1 2 | | F10 | Alcohol related disorders |  | | 8,923 36,865 | 10.2% 7.5% | | <0.001 | 0.097 |
|  |  | 1 2 | | F19 | Other psychoactive substance related disorders |  | | 7,557 21,897 | 8.7% 4.4% | | <0.001 | 0.171 |
|  |  | 1 2 | | F11 | Opioid related disorders |  | | 4,414 16,175 | 5.1% 3.3% | | <0.001 | 0.089 |
|  |  | 1 2 | | F14 | Cocaine related disorders |  | | 3,822 10,348 | 4.4% 2.1% | | <0.001 | 0.129 |
|  |  | 1 2 | | F15 | Other stimulant related disorders |  | | 3,756 10,253 | 4.3% 2.1% | | <0.001 | 0.127 |
|  |  | 1 2 | | F13 | Sedative, hypnotic, or anxiolytic related disorders |  | | 1,967 5,339 | 2.3% 1.1% | | <0.001 | 0.092 |
|  |  | 1 2 | | F18 | Inhalant related disorders |  | | 1,642 4,455 | 1.9% 0.9% | | <0.001 | 0.084 |
|  |  | 1 2 | | F16 | Hallucinogen related disorders |  | | 885 1,937 | 1.0% 0.4% | | <0.001 | 0.074 |
|  | **Procedure** | | | | | | | | | | | |
|  |  | Cohort | | |  | Mean ± SD | | Patients | % of Cohort | | P-Value | SMD |
|  |  | 1 2 | | 1010843 | Radiation Oncology Treatment |  | | 187 790 | 0.2% 0.2% | | <0.001 | 0.013 |
|  |  | 1 2 | | 1008061 | Surgical Procedures on the Urinary System |  | | 2,636 9,094 | 3.0% 1.8% | | <0.001 | 0.077 |
|  |  | 1 2 | | 1008011 | Repair initial inguinal hernia, age 5 years or older |  | | 127 450 | 0.1% 0.1% | | <0.001 | 0.016 |
|  |  | 1 2 | | 1008470 | Surgical Procedures on the Male Genital System |  | | 901 5,319 | 1.0% 1.1% | | 0.235 | 0.004 |
|  | **Medication** | | | | | | | | | | | |
|  |  | Cohort | | |  | Mean ± SD | | Patients | % of Cohort | | P-Value | SMD |
|  |  | 1 2 | | 25025 | finasteride |  | | 197 827 | 0.2% 0.2% | | <0.001 | 0.013 |
|  |  | 1 2 | | CN750 | LITHIUM SALTS |  | | 3,936 7,398 | 4.5% 1.5% | | <0.001 | 0.177 |
|  |  | 1 2 | | 6135 | ketoconazole |  | | 1,337 4,851 | 1.5% 1.0% | | <0.001 | 0.049 |
|  |  | 1 2 | | 10829 | trimethoprim |  | | 5,169 19,759 | 5.9% 4.0% | | <0.001 | 0.089 |
|  |  | 1 2 | | 7454 | nitrofurantoin |  | | 429 1,180 | 0.5% 0.2% | | <0.001 | 0.042 |
|  |  | 1 2 | | AM200 | ERYTHROMYCINS/MACROLIDES |  | | 7,878 29,978 | 9.0% 6.1% | | <0.001 | 0.112 |
|  |  | 1 2 | | AM300 | AMINOGLYCOSIDES |  | | 4,329 14,217 | 5.0% 2.9% | | <0.001 | 0.107 |
|  |  | 1 2 | | AN000 | ANTINEOPLASTICS |  | | 1,500 6,404 | 1.7% 1.3% | | <0.001 | 0.035 |
|  |  | 1 2 | | CN709 | ANTIPSYCHOTICS,OTHER |  | | 34,818 59,056 | 39.9% 12.0% | | <0.001 | 0.673 |
|  |  | 1 2 | | CN701 | PHENOTHIAZINE/RELATED ANTIPSYCHOTICS |  | | 2,801 2,119 | 3.2% 0.4% | | <0.001 | 0.209 |
|  |  | 1 2 | | CN609 | ANTIDEPRESSANTS,OTHER |  | | 26,334 62,360 | 30.2% 12.6% | | <0.001 | 0.438 |
|  |  | 1 2 | | CN601 | TRICYCLIC ANTIDEPRESSANTS |  | | 3,012 7,096 | 3.5% 1.4% | | <0.001 | 0.131 |
|  |  | 1 2 | | CV100 | BETA BLOCKERS/RELATED |  | | 9,557 25,525 | 11.0% 5.2% | | <0.001 | 0.214 |
|  |  | 1 2 | | CV200 | CALCIUM CHANNEL BLOCKERS |  | | 4,763 14,679 | 5.5% 3.0% | | <0.001 | 0.124 |
|  |  | 1 2 | | CV800 | ACE INHIBITORS |  | | 4,426 14,412 | 5.1% 2.9% | | <0.001 | 0.110 |
|  |  | 1 2 | | CV150 | ALPHA BLOCKERS/RELATED |  | | 3,508 7,587 | 4.0% 1.5% | | <0.001 | 0.152 |
|  |  | 1 2 | | CV490 | ANTIHYPERTENSIVES,OTHER |  | | 12,313 29,867 | 14.1% 6.1% | | <0.001 | 0.270 |
|  |  | 1 2 | | HS100 | ANDROGENS/ANABOLICS |  | | 549 2,326 | 0.6% 0.5% | | <0.001 | 0.021 |
|  |  | 1 2 | | HS800 | PROGESTINS |  | | 256 787 | 0.3% 0.2% | | <0.001 | 0.028 |
|  |  | 1 2 | | HS300 | ESTROGENS |  | | 163 734 | 0.2% 0.1% | | 0.008 | 0.009 |
|  |  | 1 2 | | 9997 | spironolactone |  | | 605 3,287 | 0.7% 0.7% | | 0.358 | 0.003 |
|  |  | 1 2 | | 3014 | cyproterone |  | | 10 10 | 0.0% 0.0% | | <0.001 | 0.011 |
|  |  | 1 2 | | 114477 | levetiracetam |  | | 20,701 61,461 | 23.7% 12.5% | | <0.001 | 0.296 |
|  |  | 1 2 | | 28439 | lamotrigine |  | | 7,856 21,972 | 9.0% 4.5% | | <0.001 | 0.183 |
|  |  | 1 2 | | 25480 | gabapentin |  | | 8,217 22,233 | 9.4% 4.5% | | <0.001 | 0.194 |
|  |  | 1 2 | | 38404 | topiramate |  | | 5,549 7,783 | 6.4% 1.6% | | <0.001 | 0.247 |
|  |  | 1 2 | | 32624 | oxcarbazepine |  | | 5,856 11,998 | 6.7% 2.4% | | <0.001 | 0.206 |
|  |  | 1 2 | | 623400 | lacosamide |  | | 4,152 4,982 | 4.8% 1.0% | | <0.001 | 0.225 |
|  |  | 1 2 | | 8183 | phenytoin |  | | 3,549 7,989 | 4.1% 1.6% | | <0.001 | 0.148 |
|  |  | 1 2 | | 21241 | clobazam |  | | 3,439 2,532 | 3.9% 0.5% | | <0.001 | 0.234 |
|  |  | 1 2 | | 2002 | carbamazepine |  | | 3,283 7,635 | 3.8% 1.5% | | <0.001 | 0.138 |
|  |  | 1 2 | | 39998 | zonisamide |  | | 3,039 3,461 | 3.5% 0.7% | | <0.001 | 0.195 |
|  |  | 1 2 | | 72236 | fosphenytoin |  | | 2,279 3,659 | 2.6% 0.7% | | <0.001 | 0.146 |
|  |  | 1 2 | | 4135 | ethosuximide |  | | 1,374 1,303 | 1.6% 0.3% | | <0.001 | 0.138 |
|  |  | 1 2 | | 69036 | rufinamide |  | | 613 357 | 0.7% 0.1% | | <0.001 | 0.102 |
|  |  | 1 2 | | 1739745 | brivaracetam |  | | 481 485 | 0.6% 0.1% | | <0.001 | 0.080 |
|  |  | 1 2 | | 14851 | vigabatrin |  | | 610 306 | 0.7% 0.1% | | <0.001 | 0.104 |
|  |  | 1 2 | | 24812 | felbamate |  | | 354 276 | 0.4% 0.1% | | <0.001 | 0.073 |
|  |  | 1 2 | | 1356552 | perampanel |  | | 410 368 | 0.5% 0.1% | | <0.001 | 0.076 |
|  |  | 1 2 | | 2045371 | cannabidiol |  | | 421 317 | 0.5% 0.1% | | <0.001 | 0.080 |
|  |  | 1 2 | | 1482502 | eslicarbazepine |  | | 202 353 | 0.2% 0.1% | | <0.001 | 0.041 |
|  |  | 1 2 | | 8691 | primidone |  | | 156 294 | 0.2% 0.1% | | <0.001 | 0.035 |
|  |  | 1 2 | | 2265690 | cenobamate |  | | 110 182 | 0.1% 0.0% | | <0.001 | 0.031 |
|  |  | 1 2 | | 31914 | tiagabine |  | | 43 57 | 0.0% 0.0% | | <0.001 | 0.022 |
|  |  | 1 2 | | 4328 | fenfluramine |  | | 32 13 | 0.0% 0.0% | | <0.001 | 0.024 |
|  |  | 1 2 | | 47858 | methsuximide |  | | 10 10 | 0.0% 0.0% | | <0.001 | 0.011 |
|  |  | 1 2 | | 2054968 | stiripentol |  | | 13 10 | 0.0% 0.0% | | <0.001 | 0.014 |
|  |  | 1 2 | | CN302 | BENZODIAZEPINE DERIVATIVE SEDATIVES/HYPNOTICS |  | | 47,925 127,279 | 54.9% 25.8% | | <0.001 | 0.622 |
|  |  | 1 2 | | CN309 | SEDATIVES/HYPNOTICS,OTHER |  | | 10,256 24,599 | 11.8% 5.0% | | <0.001 | 0.246 |
|  |  | 1 2 | | CN301 | BARBITURIC ACID DERIVATIVE SEDATIVES/HYPNOTICS |  | | 3,485 8,636 | 4.0% 1.7% | | <0.001 | 0.135 |
|  |  | 1 2 | | CN101 | OPIOID ANALGESICS |  | | 26,483 101,627 | 30.4% 20.6% | | <0.001 | 0.226 |
|  |  | 1 2 | | HS051 | GLUCOCORTICOIDS |  | | 21,867 92,455 | 25.1% 18.7% | | <0.001 | 0.154 |
|  | **Laboratory** | | | | | | | | | | | |
|  |  | Cohort | | |  | Mean ± SD | | Patients | % of Cohort | | P-Value | SMD |
|  |  | 1 2 | | 9083 | BMI | 25.7 +/- 7.1 25.3 +/- 7.5 | | 45,573 193,169 | 52.2% 39.1% | | <0.001 | 0.051 |
|  |  | 1 2 | |  | 0 - 0 kg/m2 |  | | 45,648 193,629 | 52.3% 39.2% | | <0.001 | 0.265 |
| **Cohort 1 (N = 77,099) and cohort 2 (N = 77,099) characteristics after propensity score matching** | | | | | | | | | | | | |
|  | **Demographics** | | | | | | | | | | | |
|  |  | Cohort | | |  | Mean ± SD | | Patients | % of Cohort | | P-Value | SMD |
|  |  | 1 2 | | AI | Age at Index | 25.1 +/- 12.8 26.4 +/- 13.5 | | 77,099 77,099 | 100% 100% | | <0.001 | 0.096 |
|  |  | 1 2 | | 2106-3 | White |  | | 44,920 45,371 | 58.3% 58.8% | | 0.020 | 0.012 |
|  |  | 1 2 | | 1002-5 | American Indian or Alaska Native |  | | 295 310 | 0.4% 0.4% | | 0.541 | 0.003 |
|  |  | 1 2 | | UNK | Unknown Race |  | | 10,562 10,110 | 13.7% 13.1% | | 0.001 | 0.017 |
|  |  | 1 2 | | 2076-8 | Native Hawaiian or Other Pacific Islander |  | | 314 321 | 0.4% 0.4% | | 0.781 | 0.001 |
|  |  | 1 2 | | 2054-5 | Black or African American |  | | 13,769 13,667 | 17.9% 17.7% | | 0.497 | 0.003 |
|  |  | 1 2 | | 2131-1 | Other Race |  | | 4,081 4,237 | 5.3% 5.5% | | 0.079 | 0.009 |
|  |  | 1 2 | | 2028-9 | Asian |  | | 3,158 3,083 | 4.1% 4.0% | | 0.332 | 0.005 |
|  | **Diagnosis** | | | | | | | | | | | |
|  |  | Cohort | | |  | Mean ± SD | | Patients | % of Cohort | | P-Value | SMD |
|  |  | 1 2 | | F80-F89 | Pervasive and specific developmental disorders |  | | 10,273 9,983 | 13.3% 12.9% | | 0.029 | 0.011 |
|  |  | 1 2 | | F40-F48 | Anxiety, dissociative, stress-related, somatoform and other nonpsychotic mental disorders |  | | 20,584 21,851 | 26.7% 28.3% | | <0.001 | 0.037 |
|  |  | 1 2 | | F90-F98 | Behavioral and emotional disorders with onset usually occurring in childhood and adolescence |  | | 12,373 12,693 | 16.0% 16.5% | | 0.027 | 0.011 |
|  |  | 1 2 | | F20-F29 | Schizophrenia, schizotypal, delusional, and other non-mood psychotic disorders |  | | 11,183 11,008 | 14.5% 14.3% | | 0.204 | 0.006 |
|  |  | 1 2 | | F60-F69 | Disorders of adult personality and behavior |  | | 5,387 5,268 | 7.0% 6.8% | | 0.232 | 0.006 |
|  |  | 1 2 | | F70-F79 | Intellectual Disabilities |  | | 4,086 3,922 | 5.3% 5.1% | | 0.060 | 0.010 |
|  |  | 1 2 | | F01-F09 | Mental disorders due to known physiological conditions |  | | 3,177 3,144 | 4.1% 4.1% | | 0.672 | 0.002 |
|  |  | 1 2 | | F99-F99 | Unspecified mental disorder (F99) |  | | 1,800 1,815 | 2.3% 2.4% | | 0.801 | 0.001 |
|  |  | 1 2 | | E08-E13 | Diabetes mellitus |  | | 3,745 4,036 | 4.9% 5.2% | | 0.001 | 0.017 |
|  |  | 1 2 | | Q00-Q07 | Congenital malformations of the nervous system |  | | 2,110 2,056 | 2.7% 2.7% | | 0.396 | 0.004 |
|  |  | 1 2 | | Q60-Q64 | Congenital malformations of the urinary system |  | | 426 455 | 0.6% 0.6% | | 0.327 | 0.005 |
|  |  | 1 2 | | N45 | Orchitis and epididymitis |  | | 394 419 | 0.5% 0.5% | | 0.379 | 0.004 |
|  |  | 1 2 | | N43 | Hydrocele and spermatocele |  | | 447 489 | 0.6% 0.6% | | 0.169 | 0.007 |
|  |  | 1 2 | | N50.1 | Vascular disorders of male genital organs |  | | 32 33 | 0.0% 0.0% | | 0.901 | 0.001 |
|  |  | 1 2 | | N49 | Inflammatory disorders of male genital organs, not elsewhere classified |  | | 119 130 | 0.2% 0.2% | | 0.485 | 0.004 |
|  |  | 1 2 | | I86.1 | Scrotal varices |  | | 210 209 | 0.3% 0.3% | | 0.961 | <0.001 |
|  |  | 1 2 | | Q53 | Undescended and ectopic testicle |  | | 411 388 | 0.5% 0.5% | | 0.415 | 0.004 |
|  |  | 1 2 | | Q55 | Other congenital malformations of male genital organs |  | | 276 262 | 0.4% 0.3% | | 0.545 | 0.003 |
|  |  | 1 2 | | Q54 | Hypospadias |  | | 175 173 | 0.2% 0.2% | | 0.915 | 0.001 |
|  |  | 1 2 | | C60-C63 | Malignant neoplasms of male genital organs |  | | 83 87 | 0.1% 0.1% | | 0.759 | 0.002 |
|  |  | 1 2 | | N44.8 | Other noninflammatory disorders of the testis |  | | 192 193 | 0.2% 0.3% | | 0.959 | <0.001 |
|  |  | 1 2 | | N44.0 | Torsion of testis |  | | 53 63 | 0.1% 0.1% | | 0.353 | 0.005 |
|  |  | 1 2 | | N30-N39 | Other diseases of the urinary system |  | | 3,709 3,859 | 4.8% 5.0% | | 0.077 | 0.009 |
|  |  | 1 2 | | K40 | Inguinal hernia |  | | 732 739 | 0.9% 1.0% | | 0.854 | 0.001 |
|  |  | 1 2 | | E84 | Cystic fibrosis |  | | 63 61 | 0.1% 0.1% | | 0.857 | 0.001 |
|  |  | 1 2 | | S30-S39 | Injuries to the abdomen, lower back, lumbar spine, pelvis and external genitals |  | | 4,321 4,585 | 5.6% 5.9% | | 0.004 | 0.015 |
|  |  | 1 2 | | E23.0 | Hypopituitarism |  | | 300 296 | 0.4% 0.4% | | 0.870 | 0.001 |
|  |  | 1 2 | | R56 | Convulsions, not elsewhere classified |  | | 25,485 23,400 | 33.1% 30.4% | | <0.001 | 0.058 |
|  |  | 1 2 | | E34.5 | Androgen insensitivity syndrome |  | | 10 10 | 0.0% 0.0% | | 1 | <0.001 |
|  |  | 1 2 | | Q87.1 | Congenital malformation syndromes predominantly associated with short stature |  | | 67 61 | 0.1% 0.1% | | 0.596 | 0.003 |
|  |  | 1 2 | | F31 | Bipolar disorder |  | | 23,349 25,084 | 30.3% 32.5% | | <0.001 | 0.048 |
|  |  | 1 2 | | F32 | Depressive episode |  | | 12,631 13,484 | 16.4% 17.5% | | <0.001 | 0.030 |
|  |  | 1 2 | | F39 | Unspecified mood [affective] disorder |  | | 5,476 5,523 | 7.1% 7.2% | | 0.642 | 0.002 |
|  |  | 1 2 | | F33 | Major depressive disorder, recurrent |  | | 3,879 4,107 | 5.0% 5.3% | | 0.009 | 0.013 |
|  |  | 1 2 | | F34 | Persistent mood [affective] disorders |  | | 1,769 1,872 | 2.3% 2.4% | | 0.084 | 0.009 |
|  |  | 1 2 | | F30 | Manic episode |  | | 1,664 1,555 | 2.2% 2.0% | | 0.052 | 0.010 |
|  |  | 1 2 | | F50-F59 | Behavioral syndromes associated with physiological disturbances and physical factors |  | | 2,137 2,272 | 2.8% 2.9% | | 0.039 | 0.011 |
|  |  | 1 2 | | J00-J99 | Diseases of the respiratory system |  | | 23,826 24,133 | 30.9% 31.3% | | 0.091 | 0.009 |
|  |  | 1 2 | | I00-I99 | Diseases of the circulatory system |  | | 17,995 18,962 | 23.3% 24.6% | | <0.001 | 0.029 |
|  |  | 1 2 | | K00-K95 | Diseases of the digestive system |  | | 21,279 22,026 | 27.6% 28.6% | | <0.001 | 0.022 |
|  |  | 1 2 | | Q65-Q79 | Congenital malformations and deformations of the musculoskeletal system |  | | 2,075 2,040 | 2.7% 2.6% | | 0.580 | 0.003 |
|  |  | 1 2 | | N17-N19 | Acute kidney failure and chronic kidney disease |  | | 3,446 3,686 | 4.5% 4.8% | | 0.004 | 0.015 |
|  |  | 1 2 | | A50-A64 | Infections with a predominantly sexual mode of transmission |  | | 869 950 | 1.1% 1.2% | | 0.056 | 0.010 |
|  |  | 1 2 | | E03 | Other hypothyroidism |  | | 2,037 2,120 | 2.6% 2.7% | | 0.192 | 0.007 |
|  |  | 1 2 | | E05 | Thyrotoxicosis [hyperthyroidism] |  | | 298 302 | 0.4% 0.4% | | 0.870 | 0.001 |
|  |  | 1 2 | | E83.1 | Disorders of iron metabolism |  | | 81 87 | 0.1% 0.1% | | 0.643 | 0.002 |
|  |  | 1 2 | | Q99 | Other chromosome abnormalities, not elsewhere classified |  | | 652 629 | 0.8% 0.8% | | 0.519 | 0.003 |
|  |  | 1 2 | | Q93 | Monosomies and deletions from the autosomes, not elsewhere classified |  | | 330 323 | 0.4% 0.4% | | 0.784 | 0.001 |
|  |  | 1 2 | | Q90 | Down syndrome |  | | 264 283 | 0.3% 0.4% | | 0.416 | 0.004 |
|  |  | 1 2 | | Q92 | Other trisomies and partial trisomies of the autosomes, not elsewhere classified |  | | 122 120 | 0.2% 0.2% | | 0.898 | 0.001 |
|  |  | 1 2 | | Q98.4 | Klinefelter syndrome, unspecified |  | | 63 73 | 0.1% 0.1% | | 0.391 | 0.004 |
|  |  | 1 2 | | Q98.5 | Karyotype 47, XYY |  | | 22 24 | 0.0% 0.0% | | 0.768 | 0.002 |
|  |  | 1 2 | | Q98.7 | Male with sex chromosome mosaicism |  | | 21 23 | 0.0% 0.0% | | 0.763 | 0.002 |
|  |  | 1 2 | | Q98.8 | Other specified sex chromosome abnormalities, male phenotype |  | | 21 26 | 0.0% 0.0% | | 0.466 | 0.004 |
|  |  | 1 2 | | Q98.0 | Klinefelter syndrome karyotype 47, XXY |  | | 10 15 | 0.0% 0.0% | | 0.317 | 0.005 |
|  |  | 1 2 | | Q98.1 | Klinefelter syndrome, male with more than two X chromosomes |  | | 10 10 | 0.0% 0.0% | | 1 | <0.001 |
|  |  | 1 2 | | Q98.6 | Male with structurally abnormal sex chromosome |  | | 10 10 | 0.0% 0.0% | | 1 | <0.001 |
|  |  | 1 2 | | Q98.9 | Sex chromosome abnormality, male phenotype, unspecified |  | | 10 10 | 0.0% 0.0% | | 1 | <0.001 |
|  |  | 1 2 | | D35.2 | Benign neoplasm of pituitary gland |  | | 75 69 | 0.1% 0.1% | | 0.617 | 0.003 |
|  |  | 1 2 | | D35.3 | Benign neoplasm of craniopharyngeal duct |  | | 32 28 | 0.0% 0.0% | | 0.606 | 0.003 |
|  |  | 1 2 | | E40-E46 | Malnutrition |  | | 1,649 1,768 | 2.1% 2.3% | | 0.040 | 0.010 |
|  |  | 1 2 | | G40.9 | Epilepsy, unspecified |  | | 23,343 21,998 | 30.3% 28.5% | | <0.001 | 0.038 |
|  |  | 1 2 | | G40.3 | Generalized idiopathic epilepsy and epileptic syndromes |  | | 6,553 6,124 | 8.5% 7.9% | | <0.001 | 0.020 |
|  |  | 1 2 | | G40.4 | Other generalized epilepsy and epileptic syndromes |  | | 5,027 4,727 | 6.5% 6.1% | | 0.002 | 0.016 |
|  |  | 1 2 | | G40.2 | Localization-related (focal) (partial) symptomatic epilepsy and epileptic syndromes with complex partial seizures |  | | 4,981 4,223 | 6.5% 5.5% | | <0.001 | 0.042 |
|  |  | 1 2 | | G40.8 | Other epilepsy and recurrent seizures |  | | 3,987 3,652 | 5.2% 4.7% | | <0.001 | 0.020 |
|  |  | 1 2 | | G40.1 | Localization-related (focal) (partial) symptomatic epilepsy and epileptic syndromes with simple partial seizures |  | | 3,616 3,221 | 4.7% 4.2% | | <0.001 | 0.025 |
|  |  | 1 2 | | G40.A | Absence epileptic syndrome |  | | 1,923 1,831 | 2.5% 2.4% | | 0.128 | 0.008 |
|  |  | 1 2 | | G40.0 | Localization-related (focal) (partial) idiopathic epilepsy and epileptic syndromes with seizures of localized onset |  | | 1,170 1,025 | 1.5% 1.3% | | 0.002 | 0.016 |
|  |  | 1 2 | | G40.5 | Epileptic seizures related to external causes |  | | 673 733 | 0.9% 1.0% | | 0.108 | 0.008 |
|  |  | 1 2 | | G40.B | Juvenile myoclonic epilepsy [impulsive petit mal] |  | | 533 501 | 0.7% 0.6% | | 0.318 | 0.005 |
|  |  | 1 2 | | F17 | Nicotine dependence |  | | 14,658 15,517 | 19.0% 20.1% | | <0.001 | 0.028 |
|  |  | 1 2 | | F12 | Cannabis related disorders |  | | 9,315 9,572 | 12.1% 12.4% | | 0.046 | 0.010 |
|  |  | 1 2 | | F10 | Alcohol related disorders |  | | 7,867 8,383 | 10.2% 10.9% | | <0.001 | 0.022 |
|  |  | 1 2 | | F19 | Other psychoactive substance related disorders |  | | 6,420 6,650 | 8.3% 8.6% | | 0.035 | 0.011 |
|  |  | 1 2 | | F11 | Opioid related disorders |  | | 3,856 4,104 | 5.0% 5.3% | | 0.004 | 0.015 |
|  |  | 1 2 | | F14 | Cocaine related disorders |  | | 3,220 3,319 | 4.2% 4.3% | | 0.211 | 0.006 |
|  |  | 1 2 | | F15 | Other stimulant related disorders |  | | 3,248 3,293 | 4.2% 4.3% | | 0.570 | 0.003 |
|  |  | 1 2 | | F13 | Sedative, hypnotic, or anxiolytic related disorders |  | | 1,634 1,707 | 2.1% 2.2% | | 0.202 | 0.007 |
|  |  | 1 2 | | F18 | Inhalant related disorders |  | | 1,326 1,385 | 1.7% 1.8% | | 0.253 | 0.006 |
|  |  | 1 2 | | F16 | Hallucinogen related disorders |  | | 726 761 | 0.9% 1.0% | | 0.362 | 0.005 |
|  | **Procedure** | | | | | | | | | | | |
|  |  | Cohort | | |  | Mean ± SD | | Patients | % of Cohort | | P-Value | SMD |
|  |  | 1 2 | | 1010843 | Radiation Oncology Treatment |  | | 147 186 | 0.2% 0.2% | | 0.032 | 0.011 |
|  |  | 1 2 | | 1008061 | Surgical Procedures on the Urinary System |  | | 2,014 2,046 | 2.6% 2.7% | | 0.611 | 0.003 |
|  |  | 1 2 | | 1008011 | Repair initial inguinal hernia, age 5 years or older |  | | 107 106 | 0.1% 0.1% | | 0.945 | <0.001 |
|  |  | 1 2 | | 1008470 | Surgical Procedures on the Male Genital System |  | | 708 721 | 0.9% 0.9% | | 0.730 | 0.002 |
|  | **Medication** | | | | | | | | | | | |
|  |  | Cohort | | |  | Mean ± SD | | Patients | % of Cohort | | P-Value | SMD |
|  |  | 1 2 | | 25025 | finasteride |  | | 172 189 | 0.2% 0.2% | | 0.370 | 0.005 |
|  |  | 1 2 | | CN750 | LITHIUM SALTS |  | | 3,214 3,200 | 4.2% 4.2% | | 0.858 | 0.001 |
|  |  | 1 2 | | 6135 | ketoconazole |  | | 1,072 1,111 | 1.4% 1.4% | | 0.401 | 0.004 |
|  |  | 1 2 | | 10829 | trimethoprim |  | | 4,201 4,456 | 5.4% 5.8% | | 0.005 | 0.014 |
|  |  | 1 2 | | 7454 | nitrofurantoin |  | | 329 357 | 0.4% 0.5% | | 0.284 | 0.005 |
|  |  | 1 2 | | AM200 | ERYTHROMYCINS/MACROLIDES |  | | 6,439 6,887 | 8.4% 8.9% | | <0.001 | 0.021 |
|  |  | 1 2 | | AM300 | AMINOGLYCOSIDES |  | | 3,373 3,566 | 4.4% 4.6% | | 0.018 | 0.012 |
|  |  | 1 2 | | AN000 | ANTINEOPLASTICS |  | | 1,239 1,367 | 1.6% 1.8% | | 0.011 | 0.013 |
|  |  | 1 2 | | CN709 | ANTIPSYCHOTICS,OTHER |  | | 29,282 30,386 | 38.0% 39.4% | | <0.001 | 0.029 |
|  |  | 1 2 | | CN701 | PHENOTHIAZINE/RELATED ANTIPSYCHOTICS |  | | 1,712 1,531 | 2.2% 2.0% | | 0.001 | 0.016 |
|  |  | 1 2 | | CN609 | ANTIDEPRESSANTS,OTHER |  | | 22,088 23,660 | 28.6% 30.7% | | <0.001 | 0.045 |
|  |  | 1 2 | | CN601 | TRICYCLIC ANTIDEPRESSANTS |  | | 2,436 2,634 | 3.2% 3.4% | | 0.005 | 0.014 |
|  |  | 1 2 | | CV100 | BETA BLOCKERS/RELATED |  | | 7,883 8,320 | 10.2% 10.8% | | <0.001 | 0.018 |
|  |  | 1 2 | | CV200 | CALCIUM CHANNEL BLOCKERS |  | | 3,996 4,243 | 5.2% 5.5% | | 0.005 | 0.014 |
|  |  | 1 2 | | CV800 | ACE INHIBITORS |  | | 3,712 4,010 | 4.8% 5.2% | | 0.001 | 0.018 |
|  |  | 1 2 | | CV150 | ALPHA BLOCKERS/RELATED |  | | 2,854 2,989 | 3.7% 3.9% | | 0.072 | 0.009 |
|  |  | 1 2 | | CV490 | ANTIHYPERTENSIVES,OTHER |  | | 9,831 10,421 | 12.8% 13.5% | | <0.001 | 0.023 |
|  |  | 1 2 | | HS100 | ANDROGENS/ANABOLICS |  | | 476 526 | 0.6% 0.7% | | 0.113 | 0.008 |
|  |  | 1 2 | | HS800 | PROGESTINS |  | | 207 215 | 0.3% 0.3% | | 0.697 | 0.002 |
|  |  | 1 2 | | HS300 | ESTROGENS |  | | 139 162 | 0.2% 0.2% | | 0.185 | 0.007 |
|  |  | 1 2 | | 9997 | spironolactone |  | | 532 581 | 0.7% 0.8% | | 0.140 | 0.008 |
|  |  | 1 2 | | 3014 | cyproterone |  | | 10 10 | 0.0% 0.0% | | 1 | <0.001 |
|  |  | 1 2 | | 114477 | levetiracetam |  | | 15,829 16,117 | 20.5% 20.9% | | 0.070 | 0.009 |
|  |  | 1 2 | | 28439 | lamotrigine |  | | 6,099 6,568 | 7.9% 8.5% | | <0.001 | 0.022 |
|  |  | 1 2 | | 25480 | gabapentin |  | | 6,958 7,527 | 9.0% 9.8% | | <0.001 | 0.025 |
|  |  | 1 2 | | 38404 | topiramate |  | | 3,611 3,721 | 4.7% 4.8% | | 0.188 | 0.007 |
|  |  | 1 2 | | 32624 | oxcarbazepine |  | | 4,126 4,123 | 5.4% 5.3% | | 0.973 | <0.001 |
|  |  | 1 2 | | 623400 | lacosamide |  | | 2,516 2,459 | 3.3% 3.2% | | 0.411 | 0.004 |
|  |  | 1 2 | | 8183 | phenytoin |  | | 2,554 2,647 | 3.3% 3.4% | | 0.190 | 0.007 |
|  |  | 1 2 | | 21241 | clobazam |  | | 1,742 1,691 | 2.3% 2.2% | | 0.379 | 0.004 |
|  |  | 1 2 | | 2002 | carbamazepine |  | | 2,426 2,537 | 3.1% 3.3% | | 0.109 | 0.008 |
|  |  | 1 2 | | 39998 | zonisamide |  | | 1,723 1,691 | 2.2% 2.2% | | 0.580 | 0.003 |
|  |  | 1 2 | | 72236 | fosphenytoin |  | | 1,277 1,272 | 1.7% 1.6% | | 0.920 | 0.001 |
|  |  | 1 2 | | 4135 | ethosuximide |  | | 869 826 | 1.1% 1.1% | | 0.294 | 0.005 |
|  |  | 1 2 | | 69036 | rufinamide |  | | 286 271 | 0.4% 0.4% | | 0.524 | 0.003 |
|  |  | 1 2 | | 1739745 | brivaracetam |  | | 250 247 | 0.3% 0.3% | | 0.893 | 0.001 |
|  |  | 1 2 | | 14851 | vigabatrin |  | | 258 239 | 0.3% 0.3% | | 0.393 | 0.004 |
|  |  | 1 2 | | 24812 | felbamate |  | | 189 190 | 0.2% 0.2% | | 0.959 | <0.001 |
|  |  | 1 2 | | 1356552 | perampanel |  | | 209 216 | 0.3% 0.3% | | 0.734 | 0.002 |
|  |  | 1 2 | | 2045371 | cannabidiol |  | | 178 176 | 0.2% 0.2% | | 0.915 | 0.001 |
|  |  | 1 2 | | 1482502 | eslicarbazepine |  | | 124 115 | 0.2% 0.1% | | 0.560 | 0.003 |
|  |  | 1 2 | | 8691 | primidone |  | | 118 134 | 0.2% 0.2% | | 0.313 | 0.005 |
|  |  | 1 2 | | 2265690 | cenobamate |  | | 62 69 | 0.1% 0.1% | | 0.541 | 0.003 |
|  |  | 1 2 | | 31914 | tiagabine |  | | 31 34 | 0.0% 0.0% | | 0.710 | 0.002 |
|  |  | 1 2 | | 4328 | fenfluramine |  | | 14 10 | 0.0% 0.0% | | 0.414 | 0.004 |
|  |  | 1 2 | | 47858 | methsuximide |  | | 10 10 | 0.0% 0.0% | | 1 | <0.001 |
|  |  | 1 2 | | 2054968 | stiripentol |  | | 10 10 | 0.0% 0.0% | | 1 | <0.001 |
|  |  | 1 2 | | CN302 | BENZODIAZEPINE DERIVATIVE SEDATIVES/HYPNOTICS |  | | 39,119 40,490 | 50.7% 52.5% | | <0.001 | 0.036 |
|  |  | 1 2 | | CN309 | SEDATIVES/HYPNOTICS,OTHER |  | | 7,997 8,307 | 10.4% 10.8% | | 0.010 | 0.013 |
|  |  | 1 2 | | CN301 | BARBITURIC ACID DERIVATIVE SEDATIVES/HYPNOTICS |  | | 2,369 2,404 | 3.1% 3.1% | | 0.607 | 0.003 |
|  |  | 1 2 | | CN101 | OPIOID ANALGESICS |  | | 22,172 24,003 | 28.8% 31.1% | | <0.001 | 0.052 |
|  |  | 1 2 | | HS051 | GLUCOCORTICOIDS |  | | 17,942 19,038 | 23.3% 24.7% | | <0.001 | 0.033 |
|  | **Laboratory** | | | | | | | | | | | |
|  |  | Cohort | | |  | Mean ± SD | | Patients | % of Cohort | | P-Value | SMD |
|  |  | 1 2 | | 9083 | BMI | 25.8 +/- 7.1 26.2 +/- 7.3 | | 38,911 41,086 | 50.5% 53.3% | | <0.001 | 0.047 |
|  |  | 1 2 | |  | 0 - 0 kg/m2 |  | | 38,983 41,169 | 50.6% 53.4% | | <0.001 | 0.057 |

**Abbreviations:** BMI = Body Mass Index; SD = Standard deviation; SMD = Standardised mean difference

**N.B.:** Uncorrected p-values were calculated using two-sided t-tests for continuous covariates and two-sided Z-tests for categorical covariates. These p-values reflect descriptive comparisons of baseline characteristics during propensity score matching. Correction for multiple comparisons is not appropriate in this context, as the aim is to assess covariate balance (reflected by a standardised mean difference of <0.1) rather than to test hypotheses. Exact p-values beyond three decimal places are not provided in the propensity score matching outputs generated by the TriNetX platform. Where p-values fall below this threshold, they are reported as <0.001.

# Supplementary Table 5: Propensity score matching men with epilepsy or bipolar disorder exposed (cohort 1) and unexposed (cohort 2) to valproate – 90-day outcome assessment

Propensity score matching was performed on all listed characteristics. Characteristics of the cohorts before and after matching are summarized in the table below.

| **Cohort 1 and cohort 2 patient count before and after propensity score matching** | | | | | | | | | | | | |
| --- | --- | --- | --- | --- | --- | --- | --- | --- | --- | --- | --- | --- |
|  | | | Cohort | | | Patient count before matching | | | | Patient count after matching | | |
|  | | | 1 - Men with epilepsy or bipolar disorder exposed to valproate_v9 | | | 88,702 | | | | 76,278 | | |
|  | | | 2 - Men with epilepsy or bipolar disorder not exposed to valproate_v9 | | | 513,666 | | | | 76,278 | | |
| **Propensity score density function - Before and after matching (cohort 1 - purple, cohort 2 - green)** | | | | | | | | | | | | |
|  |  | | 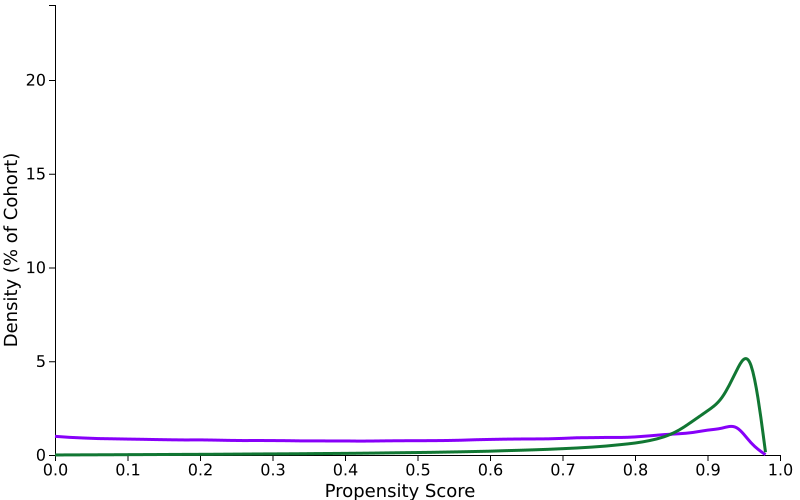 | | | | 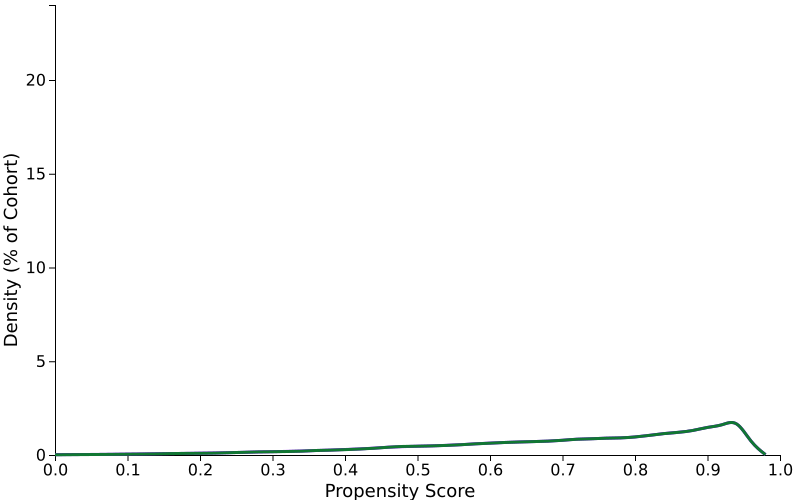 | | | | | |
| **Cohort 1 (N = 88,702) and cohort 2 (N = 513,666) characteristics before propensity score matching** | | | | | | | | | | | | |
|  | **Demographics** | | | | | | | | | | | |
|  |  | Cohort | | |  | Mean ± SD | | Patients | % of Cohort | | P-Value | SMD |
|  |  | 1 2 | | AI | Age at Index | 25.0 +/- 12.9 21.1 +/- 14.9 | | 86,195 492,716 | 100% 100% | | <0.001 | 0.279 |
|  |  | 1 2 | | 2106-3 | White |  | | 49,481 277,031 | 57.4% 56.2% | | <0.001 | 0.024 |
|  |  | 1 2 | | 1002-5 | American Indian or Alaska Native |  | | 338 1,640 | 0.4% 0.3% | | 0.006 | 0.010 |
|  |  | 1 2 | | UNK | Unknown Race |  | | 11,702 80,025 | 13.6% 16.2% | | <0.001 | 0.075 |
|  |  | 1 2 | | 2076-8 | Native Hawaiian or Other Pacific Islander |  | | 350 2,343 | 0.4% 0.5% | | 0.006 | 0.010 |
|  |  | 1 2 | | 2054-5 | Black or African American |  | | 16,157 86,445 | 18.7% 17.5% | | <0.001 | 0.031 |
|  |  | 1 2 | | 2131-1 | Other Race |  | | 4,561 29,266 | 5.3% 5.9% | | <0.001 | 0.028 |
|  |  | 1 2 | | 2028-9 | Asian |  | | 3,606 15,966 | 4.2% 3.2% | | <0.001 | 0.050 |
|  | **Diagnosis** | | | | | | | | | | | |
|  |  | Cohort | | |  | Mean ± SD | | Patients | % of Cohort | | P-Value | SMD |
|  |  | 1 2 | | F80-F89 | Pervasive and specific developmental disorders |  | | 13,694 45,129 | 15.9% 9.2% | | <0.001 | 0.204 |
|  |  | 1 2 | | F40-F48 | Anxiety, dissociative, stress-related, somatoform and other nonpsychotic mental disorders |  | | 24,104 84,955 | 28.0% 17.2% | | <0.001 | 0.258 |
|  |  | 1 2 | | F90-F98 | Behavioral and emotional disorders with onset usually occurring in childhood and adolescence |  | | 15,291 49,243 | 17.7% 10.0% | | <0.001 | 0.226 |
|  |  | 1 2 | | F20-F29 | Schizophrenia, schizotypal, delusional, and other non-mood psychotic disorders |  | | 13,796 26,196 | 16.0% 5.3% | | <0.001 | 0.352 |
|  |  | 1 2 | | F60-F69 | Disorders of adult personality and behavior |  | | 7,151 12,978 | 8.3% 2.6% | | <0.001 | 0.251 |
|  |  | 1 2 | | F70-F79 | Intellectual Disabilities |  | | 6,273 9,251 | 7.3% 1.9% | | <0.001 | 0.261 |
|  |  | 1 2 | | F01-F09 | Mental disorders due to known physiological conditions |  | | 4,479 8,069 | 5.2% 1.6% | | <0.001 | 0.197 |
|  |  | 1 2 | | F99-F99 | Unspecified mental disorder (F99) |  | | 2,550 4,760 | 3.0% 1.0% | | <0.001 | 0.144 |
|  |  | 1 2 | | E08-E13 | Diabetes mellitus |  | | 4,365 18,219 | 5.1% 3.7% | | <0.001 | 0.067 |
|  |  | 1 2 | | Q00-Q07 | Congenital malformations of the nervous system |  | | 3,106 9,624 | 3.6% 2.0% | | <0.001 | 0.101 |
|  |  | 1 2 | | Q60-Q64 | Congenital malformations of the urinary system |  | | 538 2,895 | 0.6% 0.6% | | 0.197 | 0.005 |
|  |  | 1 2 | | N45 | Orchitis and epididymitis |  | | 474 1,884 | 0.5% 0.4% | | <0.001 | 0.025 |
|  |  | 1 2 | | N43 | Hydrocele and spermatocele |  | | 556 2,736 | 0.6% 0.6% | | 0.001 | 0.012 |
|  |  | 1 2 | | N50.1 | Vascular disorders of male genital organs |  | | 43 140 | 0.0% 0.0% | | 0.001 | 0.011 |
|  |  | 1 2 | | N49 | Inflammatory disorders of male genital organs, not elsewhere classified |  | | 137 563 | 0.2% 0.1% | | <0.001 | 0.012 |
|  |  | 1 2 | | I86.1 | Scrotal varices |  | | 234 1,049 | 0.3% 0.2% | | 0.001 | 0.012 |
|  |  | 1 2 | | Q53 | Undescended and ectopic testicle |  | | 559 2,696 | 0.6% 0.5% | | <0.001 | 0.013 |
|  |  | 1 2 | | Q55 | Other congenital malformations of male genital organs |  | | 379 2,479 | 0.4% 0.5% | | 0.014 | 0.009 |
|  |  | 1 2 | | Q54 | Hypospadias |  | | 211 1,470 | 0.2% 0.3% | | 0.007 | 0.010 |
|  |  | 1 2 | | C60-C63 | Malignant neoplasms of male genital organs |  | | 92 570 | 0.1% 0.1% | | 0.473 | 0.003 |
|  |  | 1 2 | | N44.8 | Other noninflammatory disorders of the testis |  | | 249 708 | 0.3% 0.1% | | <0.001 | 0.031 |
|  |  | 1 2 | | N44.0 | Torsion of testis |  | | 60 314 | 0.1% 0.1% | | 0.531 | 0.002 |
|  |  | 1 2 | | N30-N39 | Other diseases of the urinary system |  | | 4,775 16,305 | 5.5% 3.3% | | <0.001 | 0.109 |
|  |  | 1 2 | | K40 | Inguinal hernia |  | | 882 4,868 | 1.0% 1.0% | | 0.335 | 0.004 |
|  |  | 1 2 | | E84 | Cystic fibrosis |  | | 72 337 | 0.1% 0.1% | | 0.123 | 0.005 |
|  |  | 1 2 | | S30-S39 | Injuries to the abdomen, lower back, lumbar spine, pelvis and external genitals |  | | 5,226 19,102 | 6.1% 3.9% | | <0.001 | 0.101 |
|  |  | 1 2 | | E23.0 | Hypopituitarism |  | | 373 1,467 | 0.4% 0.3% | | <0.001 | 0.022 |
|  |  | 1 2 | | R56 | Convulsions, not elsewhere classified |  | | 31,271 240,624 | 36.3% 48.8% | | <0.001 | 0.256 |
|  |  | 1 2 | | E34.5 | Androgen insensitivity syndrome |  | | 10 14 | 0.0% 0.0% | | <0.001 | 0.010 |
|  |  | 1 2 | | Q87.1 | Congenital malformation syndromes predominantly associated with short stature |  | | 88 359 | 0.1% 0.1% | | 0.004 | 0.010 |
|  |  | 1 2 | | F31 | Bipolar disorder |  | | 25,141 120,161 | 29.2% 24.4% | | <0.001 | 0.108 |
|  |  | 1 2 | | F32 | Depressive episode |  | | 14,916 49,507 | 17.3% 10.0% | | <0.001 | 0.212 |
|  |  | 1 2 | | F39 | Unspecified mood [affective] disorder |  | | 6,901 12,548 | 8.0% 2.5% | | <0.001 | 0.246 |
|  |  | 1 2 | | F33 | Major depressive disorder, recurrent |  | | 4,353 13,525 | 5.1% 2.7% | | <0.001 | 0.119 |
|  |  | 1 2 | | F34 | Persistent mood [affective] disorders |  | | 2,158 5,263 | 2.5% 1.1% | | <0.001 | 0.109 |
|  |  | 1 2 | | F30 | Manic episode |  | | 2,250 2,720 | 2.6% 0.6% | | <0.001 | 0.166 |
|  |  | 1 2 | | F50-F59 | Behavioral syndromes associated with physiological disturbances and physical factors |  | | 2,744 8,254 | 3.2% 1.7% | | <0.001 | 0.098 |
|  |  | 1 2 | | J00-J99 | Diseases of the respiratory system |  | | 28,630 145,592 | 33.2% 29.5% | | <0.001 | 0.079 |
|  |  | 1 2 | | I00-I99 | Diseases of the circulatory system |  | | 21,510 91,197 | 25.0% 18.5% | | <0.001 | 0.157 |
|  |  | 1 2 | | K00-K95 | Diseases of the digestive system |  | | 25,581 117,333 | 29.7% 23.8% | | <0.001 | 0.133 |
|  |  | 1 2 | | Q65-Q79 | Congenital malformations and deformations of the musculoskeletal system |  | | 2,771 14,433 | 3.2% 2.9% | | <0.001 | 0.017 |
|  |  | 1 2 | | N17-N19 | Acute kidney failure and chronic kidney disease |  | | 4,204 17,038 | 4.9% 3.5% | | <0.001 | 0.071 |
|  |  | 1 2 | | A50-A64 | Infections with a predominantly sexual mode of transmission |  | | 1,010 4,128 | 1.2% 0.8% | | <0.001 | 0.033 |
|  |  | 1 2 | | E03 | Other hypothyroidism |  | | 2,595 7,791 | 3.0% 1.6% | | <0.001 | 0.096 |
|  |  | 1 2 | | E05 | Thyrotoxicosis [hyperthyroidism] |  | | 366 1,071 | 0.4% 0.2% | | <0.001 | 0.037 |
|  |  | 1 2 | | E83.1 | Disorders of iron metabolism |  | | 101 548 | 0.1% 0.1% | | 0.630 | 0.002 |
|  |  | 1 2 | | Q99 | Other chromosome abnormalities, not elsewhere classified |  | | 1,008 2,759 | 1.2% 0.6% | | <0.001 | 0.066 |
|  |  | 1 2 | | Q93 | Monosomies and deletions from the autosomes, not elsewhere classified |  | | 467 1,529 | 0.5% 0.3% | | <0.001 | 0.036 |
|  |  | 1 2 | | Q90 | Down syndrome |  | | 337 1,709 | 0.4% 0.3% | | 0.044 | 0.007 |
|  |  | 1 2 | | Q92 | Other trisomies and partial trisomies of the autosomes, not elsewhere classified |  | | 190 516 | 0.2% 0.1% | | <0.001 | 0.029 |
|  |  | 1 2 | | Q98.4 | Klinefelter syndrome, unspecified |  | | 79 215 | 0.1% 0.0% | | <0.001 | 0.018 |
|  |  | 1 2 | | Q98.5 | Karyotype 47, XYY |  | | 29 89 | 0.0% 0.0% | | 0.003 | 0.010 |
|  |  | 1 2 | | Q98.7 | Male with sex chromosome mosaicism |  | | 29 57 | 0.0% 0.0% | | <0.001 | 0.015 |
|  |  | 1 2 | | Q98.8 | Other specified sex chromosome abnormalities, male phenotype |  | | 28 66 | 0.0% 0.0% | | <0.001 | 0.013 |
|  |  | 1 2 | | Q98.0 | Klinefelter syndrome karyotype 47, XXY |  | | 16 50 | 0.0% 0.0% | | 0.033 | 0.007 |
|  |  | 1 2 | | Q98.1 | Klinefelter syndrome, male with more than two X chromosomes |  | | 10 15 | 0.0% 0.0% | | <0.001 | 0.010 |
|  |  | 1 2 | | Q98.6 | Male with structurally abnormal sex chromosome |  | | 10 10 | 0.0% 0.0% | | <0.001 | 0.012 |
|  |  | 1 2 | | Q98.9 | Sex chromosome abnormality, male phenotype, unspecified |  | | 10 10 | 0.0% 0.0% | | <0.001 | 0.012 |
|  |  | 1 2 | | D35.2 | Benign neoplasm of pituitary gland |  | | 101 379 | 0.1% 0.1% | | <0.001 | 0.013 |
|  |  | 1 2 | | D35.3 | Benign neoplasm of craniopharyngeal duct |  | | 41 153 | 0.0% 0.0% | | 0.015 | 0.008 |
|  |  | 1 2 | | E40-E46 | Malnutrition |  | | 2,152 7,329 | 2.5% 1.5% | | <0.001 | 0.072 |
|  |  | 1 2 | | G40.9 | Epilepsy, unspecified |  | | 30,039 95,797 | 34.9% 19.4% | | <0.001 | 0.352 |
|  |  | 1 2 | | G40.3 | Generalized idiopathic epilepsy and epileptic syndromes |  | | 10,437 14,764 | 12.1% 3.0% | | <0.001 | 0.350 |
|  |  | 1 2 | | G40.4 | Other generalized epilepsy and epileptic syndromes |  | | 8,655 12,808 | 10.0% 2.6% | | <0.001 | 0.309 |
|  |  | 1 2 | | G40.2 | Localization-related (focal) (partial) symptomatic epilepsy and epileptic syndromes with complex partial seizures |  | | 7,841 18,826 | 9.1% 3.8% | | <0.001 | 0.216 |
|  |  | 1 2 | | G40.8 | Other epilepsy and recurrent seizures |  | | 6,331 16,925 | 7.3% 3.4% | | <0.001 | 0.174 |
|  |  | 1 2 | | G40.1 | Localization-related (focal) (partial) symptomatic epilepsy and epileptic syndromes with simple partial seizures |  | | 6,088 14,257 | 7.1% 2.9% | | <0.001 | 0.193 |
|  |  | 1 2 | | G40.A | Absence epileptic syndrome |  | | 3,121 4,931 | 3.6% 1.0% | | <0.001 | 0.175 |
|  |  | 1 2 | | G40.0 | Localization-related (focal) (partial) idiopathic epilepsy and epileptic syndromes with seizures of localized onset |  | | 2,016 4,744 | 2.3% 1.0% | | <0.001 | 0.108 |
|  |  | 1 2 | | G40.5 | Epileptic seizures related to external causes |  | | 1,218 2,562 | 1.4% 0.5% | | <0.001 | 0.091 |
|  |  | 1 2 | | G40.B | Juvenile myoclonic epilepsy [impulsive petit mal] |  | | 881 724 | 1.0% 0.1% | | <0.001 | 0.115 |
|  |  | 1 2 | | F17 | Nicotine dependence |  | | 16,592 62,797 | 19.2% 12.7% | | <0.001 | 0.178 |
|  |  | 1 2 | | F12 | Cannabis related disorders |  | | 11,049 28,270 | 12.8% 5.7% | | <0.001 | 0.246 |
|  |  | 1 2 | | F10 | Alcohol related disorders |  | | 8,774 36,521 | 10.2% 7.4% | | <0.001 | 0.098 |
|  |  | 1 2 | | F19 | Other psychoactive substance related disorders |  | | 7,553 21,763 | 8.8% 4.4% | | <0.001 | 0.176 |
|  |  | 1 2 | | F11 | Opioid related disorders |  | | 4,346 15,751 | 5.0% 3.2% | | <0.001 | 0.093 |
|  |  | 1 2 | | F14 | Cocaine related disorders |  | | 3,790 10,313 | 4.4% 2.1% | | <0.001 | 0.130 |
|  |  | 1 2 | | F15 | Other stimulant related disorders |  | | 3,648 9,728 | 4.2% 2.0% | | <0.001 | 0.130 |
|  |  | 1 2 | | F13 | Sedative, hypnotic, or anxiolytic related disorders |  | | 1,960 5,283 | 2.3% 1.1% | | <0.001 | 0.094 |
|  |  | 1 2 | | F18 | Inhalant related disorders |  | | 1,616 4,365 | 1.9% 0.9% | | <0.001 | 0.085 |
|  |  | 1 2 | | F16 | Hallucinogen related disorders |  | | 866 1,899 | 1.0% 0.4% | | <0.001 | 0.075 |
|  | **Procedure** | | | | | | | | | | | |
|  |  | Cohort | | |  | Mean ± SD | | Patients | % of Cohort | | P-Value | SMD |
|  |  | 1 2 | | 1010843 | Radiation Oncology Treatment |  | | 182 778 | 0.2% 0.2% | | <0.001 | 0.012 |
|  |  | 1 2 | | 1008061 | Surgical Procedures on the Urinary System |  | | 2,580 8,850 | 3.0% 1.8% | | <0.001 | 0.078 |
|  |  | 1 2 | | 1008011 | Repair initial inguinal hernia, age 5 years or older |  | | 126 437 | 0.1% 0.1% | | <0.001 | 0.017 |
|  |  | 1 2 | | 1008470 | Surgical Procedures on the Male Genital System |  | | 880 5,135 | 1.0% 1.0% | | 0.570 | 0.002 |
|  | **Medication** | | | | | | | | | | | |
|  |  | Cohort | | |  | Mean ± SD | | Patients | % of Cohort | | P-Value | SMD |
|  |  | 1 2 | | 25025 | finasteride |  | | 198 829 | 0.2% 0.2% | | <0.001 | 0.014 |
|  |  | 1 2 | | CN750 | LITHIUM SALTS |  | | 3,841 7,239 | 4.5% 1.5% | | <0.001 | 0.177 |
|  |  | 1 2 | | 6135 | ketoconazole |  | | 1,341 4,891 | 1.6% 1.0% | | <0.001 | 0.050 |
|  |  | 1 2 | | 10829 | trimethoprim |  | | 5,272 20,520 | 6.1% 4.2% | | <0.001 | 0.088 |
|  |  | 1 2 | | 7454 | nitrofurantoin |  | | 441 1,220 | 0.5% 0.2% | | <0.001 | 0.043 |
|  |  | 1 2 | | AM200 | ERYTHROMYCINS/MACROLIDES |  | | 8,088 31,573 | 9.4% 6.4% | | <0.001 | 0.111 |
|  |  | 1 2 | | AM300 | AMINOGLYCOSIDES |  | | 4,351 14,364 | 5.0% 2.9% | | <0.001 | 0.109 |
|  |  | 1 2 | | AN000 | ANTINEOPLASTICS |  | | 1,475 6,360 | 1.7% 1.3% | | <0.001 | 0.035 |
|  |  | 1 2 | | CN709 | ANTIPSYCHOTICS,OTHER |  | | 34,601 59,386 | 40.1% 12.1% | | <0.001 | 0.675 |
|  |  | 1 2 | | CN701 | PHENOTHIAZINE/RELATED ANTIPSYCHOTICS |  | | 2,814 2,144 | 3.3% 0.4% | | <0.001 | 0.211 |
|  |  | 1 2 | | CN609 | ANTIDEPRESSANTS,OTHER |  | | 25,945 62,277 | 30.1% 12.6% | | <0.001 | 0.436 |
|  |  | 1 2 | | CN601 | TRICYCLIC ANTIDEPRESSANTS |  | | 2,971 7,029 | 3.4% 1.4% | | <0.001 | 0.131 |
|  |  | 1 2 | | CV100 | BETA BLOCKERS/RELATED |  | | 9,614 26,703 | 11.2% 5.4% | | <0.001 | 0.209 |
|  |  | 1 2 | | CV200 | CALCIUM CHANNEL BLOCKERS |  | | 4,813 15,133 | 5.6% 3.1% | | <0.001 | 0.124 |
|  |  | 1 2 | | CV800 | ACE INHIBITORS |  | | 4,437 14,626 | 5.1% 3.0% | | <0.001 | 0.111 |
|  |  | 1 2 | | CV150 | ALPHA BLOCKERS/RELATED |  | | 3,481 7,748 | 4.0% 1.6% | | <0.001 | 0.150 |
|  |  | 1 2 | | CV490 | ANTIHYPERTENSIVES,OTHER |  | | 12,221 30,726 | 14.2% 6.2% | | <0.001 | 0.265 |
|  |  | 1 2 | | HS100 | ANDROGENS/ANABOLICS |  | | 539 2,300 | 0.6% 0.5% | | <0.001 | 0.022 |
|  |  | 1 2 | | HS800 | PROGESTINS |  | | 256 757 | 0.3% 0.2% | | <0.001 | 0.030 |
|  |  | 1 2 | | HS300 | ESTROGENS |  | | 158 665 | 0.2% 0.1% | | 0.001 | 0.012 |
|  |  | 1 2 | | 9997 | spironolactone |  | | 600 3,273 | 0.7% 0.7% | | 0.290 | 0.004 |
|  |  | 1 2 | | 3014 | cyproterone |  | | 10 10 | 0.0% 0.0% | | <0.001 | 0.012 |
|  |  | 1 2 | | 114477 | levetiracetam |  | | 20,487 60,948 | 23.8% 12.4% | | <0.001 | 0.300 |
|  |  | 1 2 | | 28439 | lamotrigine |  | | 7,736 21,889 | 9.0% 4.4% | | <0.001 | 0.182 |
|  |  | 1 2 | | 25480 | gabapentin |  | | 8,179 22,256 | 9.5% 4.5% | | <0.001 | 0.196 |
|  |  | 1 2 | | 38404 | topiramate |  | | 5,456 7,734 | 6.3% 1.6% | | <0.001 | 0.246 |
|  |  | 1 2 | | 32624 | oxcarbazepine |  | | 5,810 11,939 | 6.7% 2.4% | | <0.001 | 0.208 |
|  |  | 1 2 | | 623400 | lacosamide |  | | 4,157 4,998 | 4.8% 1.0% | | <0.001 | 0.228 |
|  |  | 1 2 | | 8183 | phenytoin |  | | 3,653 8,368 | 4.2% 1.7% | | <0.001 | 0.150 |
|  |  | 1 2 | | 21241 | clobazam |  | | 3,365 2,536 | 3.9% 0.5% | | <0.001 | 0.232 |
|  |  | 1 2 | | 2002 | carbamazepine |  | | 3,256 7,603 | 3.8% 1.5% | | <0.001 | 0.139 |
|  |  | 1 2 | | 39998 | zonisamide |  | | 2,952 3,442 | 3.4% 0.7% | | <0.001 | 0.193 |
|  |  | 1 2 | | 72236 | fosphenytoin |  | | 2,291 3,739 | 2.7% 0.8% | | <0.001 | 0.147 |
|  |  | 1 2 | | 4135 | ethosuximide |  | | 1,335 1,227 | 1.5% 0.2% | | <0.001 | 0.138 |
|  |  | 1 2 | | 69036 | rufinamide |  | | 589 361 | 0.7% 0.1% | | <0.001 | 0.099 |
|  |  | 1 2 | | 1739745 | brivaracetam |  | | 473 502 | 0.5% 0.1% | | <0.001 | 0.079 |
|  |  | 1 2 | | 14851 | vigabatrin |  | | 595 302 | 0.7% 0.1% | | <0.001 | 0.103 |
|  |  | 1 2 | | 24812 | felbamate |  | | 345 267 | 0.4% 0.1% | | <0.001 | 0.073 |
|  |  | 1 2 | | 1356552 | perampanel |  | | 419 370 | 0.5% 0.1% | | <0.001 | 0.078 |
|  |  | 1 2 | | 2045371 | cannabidiol |  | | 402 317 | 0.5% 0.1% | | <0.001 | 0.078 |
|  |  | 1 2 | | 1482502 | eslicarbazepine |  | | 204 368 | 0.2% 0.1% | | <0.001 | 0.041 |
|  |  | 1 2 | | 8691 | primidone |  | | 157 302 | 0.2% 0.1% | | <0.001 | 0.035 |
|  |  | 1 2 | | 2265690 | cenobamate |  | | 109 187 | 0.1% 0.0% | | <0.001 | 0.031 |
|  |  | 1 2 | | 31914 | tiagabine |  | | 45 57 | 0.1% 0.0% | | <0.001 | 0.023 |
|  |  | 1 2 | | 4328 | fenfluramine |  | | 30 12 | 0.0% 0.0% | | <0.001 | 0.024 |
|  |  | 1 2 | | 47858 | methsuximide |  | | 10 10 | 0.0% 0.0% | | <0.001 | 0.012 |
|  |  | 1 2 | | 2054968 | stiripentol |  | | 13 10 | 0.0% 0.0% | | <0.001 | 0.014 |
|  |  | 1 2 | | CN302 | BENZODIAZEPINE DERIVATIVE SEDATIVES/HYPNOTICS |  | | 47,568 126,920 | 55.2% 25.8% | | <0.001 | 0.628 |
|  |  | 1 2 | | CN309 | SEDATIVES/HYPNOTICS,OTHER |  | | 10,191 24,614 | 11.8% 5.0% | | <0.001 | 0.248 |
|  |  | 1 2 | | CN301 | BARBITURIC ACID DERIVATIVE SEDATIVES/HYPNOTICS |  | | 3,461 8,283 | 4.0% 1.7% | | <0.001 | 0.141 |
|  |  | 1 2 | | CN101 | OPIOID ANALGESICS |  | | 26,554 103,641 | 30.8% 21.0% | | <0.001 | 0.224 |
|  |  | 1 2 | | HS051 | GLUCOCORTICOIDS |  | | 21,764 92,947 | 25.2% 18.9% | | <0.001 | 0.154 |
|  | **Laboratory** | | | | | | | | | | | |
|  |  | Cohort | | |  | Mean ± SD | | Patients | % of Cohort | | P-Value | SMD |
|  |  | 1 2 | | 9083 | BMI | 25.8 +/- 7.1 25.5 +/- 7.5 | | 46,015 196,847 | 53.4% 40.0% | | <0.001 | 0.042 |
|  |  | 1 2 | |  | 0 - 0 kg/m2 |  | | 46,090 197,307 | 53.5% 40.0% | | <0.001 | 0.272 |
| **Cohort 1 (N = 76,278) and cohort 2 (N = 76,278) characteristics after propensity score matching** | | | | | | | | | | | | |
|  | **Demographics** | | | | | | | | | | | |
|  |  | Cohort | | |  | Mean ± SD | | Patients | % of Cohort | | P-Value | SMD |
|  |  | 1 2 | | AI | Age at Index | 25.2 +/- 12.8 26.5 +/- 13.4 | | 76,278 76,278 | 100% 100% | | <0.001 | 0.101 |
|  |  | 1 2 | | 2106-3 | White |  | | 43,703 44,542 | 57.3% 58.4% | | <0.001 | 0.022 |
|  |  | 1 2 | | 1002-5 | American Indian or Alaska Native |  | | 288 334 | 0.4% 0.4% | | 0.065 | 0.009 |
|  |  | 1 2 | | UNK | Unknown Race |  | | 10,663 9,944 | 14.0% 13.0% | | <0.001 | 0.028 |
|  |  | 1 2 | | 2076-8 | Native Hawaiian or Other Pacific Islander |  | | 315 321 | 0.4% 0.4% | | 0.812 | 0.001 |
|  |  | 1 2 | | 2054-5 | Black or African American |  | | 14,085 14,029 | 18.5% 18.4% | | 0.712 | 0.002 |
|  |  | 1 2 | | 2131-1 | Other Race |  | | 4,075 4,104 | 5.3% 5.4% | | 0.742 | 0.002 |
|  |  | 1 2 | | 2028-9 | Asian |  | | 3,149 3,004 | 4.1% 3.9% | | 0.059 | 0.010 |
|  | **Diagnosis** | | | | | | | | | | | |
|  |  | Cohort | | |  | Mean ± SD | | Patients | % of Cohort | | P-Value | SMD |
|  |  | 1 2 | | F80-F89 | Pervasive and specific developmental disorders |  | | 10,044 9,681 | 13.2% 12.7% | | 0.006 | 0.014 |
|  |  | 1 2 | | F40-F48 | Anxiety, dissociative, stress-related, somatoform and other nonpsychotic mental disorders |  | | 20,385 21,751 | 26.7% 28.5% | | <0.001 | 0.040 |
|  |  | 1 2 | | F90-F98 | Behavioral and emotional disorders with onset usually occurring in childhood and adolescence |  | | 12,139 12,559 | 15.9% 16.5% | | 0.004 | 0.015 |
|  |  | 1 2 | | F20-F29 | Schizophrenia, schizotypal, delusional, and other non-mood psychotic disorders |  | | 11,249 11,020 | 14.7% 14.4% | | 0.097 | 0.009 |
|  |  | 1 2 | | F60-F69 | Disorders of adult personality and behavior |  | | 5,230 5,156 | 6.9% 6.8% | | 0.452 | 0.004 |
|  |  | 1 2 | | F70-F79 | Intellectual Disabilities |  | | 4,029 3,851 | 5.3% 5.0% | | 0.039 | 0.011 |
|  |  | 1 2 | | F01-F09 | Mental disorders due to known physiological conditions |  | | 3,112 3,046 | 4.1% 4.0% | | 0.391 | 0.004 |
|  |  | 1 2 | | F99-F99 | Unspecified mental disorder (F99) |  | | 1,911 1,945 | 2.5% 2.5% | | 0.579 | 0.003 |
|  |  | 1 2 | | E08-E13 | Diabetes mellitus |  | | 3,781 4,065 | 5.0% 5.3% | | 0.001 | 0.017 |
|  |  | 1 2 | | Q00-Q07 | Congenital malformations of the nervous system |  | | 2,073 2,008 | 2.7% 2.6% | | 0.302 | 0.005 |
|  |  | 1 2 | | Q60-Q64 | Congenital malformations of the urinary system |  | | 414 414 | 0.5% 0.5% | | 1 | <0.001 |
|  |  | 1 2 | | N45 | Orchitis and epididymitis |  | | 397 437 | 0.5% 0.6% | | 0.165 | 0.007 |
|  |  | 1 2 | | N43 | Hydrocele and spermatocele |  | | 449 444 | 0.6% 0.6% | | 0.867 | 0.001 |
|  |  | 1 2 | | N50.1 | Vascular disorders of male genital organs |  | | 33 27 | 0.0% 0.0% | | 0.438 | 0.004 |
|  |  | 1 2 | | N49 | Inflammatory disorders of male genital organs, not elsewhere classified |  | | 114 121 | 0.1% 0.2% | | 0.648 | 0.002 |
|  |  | 1 2 | | I86.1 | Scrotal varices |  | | 204 216 | 0.3% 0.3% | | 0.558 | 0.003 |
|  |  | 1 2 | | Q53 | Undescended and ectopic testicle |  | | 396 390 | 0.5% 0.5% | | 0.830 | 0.001 |
|  |  | 1 2 | | Q55 | Other congenital malformations of male genital organs |  | | 269 278 | 0.4% 0.4% | | 0.700 | 0.002 |
|  |  | 1 2 | | Q54 | Hypospadias |  | | 168 170 | 0.2% 0.2% | | 0.913 | 0.001 |
|  |  | 1 2 | | C60-C63 | Malignant neoplasms of male genital organs |  | | 86 92 | 0.1% 0.1% | | 0.653 | 0.002 |
|  |  | 1 2 | | N44.8 | Other noninflammatory disorders of the testis |  | | 197 202 | 0.3% 0.3% | | 0.802 | 0.001 |
|  |  | 1 2 | | N44.0 | Torsion of testis |  | | 51 62 | 0.1% 0.1% | | 0.301 | 0.005 |
|  |  | 1 2 | | N30-N39 | Other diseases of the urinary system |  | | 3,728 3,861 | 4.9% 5.1% | | 0.117 | 0.008 |
|  |  | 1 2 | | K40 | Inguinal hernia |  | | 714 759 | 0.9% 1.0% | | 0.239 | 0.006 |
|  |  | 1 2 | | E84 | Cystic fibrosis |  | | 60 67 | 0.1% 0.1% | | 0.534 | 0.003 |
|  |  | 1 2 | | S30-S39 | Injuries to the abdomen, lower back, lumbar spine, pelvis and external genitals |  | | 4,343 4,673 | 5.7% 6.1% | | <0.001 | 0.018 |
|  |  | 1 2 | | E23.0 | Hypopituitarism |  | | 283 298 | 0.4% 0.4% | | 0.533 | 0.003 |
|  |  | 1 2 | | R56 | Convulsions, not elsewhere classified |  | | 25,036 22,860 | 32.8% 30.0% | | <0.001 | 0.061 |
|  |  | 1 2 | | E34.5 | Androgen insensitivity syndrome |  | | 10 10 | 0.0% 0.0% | | 1 | <0.001 |
|  |  | 1 2 | | Q87.1 | Congenital malformation syndromes predominantly associated with short stature |  | | 64 66 | 0.1% 0.1% | | 0.861 | 0.001 |
|  |  | 1 2 | | F31 | Bipolar disorder |  | | 23,103 25,171 | 30.3% 33.0% | | <0.001 | 0.058 |
|  |  | 1 2 | | F32 | Depressive episode |  | | 12,689 13,856 | 16.6% 18.2% | | <0.001 | 0.040 |
|  |  | 1 2 | | F39 | Unspecified mood [affective] disorder |  | | 5,411 5,434 | 7.1% 7.1% | | 0.819 | 0.001 |
|  |  | 1 2 | | F33 | Major depressive disorder, recurrent |  | | 3,757 4,072 | 4.9% 5.3% | | <0.001 | 0.019 |
|  |  | 1 2 | | F34 | Persistent mood [affective] disorders |  | | 1,717 1,830 | 2.3% 2.4% | | 0.055 | 0.010 |
|  |  | 1 2 | | F30 | Manic episode |  | | 1,676 1,556 | 2.2% 2.0% | | 0.033 | 0.011 |
|  |  | 1 2 | | F50-F59 | Behavioral syndromes associated with physiological disturbances and physical factors |  | | 2,148 2,240 | 2.8% 2.9% | | 0.159 | 0.007 |
|  |  | 1 2 | | J00-J99 | Diseases of the respiratory system |  | | 23,708 23,950 | 31.1% 31.4% | | 0.181 | 0.007 |
|  |  | 1 2 | | I00-I99 | Diseases of the circulatory system |  | | 17,837 18,867 | 23.4% 24.7% | | <0.001 | 0.032 |
|  |  | 1 2 | | K00-K95 | Diseases of the digestive system |  | | 21,081 21,917 | 27.6% 28.7% | | <0.001 | 0.024 |
|  |  | 1 2 | | Q65-Q79 | Congenital malformations and deformations of the musculoskeletal system |  | | 2,003 1,910 | 2.6% 2.5% | | 0.132 | 0.008 |
|  |  | 1 2 | | N17-N19 | Acute kidney failure and chronic kidney disease |  | | 3,460 3,651 | 4.5% 4.8% | | 0.020 | 0.012 |
|  |  | 1 2 | | A50-A64 | Infections with a predominantly sexual mode of transmission |  | | 876 958 | 1.1% 1.3% | | 0.054 | 0.010 |
|  |  | 1 2 | | E03 | Other hypothyroidism |  | | 2,029 2,067 | 2.7% 2.7% | | 0.547 | 0.003 |
|  |  | 1 2 | | E05 | Thyrotoxicosis [hyperthyroidism] |  | | 292 284 | 0.4% 0.4% | | 0.738 | 0.002 |
|  |  | 1 2 | | E83.1 | Disorders of iron metabolism |  | | 79 99 | 0.1% 0.1% | | 0.134 | 0.008 |
|  |  | 1 2 | | Q99 | Other chromosome abnormalities, not elsewhere classified |  | | 608 593 | 0.8% 0.8% | | 0.664 | 0.002 |
|  |  | 1 2 | | Q93 | Monosomies and deletions from the autosomes, not elsewhere classified |  | | 312 310 | 0.4% 0.4% | | 0.936 | <0.001 |
|  |  | 1 2 | | Q90 | Down syndrome |  | | 277 264 | 0.4% 0.3% | | 0.576 | 0.003 |
|  |  | 1 2 | | Q92 | Other trisomies and partial trisomies of the autosomes, not elsewhere classified |  | | 112 111 | 0.1% 0.1% | | 0.947 | <0.001 |
|  |  | 1 2 | | Q98.4 | Klinefelter syndrome, unspecified |  | | 55 61 | 0.1% 0.1% | | 0.577 | 0.003 |
|  |  | 1 2 | | Q98.5 | Karyotype 47, XYY |  | | 20 26 | 0.0% 0.0% | | 0.376 | 0.005 |
|  |  | 1 2 | | Q98.7 | Male with sex chromosome mosaicism |  | | 18 23 | 0.0% 0.0% | | 0.435 | 0.004 |
|  |  | 1 2 | | Q98.8 | Other specified sex chromosome abnormalities, male phenotype |  | | 18 26 | 0.0% 0.0% | | 0.228 | 0.006 |
|  |  | 1 2 | | Q98.0 | Klinefelter syndrome karyotype 47, XXY |  | | 11 11 | 0.0% 0.0% | | 1 | <0.001 |
|  |  | 1 2 | | Q98.1 | Klinefelter syndrome, male with more than two X chromosomes |  | | 10 10 | 0.0% 0.0% | | 1 | <0.001 |
|  |  | 1 2 | | Q98.6 | Male with structurally abnormal sex chromosome |  | | 10 0 | 0.0% 0% | | 0.002 | 0.016 |
|  |  | 1 2 | | Q98.9 | Sex chromosome abnormality, male phenotype, unspecified |  | | 0 10 | 0% 0.0% | | 0.002 | 0.016 |
|  |  | 1 2 | | D35.2 | Benign neoplasm of pituitary gland |  | | 75 74 | 0.1% 0.1% | | 0.935 | <0.001 |
|  |  | 1 2 | | D35.3 | Benign neoplasm of craniopharyngeal duct |  | | 33 25 | 0.0% 0.0% | | 0.293 | 0.005 |
|  |  | 1 2 | | E40-E46 | Malnutrition |  | | 1,644 1,697 | 2.2% 2.2% | | 0.354 | 0.005 |
|  |  | 1 2 | | G40.9 | Epilepsy, unspecified |  | | 23,003 21,632 | 30.2% 28.4% | | <0.001 | 0.040 |
|  |  | 1 2 | | G40.3 | Generalized idiopathic epilepsy and epileptic syndromes |  | | 6,420 6,000 | 8.4% 7.9% | | <0.001 | 0.020 |
|  |  | 1 2 | | G40.4 | Other generalized epilepsy and epileptic syndromes |  | | 4,991 4,665 | 6.5% 6.1% | | 0.001 | 0.018 |
|  |  | 1 2 | | G40.2 | Localization-related (focal) (partial) symptomatic epilepsy and epileptic syndromes with complex partial seizures |  | | 4,926 4,214 | 6.5% 5.5% | | <0.001 | 0.039 |
|  |  | 1 2 | | G40.8 | Other epilepsy and recurrent seizures |  | | 3,852 3,562 | 5.0% 4.7% | | 0.001 | 0.018 |
|  |  | 1 2 | | G40.1 | Localization-related (focal) (partial) symptomatic epilepsy and epileptic syndromes with simple partial seizures |  | | 3,539 3,179 | 4.6% 4.2% | | <0.001 | 0.023 |
|  |  | 1 2 | | G40.A | Absence epileptic syndrome |  | | 1,850 1,775 | 2.4% 2.3% | | 0.207 | 0.006 |
|  |  | 1 2 | | G40.0 | Localization-related (focal) (partial) idiopathic epilepsy and epileptic syndromes with seizures of localized onset |  | | 1,160 1,021 | 1.5% 1.3% | | 0.003 | 0.015 |
|  |  | 1 2 | | G40.5 | Epileptic seizures related to external causes |  | | 661 666 | 0.9% 0.9% | | 0.890 | 0.001 |
|  |  | 1 2 | | G40.B | Juvenile myoclonic epilepsy [impulsive petit mal] |  | | 533 494 | 0.7% 0.6% | | 0.222 | 0.006 |
|  |  | 1 2 | | F17 | Nicotine dependence |  | | 14,467 15,306 | 19.0% 20.1% | | <0.001 | 0.028 |
|  |  | 1 2 | | F12 | Cannabis related disorders |  | | 9,205 9,535 | 12.1% 12.5% | | 0.010 | 0.013 |
|  |  | 1 2 | | F10 | Alcohol related disorders |  | | 7,748 8,295 | 10.2% 10.9% | | <0.001 | 0.023 |
|  |  | 1 2 | | F19 | Other psychoactive substance related disorders |  | | 6,426 6,643 | 8.4% 8.7% | | 0.047 | 0.010 |
|  |  | 1 2 | | F11 | Opioid related disorders |  | | 3,802 4,031 | 5.0% 5.3% | | 0.008 | 0.014 |
|  |  | 1 2 | | F14 | Cocaine related disorders |  | | 3,211 3,412 | 4.2% 4.5% | | 0.012 | 0.013 |
|  |  | 1 2 | | F15 | Other stimulant related disorders |  | | 3,132 3,236 | 4.1% 4.2% | | 0.183 | 0.007 |
|  |  | 1 2 | | F13 | Sedative, hypnotic, or anxiolytic related disorders |  | | 1,645 1,698 | 2.2% 2.2% | | 0.354 | 0.005 |
|  |  | 1 2 | | F18 | Inhalant related disorders |  | | 1,307 1,365 | 1.7% 1.8% | | 0.258 | 0.006 |
|  |  | 1 2 | | F16 | Hallucinogen related disorders |  | | 714 716 | 0.9% 0.9% | | 0.958 | <0.001 |
|  | **Procedure** | | | | | | | | | | | |
|  |  | Cohort | | |  | Mean ± SD | | Patients | % of Cohort | | P-Value | SMD |
|  |  | 1 2 | | 1010843 | Radiation Oncology Treatment |  | | 143 157 | 0.2% 0.2% | | 0.418 | 0.004 |
|  |  | 1 2 | | 1008061 | Surgical Procedures on the Urinary System |  | | 1,988 2,097 | 2.6% 2.7% | | 0.084 | 0.009 |
|  |  | 1 2 | | 1008011 | Repair initial inguinal hernia, age 5 years or older |  | | 105 110 | 0.1% 0.1% | | 0.733 | 0.002 |
|  |  | 1 2 | | 1008470 | Surgical Procedures on the Male Genital System |  | | 692 709 | 0.9% 0.9% | | 0.648 | 0.002 |
|  | **Medication** | | | | | | | | | | | |
|  |  | Cohort | | |  | Mean ± SD | | Patients | % of Cohort | | P-Value | SMD |
|  |  | 1 2 | | 25025 | finasteride |  | | 176 209 | 0.2% 0.3% | | 0.092 | 0.009 |
|  |  | 1 2 | | CN750 | LITHIUM SALTS |  | | 3,165 3,198 | 4.1% 4.2% | | 0.673 | 0.002 |
|  |  | 1 2 | | 6135 | ketoconazole |  | | 1,075 1,126 | 1.4% 1.5% | | 0.274 | 0.006 |
|  |  | 1 2 | | 10829 | trimethoprim |  | | 4,326 4,575 | 5.7% 6.0% | | 0.007 | 0.014 |
|  |  | 1 2 | | 7454 | nitrofurantoin |  | | 337 372 | 0.4% 0.5% | | 0.188 | 0.007 |
|  |  | 1 2 | | AM200 | ERYTHROMYCINS/MACROLIDES |  | | 6,647 7,043 | 8.7% 9.2% | | <0.001 | 0.018 |
|  |  | 1 2 | | AM300 | AMINOGLYCOSIDES |  | | 3,423 3,586 | 4.5% 4.7% | | 0.046 | 0.010 |
|  |  | 1 2 | | AN000 | ANTINEOPLASTICS |  | | 1,222 1,328 | 1.6% 1.7% | | 0.034 | 0.011 |
|  |  | 1 2 | | CN709 | ANTIPSYCHOTICS,OTHER |  | | 29,155 30,295 | 38.2% 39.7% | | <0.001 | 0.031 |
|  |  | 1 2 | | CN701 | PHENOTHIAZINE/RELATED ANTIPSYCHOTICS |  | | 1,736 1,579 | 2.3% 2.1% | | 0.006 | 0.014 |
|  |  | 1 2 | | CN609 | ANTIDEPRESSANTS,OTHER |  | | 21,837 23,476 | 28.6% 30.8% | | <0.001 | 0.047 |
|  |  | 1 2 | | CN601 | TRICYCLIC ANTIDEPRESSANTS |  | | 2,437 2,669 | 3.2% 3.5% | | 0.001 | 0.017 |
|  |  | 1 2 | | CV100 | BETA BLOCKERS/RELATED |  | | 7,975 8,415 | 10.5% 11.0% | | <0.001 | 0.019 |
|  |  | 1 2 | | CV200 | CALCIUM CHANNEL BLOCKERS |  | | 4,053 4,400 | 5.3% 5.8% | | <0.001 | 0.020 |
|  |  | 1 2 | | CV800 | ACE INHIBITORS |  | | 3,754 4,152 | 4.9% 5.4% | | <0.001 | 0.024 |
|  |  | 1 2 | | CV150 | ALPHA BLOCKERS/RELATED |  | | 2,816 2,957 | 3.7% 3.9% | | 0.059 | 0.010 |
|  |  | 1 2 | | CV490 | ANTIHYPERTENSIVES,OTHER |  | | 9,836 10,360 | 12.9% 13.6% | | <0.001 | 0.020 |
|  |  | 1 2 | | HS100 | ANDROGENS/ANABOLICS |  | | 465 562 | 0.6% 0.7% | | 0.002 | 0.016 |
|  |  | 1 2 | | HS800 | PROGESTINS |  | | 206 216 | 0.3% 0.3% | | 0.626 | 0.002 |
|  |  | 1 2 | | HS300 | ESTROGENS |  | | 137 144 | 0.2% 0.2% | | 0.676 | 0.002 |
|  |  | 1 2 | | 9997 | spironolactone |  | | 529 582 | 0.7% 0.8% | | 0.111 | 0.008 |
|  |  | 1 2 | | 3014 | cyproterone |  | | 10 10 | 0.0% 0.0% | | 1 | <0.001 |
|  |  | 1 2 | | 114477 | levetiracetam |  | | 15,651 16,029 | 20.5% 21.0% | | 0.017 | 0.012 |
|  |  | 1 2 | | 28439 | lamotrigine |  | | 6,006 6,474 | 7.9% 8.5% | | <0.001 | 0.022 |
|  |  | 1 2 | | 25480 | gabapentin |  | | 6,910 7,488 | 9.1% 9.8% | | <0.001 | 0.026 |
|  |  | 1 2 | | 38404 | topiramate |  | | 3,573 3,622 | 4.7% 4.7% | | 0.554 | 0.003 |
|  |  | 1 2 | | 32624 | oxcarbazepine |  | | 4,108 4,135 | 5.4% 5.4% | | 0.760 | 0.002 |
|  |  | 1 2 | | 623400 | lacosamide |  | | 2,506 2,485 | 3.3% 3.3% | | 0.762 | 0.002 |
|  |  | 1 2 | | 8183 | phenytoin |  | | 2,636 2,848 | 3.5% 3.7% | | 0.004 | 0.015 |
|  |  | 1 2 | | 21241 | clobazam |  | | 1,767 1,689 | 2.3% 2.2% | | 0.180 | 0.007 |
|  |  | 1 2 | | 2002 | carbamazepine |  | | 2,407 2,460 | 3.2% 3.2% | | 0.440 | 0.004 |
|  |  | 1 2 | | 39998 | zonisamide |  | | 1,678 1,682 | 2.2% 2.2% | | 0.944 | <0.001 |
|  |  | 1 2 | | 72236 | fosphenytoin |  | | 1,306 1,221 | 1.7% 1.6% | | 0.088 | 0.009 |
|  |  | 1 2 | | 4135 | ethosuximide |  | | 835 771 | 1.1% 1.0% | | 0.108 | 0.008 |
|  |  | 1 2 | | 69036 | rufinamide |  | | 279 272 | 0.4% 0.4% | | 0.765 | 0.002 |
|  |  | 1 2 | | 1739745 | brivaracetam |  | | 242 251 | 0.3% 0.3% | | 0.685 | 0.002 |
|  |  | 1 2 | | 14851 | vigabatrin |  | | 255 231 | 0.3% 0.3% | | 0.276 | 0.006 |
|  |  | 1 2 | | 24812 | felbamate |  | | 185 186 | 0.2% 0.2% | | 0.959 | <0.001 |
|  |  | 1 2 | | 1356552 | perampanel |  | | 213 236 | 0.3% 0.3% | | 0.277 | 0.006 |
|  |  | 1 2 | | 2045371 | cannabidiol |  | | 183 182 | 0.2% 0.2% | | 0.958 | <0.001 |
|  |  | 1 2 | | 1482502 | eslicarbazepine |  | | 125 130 | 0.2% 0.2% | | 0.754 | 0.002 |
|  |  | 1 2 | | 8691 | primidone |  | | 121 135 | 0.2% 0.2% | | 0.381 | 0.004 |
|  |  | 1 2 | | 2265690 | cenobamate |  | | 71 60 | 0.1% 0.1% | | 0.336 | 0.005 |
|  |  | 1 2 | | 31914 | tiagabine |  | | 30 29 | 0.0% 0.0% | | 0.896 | 0.001 |
|  |  | 1 2 | | 4328 | fenfluramine |  | | 10 10 | 0.0% 0.0% | | 1 | <0.001 |
|  |  | 1 2 | | 47858 | methsuximide |  | | 10 10 | 0.0% 0.0% | | 1 | <0.001 |
|  |  | 1 2 | | 2054968 | stiripentol |  | | 10 10 | 0.0% 0.0% | | 1 | <0.001 |
|  |  | 1 2 | | CN302 | BENZODIAZEPINE DERIVATIVE SEDATIVES/HYPNOTICS |  | | 38,933 40,341 | 51.0% 52.9% | | <0.001 | 0.037 |
|  |  | 1 2 | | CN309 | SEDATIVES/HYPNOTICS,OTHER |  | | 8,028 8,297 | 10.5% 10.9% | | 0.026 | 0.011 |
|  |  | 1 2 | | CN301 | BARBITURIC ACID DERIVATIVE SEDATIVES/HYPNOTICS |  | | 2,330 2,415 | 3.1% 3.2% | | 0.210 | 0.006 |
|  |  | 1 2 | | CN101 | OPIOID ANALGESICS |  | | 22,307 24,331 | 29.2% 31.9% | | <0.001 | 0.058 |
|  |  | 1 2 | | HS051 | GLUCOCORTICOIDS |  | | 17,901 19,123 | 23.5% 25.1% | | <0.001 | 0.037 |
|  | **Laboratory** | | | | | | | | | | | |
|  |  | Cohort | | |  | Mean ± SD | | Patients | % of Cohort | | P-Value | SMD |
|  |  | 1 2 | | 9083 | BMI | 25.9 +/- 7.0 26.2 +/- 7.2 | | 39,353 41,994 | 51.6% 55.1% | | <0.001 | 0.047 |
|  |  | 1 2 | |  | 0 - 0 kg/m2 |  | | 39,423 42,069 | 51.7% 55.2% | | <0.001 | 0.070 |

**Abbreviations:** BMI = Body Mass Index; SD = Standard deviation; SMD = Standardised mean difference

**N.B.:** Uncorrected p-values were calculated using two-sided t-tests for continuous covariates and two-sided Z-tests for categorical covariates. These p-values reflect descriptive comparisons of baseline characteristics during propensity score matching. Correction for multiple comparisons is not appropriate in this context, as the aim is to assess covariate balance (reflected by a standardised mean difference of <0.1) rather than to test hypotheses. Exact p-values beyond three decimal places are not provided in the propensity score matching outputs generated by the TriNetX platform. Where p-values fall below this threshold, they are reported as <0.001.

# Supplementary Table 6: Propensity score matching men with epilepsy or bipolar disorder exposed (cohort 1) and unexposed (cohort 2) to valproate – 180-day outcome assessment

Propensity score matching was performed on all listed characteristics. Characteristics of the cohorts before and after matching are summarized in the table below.

| **Cohort 1 and cohort 2 patient count before and after propensity score matching** | | | | | | | | | | | | |
| --- | --- | --- | --- | --- | --- | --- | --- | --- | --- | --- | --- | --- |
|  | | | Cohort | | | Patient count before matching | | | | Patient count after matching | | |
|  | | | 1 - Men with epilepsy or bipolar disorder exposed to valproate_v9 | | | 88,687 | | | | 75,985 | | |
|  | | | 2 - Men with epilepsy or bipolar disorder not exposed to valproate_v9 | | | 514,702 | | | | 75,985 | | |
| **Propensity score density function - Before and after matching (cohort 1 - purple, cohort 2 - green)** | | | | | | | | | | | | |
|  |  | | 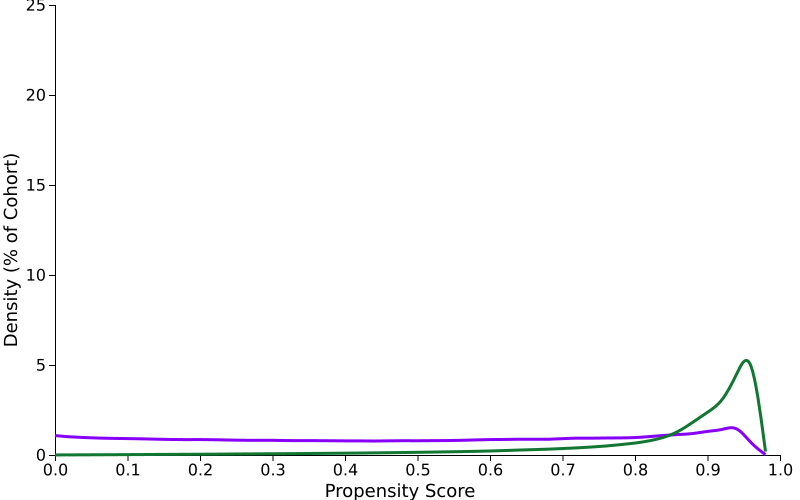 | | | | 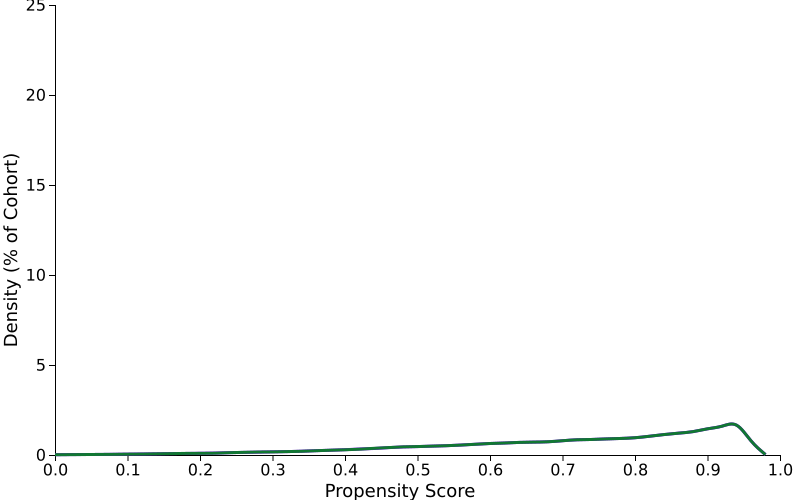 | | | | | |
| **Cohort 1 (N = 88,687) and cohort 2 (N = 514,702) characteristics before propensity score matching** | | | | | | | | | | | | |
|  | **Demographics** | | | | | | | | | | | |
|  |  | Cohort | | |  | Mean ± SD | | Patients | % of Cohort | | P-Value | SMD |
|  |  | 1 2 | | AI | Age at Index | 24.9 +/- 12.9 21.0 +/- 14.9 | | 86,184 493,843 | 100% 100% | | <0.001 | 0.276 |
|  |  | 1 2 | | 2106-3 | White |  | | 50,459 283,758 | 58.5% 57.5% | | <0.001 | 0.022 |
|  |  | 1 2 | | 1002-5 | American Indian or Alaska Native |  | | 350 1,729 | 0.4% 0.4% | | 0.011 | 0.009 |
|  |  | 1 2 | | UNK | Unknown Race |  | | 10,544 73,226 | 12.2% 14.8% | | <0.001 | 0.076 |
|  |  | 1 2 | | 2076-8 | Native Hawaiian or Other Pacific Islander |  | | 348 2,327 | 0.4% 0.5% | | 0.007 | 0.010 |
|  |  | 1 2 | | 2054-5 | Black or African American |  | | 16,099 86,288 | 18.7% 17.5% | | <0.001 | 0.031 |
|  |  | 1 2 | | 2131-1 | Other Race |  | | 4,772 30,672 | 5.5% 6.2% | | <0.001 | 0.029 |
|  |  | 1 2 | | 2028-9 | Asian |  | | 3,612 15,843 | 4.2% 3.2% | | <0.001 | 0.052 |
|  | **Diagnosis** | | | | | | | | | | | |
|  |  | Cohort | | |  | Mean ± SD | | Patients | % of Cohort | | P-Value | SMD |
|  |  | 1 2 | | F80-F89 | Pervasive and specific developmental disorders |  | | 13,913 45,913 | 16.1% 9.3% | | <0.001 | 0.207 |
|  |  | 1 2 | | F40-F48 | Anxiety, dissociative, stress-related, somatoform and other nonpsychotic mental disorders |  | | 24,076 85,315 | 27.9% 17.3% | | <0.001 | 0.257 |
|  |  | 1 2 | | F90-F98 | Behavioral and emotional disorders with onset usually occurring in childhood and adolescence |  | | 15,493 49,791 | 18.0% 10.1% | | <0.001 | 0.229 |
|  |  | 1 2 | | F20-F29 | Schizophrenia, schizotypal, delusional, and other non-mood psychotic disorders |  | | 13,686 25,734 | 15.9% 5.2% | | <0.001 | 0.353 |
|  |  | 1 2 | | F60-F69 | Disorders of adult personality and behavior |  | | 7,245 13,413 | 8.4% 2.7% | | <0.001 | 0.250 |
|  |  | 1 2 | | F70-F79 | Intellectual Disabilities |  | | 6,422 9,534 | 7.5% 1.9% | | <0.001 | 0.263 |
|  |  | 1 2 | | F01-F09 | Mental disorders due to known physiological conditions |  | | 4,586 8,375 | 5.3% 1.7% | | <0.001 | 0.198 |
|  |  | 1 2 | | F99-F99 | Unspecified mental disorder (F99) |  | | 2,529 4,840 | 2.9% 1.0% | | <0.001 | 0.141 |
|  |  | 1 2 | | E08-E13 | Diabetes mellitus |  | | 4,284 18,030 | 5.0% 3.7% | | <0.001 | 0.065 |
|  |  | 1 2 | | Q00-Q07 | Congenital malformations of the nervous system |  | | 3,177 9,812 | 3.7% 2.0% | | <0.001 | 0.103 |
|  |  | 1 2 | | Q60-Q64 | Congenital malformations of the urinary system |  | | 548 2,933 | 0.6% 0.6% | | 0.141 | 0.005 |
|  |  | 1 2 | | N45 | Orchitis and epididymitis |  | | 477 1,907 | 0.6% 0.4% | | <0.001 | 0.024 |
|  |  | 1 2 | | N43 | Hydrocele and spermatocele |  | | 563 2,781 | 0.7% 0.6% | | 0.001 | 0.012 |
|  |  | 1 2 | | N50.1 | Vascular disorders of male genital organs |  | | 43 148 | 0.0% 0.0% | | 0.003 | 0.010 |
|  |  | 1 2 | | N49 | Inflammatory disorders of male genital organs, not elsewhere classified |  | | 142 567 | 0.2% 0.1% | | <0.001 | 0.013 |
|  |  | 1 2 | | I86.1 | Scrotal varices |  | | 232 1,063 | 0.3% 0.2% | | 0.002 | 0.011 |
|  |  | 1 2 | | Q53 | Undescended and ectopic testicle |  | | 562 2,789 | 0.7% 0.6% | | 0.002 | 0.011 |
|  |  | 1 2 | | Q55 | Other congenital malformations of male genital organs |  | | 384 2,535 | 0.4% 0.5% | | 0.009 | 0.010 |
|  |  | 1 2 | | Q54 | Hypospadias |  | | 217 1,510 | 0.3% 0.3% | | 0.007 | 0.010 |
|  |  | 1 2 | | C60-C63 | Malignant neoplasms of male genital organs |  | | 94 579 | 0.1% 0.1% | | 0.515 | 0.002 |
|  |  | 1 2 | | N44.8 | Other noninflammatory disorders of the testis |  | | 249 716 | 0.3% 0.1% | | <0.001 | 0.031 |
|  |  | 1 2 | | N44.0 | Torsion of testis |  | | 57 311 | 0.1% 0.1% | | 0.734 | 0.001 |
|  |  | 1 2 | | N30-N39 | Other diseases of the urinary system |  | | 4,827 16,483 | 5.6% 3.3% | | <0.001 | 0.110 |
|  |  | 1 2 | | K40 | Inguinal hernia |  | | 886 4,959 | 1.0% 1.0% | | 0.517 | 0.002 |
|  |  | 1 2 | | E84 | Cystic fibrosis |  | | 72 339 | 0.1% 0.1% | | 0.129 | 0.005 |
|  |  | 1 2 | | S30-S39 | Injuries to the abdomen, lower back, lumbar spine, pelvis and external genitals |  | | 5,324 19,487 | 6.2% 3.9% | | <0.001 | 0.102 |
|  |  | 1 2 | | E23.0 | Hypopituitarism |  | | 385 1,496 | 0.4% 0.3% | | <0.001 | 0.024 |
|  |  | 1 2 | | R56 | Convulsions, not elsewhere classified |  | | 31,675 243,316 | 36.8% 49.3% | | <0.001 | 0.255 |
|  |  | 1 2 | | E34.5 | Androgen insensitivity syndrome |  | | 10 14 | 0.0% 0.0% | | <0.001 | 0.010 |
|  |  | 1 2 | | Q87.1 | Congenital malformation syndromes predominantly associated with short stature |  | | 92 361 | 0.1% 0.1% | | 0.001 | 0.011 |
|  |  | 1 2 | | F31 | Bipolar disorder |  | | 25,003 119,437 | 29.0% 24.2% | | <0.001 | 0.109 |
|  |  | 1 2 | | F32 | Depressive episode |  | | 14,978 50,006 | 17.4% 10.1% | | <0.001 | 0.212 |
|  |  | 1 2 | | F39 | Unspecified mood [affective] disorder |  | | 7,001 12,786 | 8.1% 2.6% | | <0.001 | 0.248 |
|  |  | 1 2 | | F33 | Major depressive disorder, recurrent |  | | 4,397 13,995 | 5.1% 2.8% | | <0.001 | 0.116 |
|  |  | 1 2 | | F34 | Persistent mood [affective] disorders |  | | 2,163 5,281 | 2.5% 1.1% | | <0.001 | 0.109 |
|  |  | 1 2 | | F30 | Manic episode |  | | 2,300 2,798 | 2.7% 0.6% | | <0.001 | 0.167 |
|  |  | 1 2 | | F50-F59 | Behavioral syndromes associated with physiological disturbances and physical factors |  | | 2,815 8,531 | 3.3% 1.7% | | <0.001 | 0.099 |
|  |  | 1 2 | | J00-J99 | Diseases of the respiratory system |  | | 28,809 146,943 | 33.4% 29.8% | | <0.001 | 0.079 |
|  |  | 1 2 | | I00-I99 | Diseases of the circulatory system |  | | 21,622 92,121 | 25.1% 18.7% | | <0.001 | 0.156 |
|  |  | 1 2 | | K00-K95 | Diseases of the digestive system |  | | 25,853 119,103 | 30.0% 24.1% | | <0.001 | 0.133 |
|  |  | 1 2 | | Q65-Q79 | Congenital malformations and deformations of the musculoskeletal system |  | | 2,858 14,918 | 3.3% 3.0% | | <0.001 | 0.017 |
|  |  | 1 2 | | N17-N19 | Acute kidney failure and chronic kidney disease |  | | 4,233 17,257 | 4.9% 3.5% | | <0.001 | 0.071 |
|  |  | 1 2 | | A50-A64 | Infections with a predominantly sexual mode of transmission |  | | 1,029 4,356 | 1.2% 0.9% | | <0.001 | 0.031 |
|  |  | 1 2 | | E03 | Other hypothyroidism |  | | 2,597 7,842 | 3.0% 1.6% | | <0.001 | 0.095 |
|  |  | 1 2 | | E05 | Thyrotoxicosis [hyperthyroidism] |  | | 364 1,094 | 0.4% 0.2% | | <0.001 | 0.035 |
|  |  | 1 2 | | E83.1 | Disorders of iron metabolism |  | | 101 568 | 0.1% 0.1% | | 0.862 | 0.001 |
|  |  | 1 2 | | Q99 | Other chromosome abnormalities, not elsewhere classified |  | | 1,060 2,836 | 1.2% 0.6% | | <0.001 | 0.069 |
|  |  | 1 2 | | Q93 | Monosomies and deletions from the autosomes, not elsewhere classified |  | | 482 1,567 | 0.6% 0.3% | | <0.001 | 0.037 |
|  |  | 1 2 | | Q90 | Down syndrome |  | | 333 1,728 | 0.4% 0.3% | | 0.097 | 0.006 |
|  |  | 1 2 | | Q92 | Other trisomies and partial trisomies of the autosomes, not elsewhere classified |  | | 195 524 | 0.2% 0.1% | | <0.001 | 0.030 |
|  |  | 1 2 | | Q98.4 | Klinefelter syndrome, unspecified |  | | 80 219 | 0.1% 0.0% | | <0.001 | 0.019 |
|  |  | 1 2 | | Q98.5 | Karyotype 47, XYY |  | | 31 93 | 0.0% 0.0% | | 0.001 | 0.010 |
|  |  | 1 2 | | Q98.7 | Male with sex chromosome mosaicism |  | | 31 58 | 0.0% 0.0% | | <0.001 | 0.016 |
|  |  | 1 2 | | Q98.8 | Other specified sex chromosome abnormalities, male phenotype |  | | 31 67 | 0.0% 0.0% | | <0.001 | 0.014 |
|  |  | 1 2 | | Q98.0 | Klinefelter syndrome karyotype 47, XXY |  | | 17 53 | 0.0% 0.0% | | 0.027 | 0.007 |
|  |  | 1 2 | | Q98.1 | Klinefelter syndrome, male with more than two X chromosomes |  | | 10 15 | 0.0% 0.0% | | <0.001 | 0.010 |
|  |  | 1 2 | | Q98.6 | Male with structurally abnormal sex chromosome |  | | 10 10 | 0.0% 0.0% | | <0.001 | 0.012 |
|  |  | 1 2 | | Q98.9 | Sex chromosome abnormality, male phenotype, unspecified |  | | 10 10 | 0.0% 0.0% | | <0.001 | 0.012 |
|  |  | 1 2 | | D35.2 | Benign neoplasm of pituitary gland |  | | 102 388 | 0.1% 0.1% | | <0.001 | 0.013 |
|  |  | 1 2 | | D35.3 | Benign neoplasm of craniopharyngeal duct |  | | 39 152 | 0.0% 0.0% | | 0.031 | 0.007 |
|  |  | 1 2 | | E40-E46 | Malnutrition |  | | 2,208 7,473 | 2.6% 1.5% | | <0.001 | 0.074 |
|  |  | 1 2 | | G40.9 | Epilepsy, unspecified |  | | 30,359 95,820 | 35.2% 19.4% | | <0.001 | 0.361 |
|  |  | 1 2 | | G40.3 | Generalized idiopathic epilepsy and epileptic syndromes |  | | 10,510 14,813 | 12.2% 3.0% | | <0.001 | 0.352 |
|  |  | 1 2 | | G40.4 | Other generalized epilepsy and epileptic syndromes |  | | 8,699 12,684 | 10.1% 2.6% | | <0.001 | 0.313 |
|  |  | 1 2 | | G40.2 | Localization-related (focal) (partial) symptomatic epilepsy and epileptic syndromes with complex partial seizures |  | | 7,932 18,900 | 9.2% 3.8% | | <0.001 | 0.219 |
|  |  | 1 2 | | G40.8 | Other epilepsy and recurrent seizures |  | | 6,508 17,150 | 7.6% 3.5% | | <0.001 | 0.179 |
|  |  | 1 2 | | G40.1 | Localization-related (focal) (partial) symptomatic epilepsy and epileptic syndromes with simple partial seizures |  | | 6,219 14,430 | 7.2% 2.9% | | <0.001 | 0.197 |
|  |  | 1 2 | | G40.A | Absence epileptic syndrome |  | | 3,177 5,043 | 3.7% 1.0% | | <0.001 | 0.176 |
|  |  | 1 2 | | G40.0 | Localization-related (focal) (partial) idiopathic epilepsy and epileptic syndromes with seizures of localized onset |  | | 2,050 4,819 | 2.4% 1.0% | | <0.001 | 0.109 |
|  |  | 1 2 | | G40.5 | Epileptic seizures related to external causes |  | | 1,230 2,536 | 1.4% 0.5% | | <0.001 | 0.093 |
|  |  | 1 2 | | G40.B | Juvenile myoclonic epilepsy [impulsive petit mal] |  | | 915 755 | 1.1% 0.2% | | <0.001 | 0.117 |
|  |  | 1 2 | | F17 | Nicotine dependence |  | | 16,451 61,658 | 19.1% 12.5% | | <0.001 | 0.182 |
|  |  | 1 2 | | F12 | Cannabis related disorders |  | | 11,042 28,228 | 12.8% 5.7% | | <0.001 | 0.247 |
|  |  | 1 2 | | F10 | Alcohol related disorders |  | | 8,838 37,075 | 10.3% 7.5% | | <0.001 | 0.097 |
|  |  | 1 2 | | F19 | Other psychoactive substance related disorders |  | | 7,572 22,083 | 8.8% 4.5% | | <0.001 | 0.174 |
|  |  | 1 2 | | F11 | Opioid related disorders |  | | 4,336 15,798 | 5.0% 3.2% | | <0.001 | 0.092 |
|  |  | 1 2 | | F14 | Cocaine related disorders |  | | 3,775 10,184 | 4.4% 2.1% | | <0.001 | 0.132 |
|  |  | 1 2 | | F15 | Other stimulant related disorders |  | | 3,677 10,014 | 4.3% 2.0% | | <0.001 | 0.128 |
|  |  | 1 2 | | F13 | Sedative, hypnotic, or anxiolytic related disorders |  | | 1,951 5,284 | 2.3% 1.1% | | <0.001 | 0.093 |
|  |  | 1 2 | | F18 | Inhalant related disorders |  | | 1,635 4,405 | 1.9% 0.9% | | <0.001 | 0.086 |
|  |  | 1 2 | | F16 | Hallucinogen related disorders |  | | 863 1,929 | 1.0% 0.4% | | <0.001 | 0.074 |
|  | **Procedure** | | | | | | | | | | | |
|  |  | Cohort | | |  | Mean ± SD | | Patients | % of Cohort | | P-Value | SMD |
|  |  | 1 2 | | 1010843 | Radiation Oncology Treatment |  | | 185 793 | 0.2% 0.2% | | <0.001 | 0.012 |
|  |  | 1 2 | | 1008061 | Surgical Procedures on the Urinary System |  | | 2,601 8,936 | 3.0% 1.8% | | <0.001 | 0.079 |
|  |  | 1 2 | | 1008011 | Repair initial inguinal hernia, age 5 years or older |  | | 127 453 | 0.1% 0.1% | | <0.001 | 0.016 |
|  |  | 1 2 | | 1008470 | Surgical Procedures on the Male Genital System |  | | 898 5,294 | 1.0% 1.1% | | 0.428 | 0.003 |
|  | **Medication** | | | | | | | | | | | |
|  |  | Cohort | | |  | Mean ± SD | | Patients | % of Cohort | | P-Value | SMD |
|  |  | 1 2 | | 25025 | finasteride |  | | 202 850 | 0.2% 0.2% | | <0.001 | 0.014 |
|  |  | 1 2 | | CN750 | LITHIUM SALTS |  | | 3,882 7,298 | 4.5% 1.5% | | <0.001 | 0.178 |
|  |  | 1 2 | | 6135 | ketoconazole |  | | 1,327 4,874 | 1.5% 1.0% | | <0.001 | 0.050 |
|  |  | 1 2 | | 10829 | trimethoprim |  | | 5,239 20,548 | 6.1% 4.2% | | <0.001 | 0.087 |
|  |  | 1 2 | | 7454 | nitrofurantoin |  | | 436 1,230 | 0.5% 0.2% | | <0.001 | 0.042 |
|  |  | 1 2 | | AM200 | ERYTHROMYCINS/MACROLIDES |  | | 8,098 31,956 | 9.4% 6.5% | | <0.001 | 0.108 |
|  |  | 1 2 | | AM300 | AMINOGLYCOSIDES |  | | 4,398 14,554 | 5.1% 2.9% | | <0.001 | 0.110 |
|  |  | 1 2 | | AN000 | ANTINEOPLASTICS |  | | 1,496 6,442 | 1.7% 1.3% | | <0.001 | 0.035 |
|  |  | 1 2 | | CN709 | ANTIPSYCHOTICS,OTHER |  | | 34,574 59,552 | 40.1% 12.1% | | <0.001 | 0.674 |
|  |  | 1 2 | | CN701 | PHENOTHIAZINE/RELATED ANTIPSYCHOTICS |  | | 2,812 2,145 | 3.3% 0.4% | | <0.001 | 0.211 |
|  |  | 1 2 | | CN609 | ANTIDEPRESSANTS,OTHER |  | | 25,991 62,540 | 30.2% 12.7% | | <0.001 | 0.437 |
|  |  | 1 2 | | CN601 | TRICYCLIC ANTIDEPRESSANTS |  | | 2,998 7,149 | 3.5% 1.4% | | <0.001 | 0.131 |
|  |  | 1 2 | | CV100 | BETA BLOCKERS/RELATED |  | | 9,714 26,962 | 11.3% 5.5% | | <0.001 | 0.211 |
|  |  | 1 2 | | CV200 | CALCIUM CHANNEL BLOCKERS |  | | 4,829 15,220 | 5.6% 3.1% | | <0.001 | 0.124 |
|  |  | 1 2 | | CV800 | ACE INHIBITORS |  | | 4,406 14,654 | 5.1% 3.0% | | <0.001 | 0.109 |
|  |  | 1 2 | | CV150 | ALPHA BLOCKERS/RELATED |  | | 3,503 7,779 | 4.1% 1.6% | | <0.001 | 0.151 |
|  |  | 1 2 | | CV490 | ANTIHYPERTENSIVES,OTHER |  | | 12,374 30,936 | 14.4% 6.3% | | <0.001 | 0.269 |
|  |  | 1 2 | | HS100 | ANDROGENS/ANABOLICS |  | | 542 2,337 | 0.6% 0.5% | | <0.001 | 0.021 |
|  |  | 1 2 | | HS800 | PROGESTINS |  | | 254 786 | 0.3% 0.2% | | <0.001 | 0.028 |
|  |  | 1 2 | | HS300 | ESTROGENS |  | | 160 726 | 0.2% 0.1% | | 0.007 | 0.009 |
|  |  | 1 2 | | 9997 | spironolactone |  | | 610 3,367 | 0.7% 0.7% | | 0.394 | 0.003 |
|  |  | 1 2 | | 3014 | cyproterone |  | | 10 10 | 0.0% 0.0% | | <0.001 | 0.012 |
|  |  | 1 2 | | 114477 | levetiracetam |  | | 20,640 61,080 | 23.9% 12.4% | | <0.001 | 0.304 |
|  |  | 1 2 | | 28439 | lamotrigine |  | | 7,787 21,799 | 9.0% 4.4% | | <0.001 | 0.185 |
|  |  | 1 2 | | 25480 | gabapentin |  | | 8,194 22,441 | 9.5% 4.5% | | <0.001 | 0.195 |
|  |  | 1 2 | | 38404 | topiramate |  | | 5,489 7,744 | 6.4% 1.6% | | <0.001 | 0.248 |
|  |  | 1 2 | | 32624 | oxcarbazepine |  | | 5,814 11,813 | 6.7% 2.4% | | <0.001 | 0.210 |
|  |  | 1 2 | | 623400 | lacosamide |  | | 4,217 4,942 | 4.9% 1.0% | | <0.001 | 0.232 |
|  |  | 1 2 | | 8183 | phenytoin |  | | 3,581 8,065 | 4.2% 1.6% | | <0.001 | 0.151 |
|  |  | 1 2 | | 21241 | clobazam |  | | 3,439 2,503 | 4.0% 0.5% | | <0.001 | 0.237 |
|  |  | 1 2 | | 2002 | carbamazepine |  | | 3,284 7,637 | 3.8% 1.5% | | <0.001 | 0.141 |
|  |  | 1 2 | | 39998 | zonisamide |  | | 2,985 3,417 | 3.5% 0.7% | | <0.001 | 0.195 |
|  |  | 1 2 | | 72236 | fosphenytoin |  | | 2,330 3,739 | 2.7% 0.8% | | <0.001 | 0.150 |
|  |  | 1 2 | | 4135 | ethosuximide |  | | 1,360 1,282 | 1.6% 0.3% | | <0.001 | 0.139 |
|  |  | 1 2 | | 69036 | rufinamide |  | | 618 343 | 0.7% 0.1% | | <0.001 | 0.104 |
|  |  | 1 2 | | 1739745 | brivaracetam |  | | 493 508 | 0.6% 0.1% | | <0.001 | 0.081 |
|  |  | 1 2 | | 14851 | vigabatrin |  | | 607 299 | 0.7% 0.1% | | <0.001 | 0.104 |
|  |  | 1 2 | | 24812 | felbamate |  | | 359 269 | 0.4% 0.1% | | <0.001 | 0.075 |
|  |  | 1 2 | | 1356552 | perampanel |  | | 414 343 | 0.5% 0.1% | | <0.001 | 0.079 |
|  |  | 1 2 | | 2045371 | cannabidiol |  | | 419 321 | 0.5% 0.1% | | <0.001 | 0.080 |
|  |  | 1 2 | | 1482502 | eslicarbazepine |  | | 191 331 | 0.2% 0.1% | | <0.001 | 0.041 |
|  |  | 1 2 | | 8691 | primidone |  | | 156 292 | 0.2% 0.1% | | <0.001 | 0.035 |
|  |  | 1 2 | | 2265690 | cenobamate |  | | 114 189 | 0.1% 0.0% | | <0.001 | 0.032 |
|  |  | 1 2 | | 31914 | tiagabine |  | | 45 58 | 0.1% 0.0% | | <0.001 | 0.023 |
|  |  | 1 2 | | 4328 | fenfluramine |  | | 32 12 | 0.0% 0.0% | | <0.001 | 0.025 |
|  |  | 1 2 | | 47858 | methsuximide |  | | 10 10 | 0.0% 0.0% | | <0.001 | 0.012 |
|  |  | 1 2 | | 2054968 | stiripentol |  | | 13 10 | 0.0% 0.0% | | <0.001 | 0.014 |
|  |  | 1 2 | | CN302 | BENZODIAZEPINE DERIVATIVE SEDATIVES/HYPNOTICS |  | | 48,008 128,693 | 55.7% 26.1% | | <0.001 | 0.632 |
|  |  | 1 2 | | CN309 | SEDATIVES/HYPNOTICS,OTHER |  | | 10,386 25,197 | 12.1% 5.1% | | <0.001 | 0.250 |
|  |  | 1 2 | | CN301 | BARBITURIC ACID DERIVATIVE SEDATIVES/HYPNOTICS |  | | 3,519 8,661 | 4.1% 1.8% | | <0.001 | 0.139 |
|  |  | 1 2 | | CN101 | OPIOID ANALGESICS |  | | 26,720 104,798 | 31.0% 21.2% | | <0.001 | 0.224 |
|  |  | 1 2 | | HS051 | GLUCOCORTICOIDS |  | | 22,035 94,682 | 25.6% 19.2% | | <0.001 | 0.154 |
|  | **Laboratory** | | | | | | | | | | | |
|  |  | Cohort | | |  | Mean ± SD | | Patients | % of Cohort | | P-Value | SMD |
|  |  | 1 2 | | 9083 | BMI | 25.8 +/- 7.1 25.4 +/- 7.5 | | 46,148 199,115 | 53.5% 40.3% | | <0.001 | 0.041 |
|  |  | 1 2 | |  | 0 - 0 kg/m2 |  | | 46,222 199,562 | 53.6% 40.4% | | <0.001 | 0.267 |
| **Cohort 1 (N = 75,985) and cohort 2 (N = 75,985) characteristics after propensity score matching** | | | | | | | | | | | | |
|  | **Demographics** | | | | | | | | | | | |
|  |  | Cohort | | |  | Mean ± SD | | Patients | % of Cohort | | P-Value | SMD |
|  |  | 1 2 | | AI | Age at Index | 25.1 +/- 12.8 26.5 +/- 13.4 | | 75,985 75,985 | 100% 100% | | <0.001 | 0.105 |
|  |  | 1 2 | | 2106-3 | White |  | | 44,398 45,474 | 58.4% 59.8% | | <0.001 | 0.029 |
|  |  | 1 2 | | 1002-5 | American Indian or Alaska Native |  | | 293 329 | 0.4% 0.4% | | 0.148 | 0.007 |
|  |  | 1 2 | | UNK | Unknown Race |  | | 9,600 8,697 | 12.6% 11.4% | | <0.001 | 0.037 |
|  |  | 1 2 | | 2076-8 | Native Hawaiian or Other Pacific Islander |  | | 316 312 | 0.4% 0.4% | | 0.873 | 0.001 |
|  |  | 1 2 | | 2054-5 | Black or African American |  | | 14,033 13,918 | 18.5% 18.3% | | 0.446 | 0.004 |
|  |  | 1 2 | | 2131-1 | Other Race |  | | 4,247 4,277 | 5.6% 5.6% | | 0.738 | 0.002 |
|  |  | 1 2 | | 2028-9 | Asian |  | | 3,098 2,978 | 4.1% 3.9% | | 0.116 | 0.008 |
|  | **Diagnosis** | | | | | | | | | | | |
|  |  | Cohort | | |  | Mean ± SD | | Patients | % of Cohort | | P-Value | SMD |
|  |  | 1 2 | | F80-F89 | Pervasive and specific developmental disorders |  | | 10,102 9,740 | 13.3% 12.8% | | 0.006 | 0.014 |
|  |  | 1 2 | | F40-F48 | Anxiety, dissociative, stress-related, somatoform and other nonpsychotic mental disorders |  | | 20,324 21,525 | 26.7% 28.3% | | <0.001 | 0.035 |
|  |  | 1 2 | | F90-F98 | Behavioral and emotional disorders with onset usually occurring in childhood and adolescence |  | | 12,197 12,492 | 16.1% 16.4% | | 0.040 | 0.011 |
|  |  | 1 2 | | F20-F29 | Schizophrenia, schizotypal, delusional, and other non-mood psychotic disorders |  | | 11,103 10,816 | 14.6% 14.2% | | 0.036 | 0.011 |
|  |  | 1 2 | | F60-F69 | Disorders of adult personality and behavior |  | | 5,321 5,244 | 7.0% 6.9% | | 0.437 | 0.004 |
|  |  | 1 2 | | F70-F79 | Intellectual Disabilities |  | | 4,084 3,817 | 5.4% 5.0% | | 0.002 | 0.016 |
|  |  | 1 2 | | F01-F09 | Mental disorders due to known physiological conditions |  | | 3,141 3,197 | 4.1% 4.2% | | 0.472 | 0.004 |
|  |  | 1 2 | | F99-F99 | Unspecified mental disorder (F99) |  | | 1,881 1,871 | 2.5% 2.5% | | 0.869 | 0.001 |
|  |  | 1 2 | | E08-E13 | Diabetes mellitus |  | | 3,672 3,963 | 4.8% 5.2% | | 0.001 | 0.018 |
|  |  | 1 2 | | Q00-Q07 | Congenital malformations of the nervous system |  | | 2,084 1,992 | 2.7% 2.6% | | 0.144 | 0.007 |
|  |  | 1 2 | | Q60-Q64 | Congenital malformations of the urinary system |  | | 412 428 | 0.5% 0.6% | | 0.580 | 0.003 |
|  |  | 1 2 | | N45 | Orchitis and epididymitis |  | | 402 427 | 0.5% 0.6% | | 0.384 | 0.004 |
|  |  | 1 2 | | N43 | Hydrocele and spermatocele |  | | 447 445 | 0.6% 0.6% | | 0.946 | <0.001 |
|  |  | 1 2 | | N50.1 | Vascular disorders of male genital organs |  | | 34 28 | 0.0% 0.0% | | 0.446 | 0.004 |
|  |  | 1 2 | | N49 | Inflammatory disorders of male genital organs, not elsewhere classified |  | | 119 130 | 0.2% 0.2% | | 0.485 | 0.004 |
|  |  | 1 2 | | I86.1 | Scrotal varices |  | | 201 209 | 0.3% 0.3% | | 0.692 | 0.002 |
|  |  | 1 2 | | Q53 | Undescended and ectopic testicle |  | | 405 367 | 0.5% 0.5% | | 0.170 | 0.007 |
|  |  | 1 2 | | Q55 | Other congenital malformations of male genital organs |  | | 279 270 | 0.4% 0.4% | | 0.700 | 0.002 |
|  |  | 1 2 | | Q54 | Hypospadias |  | | 175 158 | 0.2% 0.2% | | 0.351 | 0.005 |
|  |  | 1 2 | | C60-C63 | Malignant neoplasms of male genital organs |  | | 87 84 | 0.1% 0.1% | | 0.818 | 0.001 |
|  |  | 1 2 | | N44.8 | Other noninflammatory disorders of the testis |  | | 203 214 | 0.3% 0.3% | | 0.590 | 0.003 |
|  |  | 1 2 | | N44.0 | Torsion of testis |  | | 51 51 | 0.1% 0.1% | | 1 | <0.001 |
|  |  | 1 2 | | N30-N39 | Other diseases of the urinary system |  | | 3,741 3,903 | 4.9% 5.1% | | 0.057 | 0.010 |
|  |  | 1 2 | | K40 | Inguinal hernia |  | | 709 772 | 0.9% 1.0% | | 0.100 | 0.008 |
|  |  | 1 2 | | E84 | Cystic fibrosis |  | | 57 64 | 0.1% 0.1% | | 0.524 | 0.003 |
|  |  | 1 2 | | S30-S39 | Injuries to the abdomen, lower back, lumbar spine, pelvis and external genitals |  | | 4,416 4,624 | 5.8% 6.1% | | 0.024 | 0.012 |
|  |  | 1 2 | | E23.0 | Hypopituitarism |  | | 294 315 | 0.4% 0.4% | | 0.394 | 0.004 |
|  |  | 1 2 | | R56 | Convulsions, not elsewhere classified |  | | 25,226 22,602 | 33.2% 29.7% | | <0.001 | 0.074 |
|  |  | 1 2 | | E34.5 | Androgen insensitivity syndrome |  | | 10 10 | 0.0% 0.0% | | 1 | <0.001 |
|  |  | 1 2 | | Q87.1 | Congenital malformation syndromes predominantly associated with short stature |  | | 65 62 | 0.1% 0.1% | | 0.790 | 0.001 |
|  |  | 1 2 | | F31 | Bipolar disorder |  | | 22,943 25,475 | 30.2% 33.5% | | <0.001 | 0.072 |
|  |  | 1 2 | | F32 | Depressive episode |  | | 12,704 13,566 | 16.7% 17.9% | | <0.001 | 0.030 |
|  |  | 1 2 | | F39 | Unspecified mood [affective] disorder |  | | 5,450 5,582 | 7.2% 7.3% | | 0.192 | 0.007 |
|  |  | 1 2 | | F33 | Major depressive disorder, recurrent |  | | 3,825 4,077 | 5.0% 5.4% | | 0.004 | 0.015 |
|  |  | 1 2 | | F34 | Persistent mood [affective] disorders |  | | 1,703 1,797 | 2.2% 2.4% | | 0.108 | 0.008 |
|  |  | 1 2 | | F30 | Manic episode |  | | 1,701 1,586 | 2.2% 2.1% | | 0.043 | 0.010 |
|  |  | 1 2 | | F50-F59 | Behavioral syndromes associated with physiological disturbances and physical factors |  | | 2,194 2,298 | 2.9% 3.0% | | 0.115 | 0.008 |
|  |  | 1 2 | | J00-J99 | Diseases of the respiratory system |  | | 23,703 23,909 | 31.2% 31.5% | | 0.255 | 0.006 |
|  |  | 1 2 | | I00-I99 | Diseases of the circulatory system |  | | 17,886 18,626 | 23.5% 24.5% | | <0.001 | 0.023 |
|  |  | 1 2 | | K00-K95 | Diseases of the digestive system |  | | 21,168 21,704 | 27.9% 28.6% | | 0.002 | 0.016 |
|  |  | 1 2 | | Q65-Q79 | Congenital malformations and deformations of the musculoskeletal system |  | | 2,039 1,921 | 2.7% 2.5% | | 0.057 | 0.010 |
|  |  | 1 2 | | N17-N19 | Acute kidney failure and chronic kidney disease |  | | 3,480 3,640 | 4.6% 4.8% | | 0.052 | 0.010 |
|  |  | 1 2 | | A50-A64 | Infections with a predominantly sexual mode of transmission |  | | 906 992 | 1.2% 1.3% | | 0.047 | 0.010 |
|  |  | 1 2 | | E03 | Other hypothyroidism |  | | 2,031 2,050 | 2.7% 2.7% | | 0.763 | 0.002 |
|  |  | 1 2 | | E05 | Thyrotoxicosis [hyperthyroidism] |  | | 298 301 | 0.4% 0.4% | | 0.902 | 0.001 |
|  |  | 1 2 | | E83.1 | Disorders of iron metabolism |  | | 79 101 | 0.1% 0.1% | | 0.101 | 0.008 |
|  |  | 1 2 | | Q99 | Other chromosome abnormalities, not elsewhere classified |  | | 627 607 | 0.8% 0.8% | | 0.568 | 0.003 |
|  |  | 1 2 | | Q93 | Monosomies and deletions from the autosomes, not elsewhere classified |  | | 312 324 | 0.4% 0.4% | | 0.633 | 0.002 |
|  |  | 1 2 | | Q90 | Down syndrome |  | | 263 297 | 0.3% 0.4% | | 0.150 | 0.007 |
|  |  | 1 2 | | Q92 | Other trisomies and partial trisomies of the autosomes, not elsewhere classified |  | | 113 109 | 0.1% 0.1% | | 0.788 | 0.001 |
|  |  | 1 2 | | Q98.4 | Klinefelter syndrome, unspecified |  | | 63 64 | 0.1% 0.1% | | 0.929 | <0.001 |
|  |  | 1 2 | | Q98.5 | Karyotype 47, XYY |  | | 21 24 | 0.0% 0.0% | | 0.655 | 0.002 |
|  |  | 1 2 | | Q98.7 | Male with sex chromosome mosaicism |  | | 20 18 | 0.0% 0.0% | | 0.746 | 0.002 |
|  |  | 1 2 | | Q98.8 | Other specified sex chromosome abnormalities, male phenotype |  | | 20 22 | 0.0% 0.0% | | 0.758 | 0.002 |
|  |  | 1 2 | | Q98.0 | Klinefelter syndrome karyotype 47, XXY |  | | 14 12 | 0.0% 0.0% | | 0.695 | 0.002 |
|  |  | 1 2 | | Q98.1 | Klinefelter syndrome, male with more than two X chromosomes |  | | 10 10 | 0.0% 0.0% | | 1 | <0.001 |
|  |  | 1 2 | | Q98.6 | Male with structurally abnormal sex chromosome |  | | 10 0 | 0.0% 0% | | 0.002 | 0.016 |
|  |  | 1 2 | | Q98.9 | Sex chromosome abnormality, male phenotype, unspecified |  | | 10 0 | 0.0% 0% | | 0.002 | 0.016 |
|  |  | 1 2 | | D35.2 | Benign neoplasm of pituitary gland |  | | 76 83 | 0.1% 0.1% | | 0.579 | 0.003 |
|  |  | 1 2 | | D35.3 | Benign neoplasm of craniopharyngeal duct |  | | 32 34 | 0.0% 0.0% | | 0.805 | 0.001 |
|  |  | 1 2 | | E40-E46 | Malnutrition |  | | 1,640 1,699 | 2.2% 2.2% | | 0.302 | 0.005 |
|  |  | 1 2 | | G40.9 | Epilepsy, unspecified |  | | 23,035 21,644 | 30.3% 28.5% | | <0.001 | 0.040 |
|  |  | 1 2 | | G40.3 | Generalized idiopathic epilepsy and epileptic syndromes |  | | 6,397 6,038 | 8.4% 7.9% | | 0.001 | 0.017 |
|  |  | 1 2 | | G40.4 | Other generalized epilepsy and epileptic syndromes |  | | 4,955 4,647 | 6.5% 6.1% | | 0.001 | 0.017 |
|  |  | 1 2 | | G40.2 | Localization-related (focal) (partial) symptomatic epilepsy and epileptic syndromes with complex partial seizures |  | | 4,960 4,191 | 6.5% 5.5% | | <0.001 | 0.043 |
|  |  | 1 2 | | G40.8 | Other epilepsy and recurrent seizures |  | | 3,895 3,570 | 5.1% 4.7% | | <0.001 | 0.020 |
|  |  | 1 2 | | G40.1 | Localization-related (focal) (partial) symptomatic epilepsy and epileptic syndromes with simple partial seizures |  | | 3,591 3,198 | 4.7% 4.2% | | <0.001 | 0.025 |
|  |  | 1 2 | | G40.A | Absence epileptic syndrome |  | | 1,884 1,839 | 2.5% 2.4% | | 0.455 | 0.004 |
|  |  | 1 2 | | G40.0 | Localization-related (focal) (partial) idiopathic epilepsy and epileptic syndromes with seizures of localized onset |  | | 1,166 985 | 1.5% 1.3% | | <0.001 | 0.020 |
|  |  | 1 2 | | G40.5 | Epileptic seizures related to external causes |  | | 671 648 | 0.9% 0.9% | | 0.525 | 0.003 |
|  |  | 1 2 | | G40.B | Juvenile myoclonic epilepsy [impulsive petit mal] |  | | 560 503 | 0.7% 0.7% | | 0.079 | 0.009 |
|  |  | 1 2 | | F17 | Nicotine dependence |  | | 14,280 14,966 | 18.8% 19.7% | | <0.001 | 0.023 |
|  |  | 1 2 | | F12 | Cannabis related disorders |  | | 9,197 9,354 | 12.1% 12.3% | | 0.219 | 0.006 |
|  |  | 1 2 | | F10 | Alcohol related disorders |  | | 7,834 8,185 | 10.3% 10.8% | | 0.003 | 0.015 |
|  |  | 1 2 | | F19 | Other psychoactive substance related disorders |  | | 6,438 6,620 | 8.5% 8.7% | | 0.096 | 0.009 |
|  |  | 1 2 | | F11 | Opioid related disorders |  | | 3,777 3,961 | 5.0% 5.2% | | 0.032 | 0.011 |
|  |  | 1 2 | | F14 | Cocaine related disorders |  | | 3,182 3,343 | 4.2% 4.4% | | 0.042 | 0.010 |
|  |  | 1 2 | | F15 | Other stimulant related disorders |  | | 3,146 3,245 | 4.1% 4.3% | | 0.206 | 0.006 |
|  |  | 1 2 | | F13 | Sedative, hypnotic, or anxiolytic related disorders |  | | 1,623 1,674 | 2.1% 2.2% | | 0.369 | 0.005 |
|  |  | 1 2 | | F18 | Inhalant related disorders |  | | 1,315 1,359 | 1.7% 1.8% | | 0.391 | 0.004 |
|  |  | 1 2 | | F16 | Hallucinogen related disorders |  | | 714 742 | 0.9% 1.0% | | 0.461 | 0.004 |
|  | **Procedure** | | | | | | | | | | | |
|  |  | Cohort | | |  | Mean ± SD | | Patients | % of Cohort | | P-Value | SMD |
|  |  | 1 2 | | 1010843 | Radiation Oncology Treatment |  | | 150 175 | 0.2% 0.2% | | 0.165 | 0.007 |
|  |  | 1 2 | | 1008061 | Surgical Procedures on the Urinary System |  | | 1,985 1,999 | 2.6% 2.6% | | 0.822 | 0.001 |
|  |  | 1 2 | | 1008011 | Repair initial inguinal hernia, age 5 years or older |  | | 99 107 | 0.1% 0.1% | | 0.577 | 0.003 |
|  |  | 1 2 | | 1008470 | Surgical Procedures on the Male Genital System |  | | 709 698 | 0.9% 0.9% | | 0.768 | 0.002 |
|  | **Medication** | | | | | | | | | | | |
|  |  | Cohort | | |  | Mean ± SD | | Patients | % of Cohort | | P-Value | SMD |
|  |  | 1 2 | | 25025 | finasteride |  | | 179 198 | 0.2% 0.3% | | 0.327 | 0.005 |
|  |  | 1 2 | | CN750 | LITHIUM SALTS |  | | 3,176 3,127 | 4.2% 4.1% | | 0.528 | 0.003 |
|  |  | 1 2 | | 6135 | ketoconazole |  | | 1,050 1,073 | 1.4% 1.4% | | 0.615 | 0.003 |
|  |  | 1 2 | | 10829 | trimethoprim |  | | 4,260 4,450 | 5.6% 5.9% | | 0.036 | 0.011 |
|  |  | 1 2 | | 7454 | nitrofurantoin |  | | 335 356 | 0.4% 0.5% | | 0.423 | 0.004 |
|  |  | 1 2 | | AM200 | ERYTHROMYCINS/MACROLIDES |  | | 6,577 6,996 | 8.7% 9.2% | | <0.001 | 0.019 |
|  |  | 1 2 | | AM300 | AMINOGLYCOSIDES |  | | 3,418 3,592 | 4.5% 4.7% | | 0.033 | 0.011 |
|  |  | 1 2 | | AN000 | ANTINEOPLASTICS |  | | 1,238 1,302 | 1.6% 1.7% | | 0.200 | 0.007 |
|  |  | 1 2 | | CN709 | ANTIPSYCHOTICS,OTHER |  | | 29,038 30,186 | 38.2% 39.7% | | <0.001 | 0.031 |
|  |  | 1 2 | | CN701 | PHENOTHIAZINE/RELATED ANTIPSYCHOTICS |  | | 1,747 1,578 | 2.3% 2.1% | | 0.003 | 0.015 |
|  |  | 1 2 | | CN609 | ANTIDEPRESSANTS,OTHER |  | | 21,789 23,550 | 28.7% 31.0% | | <0.001 | 0.051 |
|  |  | 1 2 | | CN601 | TRICYCLIC ANTIDEPRESSANTS |  | | 2,440 2,671 | 3.2% 3.5% | | 0.001 | 0.017 |
|  |  | 1 2 | | CV100 | BETA BLOCKERS/RELATED |  | | 7,991 8,599 | 10.5% 11.3% | | <0.001 | 0.026 |
|  |  | 1 2 | | CV200 | CALCIUM CHANNEL BLOCKERS |  | | 4,046 4,382 | 5.3% 5.8% | | <0.001 | 0.019 |
|  |  | 1 2 | | CV800 | ACE INHIBITORS |  | | 3,691 4,018 | 4.9% 5.3% | | <0.001 | 0.020 |
|  |  | 1 2 | | CV150 | ALPHA BLOCKERS/RELATED |  | | 2,844 3,014 | 3.7% 4.0% | | 0.023 | 0.012 |
|  |  | 1 2 | | CV490 | ANTIHYPERTENSIVES,OTHER |  | | 9,865 10,493 | 13.0% 13.8% | | <0.001 | 0.024 |
|  |  | 1 2 | | HS100 | ANDROGENS/ANABOLICS |  | | 470 490 | 0.6% 0.6% | | 0.517 | 0.003 |
|  |  | 1 2 | | HS800 | PROGESTINS |  | | 208 228 | 0.3% 0.3% | | 0.337 | 0.005 |
|  |  | 1 2 | | HS300 | ESTROGENS |  | | 136 147 | 0.2% 0.2% | | 0.513 | 0.003 |
|  |  | 1 2 | | 9997 | spironolactone |  | | 527 580 | 0.7% 0.8% | | 0.110 | 0.008 |
|  |  | 1 2 | | 3014 | cyproterone |  | | 10 10 | 0.0% 0.0% | | 1 | <0.001 |
|  |  | 1 2 | | 114477 | levetiracetam |  | | 15,573 15,802 | 20.5% 20.8% | | 0.147 | 0.007 |
|  |  | 1 2 | | 28439 | lamotrigine |  | | 6,000 6,462 | 7.9% 8.5% | | <0.001 | 0.022 |
|  |  | 1 2 | | 25480 | gabapentin |  | | 6,889 7,389 | 9.1% 9.7% | | <0.001 | 0.023 |
|  |  | 1 2 | | 38404 | topiramate |  | | 3,521 3,554 | 4.6% 4.7% | | 0.688 | 0.002 |
|  |  | 1 2 | | 32624 | oxcarbazepine |  | | 4,060 4,034 | 5.3% 5.3% | | 0.766 | 0.002 |
|  |  | 1 2 | | 623400 | lacosamide |  | | 2,486 2,462 | 3.3% 3.2% | | 0.729 | 0.002 |
|  |  | 1 2 | | 8183 | phenytoin |  | | 2,539 2,631 | 3.3% 3.5% | | 0.193 | 0.007 |
|  |  | 1 2 | | 21241 | clobazam |  | | 1,771 1,711 | 2.3% 2.3% | | 0.304 | 0.005 |
|  |  | 1 2 | | 2002 | carbamazepine |  | | 2,427 2,509 | 3.2% 3.3% | | 0.235 | 0.006 |
|  |  | 1 2 | | 39998 | zonisamide |  | | 1,660 1,705 | 2.2% 2.2% | | 0.433 | 0.004 |
|  |  | 1 2 | | 72236 | fosphenytoin |  | | 1,289 1,240 | 1.7% 1.6% | | 0.326 | 0.005 |
|  |  | 1 2 | | 4135 | ethosuximide |  | | 838 824 | 1.1% 1.1% | | 0.730 | 0.002 |
|  |  | 1 2 | | 69036 | rufinamide |  | | 278 269 | 0.4% 0.4% | | 0.700 | 0.002 |
|  |  | 1 2 | | 1739745 | brivaracetam |  | | 258 256 | 0.3% 0.3% | | 0.930 | <0.001 |
|  |  | 1 2 | | 14851 | vigabatrin |  | | 257 217 | 0.3% 0.3% | | 0.066 | 0.009 |
|  |  | 1 2 | | 24812 | felbamate |  | | 195 178 | 0.3% 0.2% | | 0.378 | 0.005 |
|  |  | 1 2 | | 1356552 | perampanel |  | | 209 211 | 0.3% 0.3% | | 0.922 | 0.001 |
|  |  | 1 2 | | 2045371 | cannabidiol |  | | 193 199 | 0.3% 0.3% | | 0.762 | 0.002 |
|  |  | 1 2 | | 1482502 | eslicarbazepine |  | | 111 121 | 0.1% 0.2% | | 0.511 | 0.003 |
|  |  | 1 2 | | 8691 | primidone |  | | 115 123 | 0.2% 0.2% | | 0.604 | 0.003 |
|  |  | 1 2 | | 2265690 | cenobamate |  | | 67 62 | 0.1% 0.1% | | 0.660 | 0.002 |
|  |  | 1 2 | | 31914 | tiagabine |  | | 32 27 | 0.0% 0.0% | | 0.515 | 0.003 |
|  |  | 1 2 | | 4328 | fenfluramine |  | | 11 10 | 0.0% 0.0% | | 0.827 | 0.001 |
|  |  | 1 2 | | 47858 | methsuximide |  | | 10 10 | 0.0% 0.0% | | 1 | <0.001 |
|  |  | 1 2 | | 2054968 | stiripentol |  | | 10 10 | 0.0% 0.0% | | 1 | <0.001 |
|  |  | 1 2 | | CN302 | BENZODIAZEPINE DERIVATIVE SEDATIVES/HYPNOTICS |  | | 39,110 40,280 | 51.5% 53.0% | | <0.001 | 0.031 |
|  |  | 1 2 | | CN309 | SEDATIVES/HYPNOTICS,OTHER |  | | 8,074 8,387 | 10.6% 11.0% | | 0.010 | 0.013 |
|  |  | 1 2 | | CN301 | BARBITURIC ACID DERIVATIVE SEDATIVES/HYPNOTICS |  | | 2,360 2,351 | 3.1% 3.1% | | 0.894 | 0.001 |
|  |  | 1 2 | | CN101 | OPIOID ANALGESICS |  | | 22,285 24,094 | 29.3% 31.7% | | <0.001 | 0.052 |
|  |  | 1 2 | | HS051 | GLUCOCORTICOIDS |  | | 17,990 18,969 | 23.7% 25.0% | | <0.001 | 0.030 |
|  | **Laboratory** | | | | | | | | | | | |
|  |  | Cohort | | |  | Mean ± SD | | Patients | % of Cohort | | P-Value | SMD |
|  |  | 1 2 | | 9083 | BMI | 25.9 +/- 7.0 26.3 +/- 7.2 | | 39,301 42,396 | 51.7% 55.8% | | <0.001 | 0.055 |
|  |  | 1 2 | |  | 0 - 0 kg/m2 |  | | 39,370 42,472 | 51.8% 55.9% | | <0.001 | 0.082 |

**Abbreviations:** BMI = Body Mass Index; SD = Standard deviation; SMD = Standardised mean difference

**N.B.:** Uncorrected p-values were calculated using two-sided t-tests for continuous covariates and two-sided Z-tests for categorical covariates. These p-values reflect descriptive comparisons of baseline characteristics during propensity score matching. Correction for multiple comparisons is not appropriate in this context, as the aim is to assess covariate balance (reflected by a standardised mean difference of <0.1) rather than to test hypotheses. Exact p-values beyond three decimal places are not provided in the propensity score matching outputs generated by the TriNetX platform. Where p-values fall below this threshold, they are reported as <0.001.

# Supplementary Table 7: Propensity score matching men with epilepsy or bipolar disorder exposed (cohort 1) and unexposed (cohort 2) to valproate – 360-day outcome assessment

Propensity score matching was performed on all listed characteristics. Characteristics of the cohorts before and after matching are summarized in the table below.

| **Cohort 1 and cohort 2 patient count before and after propensity score matching** | | | | | | | | | | | | |
| --- | --- | --- | --- | --- | --- | --- | --- | --- | --- | --- | --- | --- |
|  | | | Cohort | | | Patient count before matching | | | | Patient count after matching | | |
|  | | | 1 - Men with epilepsy or bipolar disorder exposed to valproate_v9 | | | 84,068 | | | | 71,327 | | |
|  | | | 2 - Men with epilepsy or bipolar disorder not exposed to valproate_v9 | | | 496,609 | | | | 71,327 | | |
| **Propensity score density function - Before and after matching (cohort 1 - purple, cohort 2 - green)** | | | | | | | | | | | | |
|  |  | | 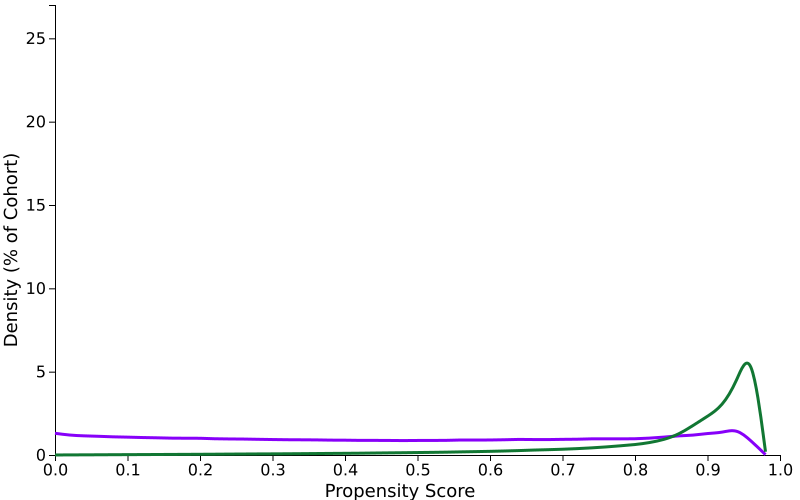 | | | | 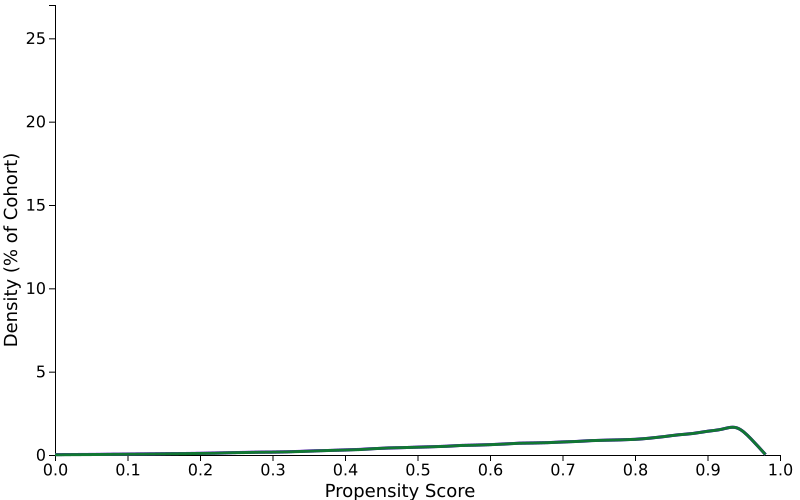 | | | | | |
| **Cohort 1 (N = 84,068) and cohort 2 (N = 496,609) characteristics before propensity score matching** | | | | | | | | | | | | |
|  | **Demographics** | | | | | | | | | | | |
|  |  | Cohort | | |  | Mean ± SD | | Patients | % of Cohort | | P-Value | SMD |
|  |  | 1 2 | | AI | Age at Index | 24.7 +/- 13.0 20.7 +/- 15.0 | | 81,563 475,681 | 100% 100% | | <0.001 | 0.283 |
|  |  | 1 2 | | 2106-3 | White |  | | 46,231 264,940 | 56.7% 55.7% | | <0.001 | 0.020 |
|  |  | 1 2 | | 1002-5 | American Indian or Alaska Native |  | | 301 1,520 | 0.4% 0.3% | | 0.022 | 0.008 |
|  |  | 1 2 | | UNK | Unknown Race |  | | 11,748 81,155 | 14.4% 17.1% | | <0.001 | 0.073 |
|  |  | 1 2 | | 2076-8 | Native Hawaiian or Other Pacific Islander |  | | 334 2,273 | 0.4% 0.5% | | 0.008 | 0.010 |
|  |  | 1 2 | | 2054-5 | Black or African American |  | | 15,455 83,414 | 18.9% 17.5% | | <0.001 | 0.037 |
|  |  | 1 2 | | 2131-1 | Other Race |  | | 3,879 26,252 | 4.8% 5.5% | | <0.001 | 0.035 |
|  |  | 1 2 | | 2028-9 | Asian |  | | 3,615 16,127 | 4.4% 3.4% | | <0.001 | 0.054 |
|  | **Diagnosis** | | | | | | | | | | | |
|  |  | Cohort | | |  | Mean ± SD | | Patients | % of Cohort | | P-Value | SMD |
|  |  | 1 2 | | F80-F89 | Pervasive and specific developmental disorders |  | | 13,288 44,334 | 16.3% 9.3% | | <0.001 | 0.210 |
|  |  | 1 2 | | F40-F48 | Anxiety, dissociative, stress-related, somatoform and other nonpsychotic mental disorders |  | | 22,405 79,841 | 27.5% 16.8% | | <0.001 | 0.260 |
|  |  | 1 2 | | F90-F98 | Behavioral and emotional disorders with onset usually occurring in childhood and adolescence |  | | 14,593 47,392 | 17.9% 10.0% | | <0.001 | 0.231 |
|  |  | 1 2 | | F20-F29 | Schizophrenia, schizotypal, delusional, and other non-mood psychotic disorders |  | | 13,088 25,135 | 16.0% 5.3% | | <0.001 | 0.354 |
|  |  | 1 2 | | F60-F69 | Disorders of adult personality and behavior |  | | 6,887 12,800 | 8.4% 2.7% | | <0.001 | 0.253 |
|  |  | 1 2 | | F70-F79 | Intellectual Disabilities |  | | 6,025 9,096 | 7.4% 1.9% | | <0.001 | 0.262 |
|  |  | 1 2 | | F01-F09 | Mental disorders due to known physiological conditions |  | | 4,260 7,814 | 5.2% 1.6% | | <0.001 | 0.198 |
|  |  | 1 2 | | F99-F99 | Unspecified mental disorder (F99) |  | | 2,360 4,532 | 2.9% 1.0% | | <0.001 | 0.142 |
|  |  | 1 2 | | E08-E13 | Diabetes mellitus |  | | 4,089 17,052 | 5.0% 3.6% | | <0.001 | 0.070 |
|  |  | 1 2 | | Q00-Q07 | Congenital malformations of the nervous system |  | | 3,094 9,474 | 3.8% 2.0% | | <0.001 | 0.108 |
|  |  | 1 2 | | Q60-Q64 | Congenital malformations of the urinary system |  | | 520 2,835 | 0.6% 0.6% | | 0.156 | 0.005 |
|  |  | 1 2 | | N45 | Orchitis and epididymitis |  | | 431 1,737 | 0.5% 0.4% | | <0.001 | 0.024 |
|  |  | 1 2 | | N43 | Hydrocele and spermatocele |  | | 535 2,649 | 0.7% 0.6% | | 0.001 | 0.013 |
|  |  | 1 2 | | N50.1 | Vascular disorders of male genital organs |  | | 36 133 | 0.0% 0.0% | | 0.014 | 0.009 |
|  |  | 1 2 | | N49 | Inflammatory disorders of male genital organs, not elsewhere classified |  | | 129 528 | 0.2% 0.1% | | <0.001 | 0.013 |
|  |  | 1 2 | | I86.1 | Scrotal varices |  | | 227 994 | 0.3% 0.2% | | <0.001 | 0.014 |
|  |  | 1 2 | | Q53 | Undescended and ectopic testicle |  | | 563 2,726 | 0.7% 0.6% | | <0.001 | 0.015 |
|  |  | 1 2 | | Q55 | Other congenital malformations of male genital organs |  | | 384 2,505 | 0.5% 0.5% | | 0.040 | 0.008 |
|  |  | 1 2 | | Q54 | Hypospadias |  | | 212 1,477 | 0.3% 0.3% | | 0.015 | 0.009 |
|  |  | 1 2 | | C60-C63 | Malignant neoplasms of male genital organs |  | | 88 510 | 0.1% 0.1% | | 0.956 | <0.001 |
|  |  | 1 2 | | N44.8 | Other noninflammatory disorders of the testis |  | | 233 656 | 0.3% 0.1% | | <0.001 | 0.032 |
|  |  | 1 2 | | N44.0 | Torsion of testis |  | | 60 299 | 0.1% 0.1% | | 0.266 | 0.004 |
|  |  | 1 2 | | N30-N39 | Other diseases of the urinary system |  | | 4,600 15,793 | 5.6% 3.3% | | <0.001 | 0.112 |
|  |  | 1 2 | | K40 | Inguinal hernia |  | | 848 4,723 | 1.0% 1.0% | | 0.215 | 0.005 |
|  |  | 1 2 | | E84 | Cystic fibrosis |  | | 68 310 | 0.1% 0.1% | | 0.065 | 0.007 |
|  |  | 1 2 | | S30-S39 | Injuries to the abdomen, lower back, lumbar spine, pelvis and external genitals |  | | 4,997 18,298 | 6.1% 3.8% | | <0.001 | 0.105 |
|  |  | 1 2 | | E23.0 | Hypopituitarism |  | | 357 1,397 | 0.4% 0.3% | | <0.001 | 0.024 |
|  |  | 1 2 | | R56 | Convulsions, not elsewhere classified |  | | 30,540 235,550 | 37.4% 49.5% | | <0.001 | 0.245 |
|  |  | 1 2 | | E34.5 | Androgen insensitivity syndrome |  | | 10 12 | 0.0% 0.0% | | <0.001 | 0.011 |
|  |  | 1 2 | | Q87.1 | Congenital malformation syndromes predominantly associated with short stature |  | | 89 352 | 0.1% 0.1% | | 0.001 | 0.012 |
|  |  | 1 2 | | F31 | Bipolar disorder |  | | 23,619 114,144 | 29.0% 24.0% | | <0.001 | 0.113 |
|  |  | 1 2 | | F32 | Depressive episode |  | | 13,811 45,843 | 16.9% 9.6% | | <0.001 | 0.216 |
|  |  | 1 2 | | F39 | Unspecified mood [affective] disorder |  | | 6,291 11,907 | 7.7% 2.5% | | <0.001 | 0.238 |
|  |  | 1 2 | | F33 | Major depressive disorder, recurrent |  | | 4,068 12,891 | 5.0% 2.7% | | <0.001 | 0.119 |
|  |  | 1 2 | | F34 | Persistent mood [affective] disorders |  | | 2,036 4,950 | 2.5% 1.0% | | <0.001 | 0.111 |
|  |  | 1 2 | | F30 | Manic episode |  | | 2,136 2,639 | 2.6% 0.6% | | <0.001 | 0.166 |
|  |  | 1 2 | | F50-F59 | Behavioral syndromes associated with physiological disturbances and physical factors |  | | 2,559 7,736 | 3.1% 1.6% | | <0.001 | 0.099 |
|  |  | 1 2 | | J00-J99 | Diseases of the respiratory system |  | | 27,348 139,677 | 33.5% 29.4% | | <0.001 | 0.090 |
|  |  | 1 2 | | I00-I99 | Diseases of the circulatory system |  | | 20,441 86,609 | 25.1% 18.2% | | <0.001 | 0.167 |
|  |  | 1 2 | | K00-K95 | Diseases of the digestive system |  | | 24,404 112,235 | 29.9% 23.6% | | <0.001 | 0.143 |
|  |  | 1 2 | | Q65-Q79 | Congenital malformations and deformations of the musculoskeletal system |  | | 2,761 14,354 | 3.4% 3.0% | | <0.001 | 0.021 |
|  |  | 1 2 | | N17-N19 | Acute kidney failure and chronic kidney disease |  | | 3,984 16,208 | 4.9% 3.4% | | <0.001 | 0.074 |
|  |  | 1 2 | | A50-A64 | Infections with a predominantly sexual mode of transmission |  | | 962 4,003 | 1.2% 0.8% | | <0.001 | 0.034 |
|  |  | 1 2 | | E03 | Other hypothyroidism |  | | 2,391 7,268 | 2.9% 1.5% | | <0.001 | 0.095 |
|  |  | 1 2 | | E05 | Thyrotoxicosis [hyperthyroidism] |  | | 342 1,041 | 0.4% 0.2% | | <0.001 | 0.036 |
|  |  | 1 2 | | E83.1 | Disorders of iron metabolism |  | | 85 490 | 0.1% 0.1% | | 0.921 | <0.001 |
|  |  | 1 2 | | Q99 | Other chromosome abnormalities, not elsewhere classified |  | | 1,019 2,751 | 1.2% 0.6% | | <0.001 | 0.071 |
|  |  | 1 2 | | Q93 | Monosomies and deletions from the autosomes, not elsewhere classified |  | | 460 1,516 | 0.6% 0.3% | | <0.001 | 0.037 |
|  |  | 1 2 | | Q90 | Down syndrome |  | | 313 1,657 | 0.4% 0.3% | | 0.115 | 0.006 |
|  |  | 1 2 | | Q92 | Other trisomies and partial trisomies of the autosomes, not elsewhere classified |  | | 187 498 | 0.2% 0.1% | | <0.001 | 0.031 |
|  |  | 1 2 | | Q98.4 | Klinefelter syndrome, unspecified |  | | 74 213 | 0.1% 0.0% | | <0.001 | 0.018 |
|  |  | 1 2 | | Q98.5 | Karyotype 47, XYY |  | | 30 91 | 0.0% 0.0% | | 0.002 | 0.011 |
|  |  | 1 2 | | Q98.7 | Male with sex chromosome mosaicism |  | | 29 58 | 0.0% 0.0% | | <0.001 | 0.015 |
|  |  | 1 2 | | Q98.8 | Other specified sex chromosome abnormalities, male phenotype |  | | 30 65 | 0.0% 0.0% | | <0.001 | 0.015 |
|  |  | 1 2 | | Q98.0 | Klinefelter syndrome karyotype 47, XXY |  | | 16 51 | 0.0% 0.0% | | 0.032 | 0.007 |
|  |  | 1 2 | | Q98.1 | Klinefelter syndrome, male with more than two X chromosomes |  | | 10 11 | 0.0% 0.0% | | <0.001 | 0.012 |
|  |  | 1 2 | | Q98.6 | Male with structurally abnormal sex chromosome |  | | 10 10 | 0.0% 0.0% | | <0.001 | 0.012 |
|  |  | 1 2 | | Q98.9 | Sex chromosome abnormality, male phenotype, unspecified |  | | 10 10 | 0.0% 0.0% | | <0.001 | 0.012 |
|  |  | 1 2 | | D35.2 | Benign neoplasm of pituitary gland |  | | 95 354 | 0.1% 0.1% | | <0.001 | 0.014 |
|  |  | 1 2 | | D35.3 | Benign neoplasm of craniopharyngeal duct |  | | 41 138 | 0.1% 0.0% | | 0.002 | 0.011 |
|  |  | 1 2 | | E40-E46 | Malnutrition |  | | 2,112 6,995 | 2.6% 1.5% | | <0.001 | 0.079 |
|  |  | 1 2 | | G40.9 | Epilepsy, unspecified |  | | 29,133 90,936 | 35.7% 19.1% | | <0.001 | 0.379 |
|  |  | 1 2 | | G40.3 | Generalized idiopathic epilepsy and epileptic syndromes |  | | 10,168 13,990 | 12.5% 2.9% | | <0.001 | 0.363 |
|  |  | 1 2 | | G40.4 | Other generalized epilepsy and epileptic syndromes |  | | 8,425 11,940 | 10.3% 2.5% | | <0.001 | 0.323 |
|  |  | 1 2 | | G40.2 | Localization-related (focal) (partial) symptomatic epilepsy and epileptic syndromes with complex partial seizures |  | | 7,675 18,135 | 9.4% 3.8% | | <0.001 | 0.227 |
|  |  | 1 2 | | G40.8 | Other epilepsy and recurrent seizures |  | | 6,245 16,223 | 7.7% 3.4% | | <0.001 | 0.187 |
|  |  | 1 2 | | G40.1 | Localization-related (focal) (partial) symptomatic epilepsy and epileptic syndromes with simple partial seizures |  | | 5,901 13,535 | 7.2% 2.8% | | <0.001 | 0.202 |
|  |  | 1 2 | | G40.A | Absence epileptic syndrome |  | | 3,104 4,849 | 3.8% 1.0% | | <0.001 | 0.182 |
|  |  | 1 2 | | G40.0 | Localization-related (focal) (partial) idiopathic epilepsy and epileptic syndromes with seizures of localized onset |  | | 1,976 4,667 | 2.4% 1.0% | | <0.001 | 0.112 |
|  |  | 1 2 | | G40.5 | Epileptic seizures related to external causes |  | | 1,138 2,367 | 1.4% 0.5% | | <0.001 | 0.093 |
|  |  | 1 2 | | G40.B | Juvenile myoclonic epilepsy [impulsive petit mal] |  | | 855 703 | 1.0% 0.1% | | <0.001 | 0.117 |
|  |  | 1 2 | | F17 | Nicotine dependence |  | | 15,341 57,790 | 18.8% 12.1% | | <0.001 | 0.185 |
|  |  | 1 2 | | F12 | Cannabis related disorders |  | | 10,473 27,100 | 12.8% 5.7% | | <0.001 | 0.248 |
|  |  | 1 2 | | F10 | Alcohol related disorders |  | | 8,391 34,884 | 10.3% 7.3% | | <0.001 | 0.104 |
|  |  | 1 2 | | F19 | Other psychoactive substance related disorders |  | | 7,300 21,317 | 9.0% 4.5% | | <0.001 | 0.179 |
|  |  | 1 2 | | F11 | Opioid related disorders |  | | 4,153 15,526 | 5.1% 3.3% | | <0.001 | 0.091 |
|  |  | 1 2 | | F14 | Cocaine related disorders |  | | 3,676 10,156 | 4.5% 2.1% | | <0.001 | 0.133 |
|  |  | 1 2 | | F15 | Other stimulant related disorders |  | | 3,406 9,229 | 4.2% 1.9% | | <0.001 | 0.130 |
|  |  | 1 2 | | F13 | Sedative, hypnotic, or anxiolytic related disorders |  | | 1,892 5,128 | 2.3% 1.1% | | <0.001 | 0.096 |
|  |  | 1 2 | | F18 | Inhalant related disorders |  | | 1,464 3,983 | 1.8% 0.8% | | <0.001 | 0.084 |
|  |  | 1 2 | | F16 | Hallucinogen related disorders |  | | 805 1,734 | 1.0% 0.4% | | <0.001 | 0.076 |
|  | **Procedure** | | | | | | | | | | | |
|  |  | Cohort | | |  | Mean ± SD | | Patients | % of Cohort | | P-Value | SMD |
|  |  | 1 2 | | 1010843 | Radiation Oncology Treatment |  | | 168 659 | 0.2% 0.1% | | <0.001 | 0.016 |
|  |  | 1 2 | | 1008061 | Surgical Procedures on the Urinary System |  | | 2,443 8,437 | 3.0% 1.8% | | <0.001 | 0.080 |
|  |  | 1 2 | | 1008011 | Repair initial inguinal hernia, age 5 years or older |  | | 111 406 | 0.1% 0.1% | | <0.001 | 0.015 |
|  |  | 1 2 | | 1008470 | Surgical Procedures on the Male Genital System |  | | 853 4,896 | 1.0% 1.0% | | 0.665 | 0.002 |
|  | **Medication** | | | | | | | | | | | |
|  |  | Cohort | | |  | Mean ± SD | | Patients | % of Cohort | | P-Value | SMD |
|  |  | 1 2 | | 25025 | finasteride |  | | 168 705 | 0.2% 0.1% | | <0.001 | 0.014 |
|  |  | 1 2 | | CN750 | LITHIUM SALTS |  | | 3,408 6,195 | 4.2% 1.3% | | <0.001 | 0.177 |
|  |  | 1 2 | | 6135 | ketoconazole |  | | 1,214 4,377 | 1.5% 0.9% | | <0.001 | 0.052 |
|  |  | 1 2 | | 10829 | trimethoprim |  | | 4,708 18,511 | 5.8% 3.9% | | <0.001 | 0.088 |
|  |  | 1 2 | | 7454 | nitrofurantoin |  | | 390 1,107 | 0.5% 0.2% | | <0.001 | 0.041 |
|  |  | 1 2 | | AM200 | ERYTHROMYCINS/MACROLIDES |  | | 7,282 28,298 | 8.9% 5.9% | | <0.001 | 0.114 |
|  |  | 1 2 | | AM300 | AMINOGLYCOSIDES |  | | 4,066 13,290 | 5.0% 2.8% | | <0.001 | 0.114 |
|  |  | 1 2 | | AN000 | ANTINEOPLASTICS |  | | 1,317 5,615 | 1.6% 1.2% | | <0.001 | 0.037 |
|  |  | 1 2 | | CN709 | ANTIPSYCHOTICS,OTHER |  | | 31,943 53,340 | 39.2% 11.2% | | <0.001 | 0.680 |
|  |  | 1 2 | | CN701 | PHENOTHIAZINE/RELATED ANTIPSYCHOTICS |  | | 2,723 2,033 | 3.3% 0.4% | | <0.001 | 0.215 |
|  |  | 1 2 | | CN609 | ANTIDEPRESSANTS,OTHER |  | | 23,460 54,923 | 28.8% 11.5% | | <0.001 | 0.439 |
|  |  | 1 2 | | CN601 | TRICYCLIC ANTIDEPRESSANTS |  | | 2,646 6,203 | 3.2% 1.3% | | <0.001 | 0.130 |
|  |  | 1 2 | | CV100 | BETA BLOCKERS/RELATED |  | | 8,723 23,716 | 10.7% 5.0% | | <0.001 | 0.214 |
|  |  | 1 2 | | CV200 | CALCIUM CHANNEL BLOCKERS |  | | 4,461 13,995 | 5.5% 2.9% | | <0.001 | 0.126 |
|  |  | 1 2 | | CV800 | ACE INHIBITORS |  | | 3,989 13,144 | 4.9% 2.8% | | <0.001 | 0.111 |
|  |  | 1 2 | | CV150 | ALPHA BLOCKERS/RELATED |  | | 3,108 6,713 | 3.8% 1.4% | | <0.001 | 0.151 |
|  |  | 1 2 | | CV490 | ANTIHYPERTENSIVES,OTHER |  | | 11,274 28,113 | 13.8% 5.9% | | <0.001 | 0.268 |
|  |  | 1 2 | | HS100 | ANDROGENS/ANABOLICS |  | | 468 1,931 | 0.6% 0.4% | | <0.001 | 0.024 |
|  |  | 1 2 | | HS800 | PROGESTINS |  | | 195 592 | 0.2% 0.1% | | <0.001 | 0.027 |
|  |  | 1 2 | | HS300 | ESTROGENS |  | | 128 561 | 0.2% 0.1% | | 0.003 | 0.011 |
|  |  | 1 2 | | 9997 | spironolactone |  | | 558 3,036 | 0.7% 0.6% | | 0.130 | 0.006 |
|  |  | 1 2 | | 3014 | cyproterone |  | | 10 10 | 0.0% 0.0% | | <0.001 | 0.012 |
|  |  | 1 2 | | 114477 | levetiracetam |  | | 19,698 57,615 | 24.2% 12.1% | | <0.001 | 0.316 |
|  |  | 1 2 | | 28439 | lamotrigine |  | | 6,996 18,824 | 8.6% 4.0% | | <0.001 | 0.191 |
|  |  | 1 2 | | 25480 | gabapentin |  | | 7,242 19,622 | 8.9% 4.1% | | <0.001 | 0.194 |
|  |  | 1 2 | | 38404 | topiramate |  | | 5,159 7,012 | 6.3% 1.5% | | <0.001 | 0.253 |
|  |  | 1 2 | | 32624 | oxcarbazepine |  | | 5,500 11,057 | 6.7% 2.3% | | <0.001 | 0.214 |
|  |  | 1 2 | | 623400 | lacosamide |  | | 3,819 4,520 | 4.7% 1.0% | | <0.001 | 0.227 |
|  |  | 1 2 | | 8183 | phenytoin |  | | 3,455 7,930 | 4.2% 1.7% | | <0.001 | 0.152 |
|  |  | 1 2 | | 21241 | clobazam |  | | 3,275 2,338 | 4.0% 0.5% | | <0.001 | 0.239 |
|  |  | 1 2 | | 2002 | carbamazepine |  | | 3,097 7,049 | 3.8% 1.5% | | <0.001 | 0.145 |
|  |  | 1 2 | | 39998 | zonisamide |  | | 2,793 3,124 | 3.4% 0.7% | | <0.001 | 0.197 |
|  |  | 1 2 | | 72236 | fosphenytoin |  | | 2,211 3,553 | 2.7% 0.7% | | <0.001 | 0.151 |
|  |  | 1 2 | | 4135 | ethosuximide |  | | 1,303 1,247 | 1.6% 0.3% | | <0.001 | 0.139 |
|  |  | 1 2 | | 69036 | rufinamide |  | | 579 313 | 0.7% 0.1% | | <0.001 | 0.104 |
|  |  | 1 2 | | 1739745 | brivaracetam |  | | 456 463 | 0.6% 0.1% | | <0.001 | 0.081 |
|  |  | 1 2 | | 14851 | vigabatrin |  | | 598 298 | 0.7% 0.1% | | <0.001 | 0.107 |
|  |  | 1 2 | | 24812 | felbamate |  | | 336 247 | 0.4% 0.1% | | <0.001 | 0.075 |
|  |  | 1 2 | | 1356552 | perampanel |  | | 387 335 | 0.5% 0.1% | | <0.001 | 0.078 |
|  |  | 1 2 | | 2045371 | cannabidiol |  | | 398 295 | 0.5% 0.1% | | <0.001 | 0.081 |
|  |  | 1 2 | | 1482502 | eslicarbazepine |  | | 181 330 | 0.2% 0.1% | | <0.001 | 0.040 |
|  |  | 1 2 | | 8691 | primidone |  | | 140 267 | 0.2% 0.1% | | <0.001 | 0.034 |
|  |  | 1 2 | | 2265690 | cenobamate |  | | 101 155 | 0.1% 0.0% | | <0.001 | 0.033 |
|  |  | 1 2 | | 31914 | tiagabine |  | | 43 49 | 0.1% 0.0% | | <0.001 | 0.024 |
|  |  | 1 2 | | 4328 | fenfluramine |  | | 27 11 | 0.0% 0.0% | | <0.001 | 0.023 |
|  |  | 1 2 | | 47858 | methsuximide |  | | 10 10 | 0.0% 0.0% | | <0.001 | 0.012 |
|  |  | 1 2 | | 2054968 | stiripentol |  | | 10 10 | 0.0% 0.0% | | <0.001 | 0.012 |
|  |  | 1 2 | | CN302 | BENZODIAZEPINE DERIVATIVE SEDATIVES/HYPNOTICS |  | | 45,095 118,817 | 55.3% 25.0% | | <0.001 | 0.650 |
|  |  | 1 2 | | CN309 | SEDATIVES/HYPNOTICS,OTHER |  | | 9,460 22,670 | 11.6% 4.8% | | <0.001 | 0.251 |
|  |  | 1 2 | | CN301 | BARBITURIC ACID DERIVATIVE SEDATIVES/HYPNOTICS |  | | 3,412 8,337 | 4.2% 1.8% | | <0.001 | 0.144 |
|  |  | 1 2 | | CN101 | OPIOID ANALGESICS |  | | 24,473 94,618 | 30.0% 19.9% | | <0.001 | 0.235 |
|  |  | 1 2 | | HS051 | GLUCOCORTICOIDS |  | | 20,259 86,432 | 24.8% 18.2% | | <0.001 | 0.163 |
|  | **Laboratory** | | | | | | | | | | | |
|  |  | Cohort | | |  | Mean ± SD | | Patients | % of Cohort | | P-Value | SMD |
|  |  | 1 2 | | 9083 | BMI | 25.7 +/- 7.1 25.3 +/- 7.5 | | 44,066 188,201 | 54.0% 39.6% | | <0.001 | 0.051 |
|  |  | 1 2 | |  | 0 - 0 kg/m2 |  | | 44,139 188,659 | 54.1% 39.7% | | <0.001 | 0.293 |
| **Cohort 1 (N = 71,327) and cohort 2 (N = 71,327) characteristics after propensity score matching** | | | | | | | | | | | | |
|  | **Demographics** | | | | | | | | | | | |
|  |  | Cohort | | |  | Mean ± SD | | Patients | % of Cohort | | P-Value | SMD |
|  |  | 1 2 | | AI | Age at Index | 24.9 +/- 12.9 26.1 +/- 13.5 | | 71,327 71,327 | 100% 100% | | <0.001 | 0.092 |
|  |  | 1 2 | | 2106-3 | White |  | | 40,273 40,869 | 56.5% 57.3% | | 0.001 | 0.017 |
|  |  | 1 2 | | 1002-5 | American Indian or Alaska Native |  | | 249 258 | 0.3% 0.4% | | 0.689 | 0.002 |
|  |  | 1 2 | | UNK | Unknown Race |  | | 10,605 10,023 | 14.9% 14.1% | | <0.001 | 0.023 |
|  |  | 1 2 | | 2076-8 | Native Hawaiian or Other Pacific Islander |  | | 304 317 | 0.4% 0.4% | | 0.601 | 0.003 |
|  |  | 1 2 | | 2054-5 | Black or African American |  | | 13,359 13,290 | 18.7% 18.6% | | 0.639 | 0.002 |
|  |  | 1 2 | | 2131-1 | Other Race |  | | 3,431 3,571 | 4.8% 5.0% | | 0.086 | 0.009 |
|  |  | 1 2 | | 2028-9 | Asian |  | | 3,106 2,999 | 4.4% 4.2% | | 0.162 | 0.007 |
|  | **Diagnosis** | | | | | | | | | | | |
|  |  | Cohort | | |  | Mean ± SD | | Patients | % of Cohort | | P-Value | SMD |
|  |  | 1 2 | | F80-F89 | Pervasive and specific developmental disorders |  | | 9,395 9,158 | 13.2% 12.8% | | 0.062 | 0.010 |
|  |  | 1 2 | | F40-F48 | Anxiety, dissociative, stress-related, somatoform and other nonpsychotic mental disorders |  | | 18,671 19,996 | 26.2% 28.0% | | <0.001 | 0.042 |
|  |  | 1 2 | | F90-F98 | Behavioral and emotional disorders with onset usually occurring in childhood and adolescence |  | | 11,310 11,630 | 15.9% 16.3% | | 0.021 | 0.012 |
|  |  | 1 2 | | F20-F29 | Schizophrenia, schizotypal, delusional, and other non-mood psychotic disorders |  | | 10,587 10,407 | 14.8% 14.6% | | 0.179 | 0.007 |
|  |  | 1 2 | | F60-F69 | Disorders of adult personality and behavior |  | | 4,999 4,952 | 7.0% 6.9% | | 0.625 | 0.003 |
|  |  | 1 2 | | F70-F79 | Intellectual Disabilities |  | | 3,760 3,562 | 5.3% 5.0% | | 0.018 | 0.013 |
|  |  | 1 2 | | F01-F09 | Mental disorders due to known physiological conditions |  | | 2,903 2,849 | 4.1% 4.0% | | 0.467 | 0.004 |
|  |  | 1 2 | | F99-F99 | Unspecified mental disorder (F99) |  | | 1,738 1,753 | 2.4% 2.5% | | 0.797 | 0.001 |
|  |  | 1 2 | | E08-E13 | Diabetes mellitus |  | | 3,474 3,777 | 4.9% 5.3% | | <0.001 | 0.019 |
|  |  | 1 2 | | Q00-Q07 | Congenital malformations of the nervous system |  | | 1,968 1,897 | 2.8% 2.7% | | 0.247 | 0.006 |
|  |  | 1 2 | | Q60-Q64 | Congenital malformations of the urinary system |  | | 403 410 | 0.6% 0.6% | | 0.806 | 0.001 |
|  |  | 1 2 | | N45 | Orchitis and epididymitis |  | | 360 392 | 0.5% 0.5% | | 0.242 | 0.006 |
|  |  | 1 2 | | N43 | Hydrocele and spermatocele |  | | 416 418 | 0.6% 0.6% | | 0.945 | <0.001 |
|  |  | 1 2 | | N50.1 | Vascular disorders of male genital organs |  | | 28 32 | 0.0% 0.0% | | 0.606 | 0.003 |
|  |  | 1 2 | | N49 | Inflammatory disorders of male genital organs, not elsewhere classified |  | | 110 123 | 0.2% 0.2% | | 0.394 | 0.005 |
|  |  | 1 2 | | I86.1 | Scrotal varices |  | | 193 194 | 0.3% 0.3% | | 0.959 | <0.001 |
|  |  | 1 2 | | Q53 | Undescended and ectopic testicle |  | | 383 372 | 0.5% 0.5% | | 0.688 | 0.002 |
|  |  | 1 2 | | Q55 | Other congenital malformations of male genital organs |  | | 267 264 | 0.4% 0.4% | | 0.896 | 0.001 |
|  |  | 1 2 | | Q54 | Hypospadias |  | | 158 135 | 0.2% 0.2% | | 0.179 | 0.007 |
|  |  | 1 2 | | C60-C63 | Malignant neoplasms of male genital organs |  | | 78 82 | 0.1% 0.1% | | 0.752 | 0.002 |
|  |  | 1 2 | | N44.8 | Other noninflammatory disorders of the testis |  | | 186 194 | 0.3% 0.3% | | 0.681 | 0.002 |
|  |  | 1 2 | | N44.0 | Torsion of testis |  | | 49 60 | 0.1% 0.1% | | 0.292 | 0.006 |
|  |  | 1 2 | | N30-N39 | Other diseases of the urinary system |  | | 3,525 3,633 | 4.9% 5.1% | | 0.190 | 0.007 |
|  |  | 1 2 | | K40 | Inguinal hernia |  | | 679 690 | 1.0% 1.0% | | 0.765 | 0.002 |
|  |  | 1 2 | | E84 | Cystic fibrosis |  | | 53 66 | 0.1% 0.1% | | 0.233 | 0.006 |
|  |  | 1 2 | | S30-S39 | Injuries to the abdomen, lower back, lumbar spine, pelvis and external genitals |  | | 4,092 4,392 | 5.7% 6.2% | | 0.001 | 0.018 |
|  |  | 1 2 | | E23.0 | Hypopituitarism |  | | 270 278 | 0.4% 0.4% | | 0.732 | 0.002 |
|  |  | 1 2 | | R56 | Convulsions, not elsewhere classified |  | | 23,982 21,933 | 33.6% 30.7% | | <0.001 | 0.062 |
|  |  | 1 2 | | E34.5 | Androgen insensitivity syndrome |  | | 10 10 | 0.0% 0.0% | | 1 | <0.001 |
|  |  | 1 2 | | Q87.1 | Congenital malformation syndromes predominantly associated with short stature |  | | 63 61 | 0.1% 0.1% | | 0.857 | 0.001 |
|  |  | 1 2 | | F31 | Bipolar disorder |  | | 21,553 23,194 | 30.2% 32.5% | | <0.001 | 0.050 |
|  |  | 1 2 | | F32 | Depressive episode |  | | 11,600 12,639 | 16.3% 17.7% | | <0.001 | 0.039 |
|  |  | 1 2 | | F39 | Unspecified mood [affective] disorder |  | | 4,866 5,041 | 6.8% 7.1% | | 0.068 | 0.010 |
|  |  | 1 2 | | F33 | Major depressive disorder, recurrent |  | | 3,488 3,824 | 4.9% 5.4% | | <0.001 | 0.021 |
|  |  | 1 2 | | F34 | Persistent mood [affective] disorders |  | | 1,613 1,671 | 2.3% 2.3% | | 0.306 | 0.005 |
|  |  | 1 2 | | F30 | Manic episode |  | | 1,544 1,488 | 2.2% 2.1% | | 0.304 | 0.005 |
|  |  | 1 2 | | F50-F59 | Behavioral syndromes associated with physiological disturbances and physical factors |  | | 1,950 2,101 | 2.7% 2.9% | | 0.016 | 0.013 |
|  |  | 1 2 | | J00-J99 | Diseases of the respiratory system |  | | 22,231 22,420 | 31.2% 31.4% | | 0.281 | 0.006 |
|  |  | 1 2 | | I00-I99 | Diseases of the circulatory system |  | | 16,697 17,429 | 23.4% 24.4% | | <0.001 | 0.024 |
|  |  | 1 2 | | K00-K95 | Diseases of the digestive system |  | | 19,717 20,288 | 27.6% 28.4% | | 0.001 | 0.018 |
|  |  | 1 2 | | Q65-Q79 | Congenital malformations and deformations of the musculoskeletal system |  | | 1,928 1,846 | 2.7% 2.6% | | 0.176 | 0.007 |
|  |  | 1 2 | | N17-N19 | Acute kidney failure and chronic kidney disease |  | | 3,224 3,425 | 4.5% 4.8% | | 0.012 | 0.013 |
|  |  | 1 2 | | A50-A64 | Infections with a predominantly sexual mode of transmission |  | | 829 929 | 1.2% 1.3% | | 0.016 | 0.013 |
|  |  | 1 2 | | E03 | Other hypothyroidism |  | | 1,835 1,886 | 2.6% 2.6% | | 0.397 | 0.004 |
|  |  | 1 2 | | E05 | Thyrotoxicosis [hyperthyroidism] |  | | 277 290 | 0.4% 0.4% | | 0.584 | 0.003 |
|  |  | 1 2 | | E83.1 | Disorders of iron metabolism |  | | 71 77 | 0.1% 0.1% | | 0.622 | 0.003 |
|  |  | 1 2 | | Q99 | Other chromosome abnormalities, not elsewhere classified |  | | 617 604 | 0.9% 0.8% | | 0.709 | 0.002 |
|  |  | 1 2 | | Q93 | Monosomies and deletions from the autosomes, not elsewhere classified |  | | 299 312 | 0.4% 0.4% | | 0.598 | 0.003 |
|  |  | 1 2 | | Q90 | Down syndrome |  | | 254 282 | 0.4% 0.4% | | 0.226 | 0.006 |
|  |  | 1 2 | | Q92 | Other trisomies and partial trisomies of the autosomes, not elsewhere classified |  | | 114 114 | 0.2% 0.2% | | 1 | <0.001 |
|  |  | 1 2 | | Q98.4 | Klinefelter syndrome, unspecified |  | | 52 58 | 0.1% 0.1% | | 0.567 | 0.003 |
|  |  | 1 2 | | Q98.5 | Karyotype 47, XYY |  | | 20 23 | 0.0% 0.0% | | 0.647 | 0.002 |
|  |  | 1 2 | | Q98.7 | Male with sex chromosome mosaicism |  | | 19 20 | 0.0% 0.0% | | 0.873 | 0.001 |
|  |  | 1 2 | | Q98.8 | Other specified sex chromosome abnormalities, male phenotype |  | | 20 24 | 0.0% 0.0% | | 0.546 | 0.003 |
|  |  | 1 2 | | Q98.0 | Klinefelter syndrome karyotype 47, XXY |  | | 10 15 | 0.0% 0.0% | | 0.317 | 0.005 |
|  |  | 1 2 | | Q98.1 | Klinefelter syndrome, male with more than two X chromosomes |  | | 10 10 | 0.0% 0.0% | | 1 | <0.001 |
|  |  | 1 2 | | Q98.6 | Male with structurally abnormal sex chromosome |  | | 10 0 | 0.0% 0% | | 0.002 | 0.017 |
|  |  | 1 2 | | Q98.9 | Sex chromosome abnormality, male phenotype, unspecified |  | | 10 0 | 0.0% 0% | | 0.002 | 0.017 |
|  |  | 1 2 | | D35.2 | Benign neoplasm of pituitary gland |  | | 75 76 | 0.1% 0.1% | | 0.935 | <0.001 |
|  |  | 1 2 | | D35.3 | Benign neoplasm of craniopharyngeal duct |  | | 36 34 | 0.1% 0.0% | | 0.811 | 0.001 |
|  |  | 1 2 | | E40-E46 | Malnutrition |  | | 1,544 1,637 | 2.2% 2.3% | | 0.095 | 0.009 |
|  |  | 1 2 | | G40.9 | Epilepsy, unspecified |  | | 21,723 20,353 | 30.5% 28.5% | | <0.001 | 0.042 |
|  |  | 1 2 | | G40.3 | Generalized idiopathic epilepsy and epileptic syndromes |  | | 6,042 5,756 | 8.5% 8.1% | | 0.006 | 0.015 |
|  |  | 1 2 | | G40.4 | Other generalized epilepsy and epileptic syndromes |  | | 4,644 4,424 | 6.5% 6.2% | | 0.017 | 0.013 |
|  |  | 1 2 | | G40.2 | Localization-related (focal) (partial) symptomatic epilepsy and epileptic syndromes with complex partial seizures |  | | 4,648 4,030 | 6.5% 5.7% | | <0.001 | 0.036 |
|  |  | 1 2 | | G40.8 | Other epilepsy and recurrent seizures |  | | 3,633 3,403 | 5.1% 4.8% | | 0.005 | 0.015 |
|  |  | 1 2 | | G40.1 | Localization-related (focal) (partial) symptomatic epilepsy and epileptic syndromes with simple partial seizures |  | | 3,290 2,946 | 4.6% 4.1% | | <0.001 | 0.024 |
|  |  | 1 2 | | G40.A | Absence epileptic syndrome |  | | 1,824 1,693 | 2.6% 2.4% | | 0.025 | 0.012 |
|  |  | 1 2 | | G40.0 | Localization-related (focal) (partial) idiopathic epilepsy and epileptic syndromes with seizures of localized onset |  | | 1,079 1,000 | 1.5% 1.4% | | 0.081 | 0.009 |
|  |  | 1 2 | | G40.5 | Epileptic seizures related to external causes |  | | 610 585 | 0.9% 0.8% | | 0.468 | 0.004 |
|  |  | 1 2 | | G40.B | Juvenile myoclonic epilepsy [impulsive petit mal] |  | | 500 470 | 0.7% 0.7% | | 0.334 | 0.005 |
|  |  | 1 2 | | F17 | Nicotine dependence |  | | 13,218 13,886 | 18.5% 19.5% | | <0.001 | 0.024 |
|  |  | 1 2 | | F12 | Cannabis related disorders |  | | 8,652 8,895 | 12.1% 12.5% | | 0.050 | 0.010 |
|  |  | 1 2 | | F10 | Alcohol related disorders |  | | 7,365 7,798 | 10.3% 10.9% | | <0.001 | 0.020 |
|  |  | 1 2 | | F19 | Other psychoactive substance related disorders |  | | 6,129 6,479 | 8.6% 9.1% | | 0.001 | 0.017 |
|  |  | 1 2 | | F11 | Opioid related disorders |  | | 3,582 3,829 | 5.0% 5.4% | | 0.003 | 0.016 |
|  |  | 1 2 | | F14 | Cocaine related disorders |  | | 3,080 3,180 | 4.3% 4.5% | | 0.196 | 0.007 |
|  |  | 1 2 | | F15 | Other stimulant related disorders |  | | 2,895 3,031 | 4.1% 4.2% | | 0.071 | 0.010 |
|  |  | 1 2 | | F13 | Sedative, hypnotic, or anxiolytic related disorders |  | | 1,544 1,581 | 2.2% 2.2% | | 0.503 | 0.004 |
|  |  | 1 2 | | F18 | Inhalant related disorders |  | | 1,173 1,218 | 1.6% 1.7% | | 0.353 | 0.005 |
|  |  | 1 2 | | F16 | Hallucinogen related disorders |  | | 648 669 | 0.9% 0.9% | | 0.561 | 0.003 |
|  | **Procedure** | | | | | | | | | | | |
|  |  | Cohort | | |  | Mean ± SD | | Patients | % of Cohort | | P-Value | SMD |
|  |  | 1 2 | | 1010843 | Radiation Oncology Treatment |  | | 134 144 | 0.2% 0.2% | | 0.548 | 0.003 |
|  |  | 1 2 | | 1008061 | Surgical Procedures on the Urinary System |  | | 1,822 1,871 | 2.6% 2.6% | | 0.414 | 0.004 |
|  |  | 1 2 | | 1008011 | Repair initial inguinal hernia, age 5 years or older |  | | 89 98 | 0.1% 0.1% | | 0.510 | 0.003 |
|  |  | 1 2 | | 1008470 | Surgical Procedures on the Male Genital System |  | | 651 665 | 0.9% 0.9% | | 0.698 | 0.002 |
|  | **Medication** | | | | | | | | | | | |
|  |  | Cohort | | |  | Mean ± SD | | Patients | % of Cohort | | P-Value | SMD |
|  |  | 1 2 | | 25025 | finasteride |  | | 144 160 | 0.2% 0.2% | | 0.358 | 0.005 |
|  |  | 1 2 | | CN750 | LITHIUM SALTS |  | | 2,745 2,728 | 3.8% 3.8% | | 0.815 | 0.001 |
|  |  | 1 2 | | 6135 | ketoconazole |  | | 943 1,021 | 1.3% 1.4% | | 0.076 | 0.009 |
|  |  | 1 2 | | 10829 | trimethoprim |  | | 3,767 3,977 | 5.3% 5.6% | | 0.014 | 0.013 |
|  |  | 1 2 | | 7454 | nitrofurantoin |  | | 301 308 | 0.4% 0.4% | | 0.776 | 0.002 |
|  |  | 1 2 | | AM200 | ERYTHROMYCINS/MACROLIDES |  | | 5,848 6,309 | 8.2% 8.8% | | <0.001 | 0.023 |
|  |  | 1 2 | | AM300 | AMINOGLYCOSIDES |  | | 3,121 3,321 | 4.4% 4.7% | | 0.011 | 0.014 |
|  |  | 1 2 | | AN000 | ANTINEOPLASTICS |  | | 1,073 1,194 | 1.5% 1.7% | | 0.010 | 0.014 |
|  |  | 1 2 | | CN709 | ANTIPSYCHOTICS,OTHER |  | | 26,577 27,645 | 37.3% 38.8% | | <0.001 | 0.031 |
|  |  | 1 2 | | CN701 | PHENOTHIAZINE/RELATED ANTIPSYCHOTICS |  | | 1,655 1,470 | 2.3% 2.1% | | 0.001 | 0.018 |
|  |  | 1 2 | | CN609 | ANTIDEPRESSANTS,OTHER |  | | 19,373 20,893 | 27.2% 29.3% | | <0.001 | 0.047 |
|  |  | 1 2 | | CN601 | TRICYCLIC ANTIDEPRESSANTS |  | | 2,111 2,323 | 3.0% 3.3% | | 0.001 | 0.017 |
|  |  | 1 2 | | CV100 | BETA BLOCKERS/RELATED |  | | 7,111 7,504 | 10.0% 10.5% | | 0.001 | 0.018 |
|  |  | 1 2 | | CV200 | CALCIUM CHANNEL BLOCKERS |  | | 3,708 3,985 | 5.2% 5.6% | | 0.001 | 0.017 |
|  |  | 1 2 | | CV800 | ACE INHIBITORS |  | | 3,308 3,638 | 4.6% 5.1% | | <0.001 | 0.021 |
|  |  | 1 2 | | CV150 | ALPHA BLOCKERS/RELATED |  | | 2,459 2,600 | 3.4% 3.6% | | 0.044 | 0.011 |
|  |  | 1 2 | | CV490 | ANTIHYPERTENSIVES,OTHER |  | | 8,828 9,467 | 12.4% 13.3% | | <0.001 | 0.027 |
|  |  | 1 2 | | HS100 | ANDROGENS/ANABOLICS |  | | 397 454 | 0.6% 0.6% | | 0.050 | 0.010 |
|  |  | 1 2 | | HS800 | PROGESTINS |  | | 157 164 | 0.2% 0.2% | | 0.696 | 0.002 |
|  |  | 1 2 | | HS300 | ESTROGENS |  | | 107 125 | 0.2% 0.2% | | 0.237 | 0.006 |
|  |  | 1 2 | | 9997 | spironolactone |  | | 481 557 | 0.7% 0.8% | | 0.018 | 0.013 |
|  |  | 1 2 | | 3014 | cyproterone |  | | 10 10 | 0.0% 0.0% | | 1 | <0.001 |
|  |  | 1 2 | | 114477 | levetiracetam |  | | 14,673 14,912 | 20.6% 20.9% | | 0.119 | 0.008 |
|  |  | 1 2 | | 28439 | lamotrigine |  | | 5,235 5,567 | 7.3% 7.8% | | 0.001 | 0.018 |
|  |  | 1 2 | | 25480 | gabapentin |  | | 6,017 6,375 | 8.4% 8.9% | | 0.001 | 0.018 |
|  |  | 1 2 | | 38404 | topiramate |  | | 3,214 3,281 | 4.5% 4.6% | | 0.395 | 0.005 |
|  |  | 1 2 | | 32624 | oxcarbazepine |  | | 3,708 3,774 | 5.2% 5.3% | | 0.433 | 0.004 |
|  |  | 1 2 | | 623400 | lacosamide |  | | 2,207 2,251 | 3.1% 3.2% | | 0.503 | 0.004 |
|  |  | 1 2 | | 8183 | phenytoin |  | | 2,421 2,546 | 3.4% 3.6% | | 0.071 | 0.010 |
|  |  | 1 2 | | 21241 | clobazam |  | | 1,634 1,576 | 2.3% 2.2% | | 0.300 | 0.005 |
|  |  | 1 2 | | 2002 | carbamazepine |  | | 2,227 2,334 | 3.1% 3.3% | | 0.107 | 0.009 |
|  |  | 1 2 | | 39998 | zonisamide |  | | 1,518 1,518 | 2.1% 2.1% | | 1 | <0.001 |
|  |  | 1 2 | | 72236 | fosphenytoin |  | | 1,199 1,170 | 1.7% 1.6% | | 0.548 | 0.003 |
|  |  | 1 2 | | 4135 | ethosuximide |  | | 815 759 | 1.1% 1.1% | | 0.156 | 0.008 |
|  |  | 1 2 | | 69036 | rufinamide |  | | 258 232 | 0.4% 0.3% | | 0.239 | 0.006 |
|  |  | 1 2 | | 1739745 | brivaracetam |  | | 218 220 | 0.3% 0.3% | | 0.924 | 0.001 |
|  |  | 1 2 | | 14851 | vigabatrin |  | | 240 233 | 0.3% 0.3% | | 0.747 | 0.002 |
|  |  | 1 2 | | 24812 | felbamate |  | | 178 175 | 0.2% 0.2% | | 0.873 | 0.001 |
|  |  | 1 2 | | 1356552 | perampanel |  | | 184 192 | 0.3% 0.3% | | 0.680 | 0.002 |
|  |  | 1 2 | | 2045371 | cannabidiol |  | | 170 180 | 0.2% 0.3% | | 0.593 | 0.003 |
|  |  | 1 2 | | 1482502 | eslicarbazepine |  | | 110 106 | 0.2% 0.1% | | 0.785 | 0.001 |
|  |  | 1 2 | | 8691 | primidone |  | | 104 116 | 0.1% 0.2% | | 0.418 | 0.004 |
|  |  | 1 2 | | 2265690 | cenobamate |  | | 53 55 | 0.1% 0.1% | | 0.847 | 0.001 |
|  |  | 1 2 | | 31914 | tiagabine |  | | 28 32 | 0.0% 0.0% | | 0.606 | 0.003 |
|  |  | 1 2 | | 4328 | fenfluramine |  | | 10 10 | 0.0% 0.0% | | 1 | <0.001 |
|  |  | 1 2 | | 47858 | methsuximide |  | | 10 10 | 0.0% 0.0% | | 1 | <0.001 |
|  |  | 1 2 | | 2054968 | stiripentol |  | | 10 10 | 0.0% 0.0% | | 1 | <0.001 |
|  |  | 1 2 | | CN302 | BENZODIAZEPINE DERIVATIVE SEDATIVES/HYPNOTICS |  | | 36,224 37,140 | 50.8% 52.1% | | <0.001 | 0.026 |
|  |  | 1 2 | | CN309 | SEDATIVES/HYPNOTICS,OTHER |  | | 7,250 7,568 | 10.2% 10.6% | | 0.006 | 0.015 |
|  |  | 1 2 | | CN301 | BARBITURIC ACID DERIVATIVE SEDATIVES/HYPNOTICS |  | | 2,245 2,310 | 3.1% 3.2% | | 0.328 | 0.005 |
|  |  | 1 2 | | CN101 | OPIOID ANALGESICS |  | | 20,118 21,598 | 28.2% 30.3% | | <0.001 | 0.046 |
|  |  | 1 2 | | HS051 | GLUCOCORTICOIDS |  | | 16,300 17,148 | 22.9% 24.0% | | <0.001 | 0.028 |
|  | **Laboratory** | | | | | | | | | | | |
|  |  | Cohort | | |  | Mean ± SD | | Patients | % of Cohort | | P-Value | SMD |
|  |  | 1 2 | | 9083 | BMI | 25.9 +/- 7.1 26.2 +/- 7.3 | | 37,203 39,421 | 52.2% 55.3% | | <0.001 | 0.039 |
|  |  | 1 2 | |  | 0 - 0 kg/m2 |  | | 37,272 39,503 | 52.3% 55.4% | | <0.001 | 0.063 |

**Abbreviations:** BMI = Body Mass Index; SD = Standard deviation; SMD = Standardised mean difference

**N.B.:** Uncorrected p-values were calculated using two-sided t-tests for continuous covariates and two-sided Z-tests for categorical covariates. These p-values reflect descriptive comparisons of baseline characteristics during propensity score matching. Correction for multiple comparisons is not appropriate in this context, as the aim is to assess covariate balance (reflected by a standardised mean difference of <0.1) rather than to test hypotheses. Exact p-values beyond three decimal places are not provided in the propensity score matching outputs generated by the TriNetX platform. Where p-values fall below this threshold, they are reported as <0.001.

# Supplementary Table 8: Propensity score matching men with epilepsy or bipolar disorder exposed (cohort 1) and unexposed (cohort 2) to valproate – 2-year outcome assessment

Propensity score matching was performed on all listed characteristics. Characteristics of the cohorts before and after matching are summarized in the table below.

| **Cohort 1 and cohort 2 patient count before and after propensity score matching** | | | | | | | | | | | | |
| --- | --- | --- | --- | --- | --- | --- | --- | --- | --- | --- | --- | --- |
|  | | | Cohort | | | Patient count before matching | | | | Patient count after matching | | |
|  | | | 1 - Men with epilepsy or bipolar disorder exposed to valproate_v9 | | | 83,222 | | | | 70,878 | | |
|  | | | 2 - Men with epilepsy or bipolar disorder not exposed to valproate_v9 | | | 478,058 | | | | 70,878 | | |
| **Propensity score density function - Before and after matching (cohort 1 - purple, cohort 2 - green)** | | | | | | | | | | | | |
|  |  | | 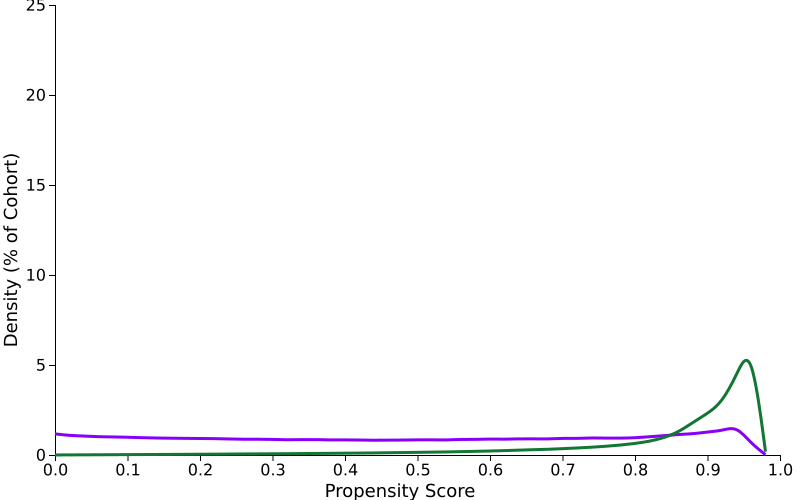 | | | | 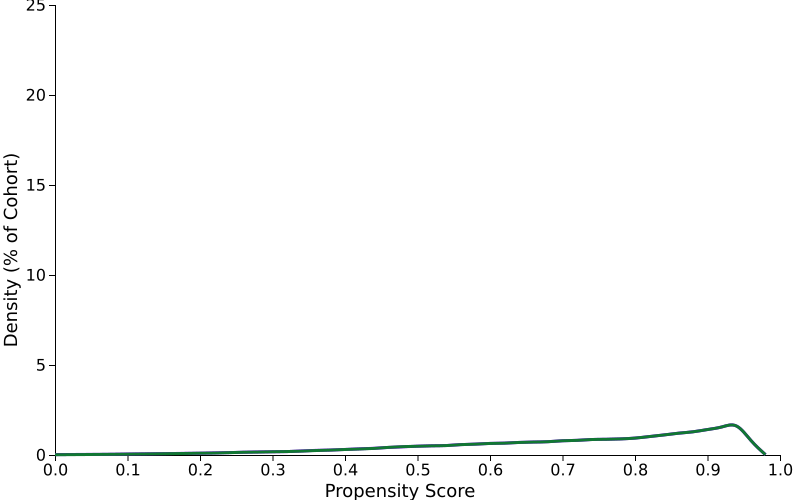 | | | | | |
| **Cohort 1 (N = 83,222) and cohort 2 (N = 478,058) characteristics before propensity score matching** | | | | | | | | | | | | |
|  | **Demographics** | | | | | | | | | | | |
|  |  | Cohort | | |  | Mean ± SD | | Patients | % of Cohort | | P-Value | SMD |
|  |  | 1 2 | | AI | Age at Index | 24.6 +/- 13.0 20.4 +/- 15.0 | | 80,835 457,515 | 100% 100% | | <0.001 | 0.296 |
|  |  | 1 2 | | 2106-3 | White |  | | 46,161 255,454 | 57.1% 55.8% | | <0.001 | 0.026 |
|  |  | 1 2 | | 1002-5 | American Indian or Alaska Native |  | | 314 1,573 | 0.4% 0.3% | | 0.048 | 0.007 |
|  |  | 1 2 | | UNK | Unknown Race |  | | 11,380 77,449 | 14.1% 16.9% | | <0.001 | 0.079 |
|  |  | 1 2 | | 2076-8 | Native Hawaiian or Other Pacific Islander |  | | 340 2,252 | 0.4% 0.5% | | 0.007 | 0.011 |
|  |  | 1 2 | | 2054-5 | Black or African American |  | | 15,152 79,322 | 18.7% 17.3% | | <0.001 | 0.037 |
|  |  | 1 2 | | 2131-1 | Other Race |  | | 3,982 26,294 | 4.9% 5.7% | | <0.001 | 0.037 |
|  |  | 1 2 | | 2028-9 | Asian |  | | 3,506 15,171 | 4.3% 3.3% | | <0.001 | 0.053 |
|  | **Diagnosis** | | | | | | | | | | | |
|  |  | Cohort | | |  | Mean ± SD | | Patients | % of Cohort | | P-Value | SMD |
|  |  | 1 2 | | F80-F89 | Pervasive and specific developmental disorders |  | | 13,430 43,922 | 16.6% 9.6% | | <0.001 | 0.209 |
|  |  | 1 2 | | F40-F48 | Anxiety, dissociative, stress-related, somatoform and other nonpsychotic mental disorders |  | | 22,505 77,122 | 27.8% 16.9% | | <0.001 | 0.266 |
|  |  | 1 2 | | F90-F98 | Behavioral and emotional disorders with onset usually occurring in childhood and adolescence |  | | 14,815 46,179 | 18.3% 10.1% | | <0.001 | 0.237 |
|  |  | 1 2 | | F20-F29 | Schizophrenia, schizotypal, delusional, and other non-mood psychotic disorders |  | | 12,896 24,031 | 16.0% 5.3% | | <0.001 | 0.353 |
|  |  | 1 2 | | F60-F69 | Disorders of adult personality and behavior |  | | 6,950 12,610 | 8.6% 2.8% | | <0.001 | 0.254 |
|  |  | 1 2 | | F70-F79 | Intellectual Disabilities |  | | 6,207 9,054 | 7.7% 2.0% | | <0.001 | 0.268 |
|  |  | 1 2 | | F01-F09 | Mental disorders due to known physiological conditions |  | | 4,261 7,528 | 5.3% 1.6% | | <0.001 | 0.199 |
|  |  | 1 2 | | F99-F99 | Unspecified mental disorder (F99) |  | | 2,349 4,456 | 2.9% 1.0% | | <0.001 | 0.140 |
|  |  | 1 2 | | E08-E13 | Diabetes mellitus |  | | 3,955 15,889 | 4.9% 3.5% | | <0.001 | 0.071 |
|  |  | 1 2 | | Q00-Q07 | Congenital malformations of the nervous system |  | | 3,101 9,540 | 3.8% 2.1% | | <0.001 | 0.103 |
|  |  | 1 2 | | Q60-Q64 | Congenital malformations of the urinary system |  | | 518 2,801 | 0.6% 0.6% | | 0.338 | 0.004 |
|  |  | 1 2 | | N45 | Orchitis and epididymitis |  | | 411 1,588 | 0.5% 0.3% | | <0.001 | 0.025 |
|  |  | 1 2 | | N43 | Hydrocele and spermatocele |  | | 528 2,552 | 0.7% 0.6% | | 0.001 | 0.012 |
|  |  | 1 2 | | N50.1 | Vascular disorders of male genital organs |  | | 34 129 | 0.0% 0.0% | | 0.037 | 0.007 |
|  |  | 1 2 | | N49 | Inflammatory disorders of male genital organs, not elsewhere classified |  | | 123 493 | 0.2% 0.1% | | 0.001 | 0.012 |
|  |  | 1 2 | | I86.1 | Scrotal varices |  | | 213 923 | 0.3% 0.2% | | <0.001 | 0.013 |
|  |  | 1 2 | | Q53 | Undescended and ectopic testicle |  | | 559 2,727 | 0.7% 0.6% | | 0.001 | 0.012 |
|  |  | 1 2 | | Q55 | Other congenital malformations of male genital organs |  | | 378 2,462 | 0.5% 0.5% | | 0.011 | 0.010 |
|  |  | 1 2 | | Q54 | Hypospadias |  | | 212 1,487 | 0.3% 0.3% | | 0.003 | 0.012 |
|  |  | 1 2 | | C60-C63 | Malignant neoplasms of male genital organs |  | | 89 505 | 0.1% 0.1% | | 0.982 | <0.001 |
|  |  | 1 2 | | N44.8 | Other noninflammatory disorders of the testis |  | | 234 656 | 0.3% 0.1% | | <0.001 | 0.031 |
|  |  | 1 2 | | N44.0 | Torsion of testis |  | | 56 284 | 0.1% 0.1% | | 0.452 | 0.003 |
|  |  | 1 2 | | N30-N39 | Other diseases of the urinary system |  | | 4,458 14,788 | 5.5% 3.2% | | <0.001 | 0.112 |
|  |  | 1 2 | | K40 | Inguinal hernia |  | | 837 4,595 | 1.0% 1.0% | | 0.415 | 0.003 |
|  |  | 1 2 | | E84 | Cystic fibrosis |  | | 71 329 | 0.1% 0.1% | | 0.126 | 0.006 |
|  |  | 1 2 | | S30-S39 | Injuries to the abdomen, lower back, lumbar spine, pelvis and external genitals |  | | 4,859 17,200 | 6.0% 3.8% | | <0.001 | 0.105 |
|  |  | 1 2 | | E23.0 | Hypopituitarism |  | | 360 1,394 | 0.4% 0.3% | | <0.001 | 0.023 |
|  |  | 1 2 | | R56 | Convulsions, not elsewhere classified |  | | 29,603 225,529 | 36.6% 49.3% | | <0.001 | 0.258 |
|  |  | 1 2 | | E34.5 | Androgen insensitivity syndrome |  | | 10 12 | 0.0% 0.0% | | <0.001 | 0.011 |
|  |  | 1 2 | | Q87.1 | Congenital malformation syndromes predominantly associated with short stature |  | | 88 341 | 0.1% 0.1% | | 0.001 | 0.011 |
|  |  | 1 2 | | F31 | Bipolar disorder |  | | 23,520 108,651 | 29.1% 23.7% | | <0.001 | 0.122 |
|  |  | 1 2 | | F32 | Depressive episode |  | | 13,786 44,654 | 17.1% 9.8% | | <0.001 | 0.215 |
|  |  | 1 2 | | F39 | Unspecified mood [affective] disorder |  | | 6,534 11,600 | 8.1% 2.5% | | <0.001 | 0.249 |
|  |  | 1 2 | | F33 | Major depressive disorder, recurrent |  | | 4,071 12,353 | 5.0% 2.7% | | <0.001 | 0.121 |
|  |  | 1 2 | | F34 | Persistent mood [affective] disorders |  | | 2,074 4,902 | 2.6% 1.1% | | <0.001 | 0.112 |
|  |  | 1 2 | | F30 | Manic episode |  | | 2,059 2,484 | 2.5% 0.5% | | <0.001 | 0.163 |
|  |  | 1 2 | | F50-F59 | Behavioral syndromes associated with physiological disturbances and physical factors |  | | 2,531 7,404 | 3.1% 1.6% | | <0.001 | 0.099 |
|  |  | 1 2 | | J00-J99 | Diseases of the respiratory system |  | | 26,786 133,861 | 33.1% 29.3% | | <0.001 | 0.084 |
|  |  | 1 2 | | I00-I99 | Diseases of the circulatory system |  | | 20,025 82,791 | 24.8% 18.1% | | <0.001 | 0.163 |
|  |  | 1 2 | | K00-K95 | Diseases of the digestive system |  | | 24,233 107,859 | 30.0% 23.6% | | <0.001 | 0.145 |
|  |  | 1 2 | | Q65-Q79 | Congenital malformations and deformations of the musculoskeletal system |  | | 2,773 14,403 | 3.4% 3.1% | | <0.001 | 0.016 |
|  |  | 1 2 | | N17-N19 | Acute kidney failure and chronic kidney disease |  | | 3,766 14,883 | 4.7% 3.3% | | <0.001 | 0.072 |
|  |  | 1 2 | | A50-A64 | Infections with a predominantly sexual mode of transmission |  | | 924 3,757 | 1.1% 0.8% | | <0.001 | 0.033 |
|  |  | 1 2 | | E03 | Other hypothyroidism |  | | 2,398 7,021 | 3.0% 1.5% | | <0.001 | 0.097 |
|  |  | 1 2 | | E05 | Thyrotoxicosis [hyperthyroidism] |  | | 341 982 | 0.4% 0.2% | | <0.001 | 0.037 |
|  |  | 1 2 | | E83.1 | Disorders of iron metabolism |  | | 86 473 | 0.1% 0.1% | | 0.807 | 0.001 |
|  |  | 1 2 | | Q99 | Other chromosome abnormalities, not elsewhere classified |  | | 1,030 2,779 | 1.3% 0.6% | | <0.001 | 0.069 |
|  |  | 1 2 | | Q93 | Monosomies and deletions from the autosomes, not elsewhere classified |  | | 468 1,519 | 0.6% 0.3% | | <0.001 | 0.037 |
|  |  | 1 2 | | Q90 | Down syndrome |  | | 312 1,640 | 0.4% 0.4% | | 0.230 | 0.005 |
|  |  | 1 2 | | Q92 | Other trisomies and partial trisomies of the autosomes, not elsewhere classified |  | | 191 515 | 0.2% 0.1% | | <0.001 | 0.030 |
|  |  | 1 2 | | Q98.4 | Klinefelter syndrome, unspecified |  | | 79 207 | 0.1% 0.0% | | <0.001 | 0.020 |
|  |  | 1 2 | | Q98.5 | Karyotype 47, XYY |  | | 31 93 | 0.0% 0.0% | | 0.002 | 0.011 |
|  |  | 1 2 | | Q98.7 | Male with sex chromosome mosaicism |  | | 31 58 | 0.0% 0.0% | | <0.001 | 0.016 |
|  |  | 1 2 | | Q98.8 | Other specified sex chromosome abnormalities, male phenotype |  | | 31 65 | 0.0% 0.0% | | <0.001 | 0.015 |
|  |  | 1 2 | | Q98.0 | Klinefelter syndrome karyotype 47, XXY |  | | 16 48 | 0.0% 0.0% | | 0.025 | 0.008 |
|  |  | 1 2 | | Q98.1 | Klinefelter syndrome, male with more than two X chromosomes |  | | 10 13 | 0.0% 0.0% | | <0.001 | 0.011 |
|  |  | 1 2 | | Q98.6 | Male with structurally abnormal sex chromosome |  | | 10 10 | 0.0% 0.0% | | <0.001 | 0.012 |
|  |  | 1 2 | | Q98.9 | Sex chromosome abnormality, male phenotype, unspecified |  | | 10 10 | 0.0% 0.0% | | <0.001 | 0.012 |
|  |  | 1 2 | | D35.2 | Benign neoplasm of pituitary gland |  | | 91 338 | 0.1% 0.1% | | <0.001 | 0.013 |
|  |  | 1 2 | | D35.3 | Benign neoplasm of craniopharyngeal duct |  | | 38 138 | 0.0% 0.0% | | 0.015 | 0.009 |
|  |  | 1 2 | | E40-E46 | Malnutrition |  | | 2,051 6,828 | 2.5% 1.5% | | <0.001 | 0.074 |
|  |  | 1 2 | | G40.9 | Epilepsy, unspecified |  | | 28,686 88,846 | 35.5% 19.4% | | <0.001 | 0.366 |
|  |  | 1 2 | | G40.3 | Generalized idiopathic epilepsy and epileptic syndromes |  | | 10,128 14,003 | 12.5% 3.1% | | <0.001 | 0.359 |
|  |  | 1 2 | | G40.4 | Other generalized epilepsy and epileptic syndromes |  | | 8,444 12,000 | 10.4% 2.6% | | <0.001 | 0.321 |
|  |  | 1 2 | | G40.2 | Localization-related (focal) (partial) symptomatic epilepsy and epileptic syndromes with complex partial seizures |  | | 7,624 17,916 | 9.4% 3.9% | | <0.001 | 0.222 |
|  |  | 1 2 | | G40.8 | Other epilepsy and recurrent seizures |  | | 6,222 16,268 | 7.7% 3.6% | | <0.001 | 0.180 |
|  |  | 1 2 | | G40.1 | Localization-related (focal) (partial) symptomatic epilepsy and epileptic syndromes with simple partial seizures |  | | 5,873 13,536 | 7.3% 3.0% | | <0.001 | 0.196 |
|  |  | 1 2 | | G40.A | Absence epileptic syndrome |  | | 3,103 4,804 | 3.8% 1.1% | | <0.001 | 0.181 |
|  |  | 1 2 | | G40.0 | Localization-related (focal) (partial) idiopathic epilepsy and epileptic syndromes with seizures of localized onset |  | | 1,916 4,383 | 2.4% 1.0% | | <0.001 | 0.111 |
|  |  | 1 2 | | G40.5 | Epileptic seizures related to external causes |  | | 1,157 2,394 | 1.4% 0.5% | | <0.001 | 0.092 |
|  |  | 1 2 | | G40.B | Juvenile myoclonic epilepsy [impulsive petit mal] |  | | 842 654 | 1.0% 0.1% | | <0.001 | 0.117 |
|  |  | 1 2 | | F17 | Nicotine dependence |  | | 15,388 56,057 | 19.0% 12.3% | | <0.001 | 0.188 |
|  |  | 1 2 | | F12 | Cannabis related disorders |  | | 10,401 25,909 | 12.9% 5.7% | | <0.001 | 0.250 |
|  |  | 1 2 | | F10 | Alcohol related disorders |  | | 8,243 32,688 | 10.2% 7.1% | | <0.001 | 0.109 |
|  |  | 1 2 | | F19 | Other psychoactive substance related disorders |  | | 7,071 20,054 | 8.7% 4.4% | | <0.001 | 0.177 |
|  |  | 1 2 | | F11 | Opioid related disorders |  | | 4,155 15,063 | 5.1% 3.3% | | <0.001 | 0.092 |
|  |  | 1 2 | | F14 | Cocaine related disorders |  | | 3,605 9,510 | 4.5% 2.1% | | <0.001 | 0.134 |
|  |  | 1 2 | | F15 | Other stimulant related disorders |  | | 3,470 9,250 | 4.3% 2.0% | | <0.001 | 0.130 |
|  |  | 1 2 | | F13 | Sedative, hypnotic, or anxiolytic related disorders |  | | 1,878 4,983 | 2.3% 1.1% | | <0.001 | 0.095 |
|  |  | 1 2 | | F18 | Inhalant related disorders |  | | 1,562 4,186 | 1.9% 0.9% | | <0.001 | 0.086 |
|  |  | 1 2 | | F16 | Hallucinogen related disorders |  | | 844 1,805 | 1.0% 0.4% | | <0.001 | 0.077 |
|  | **Procedure** | | | | | | | | | | | |
|  |  | Cohort | | |  | Mean ± SD | | Patients | % of Cohort | | P-Value | SMD |
|  |  | 1 2 | | 1010843 | Radiation Oncology Treatment |  | | 171 698 | 0.2% 0.2% | | <0.001 | 0.014 |
|  |  | 1 2 | | 1008061 | Surgical Procedures on the Urinary System |  | | 2,449 8,452 | 3.0% 1.8% | | <0.001 | 0.077 |
|  |  | 1 2 | | 1008011 | Repair initial inguinal hernia, age 5 years or older |  | | 110 408 | 0.1% 0.1% | | <0.001 | 0.014 |
|  |  | 1 2 | | 1008470 | Surgical Procedures on the Male Genital System |  | | 865 5,053 | 1.1% 1.1% | | 0.388 | 0.003 |
|  | **Medication** | | | | | | | | | | | |
|  |  | Cohort | | |  | Mean ± SD | | Patients | % of Cohort | | P-Value | SMD |
|  |  | 1 2 | | 25025 | finasteride |  | | 176 692 | 0.2% 0.2% | | <0.001 | 0.015 |
|  |  | 1 2 | | CN750 | LITHIUM SALTS |  | | 3,433 6,205 | 4.2% 1.4% | | <0.001 | 0.176 |
|  |  | 1 2 | | 6135 | ketoconazole |  | | 1,222 4,442 | 1.5% 1.0% | | <0.001 | 0.049 |
|  |  | 1 2 | | 10829 | trimethoprim |  | | 4,676 17,987 | 5.8% 3.9% | | <0.001 | 0.086 |
|  |  | 1 2 | | 7454 | nitrofurantoin |  | | 397 1,058 | 0.5% 0.2% | | <0.001 | 0.043 |
|  |  | 1 2 | | AM200 | ERYTHROMYCINS/MACROLIDES |  | | 7,112 26,478 | 8.8% 5.8% | | <0.001 | 0.116 |
|  |  | 1 2 | | AM300 | AMINOGLYCOSIDES |  | | 4,081 13,269 | 5.0% 2.9% | | <0.001 | 0.110 |
|  |  | 1 2 | | AN000 | ANTINEOPLASTICS |  | | 1,379 5,801 | 1.7% 1.3% | | <0.001 | 0.036 |
|  |  | 1 2 | | CN709 | ANTIPSYCHOTICS,OTHER |  | | 32,006 52,591 | 39.6% 11.5% | | <0.001 | 0.681 |
|  |  | 1 2 | | CN701 | PHENOTHIAZINE/RELATED ANTIPSYCHOTICS |  | | 2,670 1,950 | 3.3% 0.4% | | <0.001 | 0.214 |
|  |  | 1 2 | | CN609 | ANTIDEPRESSANTS,OTHER |  | | 23,939 55,264 | 29.6% 12.1% | | <0.001 | 0.442 |
|  |  | 1 2 | | CN601 | TRICYCLIC ANTIDEPRESSANTS |  | | 2,703 6,270 | 3.3% 1.4% | | <0.001 | 0.130 |
|  |  | 1 2 | | CV100 | BETA BLOCKERS/RELATED |  | | 8,483 21,828 | 10.5% 4.8% | | <0.001 | 0.217 |
|  |  | 1 2 | | CV200 | CALCIUM CHANNEL BLOCKERS |  | | 4,349 13,029 | 5.4% 2.8% | | <0.001 | 0.128 |
|  |  | 1 2 | | CV800 | ACE INHIBITORS |  | | 4,005 12,711 | 5.0% 2.8% | | <0.001 | 0.113 |
|  |  | 1 2 | | CV150 | ALPHA BLOCKERS/RELATED |  | | 3,106 6,433 | 3.8% 1.4% | | <0.001 | 0.153 |
|  |  | 1 2 | | CV490 | ANTIHYPERTENSIVES,OTHER |  | | 11,144 26,333 | 13.8% 5.8% | | <0.001 | 0.273 |
|  |  | 1 2 | | HS100 | ANDROGENS/ANABOLICS |  | | 477 1,991 | 0.6% 0.4% | | <0.001 | 0.022 |
|  |  | 1 2 | | HS800 | PROGESTINS |  | | 203 598 | 0.3% 0.1% | | <0.001 | 0.028 |
|  |  | 1 2 | | HS300 | ESTROGENS |  | | 139 588 | 0.2% 0.1% | | 0.002 | 0.011 |
|  |  | 1 2 | | 9997 | spironolactone |  | | 557 2,936 | 0.7% 0.6% | | 0.122 | 0.006 |
|  |  | 1 2 | | 3014 | cyproterone |  | | 10 10 | 0.0% 0.0% | | <0.001 | 0.012 |
|  |  | 1 2 | | 114477 | levetiracetam |  | | 19,346 55,990 | 23.9% 12.2% | | <0.001 | 0.307 |
|  |  | 1 2 | | 28439 | lamotrigine |  | | 7,084 19,173 | 8.8% 4.2% | | <0.001 | 0.187 |
|  |  | 1 2 | | 25480 | gabapentin |  | | 7,385 19,391 | 9.1% 4.2% | | <0.001 | 0.197 |
|  |  | 1 2 | | 38404 | topiramate |  | | 5,175 7,045 | 6.4% 1.5% | | <0.001 | 0.251 |
|  |  | 1 2 | | 32624 | oxcarbazepine |  | | 5,449 10,997 | 6.7% 2.4% | | <0.001 | 0.209 |
|  |  | 1 2 | | 623400 | lacosamide |  | | 3,798 4,570 | 4.7% 1.0% | | <0.001 | 0.224 |
|  |  | 1 2 | | 8183 | phenytoin |  | | 3,352 7,457 | 4.1% 1.6% | | <0.001 | 0.151 |
|  |  | 1 2 | | 21241 | clobazam |  | | 3,308 2,341 | 4.1% 0.5% | | <0.001 | 0.240 |
|  |  | 1 2 | | 2002 | carbamazepine |  | | 3,057 6,974 | 3.8% 1.5% | | <0.001 | 0.141 |
|  |  | 1 2 | | 39998 | zonisamide |  | | 2,782 3,161 | 3.4% 0.7% | | <0.001 | 0.194 |
|  |  | 1 2 | | 72236 | fosphenytoin |  | | 2,184 3,440 | 2.7% 0.8% | | <0.001 | 0.150 |
|  |  | 1 2 | | 4135 | ethosuximide |  | | 1,321 1,232 | 1.6% 0.3% | | <0.001 | 0.141 |
|  |  | 1 2 | | 69036 | rufinamide |  | | 596 325 | 0.7% 0.1% | | <0.001 | 0.105 |
|  |  | 1 2 | | 1739745 | brivaracetam |  | | 438 438 | 0.5% 0.1% | | <0.001 | 0.079 |
|  |  | 1 2 | | 14851 | vigabatrin |  | | 604 299 | 0.7% 0.1% | | <0.001 | 0.107 |
|  |  | 1 2 | | 24812 | felbamate |  | | 343 253 | 0.4% 0.1% | | <0.001 | 0.076 |
|  |  | 1 2 | | 1356552 | perampanel |  | | 379 325 | 0.5% 0.1% | | <0.001 | 0.077 |
|  |  | 1 2 | | 2045371 | cannabidiol |  | | 401 303 | 0.5% 0.1% | | <0.001 | 0.081 |
|  |  | 1 2 | | 1482502 | eslicarbazepine |  | | 187 325 | 0.2% 0.1% | | <0.001 | 0.041 |
|  |  | 1 2 | | 8691 | primidone |  | | 142 267 | 0.2% 0.1% | | <0.001 | 0.034 |
|  |  | 1 2 | | 2265690 | cenobamate |  | | 96 164 | 0.1% 0.0% | | <0.001 | 0.030 |
|  |  | 1 2 | | 31914 | tiagabine |  | | 39 53 | 0.0% 0.0% | | <0.001 | 0.021 |
|  |  | 1 2 | | 4328 | fenfluramine |  | | 28 10 | 0.0% 0.0% | | <0.001 | 0.024 |
|  |  | 1 2 | | 47858 | methsuximide |  | | 10 10 | 0.0% 0.0% | | <0.001 | 0.012 |
|  |  | 1 2 | | 2054968 | stiripentol |  | | 10 10 | 0.0% 0.0% | | <0.001 | 0.012 |
|  |  | 1 2 | | CN302 | BENZODIAZEPINE DERIVATIVE SEDATIVES/HYPNOTICS |  | | 44,461 114,818 | 55.0% 25.1% | | <0.001 | 0.641 |
|  |  | 1 2 | | CN309 | SEDATIVES/HYPNOTICS,OTHER |  | | 9,289 21,871 | 11.5% 4.8% | | <0.001 | 0.247 |
|  |  | 1 2 | | CN301 | BARBITURIC ACID DERIVATIVE SEDATIVES/HYPNOTICS |  | | 3,352 8,224 | 4.1% 1.8% | | <0.001 | 0.139 |
|  |  | 1 2 | | CN101 | OPIOID ANALGESICS |  | | 23,978 90,311 | 29.7% 19.7% | | <0.001 | 0.232 |
|  |  | 1 2 | | HS051 | GLUCOCORTICOIDS |  | | 19,854 82,894 | 24.6% 18.1% | | <0.001 | 0.158 |
|  | **Laboratory** | | | | | | | | | | | |
|  |  | Cohort | | |  | Mean ± SD | | Patients | % of Cohort | | P-Value | SMD |
|  |  | 1 2 | | 9083 | BMI | 25.6 +/- 7.1 25.1 +/- 7.6 | | 42,944 177,245 | 53.1% 38.7% | | <0.001 | 0.066 |
|  |  | 1 2 | |  | 0 - 0 kg/m2 |  | | 43,018 177,708 | 53.2% 38.8% | | <0.001 | 0.291 |
| **Cohort 1 (N = 70,878) and cohort 2 (N = 70,878) characteristics after propensity score matching** | | | | | | | | | | | | |
|  | **Demographics** | | | | | | | | | | | |
|  |  | Cohort | | |  | Mean ± SD | | Patients | % of Cohort | | P-Value | SMD |
|  |  | 1 2 | | AI | Age at Index | 24.8 +/- 12.9 26.1 +/- 13.6 | | 70,878 70,878 | 100% 100% | | <0.001 | 0.095 |
|  |  | 1 2 | | 2106-3 | White |  | | 40,353 41,112 | 56.9% 58.0% | | <0.001 | 0.022 |
|  |  | 1 2 | | 1002-5 | American Indian or Alaska Native |  | | 260 312 | 0.4% 0.4% | | 0.029 | 0.012 |
|  |  | 1 2 | | UNK | Unknown Race |  | | 10,342 9,577 | 14.6% 13.5% | | <0.001 | 0.031 |
|  |  | 1 2 | | 2076-8 | Native Hawaiian or Other Pacific Islander |  | | 305 282 | 0.4% 0.4% | | 0.341 | 0.005 |
|  |  | 1 2 | | 2054-5 | Black or African American |  | | 13,101 13,000 | 18.5% 18.3% | | 0.489 | 0.004 |
|  |  | 1 2 | | 2131-1 | Other Race |  | | 3,498 3,553 | 4.9% 5.0% | | 0.502 | 0.004 |
|  |  | 1 2 | | 2028-9 | Asian |  | | 3,019 3,042 | 4.3% 4.3% | | 0.763 | 0.002 |
|  | **Diagnosis** | | | | | | | | | | | |
|  |  | Cohort | | |  | Mean ± SD | | Patients | % of Cohort | | P-Value | SMD |
|  |  | 1 2 | | F80-F89 | Pervasive and specific developmental disorders |  | | 9,664 9,293 | 13.6% 13.1% | | 0.004 | 0.015 |
|  |  | 1 2 | | F40-F48 | Anxiety, dissociative, stress-related, somatoform and other nonpsychotic mental disorders |  | | 18,897 20,110 | 26.7% 28.4% | | <0.001 | 0.038 |
|  |  | 1 2 | | F90-F98 | Behavioral and emotional disorders with onset usually occurring in childhood and adolescence |  | | 11,581 11,855 | 16.3% 16.7% | | 0.050 | 0.010 |
|  |  | 1 2 | | F20-F29 | Schizophrenia, schizotypal, delusional, and other non-mood psychotic disorders |  | | 10,404 10,200 | 14.7% 14.4% | | 0.124 | 0.008 |
|  |  | 1 2 | | F60-F69 | Disorders of adult personality and behavior |  | | 5,043 4,982 | 7.1% 7.0% | | 0.527 | 0.003 |
|  |  | 1 2 | | F70-F79 | Intellectual Disabilities |  | | 3,918 3,721 | 5.5% 5.2% | | 0.020 | 0.012 |
|  |  | 1 2 | | F01-F09 | Mental disorders due to known physiological conditions |  | | 2,945 2,912 | 4.2% 4.1% | | 0.660 | 0.002 |
|  |  | 1 2 | | F99-F99 | Unspecified mental disorder (F99) |  | | 1,732 1,736 | 2.4% 2.4% | | 0.945 | <0.001 |
|  |  | 1 2 | | E08-E13 | Diabetes mellitus |  | | 3,395 3,616 | 4.8% 5.1% | | 0.007 | 0.014 |
|  |  | 1 2 | | Q00-Q07 | Congenital malformations of the nervous system |  | | 2,017 1,986 | 2.8% 2.8% | | 0.619 | 0.003 |
|  |  | 1 2 | | Q60-Q64 | Congenital malformations of the urinary system |  | | 389 447 | 0.5% 0.6% | | 0.044 | 0.011 |
|  |  | 1 2 | | N45 | Orchitis and epididymitis |  | | 346 390 | 0.5% 0.6% | | 0.104 | 0.009 |
|  |  | 1 2 | | N43 | Hydrocele and spermatocele |  | | 425 421 | 0.6% 0.6% | | 0.890 | 0.001 |
|  |  | 1 2 | | N50.1 | Vascular disorders of male genital organs |  | | 23 23 | 0.0% 0.0% | | 1 | <0.001 |
|  |  | 1 2 | | N49 | Inflammatory disorders of male genital organs, not elsewhere classified |  | | 104 112 | 0.1% 0.2% | | 0.586 | 0.003 |
|  |  | 1 2 | | I86.1 | Scrotal varices |  | | 179 176 | 0.3% 0.2% | | 0.873 | 0.001 |
|  |  | 1 2 | | Q53 | Undescended and ectopic testicle |  | | 391 399 | 0.6% 0.6% | | 0.775 | 0.002 |
|  |  | 1 2 | | Q55 | Other congenital malformations of male genital organs |  | | 260 280 | 0.4% 0.4% | | 0.389 | 0.005 |
|  |  | 1 2 | | Q54 | Hypospadias |  | | 168 168 | 0.2% 0.2% | | 1 | <0.001 |
|  |  | 1 2 | | C60-C63 | Malignant neoplasms of male genital organs |  | | 79 99 | 0.1% 0.1% | | 0.134 | 0.008 |
|  |  | 1 2 | | N44.8 | Other noninflammatory disorders of the testis |  | | 184 187 | 0.3% 0.3% | | 0.876 | 0.001 |
|  |  | 1 2 | | N44.0 | Torsion of testis |  | | 50 51 | 0.1% 0.1% | | 0.921 | 0.001 |
|  |  | 1 2 | | N30-N39 | Other diseases of the urinary system |  | | 3,423 3,617 | 4.8% 5.1% | | 0.018 | 0.013 |
|  |  | 1 2 | | K40 | Inguinal hernia |  | | 676 729 | 1.0% 1.0% | | 0.155 | 0.008 |
|  |  | 1 2 | | E84 | Cystic fibrosis |  | | 60 60 | 0.1% 0.1% | | 1 | <0.001 |
|  |  | 1 2 | | S30-S39 | Injuries to the abdomen, lower back, lumbar spine, pelvis and external genitals |  | | 3,979 4,251 | 5.6% 6.0% | | 0.002 | 0.016 |
|  |  | 1 2 | | E23.0 | Hypopituitarism |  | | 280 270 | 0.4% 0.4% | | 0.669 | 0.002 |
|  |  | 1 2 | | R56 | Convulsions, not elsewhere classified |  | | 23,343 21,340 | 32.9% 30.1% | | <0.001 | 0.061 |
|  |  | 1 2 | | E34.5 | Androgen insensitivity syndrome |  | | 10 10 | 0.0% 0.0% | | 1 | <0.001 |
|  |  | 1 2 | | Q87.1 | Congenital malformation syndromes predominantly associated with short stature |  | | 62 54 | 0.1% 0.1% | | 0.457 | 0.004 |
|  |  | 1 2 | | F31 | Bipolar disorder |  | | 21,507 23,163 | 30.3% 32.7% | | <0.001 | 0.050 |
|  |  | 1 2 | | F32 | Depressive episode |  | | 11,651 12,573 | 16.4% 17.7% | | <0.001 | 0.035 |
|  |  | 1 2 | | F39 | Unspecified mood [affective] disorder |  | | 5,036 5,120 | 7.1% 7.2% | | 0.387 | 0.005 |
|  |  | 1 2 | | F33 | Major depressive disorder, recurrent |  | | 3,526 3,763 | 5.0% 5.3% | | 0.004 | 0.015 |
|  |  | 1 2 | | F34 | Persistent mood [affective] disorders |  | | 1,635 1,749 | 2.3% 2.5% | | 0.047 | 0.011 |
|  |  | 1 2 | | F30 | Manic episode |  | | 1,501 1,441 | 2.1% 2.0% | | 0.264 | 0.006 |
|  |  | 1 2 | | F50-F59 | Behavioral syndromes associated with physiological disturbances and physical factors |  | | 1,956 2,071 | 2.8% 2.9% | | 0.066 | 0.010 |
|  |  | 1 2 | | J00-J99 | Diseases of the respiratory system |  | | 21,862 22,197 | 30.8% 31.3% | | 0.055 | 0.010 |
|  |  | 1 2 | | I00-I99 | Diseases of the circulatory system |  | | 16,423 17,202 | 23.2% 24.3% | | <0.001 | 0.026 |
|  |  | 1 2 | | K00-K95 | Diseases of the digestive system |  | | 19,678 20,373 | 27.8% 28.7% | | <0.001 | 0.022 |
|  |  | 1 2 | | Q65-Q79 | Congenital malformations and deformations of the musculoskeletal system |  | | 1,955 1,908 | 2.8% 2.7% | | 0.443 | 0.004 |
|  |  | 1 2 | | N17-N19 | Acute kidney failure and chronic kidney disease |  | | 3,075 3,272 | 4.3% 4.6% | | 0.011 | 0.013 |
|  |  | 1 2 | | A50-A64 | Infections with a predominantly sexual mode of transmission |  | | 801 918 | 1.1% 1.3% | | 0.005 | 0.015 |
|  |  | 1 2 | | E03 | Other hypothyroidism |  | | 1,855 1,877 | 2.6% 2.6% | | 0.715 | 0.002 |
|  |  | 1 2 | | E05 | Thyrotoxicosis [hyperthyroidism] |  | | 279 282 | 0.4% 0.4% | | 0.899 | 0.001 |
|  |  | 1 2 | | E83.1 | Disorders of iron metabolism |  | | 70 81 | 0.1% 0.1% | | 0.370 | 0.005 |
|  |  | 1 2 | | Q99 | Other chromosome abnormalities, not elsewhere classified |  | | 621 596 | 0.9% 0.8% | | 0.472 | 0.004 |
|  |  | 1 2 | | Q93 | Monosomies and deletions from the autosomes, not elsewhere classified |  | | 312 299 | 0.4% 0.4% | | 0.598 | 0.003 |
|  |  | 1 2 | | Q90 | Down syndrome |  | | 250 257 | 0.4% 0.4% | | 0.755 | 0.002 |
|  |  | 1 2 | | Q92 | Other trisomies and partial trisomies of the autosomes, not elsewhere classified |  | | 109 112 | 0.2% 0.2% | | 0.840 | 0.001 |
|  |  | 1 2 | | Q98.4 | Klinefelter syndrome, unspecified |  | | 58 66 | 0.1% 0.1% | | 0.472 | 0.004 |
|  |  | 1 2 | | Q98.5 | Karyotype 47, XYY |  | | 22 23 | 0.0% 0.0% | | 0.881 | 0.001 |
|  |  | 1 2 | | Q98.7 | Male with sex chromosome mosaicism |  | | 20 20 | 0.0% 0.0% | | 1 | <0.001 |
|  |  | 1 2 | | Q98.8 | Other specified sex chromosome abnormalities, male phenotype |  | | 21 21 | 0.0% 0.0% | | 1 | <0.001 |
|  |  | 1 2 | | Q98.0 | Klinefelter syndrome karyotype 47, XXY |  | | 12 13 | 0.0% 0.0% | | 0.841 | 0.001 |
|  |  | 1 2 | | Q98.1 | Klinefelter syndrome, male with more than two X chromosomes |  | | 10 10 | 0.0% 0.0% | | 1 | <0.001 |
|  |  | 1 2 | | Q98.6 | Male with structurally abnormal sex chromosome |  | | 10 10 | 0.0% 0.0% | | 1 | <0.001 |
|  |  | 1 2 | | Q98.9 | Sex chromosome abnormality, male phenotype, unspecified |  | | 10 10 | 0.0% 0.0% | | 1 | <0.001 |
|  |  | 1 2 | | D35.2 | Benign neoplasm of pituitary gland |  | | 64 71 | 0.1% 0.1% | | 0.547 | 0.003 |
|  |  | 1 2 | | D35.3 | Benign neoplasm of craniopharyngeal duct |  | | 29 30 | 0.0% 0.0% | | 0.896 | 0.001 |
|  |  | 1 2 | | E40-E46 | Malnutrition |  | | 1,531 1,543 | 2.2% 2.2% | | 0.827 | 0.001 |
|  |  | 1 2 | | G40.9 | Epilepsy, unspecified |  | | 21,569 20,178 | 30.4% 28.5% | | <0.001 | 0.043 |
|  |  | 1 2 | | G40.3 | Generalized idiopathic epilepsy and epileptic syndromes |  | | 6,052 5,780 | 8.5% 8.2% | | 0.009 | 0.014 |
|  |  | 1 2 | | G40.4 | Other generalized epilepsy and epileptic syndromes |  | | 4,722 4,447 | 6.7% 6.3% | | 0.003 | 0.016 |
|  |  | 1 2 | | G40.2 | Localization-related (focal) (partial) symptomatic epilepsy and epileptic syndromes with complex partial seizures |  | | 4,670 4,001 | 6.6% 5.6% | | <0.001 | 0.039 |
|  |  | 1 2 | | G40.8 | Other epilepsy and recurrent seizures |  | | 3,696 3,465 | 5.2% 4.9% | | 0.005 | 0.015 |
|  |  | 1 2 | | G40.1 | Localization-related (focal) (partial) symptomatic epilepsy and epileptic syndromes with simple partial seizures |  | | 3,345 2,991 | 4.7% 4.2% | | <0.001 | 0.024 |
|  |  | 1 2 | | G40.A | Absence epileptic syndrome |  | | 1,845 1,727 | 2.6% 2.4% | | 0.046 | 0.011 |
|  |  | 1 2 | | G40.0 | Localization-related (focal) (partial) idiopathic epilepsy and epileptic syndromes with seizures of localized onset |  | | 1,057 914 | 1.5% 1.3% | | 0.001 | 0.017 |
|  |  | 1 2 | | G40.5 | Epileptic seizures related to external causes |  | | 604 625 | 0.9% 0.9% | | 0.547 | 0.003 |
|  |  | 1 2 | | G40.B | Juvenile myoclonic epilepsy [impulsive petit mal] |  | | 497 486 | 0.7% 0.7% | | 0.725 | 0.002 |
|  |  | 1 2 | | F17 | Nicotine dependence |  | | 13,323 14,174 | 18.8% 20.0% | | <0.001 | 0.030 |
|  |  | 1 2 | | F12 | Cannabis related disorders |  | | 8,636 8,916 | 12.2% 12.6% | | 0.024 | 0.012 |
|  |  | 1 2 | | F10 | Alcohol related disorders |  | | 7,233 7,859 | 10.2% 11.1% | | <0.001 | 0.029 |
|  |  | 1 2 | | F19 | Other psychoactive substance related disorders |  | | 5,991 6,308 | 8.5% 8.9% | | 0.003 | 0.016 |
|  |  | 1 2 | | F11 | Opioid related disorders |  | | 3,618 3,988 | 5.1% 5.6% | | <0.001 | 0.023 |
|  |  | 1 2 | | F14 | Cocaine related disorders |  | | 3,043 3,207 | 4.3% 4.5% | | 0.034 | 0.011 |
|  |  | 1 2 | | F15 | Other stimulant related disorders |  | | 2,971 3,089 | 4.2% 4.4% | | 0.121 | 0.008 |
|  |  | 1 2 | | F13 | Sedative, hypnotic, or anxiolytic related disorders |  | | 1,552 1,663 | 2.2% 2.3% | | 0.048 | 0.011 |
|  |  | 1 2 | | F18 | Inhalant related disorders |  | | 1,253 1,334 | 1.8% 1.9% | | 0.108 | 0.009 |
|  |  | 1 2 | | F16 | Hallucinogen related disorders |  | | 694 713 | 1.0% 1.0% | | 0.611 | 0.003 |
|  | **Procedure** | | | | | | | | | | | |
|  |  | Cohort | | |  | Mean ± SD | | Patients | % of Cohort | | P-Value | SMD |
|  |  | 1 2 | | 1010843 | Radiation Oncology Treatment |  | | 136 146 | 0.2% 0.2% | | 0.551 | 0.003 |
|  |  | 1 2 | | 1008061 | Surgical Procedures on the Urinary System |  | | 1,863 1,913 | 2.6% 2.7% | | 0.410 | 0.004 |
|  |  | 1 2 | | 1008011 | Repair initial inguinal hernia, age 5 years or older |  | | 88 97 | 0.1% 0.1% | | 0.508 | 0.004 |
|  |  | 1 2 | | 1008470 | Surgical Procedures on the Male Genital System |  | | 665 675 | 0.9% 1.0% | | 0.784 | 0.001 |
|  | **Medication** | | | | | | | | | | | |
|  |  | Cohort | | |  | Mean ± SD | | Patients | % of Cohort | | P-Value | SMD |
|  |  | 1 2 | | 25025 | finasteride |  | | 158 186 | 0.2% 0.3% | | 0.131 | 0.008 |
|  |  | 1 2 | | CN750 | LITHIUM SALTS |  | | 2,762 2,758 | 3.9% 3.9% | | 0.956 | <0.001 |
|  |  | 1 2 | | 6135 | ketoconazole |  | | 956 1,040 | 1.3% 1.5% | | 0.058 | 0.010 |
|  |  | 1 2 | | 10829 | trimethoprim |  | | 3,766 4,033 | 5.3% 5.7% | | 0.002 | 0.017 |
|  |  | 1 2 | | 7454 | nitrofurantoin |  | | 304 332 | 0.4% 0.5% | | 0.266 | 0.006 |
|  |  | 1 2 | | AM200 | ERYTHROMYCINS/MACROLIDES |  | | 5,701 6,076 | 8.0% 8.6% | | <0.001 | 0.019 |
|  |  | 1 2 | | AM300 | AMINOGLYCOSIDES |  | | 3,154 3,330 | 4.4% 4.7% | | 0.025 | 0.012 |
|  |  | 1 2 | | AN000 | ANTINEOPLASTICS |  | | 1,132 1,235 | 1.6% 1.7% | | 0.033 | 0.011 |
|  |  | 1 2 | | CN709 | ANTIPSYCHOTICS,OTHER |  | | 26,724 27,675 | 37.7% 39.0% | | <0.001 | 0.028 |
|  |  | 1 2 | | CN701 | PHENOTHIAZINE/RELATED ANTIPSYCHOTICS |  | | 1,620 1,436 | 2.3% 2.0% | | 0.001 | 0.018 |
|  |  | 1 2 | | CN609 | ANTIDEPRESSANTS,OTHER |  | | 19,952 21,439 | 28.1% 30.2% | | <0.001 | 0.046 |
|  |  | 1 2 | | CN601 | TRICYCLIC ANTIDEPRESSANTS |  | | 2,202 2,376 | 3.1% 3.4% | | 0.009 | 0.014 |
|  |  | 1 2 | | CV100 | BETA BLOCKERS/RELATED |  | | 6,868 7,243 | 9.7% 10.2% | | 0.001 | 0.018 |
|  |  | 1 2 | | CV200 | CALCIUM CHANNEL BLOCKERS |  | | 3,611 3,926 | 5.1% 5.5% | | <0.001 | 0.020 |
|  |  | 1 2 | | CV800 | ACE INHIBITORS |  | | 3,330 3,534 | 4.7% 5.0% | | 0.012 | 0.013 |
|  |  | 1 2 | | CV150 | ALPHA BLOCKERS/RELATED |  | | 2,482 2,613 | 3.5% 3.7% | | 0.062 | 0.010 |
|  |  | 1 2 | | CV490 | ANTIHYPERTENSIVES,OTHER |  | | 8,758 9,210 | 12.4% 13.0% | | <0.001 | 0.019 |
|  |  | 1 2 | | HS100 | ANDROGENS/ANABOLICS |  | | 414 455 | 0.6% 0.6% | | 0.163 | 0.007 |
|  |  | 1 2 | | HS800 | PROGESTINS |  | | 158 186 | 0.2% 0.3% | | 0.131 | 0.008 |
|  |  | 1 2 | | HS300 | ESTROGENS |  | | 118 128 | 0.2% 0.2% | | 0.523 | 0.003 |
|  |  | 1 2 | | 9997 | spironolactone |  | | 486 547 | 0.7% 0.8% | | 0.057 | 0.010 |
|  |  | 1 2 | | 3014 | cyproterone |  | | 10 10 | 0.0% 0.0% | | 1 | <0.001 |
|  |  | 1 2 | | 114477 | levetiracetam |  | | 14,522 14,753 | 20.5% 20.8% | | 0.130 | 0.008 |
|  |  | 1 2 | | 28439 | lamotrigine |  | | 5,361 5,805 | 7.6% 8.2% | | <0.001 | 0.023 |
|  |  | 1 2 | | 25480 | gabapentin |  | | 6,150 6,654 | 8.7% 9.4% | | <0.001 | 0.025 |
|  |  | 1 2 | | 38404 | topiramate |  | | 3,279 3,400 | 4.6% 4.8% | | 0.129 | 0.008 |
|  |  | 1 2 | | 32624 | oxcarbazepine |  | | 3,780 3,777 | 5.3% 5.3% | | 0.972 | <0.001 |
|  |  | 1 2 | | 623400 | lacosamide |  | | 2,221 2,216 | 3.1% 3.1% | | 0.939 | <0.001 |
|  |  | 1 2 | | 8183 | phenytoin |  | | 2,363 2,502 | 3.3% 3.5% | | 0.043 | 0.011 |
|  |  | 1 2 | | 21241 | clobazam |  | | 1,667 1,585 | 2.4% 2.2% | | 0.146 | 0.008 |
|  |  | 1 2 | | 2002 | carbamazepine |  | | 2,223 2,337 | 3.1% 3.3% | | 0.086 | 0.009 |
|  |  | 1 2 | | 39998 | zonisamide |  | | 1,523 1,528 | 2.1% 2.2% | | 0.927 | <0.001 |
|  |  | 1 2 | | 72236 | fosphenytoin |  | | 1,192 1,142 | 1.7% 1.6% | | 0.297 | 0.006 |
|  |  | 1 2 | | 4135 | ethosuximide |  | | 818 760 | 1.2% 1.1% | | 0.142 | 0.008 |
|  |  | 1 2 | | 69036 | rufinamide |  | | 262 244 | 0.4% 0.3% | | 0.423 | 0.004 |
|  |  | 1 2 | | 1739745 | brivaracetam |  | | 222 226 | 0.3% 0.3% | | 0.850 | 0.001 |
|  |  | 1 2 | | 14851 | vigabatrin |  | | 249 226 | 0.4% 0.3% | | 0.290 | 0.006 |
|  |  | 1 2 | | 24812 | felbamate |  | | 178 175 | 0.3% 0.2% | | 0.873 | 0.001 |
|  |  | 1 2 | | 1356552 | perampanel |  | | 188 206 | 0.3% 0.3% | | 0.364 | 0.005 |
|  |  | 1 2 | | 2045371 | cannabidiol |  | | 166 179 | 0.2% 0.3% | | 0.483 | 0.004 |
|  |  | 1 2 | | 1482502 | eslicarbazepine |  | | 114 117 | 0.2% 0.2% | | 0.843 | 0.001 |
|  |  | 1 2 | | 8691 | primidone |  | | 104 121 | 0.1% 0.2% | | 0.257 | 0.006 |
|  |  | 1 2 | | 2265690 | cenobamate |  | | 57 55 | 0.1% 0.1% | | 0.850 | 0.001 |
|  |  | 1 2 | | 31914 | tiagabine |  | | 28 26 | 0.0% 0.0% | | 0.785 | 0.001 |
|  |  | 1 2 | | 4328 | fenfluramine |  | | 10 10 | 0.0% 0.0% | | 1 | <0.001 |
|  |  | 1 2 | | 47858 | methsuximide |  | | 10 10 | 0.0% 0.0% | | 1 | <0.001 |
|  |  | 1 2 | | 2054968 | stiripentol |  | | 10 10 | 0.0% 0.0% | | 1 | <0.001 |
|  |  | 1 2 | | CN302 | BENZODIAZEPINE DERIVATIVE SEDATIVES/HYPNOTICS |  | | 35,835 37,027 | 50.6% 52.2% | | <0.001 | 0.034 |
|  |  | 1 2 | | CN309 | SEDATIVES/HYPNOTICS,OTHER |  | | 7,158 7,438 | 10.1% 10.5% | | 0.014 | 0.013 |
|  |  | 1 2 | | CN301 | BARBITURIC ACID DERIVATIVE SEDATIVES/HYPNOTICS |  | | 2,227 2,317 | 3.1% 3.3% | | 0.175 | 0.007 |
|  |  | 1 2 | | CN101 | OPIOID ANALGESICS |  | | 19,813 21,486 | 28.0% 30.3% | | <0.001 | 0.052 |
|  |  | 1 2 | | HS051 | GLUCOCORTICOIDS |  | | 16,013 16,847 | 22.6% 23.8% | | <0.001 | 0.028 |
|  | **Laboratory** | | | | | | | | | | | |
|  |  | Cohort | | |  | Mean ± SD | | Patients | % of Cohort | | P-Value | SMD |
|  |  | 1 2 | | 9083 | BMI | 25.8 +/- 7.1 26.1 +/- 7.3 | | 36,364 38,354 | 51.3% 54.1% | | <0.001 | 0.042 |
|  |  | 1 2 | |  | 0 - 0 kg/m2 |  | | 36,434 38,442 | 51.4% 54.2% | | <0.001 | 0.057 |

**Abbreviations:** BMI = Body Mass Index; SD = Standard deviation; SMD = Standardised mean difference

**N.B.:** Uncorrected p-values were calculated using two-sided t-tests for continuous covariates and two-sided Z-tests for categorical covariates. These p-values reflect descriptive comparisons of baseline characteristics during propensity score matching. Correction for multiple comparisons is not appropriate in this context, as the aim is to assess covariate balance (reflected by a standardised mean difference of <0.1) rather than to test hypotheses. Exact p-values beyond three decimal places are not provided in the propensity score matching outputs generated by the TriNetX platform. Where p-values fall below this threshold, they are reported as <0.001.

# Supplementary Table 9: Propensity score matching men with epilepsy or bipolar disorder exposed (cohort 1) and unexposed (cohort 2) to valproate – 5-year outcome assessment

Propensity score matching was performed on all listed characteristics. Characteristics of the cohorts before and after matching are summarized in the table below.

| **Cohort 1 and cohort 2 patient count before and after propensity score matching** | | | | | | | | | | | | |
| --- | --- | --- | --- | --- | --- | --- | --- | --- | --- | --- | --- | --- |
|  | | | Cohort | | | Patient count before matching | | | | Patient count after matching | | |
|  | | | 1 - Men with epilepsy or bipolar disorder exposed to valproate_v9 | | | 87,720 | | | | 75,233 | | |
|  | | | 2 - Men with epilepsy or bipolar disorder not exposed to valproate_v9 | | | 503,955 | | | | 75,233 | | |
| **Propensity score density function - Before and after matching (cohort 1 - purple, cohort 2 - green)** | | | | | | | | | | | | |
|  |  | | 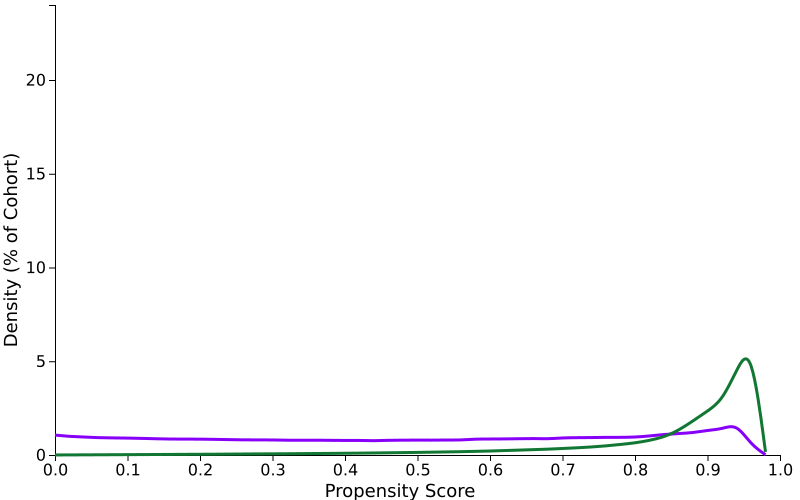 | | | | 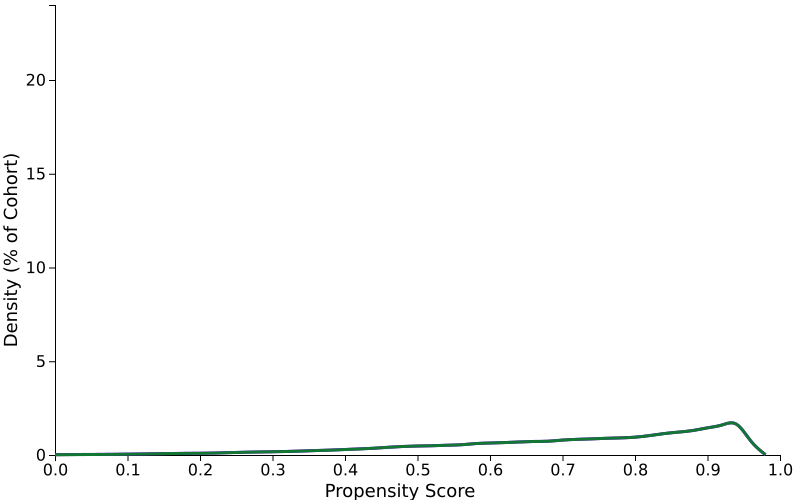 | | | | | |
| **Cohort 1 (N = 87,720) and cohort 2 (N = 503,955) characteristics before propensity score matching** | | | | | | | | | | | | |
|  | **Demographics** | | | | | | | | | | | |
|  |  | Cohort | | |  | Mean ± SD | | Patients | % of Cohort | | P-Value | SMD |
|  |  | 1 2 | | AI | Age at Index | 24.9 +/- 12.9 20.9 +/- 15.0 | | 85,213 482,973 | 100% 100% | | <0.001 | 0.283 |
|  |  | 1 2 | | 2106-3 | White |  | | 48,946 272,239 | 57.4% 56.4% | | <0.001 | 0.022 |
|  |  | 1 2 | | 1002-5 | American Indian or Alaska Native |  | | 345 1,694 | 0.4% 0.4% | | 0.015 | 0.009 |
|  |  | 1 2 | | UNK | Unknown Race |  | | 11,660 78,981 | 13.7% 16.4% | | <0.001 | 0.075 |
|  |  | 1 2 | | 2076-8 | Native Hawaiian or Other Pacific Islander |  | | 344 2,298 | 0.4% 0.5% | | 0.004 | 0.011 |
|  |  | 1 2 | | 2054-5 | Black or African American |  | | 15,598 81,915 | 18.3% 17.0% | | <0.001 | 0.035 |
|  |  | 1 2 | | 2131-1 | Other Race |  | | 4,714 30,016 | 5.5% 6.2% | | <0.001 | 0.029 |
|  |  | 1 2 | | 2028-9 | Asian |  | | 3,606 15,830 | 4.2% 3.3% | | <0.001 | 0.050 |
|  | **Diagnosis** | | | | | | | | | | | |
|  |  | Cohort | | |  | Mean ± SD | | Patients | % of Cohort | | P-Value | SMD |
|  |  | 1 2 | | F80-F89 | Pervasive and specific developmental disorders |  | | 13,824 44,978 | 16.2% 9.3% | | <0.001 | 0.208 |
|  |  | 1 2 | | F40-F48 | Anxiety, dissociative, stress-related, somatoform and other nonpsychotic mental disorders |  | | 23,781 82,865 | 27.9% 17.2% | | <0.001 | 0.259 |
|  |  | 1 2 | | F90-F98 | Behavioral and emotional disorders with onset usually occurring in childhood and adolescence |  | | 15,311 48,177 | 18.0% 10.0% | | <0.001 | 0.232 |
|  |  | 1 2 | | F20-F29 | Schizophrenia, schizotypal, delusional, and other non-mood psychotic disorders |  | | 13,470 25,271 | 15.8% 5.2% | | <0.001 | 0.350 |
|  |  | 1 2 | | F60-F69 | Disorders of adult personality and behavior |  | | 7,134 13,013 | 8.4% 2.7% | | <0.001 | 0.250 |
|  |  | 1 2 | | F70-F79 | Intellectual Disabilities |  | | 6,359 9,256 | 7.5% 1.9% | | <0.001 | 0.265 |
|  |  | 1 2 | | F01-F09 | Mental disorders due to known physiological conditions |  | | 4,519 8,104 | 5.3% 1.7% | | <0.001 | 0.198 |
|  |  | 1 2 | | F99-F99 | Unspecified mental disorder (F99) |  | | 2,466 4,670 | 2.9% 1.0% | | <0.001 | 0.140 |
|  |  | 1 2 | | E08-E13 | Diabetes mellitus |  | | 4,224 17,423 | 5.0% 3.6% | | <0.001 | 0.067 |
|  |  | 1 2 | | Q00-Q07 | Congenital malformations of the nervous system |  | | 3,152 9,682 | 3.7% 2.0% | | <0.001 | 0.102 |
|  |  | 1 2 | | Q60-Q64 | Congenital malformations of the urinary system |  | | 539 2,881 | 0.6% 0.6% | | 0.210 | 0.005 |
|  |  | 1 2 | | N45 | Orchitis and epididymitis |  | | 452 1,756 | 0.5% 0.4% | | <0.001 | 0.025 |
|  |  | 1 2 | | N43 | Hydrocele and spermatocele |  | | 547 2,688 | 0.6% 0.6% | | 0.002 | 0.011 |
|  |  | 1 2 | | N50.1 | Vascular disorders of male genital organs |  | | 43 145 | 0.1% 0.0% | | 0.002 | 0.010 |
|  |  | 1 2 | | N49 | Inflammatory disorders of male genital organs, not elsewhere classified |  | | 134 529 | 0.2% 0.1% | | <0.001 | 0.013 |
|  |  | 1 2 | | I86.1 | Scrotal varices |  | | 231 1,009 | 0.3% 0.2% | | <0.001 | 0.013 |
|  |  | 1 2 | | Q53 | Undescended and ectopic testicle |  | | 564 2,748 | 0.7% 0.6% | | 0.001 | 0.012 |
|  |  | 1 2 | | Q55 | Other congenital malformations of male genital organs |  | | 384 2,490 | 0.5% 0.5% | | 0.014 | 0.009 |
|  |  | 1 2 | | Q54 | Hypospadias |  | | 215 1,487 | 0.3% 0.3% | | 0.006 | 0.011 |
|  |  | 1 2 | | C60-C63 | Malignant neoplasms of male genital organs |  | | 93 560 | 0.1% 0.1% | | 0.589 | 0.002 |
|  |  | 1 2 | | N44.8 | Other noninflammatory disorders of the testis |  | | 250 709 | 0.3% 0.1% | | <0.001 | 0.031 |
|  |  | 1 2 | | N44.0 | Torsion of testis |  | | 58 300 | 0.1% 0.1% | | 0.523 | 0.002 |
|  |  | 1 2 | | N30-N39 | Other diseases of the urinary system |  | | 4,672 15,696 | 5.5% 3.2% | | <0.001 | 0.109 |
|  |  | 1 2 | | K40 | Inguinal hernia |  | | 875 4,822 | 1.0% 1.0% | | 0.442 | 0.003 |
|  |  | 1 2 | | E84 | Cystic fibrosis |  | | 70 335 | 0.1% 0.1% | | 0.197 | 0.005 |
|  |  | 1 2 | | S30-S39 | Injuries to the abdomen, lower back, lumbar spine, pelvis and external genitals |  | | 5,087 18,228 | 6.0% 3.8% | | <0.001 | 0.102 |
|  |  | 1 2 | | E23.0 | Hypopituitarism |  | | 379 1,458 | 0.4% 0.3% | | <0.001 | 0.023 |
|  |  | 1 2 | | R56 | Convulsions, not elsewhere classified |  | | 31,306 238,576 | 36.7% 49.4% | | <0.001 | 0.258 |
|  |  | 1 2 | | E34.5 | Androgen insensitivity syndrome |  | | 10 15 | 0.0% 0.0% | | <0.001 | 0.010 |
|  |  | 1 2 | | Q87.1 | Congenital malformation syndromes predominantly associated with short stature |  | | 90 350 | 0.1% 0.1% | | 0.001 | 0.011 |
|  |  | 1 2 | | F31 | Bipolar disorder |  | | 24,484 115,324 | 28.7% 23.9% | | <0.001 | 0.110 |
|  |  | 1 2 | | F32 | Depressive episode |  | | 14,575 47,893 | 17.1% 9.9% | | <0.001 | 0.211 |
|  |  | 1 2 | | F39 | Unspecified mood [affective] disorder |  | | 6,773 12,186 | 7.9% 2.5% | | <0.001 | 0.245 |
|  |  | 1 2 | | F33 | Major depressive disorder, recurrent |  | | 4,301 13,294 | 5.0% 2.8% | | <0.001 | 0.119 |
|  |  | 1 2 | | F34 | Persistent mood [affective] disorders |  | | 2,120 5,088 | 2.5% 1.1% | | <0.001 | 0.109 |
|  |  | 1 2 | | F30 | Manic episode |  | | 2,201 2,679 | 2.6% 0.6% | | <0.001 | 0.164 |
|  |  | 1 2 | | F50-F59 | Behavioral syndromes associated with physiological disturbances and physical factors |  | | 2,679 8,015 | 3.1% 1.7% | | <0.001 | 0.097 |
|  |  | 1 2 | | J00-J99 | Diseases of the respiratory system |  | | 28,223 141,370 | 33.1% 29.3% | | <0.001 | 0.083 |
|  |  | 1 2 | | I00-I99 | Diseases of the circulatory system |  | | 21,339 89,612 | 25.0% 18.6% | | <0.001 | 0.158 |
|  |  | 1 2 | | K00-K95 | Diseases of the digestive system |  | | 25,417 114,753 | 29.8% 23.8% | | <0.001 | 0.137 |
|  |  | 1 2 | | Q65-Q79 | Congenital malformations and deformations of the musculoskeletal system |  | | 2,835 14,656 | 3.3% 3.0% | | <0.001 | 0.017 |
|  |  | 1 2 | | N17-N19 | Acute kidney failure and chronic kidney disease |  | | 4,142 16,687 | 4.9% 3.5% | | <0.001 | 0.070 |
|  |  | 1 2 | | A50-A64 | Infections with a predominantly sexual mode of transmission |  | | 974 4,012 | 1.1% 0.8% | | <0.001 | 0.032 |
|  |  | 1 2 | | E03 | Other hypothyroidism |  | | 2,563 7,606 | 3.0% 1.6% | | <0.001 | 0.096 |
|  |  | 1 2 | | E05 | Thyrotoxicosis [hyperthyroidism] |  | | 366 1,066 | 0.4% 0.2% | | <0.001 | 0.037 |
|  |  | 1 2 | | E83.1 | Disorders of iron metabolism |  | | 98 547 | 0.1% 0.1% | | 0.889 | 0.001 |
|  |  | 1 2 | | Q99 | Other chromosome abnormalities, not elsewhere classified |  | | 1,048 2,813 | 1.2% 0.6% | | <0.001 | 0.068 |
|  |  | 1 2 | | Q93 | Monosomies and deletions from the autosomes, not elsewhere classified |  | | 479 1,555 | 0.6% 0.3% | | <0.001 | 0.036 |
|  |  | 1 2 | | Q90 | Down syndrome |  | | 325 1,699 | 0.4% 0.4% | | 0.181 | 0.005 |
|  |  | 1 2 | | Q92 | Other trisomies and partial trisomies of the autosomes, not elsewhere classified |  | | 191 528 | 0.2% 0.1% | | <0.001 | 0.028 |
|  |  | 1 2 | | Q98.4 | Klinefelter syndrome, unspecified |  | | 81 218 | 0.1% 0.0% | | <0.001 | 0.019 |
|  |  | 1 2 | | Q98.5 | Karyotype 47, XYY |  | | 31 94 | 0.0% 0.0% | | 0.002 | 0.010 |
|  |  | 1 2 | | Q98.7 | Male with sex chromosome mosaicism |  | | 31 59 | 0.0% 0.0% | | <0.001 | 0.016 |
|  |  | 1 2 | | Q98.8 | Other specified sex chromosome abnormalities, male phenotype |  | | 31 67 | 0.0% 0.0% | | <0.001 | 0.014 |
|  |  | 1 2 | | Q98.0 | Klinefelter syndrome karyotype 47, XXY |  | | 17 49 | 0.0% 0.0% | | 0.014 | 0.008 |
|  |  | 1 2 | | Q98.1 | Klinefelter syndrome, male with more than two X chromosomes |  | | 10 15 | 0.0% 0.0% | | <0.001 | 0.010 |
|  |  | 1 2 | | Q98.6 | Male with structurally abnormal sex chromosome |  | | 10 10 | 0.0% 0.0% | | <0.001 | 0.012 |
|  |  | 1 2 | | Q98.9 | Sex chromosome abnormality, male phenotype, unspecified |  | | 10 10 | 0.0% 0.0% | | <0.001 | 0.012 |
|  |  | 1 2 | | D35.2 | Benign neoplasm of pituitary gland |  | | 97 371 | 0.1% 0.1% | | 0.001 | 0.012 |
|  |  | 1 2 | | D35.3 | Benign neoplasm of craniopharyngeal duct |  | | 40 148 | 0.0% 0.0% | | 0.016 | 0.008 |
|  |  | 1 2 | | E40-E46 | Malnutrition |  | | 2,190 7,433 | 2.6% 1.5% | | <0.001 | 0.073 |
|  |  | 1 2 | | G40.9 | Epilepsy, unspecified |  | | 30,017 94,298 | 35.2% 19.5% | | <0.001 | 0.358 |
|  |  | 1 2 | | G40.3 | Generalized idiopathic epilepsy and epileptic syndromes |  | | 10,420 14,522 | 12.2% 3.0% | | <0.001 | 0.353 |
|  |  | 1 2 | | G40.4 | Other generalized epilepsy and epileptic syndromes |  | | 8,699 12,719 | 10.2% 2.6% | | <0.001 | 0.313 |
|  |  | 1 2 | | G40.2 | Localization-related (focal) (partial) symptomatic epilepsy and epileptic syndromes with complex partial seizures |  | | 7,854 18,510 | 9.2% 3.8% | | <0.001 | 0.219 |
|  |  | 1 2 | | G40.8 | Other epilepsy and recurrent seizures |  | | 6,427 16,966 | 7.5% 3.5% | | <0.001 | 0.177 |
|  |  | 1 2 | | G40.1 | Localization-related (focal) (partial) symptomatic epilepsy and epileptic syndromes with simple partial seizures |  | | 6,148 14,213 | 7.2% 2.9% | | <0.001 | 0.195 |
|  |  | 1 2 | | G40.A | Absence epileptic syndrome |  | | 3,187 4,973 | 3.7% 1.0% | | <0.001 | 0.178 |
|  |  | 1 2 | | G40.0 | Localization-related (focal) (partial) idiopathic epilepsy and epileptic syndromes with seizures of localized onset |  | | 2,032 4,610 | 2.4% 1.0% | | <0.001 | 0.112 |
|  |  | 1 2 | | G40.5 | Epileptic seizures related to external causes |  | | 1,219 2,586 | 1.4% 0.5% | | <0.001 | 0.091 |
|  |  | 1 2 | | G40.B | Juvenile myoclonic epilepsy [impulsive petit mal] |  | | 887 704 | 1.0% 0.1% | | <0.001 | 0.117 |
|  |  | 1 2 | | F17 | Nicotine dependence |  | | 16,381 61,494 | 19.2% 12.7% | | <0.001 | 0.178 |
|  |  | 1 2 | | F12 | Cannabis related disorders |  | | 10,895 27,936 | 12.8% 5.8% | | <0.001 | 0.243 |
|  |  | 1 2 | | F10 | Alcohol related disorders |  | | 8,654 35,834 | 10.2% 7.4% | | <0.001 | 0.097 |
|  |  | 1 2 | | F19 | Other psychoactive substance related disorders |  | | 7,398 21,340 | 8.7% 4.4% | | <0.001 | 0.173 |
|  |  | 1 2 | | F11 | Opioid related disorders |  | | 4,284 15,649 | 5.0% 3.2% | | <0.001 | 0.090 |
|  |  | 1 2 | | F14 | Cocaine related disorders |  | | 3,691 9,952 | 4.3% 2.1% | | <0.001 | 0.129 |
|  |  | 1 2 | | F15 | Other stimulant related disorders |  | | 3,693 10,187 | 4.3% 2.1% | | <0.001 | 0.126 |
|  |  | 1 2 | | F13 | Sedative, hypnotic, or anxiolytic related disorders |  | | 1,925 5,238 | 2.3% 1.1% | | <0.001 | 0.092 |
|  |  | 1 2 | | F18 | Inhalant related disorders |  | | 1,595 4,338 | 1.9% 0.9% | | <0.001 | 0.083 |
|  |  | 1 2 | | F16 | Hallucinogen related disorders |  | | 878 1,920 | 1.0% 0.4% | | <0.001 | 0.075 |
|  | **Procedure** | | | | | | | | | | | |
|  |  | Cohort | | |  | Mean ± SD | | Patients | % of Cohort | | P-Value | SMD |
|  |  | 1 2 | | 1010843 | Radiation Oncology Treatment |  | | 181 771 | 0.2% 0.2% | | 0.001 | 0.012 |
|  |  | 1 2 | | 1008061 | Surgical Procedures on the Urinary System |  | | 2,584 8,953 | 3.0% 1.9% | | <0.001 | 0.076 |
|  |  | 1 2 | | 1008011 | Repair initial inguinal hernia, age 5 years or older |  | | 124 445 | 0.1% 0.1% | | <0.001 | 0.015 |
|  |  | 1 2 | | 1008470 | Surgical Procedures on the Male Genital System |  | | 891 5,245 | 1.0% 1.1% | | 0.293 | 0.004 |
|  | **Medication** | | | | | | | | | | | |
|  |  | Cohort | | |  | Mean ± SD | | Patients | % of Cohort | | P-Value | SMD |
|  |  | 1 2 | | 25025 | finasteride |  | | 197 839 | 0.2% 0.2% | | <0.001 | 0.013 |
|  |  | 1 2 | | CN750 | LITHIUM SALTS |  | | 3,806 7,235 | 4.5% 1.5% | | <0.001 | 0.175 |
|  |  | 1 2 | | 6135 | ketoconazole |  | | 1,309 4,762 | 1.5% 1.0% | | <0.001 | 0.049 |
|  |  | 1 2 | | 10829 | trimethoprim |  | | 5,009 19,312 | 5.9% 4.0% | | <0.001 | 0.087 |
|  |  | 1 2 | | 7454 | nitrofurantoin |  | | 422 1,159 | 0.5% 0.2% | | <0.001 | 0.042 |
|  |  | 1 2 | | AM200 | ERYTHROMYCINS/MACROLIDES |  | | 7,660 29,292 | 9.0% 6.1% | | <0.001 | 0.111 |
|  |  | 1 2 | | AM300 | AMINOGLYCOSIDES |  | | 4,301 14,125 | 5.0% 2.9% | | <0.001 | 0.109 |
|  |  | 1 2 | | AN000 | ANTINEOPLASTICS |  | | 1,483 6,373 | 1.7% 1.3% | | <0.001 | 0.034 |
|  |  | 1 2 | | CN709 | ANTIPSYCHOTICS,OTHER |  | | 33,866 57,545 | 39.7% 11.9% | | <0.001 | 0.671 |
|  |  | 1 2 | | CN701 | PHENOTHIAZINE/RELATED ANTIPSYCHOTICS |  | | 2,744 2,075 | 3.2% 0.4% | | <0.001 | 0.210 |
|  |  | 1 2 | | CN609 | ANTIDEPRESSANTS,OTHER |  | | 25,570 60,865 | 30.0% 12.6% | | <0.001 | 0.435 |
|  |  | 1 2 | | CN601 | TRICYCLIC ANTIDEPRESSANTS |  | | 2,931 6,955 | 3.4% 1.4% | | <0.001 | 0.130 |
|  |  | 1 2 | | CV100 | BETA BLOCKERS/RELATED |  | | 9,414 25,352 | 11.0% 5.2% | | <0.001 | 0.213 |
|  |  | 1 2 | | CV200 | CALCIUM CHANNEL BLOCKERS |  | | 4,718 14,579 | 5.5% 3.0% | | <0.001 | 0.125 |
|  |  | 1 2 | | CV800 | ACE INHIBITORS |  | | 4,298 14,048 | 5.0% 2.9% | | <0.001 | 0.109 |
|  |  | 1 2 | | CV150 | ALPHA BLOCKERS/RELATED |  | | 3,417 7,425 | 4.0% 1.5% | | <0.001 | 0.151 |
|  |  | 1 2 | | CV490 | ANTIHYPERTENSIVES,OTHER |  | | 12,048 29,396 | 14.1% 6.1% | | <0.001 | 0.269 |
|  |  | 1 2 | | HS100 | ANDROGENS/ANABOLICS |  | | 542 2,296 | 0.6% 0.5% | | <0.001 | 0.022 |
|  |  | 1 2 | | HS800 | PROGESTINS |  | | 250 776 | 0.3% 0.2% | | <0.001 | 0.028 |
|  |  | 1 2 | | HS300 | ESTROGENS |  | | 160 726 | 0.2% 0.2% | | 0.011 | 0.009 |
|  |  | 1 2 | | 9997 | spironolactone |  | | 600 3,261 | 0.7% 0.7% | | 0.343 | 0.003 |
|  |  | 1 2 | | 3014 | cyproterone |  | | 10 10 | 0.0% 0.0% | | <0.001 | 0.012 |
|  |  | 1 2 | | 114477 | levetiracetam |  | | 20,438 60,678 | 24.0% 12.6% | | <0.001 | 0.299 |
|  |  | 1 2 | | 28439 | lamotrigine |  | | 7,768 21,733 | 9.1% 4.5% | | <0.001 | 0.184 |
|  |  | 1 2 | | 25480 | gabapentin |  | | 8,009 21,807 | 9.4% 4.5% | | <0.001 | 0.193 |
|  |  | 1 2 | | 38404 | topiramate |  | | 5,426 7,579 | 6.4% 1.6% | | <0.001 | 0.248 |
|  |  | 1 2 | | 32624 | oxcarbazepine |  | | 5,675 11,565 | 6.7% 2.4% | | <0.001 | 0.206 |
|  |  | 1 2 | | 623400 | lacosamide |  | | 4,131 4,982 | 4.8% 1.0% | | <0.001 | 0.227 |
|  |  | 1 2 | | 8183 | phenytoin |  | | 3,514 7,798 | 4.1% 1.6% | | <0.001 | 0.151 |
|  |  | 1 2 | | 21241 | clobazam |  | | 3,438 2,521 | 4.0% 0.5% | | <0.001 | 0.237 |
|  |  | 1 2 | | 2002 | carbamazepine |  | | 3,206 7,455 | 3.8% 1.5% | | <0.001 | 0.138 |
|  |  | 1 2 | | 39998 | zonisamide |  | | 2,949 3,374 | 3.5% 0.7% | | <0.001 | 0.194 |
|  |  | 1 2 | | 72236 | fosphenytoin |  | | 2,282 3,665 | 2.7% 0.8% | | <0.001 | 0.148 |
|  |  | 1 2 | | 4135 | ethosuximide |  | | 1,353 1,281 | 1.6% 0.3% | | <0.001 | 0.138 |
|  |  | 1 2 | | 69036 | rufinamide |  | | 603 352 | 0.7% 0.1% | | <0.001 | 0.102 |
|  |  | 1 2 | | 1739745 | brivaracetam |  | | 484 505 | 0.6% 0.1% | | <0.001 | 0.080 |
|  |  | 1 2 | | 14851 | vigabatrin |  | | 608 302 | 0.7% 0.1% | | <0.001 | 0.105 |
|  |  | 1 2 | | 24812 | felbamate |  | | 352 267 | 0.4% 0.1% | | <0.001 | 0.074 |
|  |  | 1 2 | | 1356552 | perampanel |  | | 424 363 | 0.5% 0.1% | | <0.001 | 0.079 |
|  |  | 1 2 | | 2045371 | cannabidiol |  | | 417 320 | 0.5% 0.1% | | <0.001 | 0.080 |
|  |  | 1 2 | | 1482502 | eslicarbazepine |  | | 201 361 | 0.2% 0.1% | | <0.001 | 0.041 |
|  |  | 1 2 | | 8691 | primidone |  | | 159 287 | 0.2% 0.1% | | <0.001 | 0.036 |
|  |  | 1 2 | | 2265690 | cenobamate |  | | 112 189 | 0.1% 0.0% | | <0.001 | 0.032 |
|  |  | 1 2 | | 31914 | tiagabine |  | | 44 58 | 0.1% 0.0% | | <0.001 | 0.022 |
|  |  | 1 2 | | 4328 | fenfluramine |  | | 32 12 | 0.0% 0.0% | | <0.001 | 0.025 |
|  |  | 1 2 | | 47858 | methsuximide |  | | 10 10 | 0.0% 0.0% | | <0.001 | 0.012 |
|  |  | 1 2 | | 2054968 | stiripentol |  | | 13 10 | 0.0% 0.0% | | <0.001 | 0.014 |
|  |  | 1 2 | | CN302 | BENZODIAZEPINE DERIVATIVE SEDATIVES/HYPNOTICS |  | | 47,107 125,545 | 55.3% 26.0% | | <0.001 | 0.625 |
|  |  | 1 2 | | CN309 | SEDATIVES/HYPNOTICS,OTHER |  | | 10,049 24,220 | 11.8% 5.0% | | <0.001 | 0.246 |
|  |  | 1 2 | | CN301 | BARBITURIC ACID DERIVATIVE SEDATIVES/HYPNOTICS |  | | 3,451 8,529 | 4.0% 1.8% | | <0.001 | 0.136 |
|  |  | 1 2 | | CN101 | OPIOID ANALGESICS |  | | 25,958 100,087 | 30.5% 20.7% | | <0.001 | 0.225 |
|  |  | 1 2 | | HS051 | GLUCOCORTICOIDS |  | | 21,578 91,490 | 25.3% 18.9% | | <0.001 | 0.154 |
|  | **Laboratory** | | | | | | | | | | | |
|  |  | Cohort | | |  | Mean ± SD | | Patients | % of Cohort | | P-Value | SMD |
|  |  | 1 2 | | 9083 | BMI | 25.7 +/- 7.1 25.3 +/- 7.5 | | 46,017 194,896 | 54.0% 40.4% | | <0.001 | 0.052 |
|  |  | 1 2 | |  | 0 - 0 kg/m2 |  | | 46,093 195,366 | 54.1% 40.5% | | <0.001 | 0.276 |
| **Cohort 1 (N = 75,233) and cohort 2 (N = 75,233) characteristics after propensity score matching** | | | | | | | | | | | | |
|  | **Demographics** | | | | | | | | | | | |
|  |  | Cohort | | |  | Mean ± SD | | Patients | % of Cohort | | P-Value | SMD |
|  |  | 1 2 | | AI | Age at Index | 25.1 +/- 12.9 26.2 +/- 13.5 | | 75,233 75,233 | 100% 100% | | <0.001 | 0.085 |
|  |  | 1 2 | | 2106-3 | White |  | | 43,128 44,020 | 57.3% 58.5% | | <0.001 | 0.024 |
|  |  | 1 2 | | 1002-5 | American Indian or Alaska Native |  | | 290 315 | 0.4% 0.4% | | 0.308 | 0.005 |
|  |  | 1 2 | | UNK | Unknown Race |  | | 10,583 9,900 | 14.1% 13.2% | | <0.001 | 0.026 |
|  |  | 1 2 | | 2076-8 | Native Hawaiian or Other Pacific Islander |  | | 309 298 | 0.4% 0.4% | | 0.655 | 0.002 |
|  |  | 1 2 | | 2054-5 | Black or African American |  | | 13,573 13,458 | 18.0% 17.9% | | 0.440 | 0.004 |
|  |  | 1 2 | | 2131-1 | Other Race |  | | 4,218 4,234 | 5.6% 5.6% | | 0.858 | 0.001 |
|  |  | 1 2 | | 2028-9 | Asian |  | | 3,132 3,008 | 4.2% 4.0% | | 0.106 | 0.008 |
|  | **Diagnosis** | | | | | | | | | | | |
|  |  | Cohort | | |  | Mean ± SD | | Patients | % of Cohort | | P-Value | SMD |
|  |  | 1 2 | | F80-F89 | Pervasive and specific developmental disorders |  | | 10,070 9,773 | 13.4% 13.0% | | 0.024 | 0.012 |
|  |  | 1 2 | | F40-F48 | Anxiety, dissociative, stress-related, somatoform and other nonpsychotic mental disorders |  | | 20,115 21,203 | 26.7% 28.2% | | <0.001 | 0.032 |
|  |  | 1 2 | | F90-F98 | Behavioral and emotional disorders with onset usually occurring in childhood and adolescence |  | | 12,065 12,530 | 16.0% 16.7% | | 0.001 | 0.017 |
|  |  | 1 2 | | F20-F29 | Schizophrenia, schizotypal, delusional, and other non-mood psychotic disorders |  | | 10,993 10,728 | 14.6% 14.3% | | 0.052 | 0.010 |
|  |  | 1 2 | | F60-F69 | Disorders of adult personality and behavior |  | | 5,220 5,145 | 6.9% 6.8% | | 0.445 | 0.004 |
|  |  | 1 2 | | F70-F79 | Intellectual Disabilities |  | | 4,049 3,873 | 5.4% 5.1% | | 0.042 | 0.010 |
|  |  | 1 2 | | F01-F09 | Mental disorders due to known physiological conditions |  | | 3,129 3,075 | 4.2% 4.1% | | 0.484 | 0.004 |
|  |  | 1 2 | | F99-F99 | Unspecified mental disorder (F99) |  | | 1,847 1,817 | 2.5% 2.4% | | 0.616 | 0.003 |
|  |  | 1 2 | | E08-E13 | Diabetes mellitus |  | | 3,655 3,879 | 4.9% 5.2% | | 0.008 | 0.014 |
|  |  | 1 2 | | Q00-Q07 | Congenital malformations of the nervous system |  | | 2,058 2,016 | 2.7% 2.7% | | 0.505 | 0.003 |
|  |  | 1 2 | | Q60-Q64 | Congenital malformations of the urinary system |  | | 418 430 | 0.6% 0.6% | | 0.679 | 0.002 |
|  |  | 1 2 | | N45 | Orchitis and epididymitis |  | | 385 400 | 0.5% 0.5% | | 0.591 | 0.003 |
|  |  | 1 2 | | N43 | Hydrocele and spermatocele |  | | 435 470 | 0.6% 0.6% | | 0.243 | 0.006 |
|  |  | 1 2 | | N50.1 | Vascular disorders of male genital organs |  | | 34 26 | 0.0% 0.0% | | 0.302 | 0.005 |
|  |  | 1 2 | | N49 | Inflammatory disorders of male genital organs, not elsewhere classified |  | | 114 122 | 0.2% 0.2% | | 0.602 | 0.003 |
|  |  | 1 2 | | I86.1 | Scrotal varices |  | | 199 206 | 0.3% 0.3% | | 0.728 | 0.002 |
|  |  | 1 2 | | Q53 | Undescended and ectopic testicle |  | | 391 382 | 0.5% 0.5% | | 0.746 | 0.002 |
|  |  | 1 2 | | Q55 | Other congenital malformations of male genital organs |  | | 278 269 | 0.4% 0.4% | | 0.700 | 0.002 |
|  |  | 1 2 | | Q54 | Hypospadias |  | | 172 150 | 0.2% 0.2% | | 0.220 | 0.006 |
|  |  | 1 2 | | C60-C63 | Malignant neoplasms of male genital organs |  | | 87 93 | 0.1% 0.1% | | 0.655 | 0.002 |
|  |  | 1 2 | | N44.8 | Other noninflammatory disorders of the testis |  | | 200 217 | 0.3% 0.3% | | 0.404 | 0.004 |
|  |  | 1 2 | | N44.0 | Torsion of testis |  | | 52 55 | 0.1% 0.1% | | 0.772 | 0.001 |
|  |  | 1 2 | | N30-N39 | Other diseases of the urinary system |  | | 3,633 3,752 | 4.8% 5.0% | | 0.156 | 0.007 |
|  |  | 1 2 | | K40 | Inguinal hernia |  | | 708 728 | 0.9% 1.0% | | 0.596 | 0.003 |
|  |  | 1 2 | | E84 | Cystic fibrosis |  | | 61 65 | 0.1% 0.1% | | 0.721 | 0.002 |
|  |  | 1 2 | | S30-S39 | Injuries to the abdomen, lower back, lumbar spine, pelvis and external genitals |  | | 4,219 4,358 | 5.6% 5.8% | | 0.122 | 0.008 |
|  |  | 1 2 | | E23.0 | Hypopituitarism |  | | 290 311 | 0.4% 0.4% | | 0.391 | 0.004 |
|  |  | 1 2 | | R56 | Convulsions, not elsewhere classified |  | | 25,019 23,194 | 33.3% 30.8% | | <0.001 | 0.052 |
|  |  | 1 2 | | E34.5 | Androgen insensitivity syndrome |  | | 10 10 | 0.0% 0.0% | | 1 | <0.001 |
|  |  | 1 2 | | Q87.1 | Congenital malformation syndromes predominantly associated with short stature |  | | 68 66 | 0.1% 0.1% | | 0.863 | 0.001 |
|  |  | 1 2 | | F31 | Bipolar disorder |  | | 22,517 24,119 | 29.9% 32.1% | | <0.001 | 0.046 |
|  |  | 1 2 | | F32 | Depressive episode |  | | 12,399 13,209 | 16.5% 17.6% | | <0.001 | 0.029 |
|  |  | 1 2 | | F39 | Unspecified mood [affective] disorder |  | | 5,296 5,368 | 7.0% 7.1% | | 0.469 | 0.004 |
|  |  | 1 2 | | F33 | Major depressive disorder, recurrent |  | | 3,734 3,967 | 5.0% 5.3% | | 0.006 | 0.014 |
|  |  | 1 2 | | F34 | Persistent mood [affective] disorders |  | | 1,699 1,745 | 2.3% 2.3% | | 0.428 | 0.004 |
|  |  | 1 2 | | F30 | Manic episode |  | | 1,641 1,539 | 2.2% 2.0% | | 0.068 | 0.009 |
|  |  | 1 2 | | F50-F59 | Behavioral syndromes associated with physiological disturbances and physical factors |  | | 2,095 2,155 | 2.8% 2.9% | | 0.350 | 0.005 |
|  |  | 1 2 | | J00-J99 | Diseases of the respiratory system |  | | 23,256 23,373 | 30.9% 31.1% | | 0.514 | 0.003 |
|  |  | 1 2 | | I00-I99 | Diseases of the circulatory system |  | | 17,705 18,375 | 23.5% 24.4% | | <0.001 | 0.021 |
|  |  | 1 2 | | K00-K95 | Diseases of the digestive system |  | | 20,856 21,326 | 27.7% 28.3% | | 0.007 | 0.014 |
|  |  | 1 2 | | Q65-Q79 | Congenital malformations and deformations of the musculoskeletal system |  | | 2,029 1,989 | 2.7% 2.6% | | 0.522 | 0.003 |
|  |  | 1 2 | | N17-N19 | Acute kidney failure and chronic kidney disease |  | | 3,394 3,595 | 4.5% 4.8% | | 0.014 | 0.013 |
|  |  | 1 2 | | A50-A64 | Infections with a predominantly sexual mode of transmission |  | | 856 911 | 1.1% 1.2% | | 0.188 | 0.007 |
|  |  | 1 2 | | E03 | Other hypothyroidism |  | | 1,995 2,092 | 2.7% 2.8% | | 0.124 | 0.008 |
|  |  | 1 2 | | E05 | Thyrotoxicosis [hyperthyroidism] |  | | 297 292 | 0.4% 0.4% | | 0.836 | 0.001 |
|  |  | 1 2 | | E83.1 | Disorders of iron metabolism |  | | 77 79 | 0.1% 0.1% | | 0.873 | 0.001 |
|  |  | 1 2 | | Q99 | Other chromosome abnormalities, not elsewhere classified |  | | 635 632 | 0.8% 0.8% | | 0.933 | <0.001 |
|  |  | 1 2 | | Q93 | Monosomies and deletions from the autosomes, not elsewhere classified |  | | 323 313 | 0.4% 0.4% | | 0.691 | 0.002 |
|  |  | 1 2 | | Q90 | Down syndrome |  | | 260 265 | 0.3% 0.4% | | 0.827 | 0.001 |
|  |  | 1 2 | | Q92 | Other trisomies and partial trisomies of the autosomes, not elsewhere classified |  | | 111 114 | 0.1% 0.2% | | 0.841 | 0.001 |
|  |  | 1 2 | | Q98.4 | Klinefelter syndrome, unspecified |  | | 58 63 | 0.1% 0.1% | | 0.649 | 0.002 |
|  |  | 1 2 | | Q98.5 | Karyotype 47, XYY |  | | 21 21 | 0.0% 0.0% | | 1 | <0.001 |
|  |  | 1 2 | | Q98.7 | Male with sex chromosome mosaicism |  | | 20 19 | 0.0% 0.0% | | 0.873 | 0.001 |
|  |  | 1 2 | | Q98.8 | Other specified sex chromosome abnormalities, male phenotype |  | | 20 23 | 0.0% 0.0% | | 0.647 | 0.002 |
|  |  | 1 2 | | Q98.0 | Klinefelter syndrome karyotype 47, XXY |  | | 10 11 | 0.0% 0.0% | | 0.827 | 0.001 |
|  |  | 1 2 | | Q98.1 | Klinefelter syndrome, male with more than two X chromosomes |  | | 10 10 | 0.0% 0.0% | | 1 | <0.001 |
|  |  | 1 2 | | Q98.6 | Male with structurally abnormal sex chromosome |  | | 10 0 | 0.0% 0% | | 0.002 | 0.016 |
|  |  | 1 2 | | Q98.9 | Sex chromosome abnormality, male phenotype, unspecified |  | | 10 10 | 0.0% 0.0% | | 1 | <0.001 |
|  |  | 1 2 | | D35.2 | Benign neoplasm of pituitary gland |  | | 72 80 | 0.1% 0.1% | | 0.516 | 0.003 |
|  |  | 1 2 | | D35.3 | Benign neoplasm of craniopharyngeal duct |  | | 31 42 | 0.0% 0.1% | | 0.198 | 0.007 |
|  |  | 1 2 | | E40-E46 | Malnutrition |  | | 1,659 1,692 | 2.2% 2.2% | | 0.564 | 0.003 |
|  |  | 1 2 | | G40.9 | Epilepsy, unspecified |  | | 22,874 21,243 | 30.4% 28.2% | | <0.001 | 0.048 |
|  |  | 1 2 | | G40.3 | Generalized idiopathic epilepsy and epileptic syndromes |  | | 6,384 6,038 | 8.5% 8.0% | | 0.001 | 0.017 |
|  |  | 1 2 | | G40.4 | Other generalized epilepsy and epileptic syndromes |  | | 4,985 4,714 | 6.6% 6.3% | | 0.004 | 0.015 |
|  |  | 1 2 | | G40.2 | Localization-related (focal) (partial) symptomatic epilepsy and epileptic syndromes with complex partial seizures |  | | 4,901 4,244 | 6.5% 5.6% | | <0.001 | 0.037 |
|  |  | 1 2 | | G40.8 | Other epilepsy and recurrent seizures |  | | 3,893 3,663 | 5.2% 4.9% | | 0.007 | 0.014 |
|  |  | 1 2 | | G40.1 | Localization-related (focal) (partial) symptomatic epilepsy and epileptic syndromes with simple partial seizures |  | | 3,582 3,124 | 4.8% 4.2% | | <0.001 | 0.030 |
|  |  | 1 2 | | G40.A | Absence epileptic syndrome |  | | 1,897 1,883 | 2.5% 2.5% | | 0.818 | 0.001 |
|  |  | 1 2 | | G40.0 | Localization-related (focal) (partial) idiopathic epilepsy and epileptic syndromes with seizures of localized onset |  | | 1,158 1,008 | 1.5% 1.3% | | 0.001 | 0.017 |
|  |  | 1 2 | | G40.5 | Epileptic seizures related to external causes |  | | 675 672 | 0.9% 0.9% | | 0.935 | <0.001 |
|  |  | 1 2 | | G40.B | Juvenile myoclonic epilepsy [impulsive petit mal] |  | | 529 510 | 0.7% 0.7% | | 0.554 | 0.003 |
|  |  | 1 2 | | F17 | Nicotine dependence |  | | 14,275 14,861 | 19.0% 19.8% | | <0.001 | 0.020 |
|  |  | 1 2 | | F12 | Cannabis related disorders |  | | 9,092 9,250 | 12.1% 12.3% | | 0.213 | 0.006 |
|  |  | 1 2 | | F10 | Alcohol related disorders |  | | 7,652 8,070 | 10.2% 10.7% | | <0.001 | 0.018 |
|  |  | 1 2 | | F19 | Other psychoactive substance related disorders |  | | 6,281 6,399 | 8.3% 8.5% | | 0.273 | 0.006 |
|  |  | 1 2 | | F11 | Opioid related disorders |  | | 3,731 3,962 | 5.0% 5.3% | | 0.007 | 0.014 |
|  |  | 1 2 | | F14 | Cocaine related disorders |  | | 3,121 3,212 | 4.1% 4.3% | | 0.243 | 0.006 |
|  |  | 1 2 | | F15 | Other stimulant related disorders |  | | 3,173 3,218 | 4.2% 4.3% | | 0.565 | 0.003 |
|  |  | 1 2 | | F13 | Sedative, hypnotic, or anxiolytic related disorders |  | | 1,599 1,619 | 2.1% 2.2% | | 0.722 | 0.002 |
|  |  | 1 2 | | F18 | Inhalant related disorders |  | | 1,291 1,321 | 1.7% 1.8% | | 0.554 | 0.003 |
|  |  | 1 2 | | F16 | Hallucinogen related disorders |  | | 726 743 | 1.0% 1.0% | | 0.656 | 0.002 |
|  | **Procedure** | | | | | | | | | | | |
|  |  | Cohort | | |  | Mean ± SD | | Patients | % of Cohort | | P-Value | SMD |
|  |  | 1 2 | | 1010843 | Radiation Oncology Treatment |  | | 145 164 | 0.2% 0.2% | | 0.279 | 0.006 |
|  |  | 1 2 | | 1008061 | Surgical Procedures on the Urinary System |  | | 1,985 1,997 | 2.6% 2.7% | | 0.847 | 0.001 |
|  |  | 1 2 | | 1008011 | Repair initial inguinal hernia, age 5 years or older |  | | 103 114 | 0.1% 0.2% | | 0.455 | 0.004 |
|  |  | 1 2 | | 1008470 | Surgical Procedures on the Male Genital System |  | | 687 699 | 0.9% 0.9% | | 0.746 | 0.002 |
|  | **Medication** | | | | | | | | | | | |
|  |  | Cohort | | |  | Mean ± SD | | Patients | % of Cohort | | P-Value | SMD |
|  |  | 1 2 | | 25025 | finasteride |  | | 175 193 | 0.2% 0.3% | | 0.347 | 0.005 |
|  |  | 1 2 | | CN750 | LITHIUM SALTS |  | | 3,116 3,166 | 4.1% 4.2% | | 0.519 | 0.003 |
|  |  | 1 2 | | 6135 | ketoconazole |  | | 1,052 1,081 | 1.4% 1.4% | | 0.527 | 0.003 |
|  |  | 1 2 | | 10829 | trimethoprim |  | | 4,060 4,372 | 5.4% 5.8% | | <0.001 | 0.018 |
|  |  | 1 2 | | 7454 | nitrofurantoin |  | | 317 336 | 0.4% 0.4% | | 0.456 | 0.004 |
|  |  | 1 2 | | AM200 | ERYTHROMYCINS/MACROLIDES |  | | 6,269 6,591 | 8.3% 8.8% | | 0.003 | 0.015 |
|  |  | 1 2 | | AM300 | AMINOGLYCOSIDES |  | | 3,354 3,454 | 4.5% 4.6% | | 0.215 | 0.006 |
|  |  | 1 2 | | AN000 | ANTINEOPLASTICS |  | | 1,213 1,334 | 1.6% 1.8% | | 0.016 | 0.012 |
|  |  | 1 2 | | CN709 | ANTIPSYCHOTICS,OTHER |  | | 28,519 29,518 | 37.9% 39.2% | | <0.001 | 0.027 |
|  |  | 1 2 | | CN701 | PHENOTHIAZINE/RELATED ANTIPSYCHOTICS |  | | 1,700 1,524 | 2.3% 2.0% | | 0.002 | 0.016 |
|  |  | 1 2 | | CN609 | ANTIDEPRESSANTS,OTHER |  | | 21,490 22,954 | 28.6% 30.5% | | <0.001 | 0.043 |
|  |  | 1 2 | | CN601 | TRICYCLIC ANTIDEPRESSANTS |  | | 2,376 2,495 | 3.2% 3.3% | | 0.083 | 0.009 |
|  |  | 1 2 | | CV100 | BETA BLOCKERS/RELATED |  | | 7,753 8,219 | 10.3% 10.9% | | <0.001 | 0.020 |
|  |  | 1 2 | | CV200 | CALCIUM CHANNEL BLOCKERS |  | | 3,965 4,214 | 5.3% 5.6% | | 0.005 | 0.015 |
|  |  | 1 2 | | CV800 | ACE INHIBITORS |  | | 3,617 3,916 | 4.8% 5.2% | | <0.001 | 0.018 |
|  |  | 1 2 | | CV150 | ALPHA BLOCKERS/RELATED |  | | 2,784 2,902 | 3.7% 3.9% | | 0.111 | 0.008 |
|  |  | 1 2 | | CV490 | ANTIHYPERTENSIVES,OTHER |  | | 9,593 10,068 | 12.8% 13.4% | | <0.001 | 0.019 |
|  |  | 1 2 | | HS100 | ANDROGENS/ANABOLICS |  | | 466 516 | 0.6% 0.7% | | 0.109 | 0.008 |
|  |  | 1 2 | | HS800 | PROGESTINS |  | | 199 215 | 0.3% 0.3% | | 0.431 | 0.004 |
|  |  | 1 2 | | HS300 | ESTROGENS |  | | 140 157 | 0.2% 0.2% | | 0.323 | 0.005 |
|  |  | 1 2 | | 9997 | spironolactone |  | | 528 573 | 0.7% 0.8% | | 0.173 | 0.007 |
|  |  | 1 2 | | 3014 | cyproterone |  | | 10 10 | 0.0% 0.0% | | 1 | <0.001 |
|  |  | 1 2 | | 114477 | levetiracetam |  | | 15,603 16,621 | 20.7% 22.1% | | <0.001 | 0.033 |
|  |  | 1 2 | | 28439 | lamotrigine |  | | 6,002 6,445 | 8.0% 8.6% | | <0.001 | 0.021 |
|  |  | 1 2 | | 25480 | gabapentin |  | | 6,726 7,276 | 8.9% 9.7% | | <0.001 | 0.025 |
|  |  | 1 2 | | 38404 | topiramate |  | | 3,527 3,586 | 4.7% 4.8% | | 0.474 | 0.004 |
|  |  | 1 2 | | 32624 | oxcarbazepine |  | | 3,993 3,996 | 5.3% 5.3% | | 0.972 | <0.001 |
|  |  | 1 2 | | 623400 | lacosamide |  | | 2,437 2,481 | 3.2% 3.3% | | 0.524 | 0.003 |
|  |  | 1 2 | | 8183 | phenytoin |  | | 2,508 2,600 | 3.3% 3.5% | | 0.190 | 0.007 |
|  |  | 1 2 | | 21241 | clobazam |  | | 1,736 1,693 | 2.3% 2.3% | | 0.458 | 0.004 |
|  |  | 1 2 | | 2002 | carbamazepine |  | | 2,378 2,486 | 3.2% 3.3% | | 0.115 | 0.008 |
|  |  | 1 2 | | 39998 | zonisamide |  | | 1,655 1,648 | 2.2% 2.2% | | 0.902 | 0.001 |
|  |  | 1 2 | | 72236 | fosphenytoin |  | | 1,282 1,237 | 1.7% 1.6% | | 0.366 | 0.005 |
|  |  | 1 2 | | 4135 | ethosuximide |  | | 861 800 | 1.1% 1.1% | | 0.132 | 0.008 |
|  |  | 1 2 | | 69036 | rufinamide |  | | 272 270 | 0.4% 0.4% | | 0.931 | <0.001 |
|  |  | 1 2 | | 1739745 | brivaracetam |  | | 244 262 | 0.3% 0.3% | | 0.423 | 0.004 |
|  |  | 1 2 | | 14851 | vigabatrin |  | | 234 237 | 0.3% 0.3% | | 0.890 | 0.001 |
|  |  | 1 2 | | 24812 | felbamate |  | | 188 184 | 0.2% 0.2% | | 0.836 | 0.001 |
|  |  | 1 2 | | 1356552 | perampanel |  | | 213 220 | 0.3% 0.3% | | 0.736 | 0.002 |
|  |  | 1 2 | | 2045371 | cannabidiol |  | | 177 181 | 0.2% 0.2% | | 0.832 | 0.001 |
|  |  | 1 2 | | 1482502 | eslicarbazepine |  | | 122 122 | 0.2% 0.2% | | 1 | <0.001 |
|  |  | 1 2 | | 8691 | primidone |  | | 114 123 | 0.2% 0.2% | | 0.558 | 0.003 |
|  |  | 1 2 | | 2265690 | cenobamate |  | | 66 70 | 0.1% 0.1% | | 0.731 | 0.002 |
|  |  | 1 2 | | 31914 | tiagabine |  | | 31 36 | 0.0% 0.0% | | 0.541 | 0.003 |
|  |  | 1 2 | | 4328 | fenfluramine |  | | 10 10 | 0.0% 0.0% | | 1 | <0.001 |
|  |  | 1 2 | | 47858 | methsuximide |  | | 10 10 | 0.0% 0.0% | | 1 | <0.001 |
|  |  | 1 2 | | 2054968 | stiripentol |  | | 10 10 | 0.0% 0.0% | | 1 | <0.001 |
|  |  | 1 2 | | CN302 | BENZODIAZEPINE DERIVATIVE SEDATIVES/HYPNOTICS |  | | 38,438 39,590 | 51.1% 52.6% | | <0.001 | 0.031 |
|  |  | 1 2 | | CN309 | SEDATIVES/HYPNOTICS,OTHER |  | | 7,860 8,063 | 10.4% 10.7% | | 0.089 | 0.009 |
|  |  | 1 2 | | CN301 | BARBITURIC ACID DERIVATIVE SEDATIVES/HYPNOTICS |  | | 2,334 2,347 | 3.1% 3.1% | | 0.847 | 0.001 |
|  |  | 1 2 | | CN101 | OPIOID ANALGESICS |  | | 21,710 23,198 | 28.9% 30.8% | | <0.001 | 0.043 |
|  |  | 1 2 | | HS051 | GLUCOCORTICOIDS |  | | 17,654 18,489 | 23.5% 24.6% | | <0.001 | 0.026 |
|  | **Laboratory** | | | | | | | | | | | |
|  |  | Cohort | | |  | Mean ± SD | | Patients | % of Cohort | | P-Value | SMD |
|  |  | 1 2 | | 9083 | BMI | 25.8 +/- 7.0 26.1 +/- 7.3 | | 39,245 41,407 | 52.2% 55.0% | | <0.001 | 0.043 |
|  |  | 1 2 | |  | 0 - 0 kg/m2 |  | | 39,316 41,496 | 52.3% 55.2% | | <0.001 | 0.058 |

**Abbreviations:** BMI = Body Mass Index; SD = Standard deviation; SMD = Standardised mean difference

**N.B.:** Uncorrected p-values were calculated using two-sided t-tests for continuous covariates and two-sided Z-tests for categorical covariates. These p-values reflect descriptive comparisons of baseline characteristics during propensity score matching. Correction for multiple comparisons is not appropriate in this context, as the aim is to assess covariate balance (reflected by a standardised mean difference of <0.1) rather than to test hypotheses. Exact p-values beyond three decimal places are not provided in the propensity score matching outputs generated by the TriNetX platform. Where p-values fall below this threshold, they are reported as <0.001.

# Supplementary Table 10: Propensity score matching men with epilepsy or bipolar disorder exposed (cohort 1) and unexposed (cohort 2) to valproate – 10-year outcome assessment

Propensity score matching was performed on all listed characteristics. Characteristics of the cohorts before and after matching are summarized in the table below.

| **Cohort 1 and cohort 2 patient count before and after propensity score matching** | | | | | | | | | | | | |
| --- | --- | --- | --- | --- | --- | --- | --- | --- | --- | --- | --- | --- |
|  | | | Cohort | | | Patient count before matching | | | | Patient count after matching | | |
|  | | | 1 - Men with epilepsy or bipolar disorder exposed to valproate_v9 | | | 82,046 | | | | 70,425 | | |
|  | | | 2 - Men with epilepsy or bipolar disorder not exposed to valproate_v9 | | | 469,361 | | | | 70,425 | | |
| **Propensity score density function - Before and after matching (cohort 1 - purple, cohort 2 - green)** | | | | | | | | | | | | |
|  |  | | 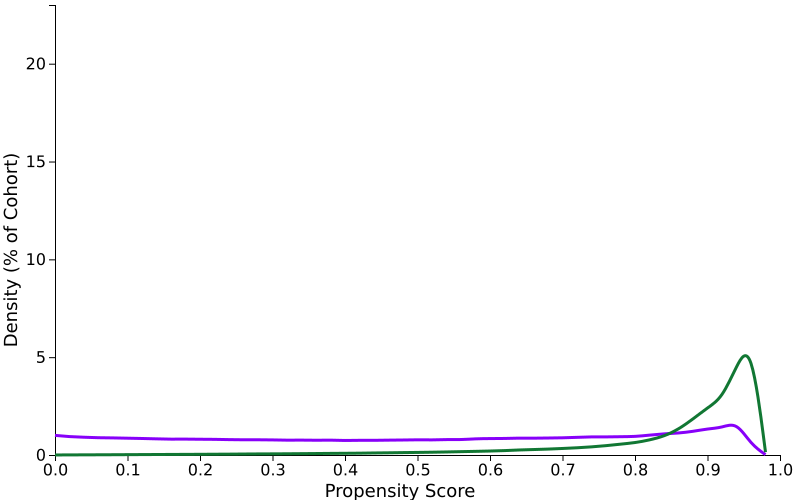 | | | | 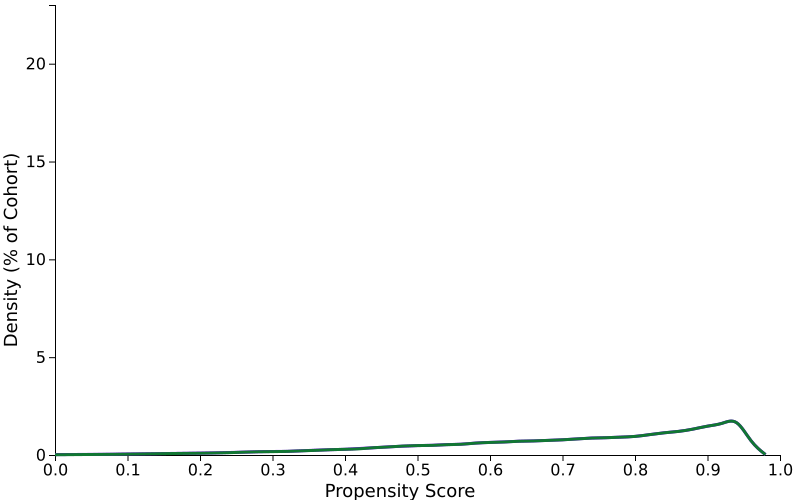 | | | | | |
| **Cohort 1 (N = 82,046) and cohort 2 (N = 469,361) characteristics before propensity score matching** | | | | | | | | | | | | |
|  | **Demographics** | | | | | | | | | | | |
|  |  | Cohort | | |  | Mean ± SD | | Patients | % of Cohort | | P-Value | SMD |
|  |  | 1 2 | | AI | Age at Index | 24.6 +/- 12.9 20.7 +/- 14.9 | | 79,536 451,492 | 100% 100% | | <0.001 | 0.282 |
|  |  | 1 2 | | 2106-3 | White |  | | 45,093 250,264 | 56.7% 55.4% | | <0.001 | 0.025 |
|  |  | 1 2 | | 1002-5 | American Indian or Alaska Native |  | | 340 1,690 | 0.4% 0.4% | | 0.025 | 0.008 |
|  |  | 1 2 | | UNK | Unknown Race |  | | 11,441 77,942 | 14.4% 17.3% | | <0.001 | 0.079 |
|  |  | 1 2 | | 2076-8 | Native Hawaiian or Other Pacific Islander |  | | 338 2,225 | 0.4% 0.5% | | 0.011 | 0.010 |
|  |  | 1 2 | | 2054-5 | Black or African American |  | | 14,244 74,826 | 17.9% 16.6% | | <0.001 | 0.035 |
|  |  | 1 2 | | 2131-1 | Other Race |  | | 4,498 28,998 | 5.7% 6.4% | | <0.001 | 0.032 |
|  |  | 1 2 | | 2028-9 | Asian |  | | 3,582 15,547 | 4.5% 3.4% | | <0.001 | 0.054 |
|  | **Diagnosis** | | | | | | | | | | | |
|  |  | Cohort | | |  | Mean ± SD | | Patients | % of Cohort | | P-Value | SMD |
|  |  | 1 2 | | F80-F89 | Pervasive and specific developmental disorders |  | | 12,883 41,949 | 16.2% 9.3% | | <0.001 | 0.208 |
|  |  | 1 2 | | F40-F48 | Anxiety, dissociative, stress-related, somatoform and other nonpsychotic mental disorders |  | | 21,194 73,711 | 26.6% 16.3% | | <0.001 | 0.253 |
|  |  | 1 2 | | F90-F98 | Behavioral and emotional disorders with onset usually occurring in childhood and adolescence |  | | 13,754 43,395 | 17.3% 9.6% | | <0.001 | 0.227 |
|  |  | 1 2 | | F20-F29 | Schizophrenia, schizotypal, delusional, and other non-mood psychotic disorders |  | | 12,183 22,724 | 15.3% 5.0% | | <0.001 | 0.345 |
|  |  | 1 2 | | F60-F69 | Disorders of adult personality and behavior |  | | 6,482 12,040 | 8.1% 2.7% | | <0.001 | 0.244 |
|  |  | 1 2 | | F70-F79 | Intellectual Disabilities |  | | 5,776 8,591 | 7.3% 1.9% | | <0.001 | 0.258 |
|  |  | 1 2 | | F01-F09 | Mental disorders due to known physiological conditions |  | | 4,039 7,406 | 5.1% 1.6% | | <0.001 | 0.192 |
|  |  | 1 2 | | F99-F99 | Unspecified mental disorder (F99) |  | | 2,241 4,217 | 2.8% 0.9% | | <0.001 | 0.139 |
|  |  | 1 2 | | E08-E13 | Diabetes mellitus |  | | 3,705 15,475 | 4.7% 3.4% | | <0.001 | 0.063 |
|  |  | 1 2 | | Q00-Q07 | Congenital malformations of the nervous system |  | | 2,953 9,025 | 3.7% 2.0% | | <0.001 | 0.103 |
|  |  | 1 2 | | Q60-Q64 | Congenital malformations of the urinary system |  | | 503 2,636 | 0.6% 0.6% | | 0.099 | 0.006 |
|  |  | 1 2 | | N45 | Orchitis and epididymitis |  | | 403 1,550 | 0.5% 0.3% | | <0.001 | 0.025 |
|  |  | 1 2 | | N43 | Hydrocele and spermatocele |  | | 476 2,419 | 0.6% 0.5% | | 0.027 | 0.008 |
|  |  | 1 2 | | N50.1 | Vascular disorders of male genital organs |  | | 37 135 | 0.0% 0.0% | | 0.016 | 0.009 |
|  |  | 1 2 | | N49 | Inflammatory disorders of male genital organs, not elsewhere classified |  | | 116 468 | 0.1% 0.1% | | 0.001 | 0.012 |
|  |  | 1 2 | | I86.1 | Scrotal varices |  | | 209 895 | 0.3% 0.2% | | <0.001 | 0.013 |
|  |  | 1 2 | | Q53 | Undescended and ectopic testicle |  | | 539 2,583 | 0.7% 0.6% | | <0.001 | 0.013 |
|  |  | 1 2 | | Q55 | Other congenital malformations of male genital organs |  | | 352 2,281 | 0.4% 0.5% | | 0.020 | 0.009 |
|  |  | 1 2 | | Q54 | Hypospadias |  | | 200 1,395 | 0.3% 0.3% | | 0.006 | 0.011 |
|  |  | 1 2 | | C60-C63 | Malignant neoplasms of male genital organs |  | | 85 513 | 0.1% 0.1% | | 0.601 | 0.002 |
|  |  | 1 2 | | N44.8 | Other noninflammatory disorders of the testis |  | | 222 652 | 0.3% 0.1% | | <0.001 | 0.029 |
|  |  | 1 2 | | N44.0 | Torsion of testis |  | | 45 277 | 0.1% 0.1% | | 0.614 | 0.002 |
|  |  | 1 2 | | N30-N39 | Other diseases of the urinary system |  | | 4,243 14,202 | 5.3% 3.1% | | <0.001 | 0.109 |
|  |  | 1 2 | | K40 | Inguinal hernia |  | | 766 4,378 | 1.0% 1.0% | | 0.861 | 0.001 |
|  |  | 1 2 | | E84 | Cystic fibrosis |  | | 59 300 | 0.1% 0.1% | | 0.439 | 0.003 |
|  |  | 1 2 | | S30-S39 | Injuries to the abdomen, lower back, lumbar spine, pelvis and external genitals |  | | 4,430 15,827 | 5.6% 3.5% | | <0.001 | 0.099 |
|  |  | 1 2 | | E23.0 | Hypopituitarism |  | | 333 1,335 | 0.4% 0.3% | | <0.001 | 0.021 |
|  |  | 1 2 | | R56 | Convulsions, not elsewhere classified |  | | 29,181 222,822 | 36.7% 49.4% | | <0.001 | 0.258 |
|  |  | 1 2 | | E34.5 | Androgen insensitivity syndrome |  | | 10 14 | 0.0% 0.0% | | <0.001 | 0.011 |
|  |  | 1 2 | | Q87.1 | Congenital malformation syndromes predominantly associated with short stature |  | | 82 333 | 0.1% 0.1% | | 0.006 | 0.010 |
|  |  | 1 2 | | F31 | Bipolar disorder |  | | 22,318 105,182 | 28.1% 23.3% | | <0.001 | 0.109 |
|  |  | 1 2 | | F32 | Depressive episode |  | | 12,854 42,359 | 16.2% 9.4% | | <0.001 | 0.204 |
|  |  | 1 2 | | F39 | Unspecified mood [affective] disorder |  | | 6,046 11,073 | 7.6% 2.5% | | <0.001 | 0.237 |
|  |  | 1 2 | | F33 | Major depressive disorder, recurrent |  | | 3,903 12,352 | 4.9% 2.7% | | <0.001 | 0.113 |
|  |  | 1 2 | | F34 | Persistent mood [affective] disorders |  | | 1,944 4,748 | 2.4% 1.1% | | <0.001 | 0.106 |
|  |  | 1 2 | | F30 | Manic episode |  | | 2,030 2,517 | 2.6% 0.6% | | <0.001 | 0.162 |
|  |  | 1 2 | | F50-F59 | Behavioral syndromes associated with physiological disturbances and physical factors |  | | 2,450 7,329 | 3.1% 1.6% | | <0.001 | 0.096 |
|  |  | 1 2 | | J00-J99 | Diseases of the respiratory system |  | | 25,765 128,150 | 32.4% 28.4% | | <0.001 | 0.087 |
|  |  | 1 2 | | I00-I99 | Diseases of the circulatory system |  | | 18,994 79,470 | 23.9% 17.6% | | <0.001 | 0.155 |
|  |  | 1 2 | | K00-K95 | Diseases of the digestive system |  | | 22,995 103,597 | 28.9% 22.9% | | <0.001 | 0.136 |
|  |  | 1 2 | | Q65-Q79 | Congenital malformations and deformations of the musculoskeletal system |  | | 2,590 13,390 | 3.3% 3.0% | | <0.001 | 0.017 |
|  |  | 1 2 | | N17-N19 | Acute kidney failure and chronic kidney disease |  | | 3,720 14,983 | 4.7% 3.3% | | <0.001 | 0.069 |
|  |  | 1 2 | | A50-A64 | Infections with a predominantly sexual mode of transmission |  | | 919 3,747 | 1.2% 0.8% | | <0.001 | 0.033 |
|  |  | 1 2 | | E03 | Other hypothyroidism |  | | 2,250 6,756 | 2.8% 1.5% | | <0.001 | 0.092 |
|  |  | 1 2 | | E05 | Thyrotoxicosis [hyperthyroidism] |  | | 322 980 | 0.4% 0.2% | | <0.001 | 0.034 |
|  |  | 1 2 | | E83.1 | Disorders of iron metabolism |  | | 88 482 | 0.1% 0.1% | | 0.758 | 0.001 |
|  |  | 1 2 | | Q99 | Other chromosome abnormalities, not elsewhere classified |  | | 982 2,649 | 1.2% 0.6% | | <0.001 | 0.068 |
|  |  | 1 2 | | Q93 | Monosomies and deletions from the autosomes, not elsewhere classified |  | | 439 1,467 | 0.6% 0.3% | | <0.001 | 0.034 |
|  |  | 1 2 | | Q90 | Down syndrome |  | | 301 1,582 | 0.4% 0.4% | | 0.220 | 0.005 |
|  |  | 1 2 | | Q92 | Other trisomies and partial trisomies of the autosomes, not elsewhere classified |  | | 178 498 | 0.2% 0.1% | | <0.001 | 0.028 |
|  |  | 1 2 | | Q98.4 | Klinefelter syndrome, unspecified |  | | 70 201 | 0.1% 0.0% | | <0.001 | 0.017 |
|  |  | 1 2 | | Q98.5 | Karyotype 47, XYY |  | | 28 89 | 0.0% 0.0% | | 0.007 | 0.009 |
|  |  | 1 2 | | Q98.7 | Male with sex chromosome mosaicism |  | | 26 57 | 0.0% 0.0% | | <0.001 | 0.013 |
|  |  | 1 2 | | Q98.8 | Other specified sex chromosome abnormalities, male phenotype |  | | 28 64 | 0.0% 0.0% | | <0.001 | 0.013 |
|  |  | 1 2 | | Q98.0 | Klinefelter syndrome karyotype 47, XXY |  | | 15 46 | 0.0% 0.0% | | 0.035 | 0.007 |
|  |  | 1 2 | | Q98.1 | Klinefelter syndrome, male with more than two X chromosomes |  | | 10 14 | 0.0% 0.0% | | <0.001 | 0.011 |
|  |  | 1 2 | | Q98.6 | Male with structurally abnormal sex chromosome |  | | 10 10 | 0.0% 0.0% | | <0.001 | 0.012 |
|  |  | 1 2 | | Q98.9 | Sex chromosome abnormality, male phenotype, unspecified |  | | 10 10 | 0.0% 0.0% | | <0.001 | 0.012 |
|  |  | 1 2 | | D35.2 | Benign neoplasm of pituitary gland |  | | 85 352 | 0.1% 0.1% | | 0.009 | 0.010 |
|  |  | 1 2 | | D35.3 | Benign neoplasm of craniopharyngeal duct |  | | 39 139 | 0.0% 0.0% | | 0.010 | 0.009 |
|  |  | 1 2 | | E40-E46 | Malnutrition |  | | 1,963 6,568 | 2.5% 1.5% | | <0.001 | 0.073 |
|  |  | 1 2 | | G40.9 | Epilepsy, unspecified |  | | 28,014 88,602 | 35.2% 19.6% | | <0.001 | 0.355 |
|  |  | 1 2 | | G40.3 | Generalized idiopathic epilepsy and epileptic syndromes |  | | 9,675 13,679 | 12.2% 3.0% | | <0.001 | 0.350 |
|  |  | 1 2 | | G40.4 | Other generalized epilepsy and epileptic syndromes |  | | 7,967 11,858 | 10.0% 2.6% | | <0.001 | 0.307 |
|  |  | 1 2 | | G40.2 | Localization-related (focal) (partial) symptomatic epilepsy and epileptic syndromes with complex partial seizures |  | | 7,436 17,963 | 9.3% 4.0% | | <0.001 | 0.217 |
|  |  | 1 2 | | G40.8 | Other epilepsy and recurrent seizures |  | | 6,122 16,014 | 7.7% 3.5% | | <0.001 | 0.181 |
|  |  | 1 2 | | G40.1 | Localization-related (focal) (partial) symptomatic epilepsy and epileptic syndromes with simple partial seizures |  | | 5,892 13,724 | 7.4% 3.0% | | <0.001 | 0.197 |
|  |  | 1 2 | | G40.A | Absence epileptic syndrome |  | | 2,882 4,683 | 3.6% 1.0% | | <0.001 | 0.172 |
|  |  | 1 2 | | G40.0 | Localization-related (focal) (partial) idiopathic epilepsy and epileptic syndromes with seizures of localized onset |  | | 1,947 4,509 | 2.4% 1.0% | | <0.001 | 0.112 |
|  |  | 1 2 | | G40.5 | Epileptic seizures related to external causes |  | | 1,125 2,378 | 1.4% 0.5% | | <0.001 | 0.091 |
|  |  | 1 2 | | G40.B | Juvenile myoclonic epilepsy [impulsive petit mal] |  | | 826 681 | 1.0% 0.2% | | <0.001 | 0.116 |
|  |  | 1 2 | | F17 | Nicotine dependence |  | | 14,162 51,964 | 17.8% 11.5% | | <0.001 | 0.179 |
|  |  | 1 2 | | F12 | Cannabis related disorders |  | | 9,773 24,668 | 12.3% 5.5% | | <0.001 | 0.242 |
|  |  | 1 2 | | F10 | Alcohol related disorders |  | | 7,857 32,258 | 9.9% 7.1% | | <0.001 | 0.098 |
|  |  | 1 2 | | F19 | Other psychoactive substance related disorders |  | | 6,682 19,031 | 8.4% 4.2% | | <0.001 | 0.173 |
|  |  | 1 2 | | F11 | Opioid related disorders |  | | 3,800 14,015 | 4.8% 3.1% | | <0.001 | 0.086 |
|  |  | 1 2 | | F14 | Cocaine related disorders |  | | 3,391 9,027 | 4.3% 2.0% | | <0.001 | 0.130 |
|  |  | 1 2 | | F15 | Other stimulant related disorders |  | | 3,260 8,768 | 4.1% 1.9% | | <0.001 | 0.126 |
|  |  | 1 2 | | F13 | Sedative, hypnotic, or anxiolytic related disorders |  | | 1,739 4,700 | 2.2% 1.0% | | <0.001 | 0.091 |
|  |  | 1 2 | | F18 | Inhalant related disorders |  | | 1,548 4,247 | 1.9% 0.9% | | <0.001 | 0.084 |
|  |  | 1 2 | | F16 | Hallucinogen related disorders |  | | 791 1,699 | 1.0% 0.4% | | <0.001 | 0.075 |
|  | **Procedure** | | | | | | | | | | | |
|  |  | Cohort | | |  | Mean ± SD | | Patients | % of Cohort | | P-Value | SMD |
|  |  | 1 2 | | 1010843 | Radiation Oncology Treatment |  | | 165 703 | 0.2% 0.2% | | 0.001 | 0.012 |
|  |  | 1 2 | | 1008061 | Surgical Procedures on the Urinary System |  | | 2,329 8,015 | 2.9% 1.8% | | <0.001 | 0.076 |
|  |  | 1 2 | | 1008011 | Repair initial inguinal hernia, age 5 years or older |  | | 113 405 | 0.1% 0.1% | | <0.001 | 0.015 |
|  |  | 1 2 | | 1008470 | Surgical Procedures on the Male Genital System |  | | 802 4,668 | 1.0% 1.0% | | 0.510 | 0.003 |
|  | **Medication** | | | | | | | | | | | |
|  |  | Cohort | | |  | Mean ± SD | | Patients | % of Cohort | | P-Value | SMD |
|  |  | 1 2 | | 25025 | finasteride |  | | 177 808 | 0.2% 0.2% | | 0.008 | 0.010 |
|  |  | 1 2 | | CN750 | LITHIUM SALTS |  | | 3,570 6,813 | 4.5% 1.5% | | <0.001 | 0.175 |
|  |  | 1 2 | | 6135 | ketoconazole |  | | 1,225 4,385 | 1.5% 1.0% | | <0.001 | 0.051 |
|  |  | 1 2 | | 10829 | trimethoprim |  | | 4,500 17,246 | 5.7% 3.8% | | <0.001 | 0.087 |
|  |  | 1 2 | | 7454 | nitrofurantoin |  | | 365 1,081 | 0.5% 0.2% | | <0.001 | 0.037 |
|  |  | 1 2 | | AM200 | ERYTHROMYCINS/MACROLIDES |  | | 6,945 26,048 | 8.7% 5.8% | | <0.001 | 0.114 |
|  |  | 1 2 | | AM300 | AMINOGLYCOSIDES |  | | 3,943 12,634 | 5.0% 2.8% | | <0.001 | 0.112 |
|  |  | 1 2 | | AN000 | ANTINEOPLASTICS |  | | 1,347 5,861 | 1.7% 1.3% | | <0.001 | 0.033 |
|  |  | 1 2 | | CN709 | ANTIPSYCHOTICS,OTHER |  | | 31,037 52,158 | 39.0% 11.6% | | <0.001 | 0.666 |
|  |  | 1 2 | | CN701 | PHENOTHIAZINE/RELATED ANTIPSYCHOTICS |  | | 2,556 1,924 | 3.2% 0.4% | | <0.001 | 0.210 |
|  |  | 1 2 | | CN609 | ANTIDEPRESSANTS,OTHER |  | | 23,171 55,319 | 29.1% 12.3% | | <0.001 | 0.426 |
|  |  | 1 2 | | CN601 | TRICYCLIC ANTIDEPRESSANTS |  | | 2,636 6,275 | 3.3% 1.4% | | <0.001 | 0.127 |
|  |  | 1 2 | | CV100 | BETA BLOCKERS/RELATED |  | | 8,446 22,712 | 10.6% 5.0% | | <0.001 | 0.209 |
|  |  | 1 2 | | CV200 | CALCIUM CHANNEL BLOCKERS |  | | 4,290 13,253 | 5.4% 2.9% | | <0.001 | 0.123 |
|  |  | 1 2 | | CV800 | ACE INHIBITORS |  | | 3,836 12,686 | 4.8% 2.8% | | <0.001 | 0.105 |
|  |  | 1 2 | | CV150 | ALPHA BLOCKERS/RELATED |  | | 3,031 6,599 | 3.8% 1.5% | | <0.001 | 0.147 |
|  |  | 1 2 | | CV490 | ANTIHYPERTENSIVES,OTHER |  | | 10,866 26,343 | 13.7% 5.8% | | <0.001 | 0.266 |
|  |  | 1 2 | | HS100 | ANDROGENS/ANABOLICS |  | | 500 2,157 | 0.6% 0.5% | | <0.001 | 0.020 |
|  |  | 1 2 | | HS800 | PROGESTINS |  | | 230 743 | 0.3% 0.2% | | <0.001 | 0.026 |
|  |  | 1 2 | | HS300 | ESTROGENS |  | | 146 701 | 0.2% 0.2% | | 0.065 | 0.007 |
|  |  | 1 2 | | 9997 | spironolactone |  | | 541 3,029 | 0.7% 0.7% | | 0.767 | 0.001 |
|  |  | 1 2 | | 3014 | cyproterone |  | | 10 10 | 0.0% 0.0% | | <0.001 | 0.012 |
|  |  | 1 2 | | 114477 | levetiracetam |  | | 19,192 56,521 | 24.1% 12.5% | | <0.001 | 0.304 |
|  |  | 1 2 | | 28439 | lamotrigine |  | | 7,197 20,406 | 9.0% 4.5% | | <0.001 | 0.181 |
|  |  | 1 2 | | 25480 | gabapentin |  | | 7,398 20,150 | 9.3% 4.5% | | <0.001 | 0.192 |
|  |  | 1 2 | | 38404 | topiramate |  | | 5,036 7,007 | 6.3% 1.6% | | <0.001 | 0.248 |
|  |  | 1 2 | | 32624 | oxcarbazepine |  | | 5,439 11,017 | 6.8% 2.4% | | <0.001 | 0.210 |
|  |  | 1 2 | | 623400 | lacosamide |  | | 3,896 4,790 | 4.9% 1.1% | | <0.001 | 0.227 |
|  |  | 1 2 | | 8183 | phenytoin |  | | 3,237 7,275 | 4.1% 1.6% | | <0.001 | 0.148 |
|  |  | 1 2 | | 21241 | clobazam |  | | 3,321 2,479 | 4.2% 0.5% | | <0.001 | 0.241 |
|  |  | 1 2 | | 2002 | carbamazepine |  | | 3,020 7,058 | 3.8% 1.6% | | <0.001 | 0.139 |
|  |  | 1 2 | | 39998 | zonisamide |  | | 2,833 3,331 | 3.6% 0.7% | | <0.001 | 0.196 |
|  |  | 1 2 | | 72236 | fosphenytoin |  | | 2,094 3,256 | 2.6% 0.7% | | <0.001 | 0.149 |
|  |  | 1 2 | | 4135 | ethosuximide |  | | 1,258 1,214 | 1.6% 0.3% | | <0.001 | 0.137 |
|  |  | 1 2 | | 69036 | rufinamide |  | | 580 348 | 0.7% 0.1% | | <0.001 | 0.103 |
|  |  | 1 2 | | 1739745 | brivaracetam |  | | 435 486 | 0.5% 0.1% | | <0.001 | 0.077 |
|  |  | 1 2 | | 14851 | vigabatrin |  | | 593 300 | 0.7% 0.1% | | <0.001 | 0.107 |
|  |  | 1 2 | | 24812 | felbamate |  | | 342 268 | 0.4% 0.1% | | <0.001 | 0.075 |
|  |  | 1 2 | | 1356552 | perampanel |  | | 398 364 | 0.5% 0.1% | | <0.001 | 0.078 |
|  |  | 1 2 | | 2045371 | cannabidiol |  | | 407 310 | 0.5% 0.1% | | <0.001 | 0.082 |
|  |  | 1 2 | | 1482502 | eslicarbazepine |  | | 182 352 | 0.2% 0.1% | | <0.001 | 0.039 |
|  |  | 1 2 | | 8691 | primidone |  | | 135 260 | 0.2% 0.1% | | <0.001 | 0.033 |
|  |  | 1 2 | | 2265690 | cenobamate |  | | 103 185 | 0.1% 0.0% | | <0.001 | 0.030 |
|  |  | 1 2 | | 31914 | tiagabine |  | | 36 52 | 0.0% 0.0% | | <0.001 | 0.020 |
|  |  | 1 2 | | 4328 | fenfluramine |  | | 30 13 | 0.0% 0.0% | | <0.001 | 0.024 |
|  |  | 1 2 | | 47858 | methsuximide |  | | 10 10 | 0.0% 0.0% | | <0.001 | 0.012 |
|  |  | 1 2 | | 2054968 | stiripentol |  | | 13 10 | 0.0% 0.0% | | <0.001 | 0.015 |
|  |  | 1 2 | | CN302 | BENZODIAZEPINE DERIVATIVE SEDATIVES/HYPNOTICS |  | | 43,703 114,124 | 54.9% 25.3% | | <0.001 | 0.635 |
|  |  | 1 2 | | CN309 | SEDATIVES/HYPNOTICS,OTHER |  | | 9,029 21,530 | 11.4% 4.8% | | <0.001 | 0.244 |
|  |  | 1 2 | | CN301 | BARBITURIC ACID DERIVATIVE SEDATIVES/HYPNOTICS |  | | 3,282 8,037 | 4.1% 1.8% | | <0.001 | 0.139 |
|  |  | 1 2 | | CN101 | OPIOID ANALGESICS |  | | 23,581 89,913 | 29.6% 19.9% | | <0.001 | 0.227 |
|  |  | 1 2 | | HS051 | GLUCOCORTICOIDS |  | | 19,540 82,597 | 24.6% 18.3% | | <0.001 | 0.153 |
|  | **Laboratory** | | | | | | | | | | | |
|  |  | Cohort | | |  | Mean ± SD | | Patients | % of Cohort | | P-Value | SMD |
|  |  | 1 2 | | 9083 | BMI | 25.6 +/- 7.0 25.2 +/- 7.5 | | 41,191 175,372 | 51.8% 38.8% | | <0.001 | 0.058 |
|  |  | 1 2 | |  | 0 - 0 kg/m2 |  | | 41,265 175,809 | 51.9% 38.9% | | <0.001 | 0.262 |
| **Cohort 1 (N = 70,425) and cohort 2 (N = 70,425) characteristics after propensity score matching** | | | | | | | | | | | | |
|  | **Demographics** | | | | | | | | | | | |
|  |  | Cohort | | |  | Mean ± SD | | Patients | % of Cohort | | P-Value | SMD |
|  |  | 1 2 | | AI | Age at Index | 24.8 +/- 12.8 26.1 +/- 13.5 | | 70,425 70,425 | 100% 100% | | <0.001 | 0.094 |
|  |  | 1 2 | | 2106-3 | White |  | | 39,819 40,438 | 56.5% 57.4% | | 0.001 | 0.018 |
|  |  | 1 2 | | 1002-5 | American Indian or Alaska Native |  | | 287 318 | 0.4% 0.5% | | 0.207 | 0.007 |
|  |  | 1 2 | | UNK | Unknown Race |  | | 10,450 9,932 | 14.8% 14.1% | | <0.001 | 0.021 |
|  |  | 1 2 | | 2076-8 | Native Hawaiian or Other Pacific Islander |  | | 304 317 | 0.4% 0.5% | | 0.601 | 0.003 |
|  |  | 1 2 | | 2054-5 | Black or African American |  | | 12,431 12,371 | 17.7% 17.6% | | 0.675 | 0.002 |
|  |  | 1 2 | | 2131-1 | Other Race |  | | 4,026 4,019 | 5.7% 5.7% | | 0.936 | <0.001 |
|  |  | 1 2 | | 2028-9 | Asian |  | | 3,108 3,030 | 4.4% 4.3% | | 0.309 | 0.005 |
|  | **Diagnosis** | | | | | | | | | | | |
|  |  | Cohort | | |  | Mean ± SD | | Patients | % of Cohort | | P-Value | SMD |
|  |  | 1 2 | | F80-F89 | Pervasive and specific developmental disorders |  | | 9,451 9,267 | 13.4% 13.2% | | 0.149 | 0.008 |
|  |  | 1 2 | | F40-F48 | Anxiety, dissociative, stress-related, somatoform and other nonpsychotic mental disorders |  | | 17,871 18,982 | 25.4% 27.0% | | <0.001 | 0.036 |
|  |  | 1 2 | | F90-F98 | Behavioral and emotional disorders with onset usually occurring in childhood and adolescence |  | | 10,880 11,296 | 15.4% 16.0% | | 0.002 | 0.016 |
|  |  | 1 2 | | F20-F29 | Schizophrenia, schizotypal, delusional, and other non-mood psychotic disorders |  | | 9,945 9,720 | 14.1% 13.8% | | 0.084 | 0.009 |
|  |  | 1 2 | | F60-F69 | Disorders of adult personality and behavior |  | | 4,763 4,738 | 6.8% 6.7% | | 0.791 | 0.001 |
|  |  | 1 2 | | F70-F79 | Intellectual Disabilities |  | | 3,717 3,463 | 5.3% 4.9% | | 0.002 | 0.016 |
|  |  | 1 2 | | F01-F09 | Mental disorders due to known physiological conditions |  | | 2,840 2,863 | 4.0% 4.1% | | 0.756 | 0.002 |
|  |  | 1 2 | | F99-F99 | Unspecified mental disorder (F99) |  | | 1,691 1,669 | 2.4% 2.4% | | 0.701 | 0.002 |
|  |  | 1 2 | | E08-E13 | Diabetes mellitus |  | | 3,221 3,551 | 4.6% 5.0% | | <0.001 | 0.022 |
|  |  | 1 2 | | Q00-Q07 | Congenital malformations of the nervous system |  | | 1,944 1,977 | 2.8% 2.8% | | 0.593 | 0.003 |
|  |  | 1 2 | | Q60-Q64 | Congenital malformations of the urinary system |  | | 393 420 | 0.6% 0.6% | | 0.342 | 0.005 |
|  |  | 1 2 | | N45 | Orchitis and epididymitis |  | | 349 378 | 0.5% 0.5% | | 0.281 | 0.006 |
|  |  | 1 2 | | N43 | Hydrocele and spermatocele |  | | 396 410 | 0.6% 0.6% | | 0.621 | 0.003 |
|  |  | 1 2 | | N50.1 | Vascular disorders of male genital organs |  | | 30 28 | 0.0% 0.0% | | 0.793 | 0.001 |
|  |  | 1 2 | | N49 | Inflammatory disorders of male genital organs, not elsewhere classified |  | | 97 96 | 0.1% 0.1% | | 0.943 | <0.001 |
|  |  | 1 2 | | I86.1 | Scrotal varices |  | | 180 198 | 0.3% 0.3% | | 0.354 | 0.005 |
|  |  | 1 2 | | Q53 | Undescended and ectopic testicle |  | | 384 404 | 0.5% 0.6% | | 0.475 | 0.004 |
|  |  | 1 2 | | Q55 | Other congenital malformations of male genital organs |  | | 240 239 | 0.3% 0.3% | | 0.963 | <0.001 |
|  |  | 1 2 | | Q54 | Hypospadias |  | | 154 152 | 0.2% 0.2% | | 0.909 | 0.001 |
|  |  | 1 2 | | C60-C63 | Malignant neoplasms of male genital organs |  | | 76 92 | 0.1% 0.1% | | 0.217 | 0.007 |
|  |  | 1 2 | | N44.8 | Other noninflammatory disorders of the testis |  | | 185 183 | 0.3% 0.3% | | 0.917 | 0.001 |
|  |  | 1 2 | | N44.0 | Torsion of testis |  | | 43 45 | 0.1% 0.1% | | 0.831 | 0.001 |
|  |  | 1 2 | | N30-N39 | Other diseases of the urinary system |  | | 3,280 3,514 | 4.7% 5.0% | | 0.004 | 0.016 |
|  |  | 1 2 | | K40 | Inguinal hernia |  | | 625 656 | 0.9% 0.9% | | 0.384 | 0.005 |
|  |  | 1 2 | | E84 | Cystic fibrosis |  | | 49 59 | 0.1% 0.1% | | 0.336 | 0.005 |
|  |  | 1 2 | | S30-S39 | Injuries to the abdomen, lower back, lumbar spine, pelvis and external genitals |  | | 3,688 3,884 | 5.2% 5.5% | | 0.021 | 0.012 |
|  |  | 1 2 | | E23.0 | Hypopituitarism |  | | 266 266 | 0.4% 0.4% | | 1 | <0.001 |
|  |  | 1 2 | | R56 | Convulsions, not elsewhere classified |  | | 23,460 21,445 | 33.3% 30.5% | | <0.001 | 0.061 |
|  |  | 1 2 | | E34.5 | Androgen insensitivity syndrome |  | | 10 10 | 0.0% 0.0% | | 1 | <0.001 |
|  |  | 1 2 | | Q87.1 | Congenital malformation syndromes predominantly associated with short stature |  | | 64 62 | 0.1% 0.1% | | 0.859 | 0.001 |
|  |  | 1 2 | | F31 | Bipolar disorder |  | | 20,530 22,339 | 29.2% 31.7% | | <0.001 | 0.056 |
|  |  | 1 2 | | F32 | Depressive episode |  | | 10,947 11,869 | 15.5% 16.9% | | <0.001 | 0.036 |
|  |  | 1 2 | | F39 | Unspecified mood [affective] disorder |  | | 4,722 4,803 | 6.7% 6.8% | | 0.390 | 0.005 |
|  |  | 1 2 | | F33 | Major depressive disorder, recurrent |  | | 3,417 3,686 | 4.9% 5.2% | | 0.001 | 0.017 |
|  |  | 1 2 | | F34 | Persistent mood [affective] disorders |  | | 1,569 1,694 | 2.2% 2.4% | | 0.027 | 0.012 |
|  |  | 1 2 | | F30 | Manic episode |  | | 1,531 1,414 | 2.2% 2.0% | | 0.029 | 0.012 |
|  |  | 1 2 | | F50-F59 | Behavioral syndromes associated with physiological disturbances and physical factors |  | | 1,922 2,060 | 2.7% 2.9% | | 0.027 | 0.012 |
|  |  | 1 2 | | J00-J99 | Diseases of the respiratory system |  | | 21,273 21,567 | 30.2% 30.6% | | 0.089 | 0.009 |
|  |  | 1 2 | | I00-I99 | Diseases of the circulatory system |  | | 15,780 16,786 | 22.4% 23.8% | | <0.001 | 0.034 |
|  |  | 1 2 | | K00-K95 | Diseases of the digestive system |  | | 18,859 19,870 | 26.8% 28.2% | | <0.001 | 0.032 |
|  |  | 1 2 | | Q65-Q79 | Congenital malformations and deformations of the musculoskeletal system |  | | 1,864 1,904 | 2.6% 2.7% | | 0.509 | 0.004 |
|  |  | 1 2 | | N17-N19 | Acute kidney failure and chronic kidney disease |  | | 3,067 3,371 | 4.4% 4.8% | | <0.001 | 0.021 |
|  |  | 1 2 | | A50-A64 | Infections with a predominantly sexual mode of transmission |  | | 814 890 | 1.2% 1.3% | | 0.064 | 0.010 |
|  |  | 1 2 | | E03 | Other hypothyroidism |  | | 1,762 1,798 | 2.5% 2.6% | | 0.541 | 0.003 |
|  |  | 1 2 | | E05 | Thyrotoxicosis [hyperthyroidism] |  | | 273 258 | 0.4% 0.4% | | 0.514 | 0.003 |
|  |  | 1 2 | | E83.1 | Disorders of iron metabolism |  | | 73 87 | 0.1% 0.1% | | 0.268 | 0.006 |
|  |  | 1 2 | | Q99 | Other chromosome abnormalities, not elsewhere classified |  | | 594 596 | 0.8% 0.8% | | 0.954 | <0.001 |
|  |  | 1 2 | | Q93 | Monosomies and deletions from the autosomes, not elsewhere classified |  | | 299 298 | 0.4% 0.4% | | 0.967 | <0.001 |
|  |  | 1 2 | | Q90 | Down syndrome |  | | 243 258 | 0.3% 0.4% | | 0.502 | 0.004 |
|  |  | 1 2 | | Q92 | Other trisomies and partial trisomies of the autosomes, not elsewhere classified |  | | 109 118 | 0.2% 0.2% | | 0.550 | 0.003 |
|  |  | 1 2 | | Q98.4 | Klinefelter syndrome, unspecified |  | | 53 57 | 0.1% 0.1% | | 0.703 | 0.002 |
|  |  | 1 2 | | Q98.5 | Karyotype 47, XYY |  | | 19 20 | 0.0% 0.0% | | 0.873 | 0.001 |
|  |  | 1 2 | | Q98.7 | Male with sex chromosome mosaicism |  | | 17 16 | 0.0% 0.0% | | 0.862 | 0.001 |
|  |  | 1 2 | | Q98.8 | Other specified sex chromosome abnormalities, male phenotype |  | | 19 18 | 0.0% 0.0% | | 0.869 | 0.001 |
|  |  | 1 2 | | Q98.0 | Klinefelter syndrome karyotype 47, XXY |  | | 10 12 | 0.0% 0.0% | | 0.670 | 0.002 |
|  |  | 1 2 | | Q98.1 | Klinefelter syndrome, male with more than two X chromosomes |  | | 10 10 | 0.0% 0.0% | | 1 | <0.001 |
|  |  | 1 2 | | Q98.6 | Male with structurally abnormal sex chromosome |  | | 10 0 | 0.0% 0% | | 0.002 | 0.017 |
|  |  | 1 2 | | Q98.9 | Sex chromosome abnormality, male phenotype, unspecified |  | | 10 10 | 0.0% 0.0% | | 1 | <0.001 |
|  |  | 1 2 | | D35.2 | Benign neoplasm of pituitary gland |  | | 68 80 | 0.1% 0.1% | | 0.324 | 0.005 |
|  |  | 1 2 | | D35.3 | Benign neoplasm of craniopharyngeal duct |  | | 32 36 | 0.0% 0.1% | | 0.628 | 0.003 |
|  |  | 1 2 | | E40-E46 | Malnutrition |  | | 1,469 1,600 | 2.1% 2.3% | | 0.017 | 0.013 |
|  |  | 1 2 | | G40.9 | Epilepsy, unspecified |  | | 21,477 20,009 | 30.5% 28.4% | | <0.001 | 0.046 |
|  |  | 1 2 | | G40.3 | Generalized idiopathic epilepsy and epileptic syndromes |  | | 6,017 5,703 | 8.5% 8.1% | | 0.002 | 0.016 |
|  |  | 1 2 | | G40.4 | Other generalized epilepsy and epileptic syndromes |  | | 4,628 4,351 | 6.6% 6.2% | | 0.003 | 0.016 |
|  |  | 1 2 | | G40.2 | Localization-related (focal) (partial) symptomatic epilepsy and epileptic syndromes with complex partial seizures |  | | 4,724 4,087 | 6.7% 5.8% | | <0.001 | 0.037 |
|  |  | 1 2 | | G40.8 | Other epilepsy and recurrent seizures |  | | 3,714 3,497 | 5.3% 5.0% | | 0.009 | 0.014 |
|  |  | 1 2 | | G40.1 | Localization-related (focal) (partial) symptomatic epilepsy and epileptic syndromes with simple partial seizures |  | | 3,464 3,133 | 4.9% 4.4% | | <0.001 | 0.022 |
|  |  | 1 2 | | G40.A | Absence epileptic syndrome |  | | 1,766 1,724 | 2.5% 2.4% | | 0.472 | 0.004 |
|  |  | 1 2 | | G40.0 | Localization-related (focal) (partial) idiopathic epilepsy and epileptic syndromes with seizures of localized onset |  | | 1,120 962 | 1.6% 1.4% | | <0.001 | 0.019 |
|  |  | 1 2 | | G40.5 | Epileptic seizures related to external causes |  | | 636 584 | 0.9% 0.8% | | 0.135 | 0.008 |
|  |  | 1 2 | | G40.B | Juvenile myoclonic epilepsy [impulsive petit mal] |  | | 513 461 | 0.7% 0.7% | | 0.095 | 0.009 |
|  |  | 1 2 | | F17 | Nicotine dependence |  | | 12,289 13,123 | 17.4% 18.6% | | <0.001 | 0.031 |
|  |  | 1 2 | | F12 | Cannabis related disorders |  | | 8,149 8,351 | 11.6% 11.9% | | 0.094 | 0.009 |
|  |  | 1 2 | | F10 | Alcohol related disorders |  | | 6,965 7,495 | 9.9% 10.6% | | <0.001 | 0.025 |
|  |  | 1 2 | | F19 | Other psychoactive substance related disorders |  | | 5,685 5,937 | 8.1% 8.4% | | 0.015 | 0.013 |
|  |  | 1 2 | | F11 | Opioid related disorders |  | | 3,323 3,558 | 4.7% 5.1% | | 0.004 | 0.015 |
|  |  | 1 2 | | F14 | Cocaine related disorders |  | | 2,846 2,983 | 4.0% 4.2% | | 0.067 | 0.010 |
|  |  | 1 2 | | F15 | Other stimulant related disorders |  | | 2,797 2,914 | 4.0% 4.1% | | 0.114 | 0.008 |
|  |  | 1 2 | | F13 | Sedative, hypnotic, or anxiolytic related disorders |  | | 1,463 1,517 | 2.1% 2.2% | | 0.317 | 0.005 |
|  |  | 1 2 | | F18 | Inhalant related disorders |  | | 1,276 1,371 | 1.8% 1.9% | | 0.062 | 0.010 |
|  |  | 1 2 | | F16 | Hallucinogen related disorders |  | | 660 687 | 0.9% 1.0% | | 0.460 | 0.004 |
|  | **Procedure** | | | | | | | | | | | |
|  |  | Cohort | | |  | Mean ± SD | | Patients | % of Cohort | | P-Value | SMD |
|  |  | 1 2 | | 1010843 | Radiation Oncology Treatment |  | | 134 139 | 0.2% 0.2% | | 0.762 | 0.002 |
|  |  | 1 2 | | 1008061 | Surgical Procedures on the Urinary System |  | | 1,775 1,852 | 2.5% 2.6% | | 0.195 | 0.007 |
|  |  | 1 2 | | 1008011 | Repair initial inguinal hernia, age 5 years or older |  | | 91 112 | 0.1% 0.2% | | 0.140 | 0.008 |
|  |  | 1 2 | | 1008470 | Surgical Procedures on the Male Genital System |  | | 647 640 | 0.9% 0.9% | | 0.845 | 0.001 |
|  | **Medication** | | | | | | | | | | | |
|  |  | Cohort | | |  | Mean ± SD | | Patients | % of Cohort | | P-Value | SMD |
|  |  | 1 2 | | 25025 | finasteride |  | | 162 175 | 0.2% 0.2% | | 0.478 | 0.004 |
|  |  | 1 2 | | CN750 | LITHIUM SALTS |  | | 2,921 2,944 | 4.1% 4.2% | | 0.759 | 0.002 |
|  |  | 1 2 | | 6135 | ketoconazole |  | | 978 1,002 | 1.4% 1.4% | | 0.587 | 0.003 |
|  |  | 1 2 | | 10829 | trimethoprim |  | | 3,671 3,988 | 5.2% 5.7% | | <0.001 | 0.020 |
|  |  | 1 2 | | 7454 | nitrofurantoin |  | | 284 315 | 0.4% 0.4% | | 0.204 | 0.007 |
|  |  | 1 2 | | AM200 | ERYTHROMYCINS/MACROLIDES |  | | 5,634 6,153 | 8% 8.7% | | <0.001 | 0.027 |
|  |  | 1 2 | | AM300 | AMINOGLYCOSIDES |  | | 3,075 3,337 | 4.4% 4.7% | | 0.001 | 0.018 |
|  |  | 1 2 | | AN000 | ANTINEOPLASTICS |  | | 1,115 1,275 | 1.6% 1.8% | | 0.001 | 0.018 |
|  |  | 1 2 | | CN709 | ANTIPSYCHOTICS,OTHER |  | | 26,127 27,126 | 37.1% 38.5% | | <0.001 | 0.029 |
|  |  | 1 2 | | CN701 | PHENOTHIAZINE/RELATED ANTIPSYCHOTICS |  | | 1,588 1,378 | 2.3% 2.0% | | <0.001 | 0.021 |
|  |  | 1 2 | | CN609 | ANTIDEPRESSANTS,OTHER |  | | 19,480 21,055 | 27.7% 29.9% | | <0.001 | 0.049 |
|  |  | 1 2 | | CN601 | TRICYCLIC ANTIDEPRESSANTS |  | | 2,161 2,342 | 3.1% 3.3% | | 0.006 | 0.015 |
|  |  | 1 2 | | CV100 | BETA BLOCKERS/RELATED |  | | 6,976 7,396 | 9.9% 10.5% | | <0.001 | 0.020 |
|  |  | 1 2 | | CV200 | CALCIUM CHANNEL BLOCKERS |  | | 3,617 3,945 | 5.1% 5.6% | | <0.001 | 0.021 |
|  |  | 1 2 | | CV800 | ACE INHIBITORS |  | | 3,246 3,610 | 4.6% 5.1% | | <0.001 | 0.024 |
|  |  | 1 2 | | CV150 | ALPHA BLOCKERS/RELATED |  | | 2,468 2,563 | 3.5% 3.6% | | 0.173 | 0.007 |
|  |  | 1 2 | | CV490 | ANTIHYPERTENSIVES,OTHER |  | | 8,640 9,203 | 12.3% 13.1% | | <0.001 | 0.024 |
|  |  | 1 2 | | HS100 | ANDROGENS/ANABOLICS |  | | 440 491 | 0.6% 0.7% | | 0.094 | 0.009 |
|  |  | 1 2 | | HS800 | PROGESTINS |  | | 192 219 | 0.3% 0.3% | | 0.182 | 0.007 |
|  |  | 1 2 | | HS300 | ESTROGENS |  | | 122 156 | 0.2% 0.2% | | 0.041 | 0.011 |
|  |  | 1 2 | | 9997 | spironolactone |  | | 475 552 | 0.7% 0.8% | | 0.016 | 0.013 |
|  |  | 1 2 | | 3014 | cyproterone |  | | 10 10 | 0.0% 0.0% | | 1 | <0.001 |
|  |  | 1 2 | | 114477 | levetiracetam |  | | 14,699 15,189 | 20.9% 21.6% | | 0.001 | 0.017 |
|  |  | 1 2 | | 28439 | lamotrigine |  | | 5,607 5,952 | 8.0% 8.5% | | 0.001 | 0.018 |
|  |  | 1 2 | | 25480 | gabapentin |  | | 6,245 6,759 | 8.9% 9.6% | | <0.001 | 0.025 |
|  |  | 1 2 | | 38404 | topiramate |  | | 3,281 3,342 | 4.7% 4.7% | | 0.443 | 0.004 |
|  |  | 1 2 | | 32624 | oxcarbazepine |  | | 3,821 3,896 | 5.4% 5.5% | | 0.380 | 0.005 |
|  |  | 1 2 | | 623400 | lacosamide |  | | 2,375 2,382 | 3.4% 3.4% | | 0.918 | 0.001 |
|  |  | 1 2 | | 8183 | phenytoin |  | | 2,297 2,484 | 3.3% 3.5% | | 0.006 | 0.015 |
|  |  | 1 2 | | 21241 | clobazam |  | | 1,755 1,690 | 2.5% 2.4% | | 0.262 | 0.006 |
|  |  | 1 2 | | 2002 | carbamazepine |  | | 2,256 2,385 | 3.2% 3.4% | | 0.054 | 0.010 |
|  |  | 1 2 | | 39998 | zonisamide |  | | 1,642 1,668 | 2.3% 2.4% | | 0.647 | 0.002 |
|  |  | 1 2 | | 72236 | fosphenytoin |  | | 1,168 1,155 | 1.7% 1.6% | | 0.786 | 0.001 |
|  |  | 1 2 | | 4135 | ethosuximide |  | | 820 770 | 1.2% 1.1% | | 0.207 | 0.007 |
|  |  | 1 2 | | 69036 | rufinamide |  | | 284 265 | 0.4% 0.4% | | 0.417 | 0.004 |
|  |  | 1 2 | | 1739745 | brivaracetam |  | | 235 242 | 0.3% 0.3% | | 0.748 | 0.002 |
|  |  | 1 2 | | 14851 | vigabatrin |  | | 249 228 | 0.4% 0.3% | | 0.335 | 0.005 |
|  |  | 1 2 | | 24812 | felbamate |  | | 182 193 | 0.3% 0.3% | | 0.569 | 0.003 |
|  |  | 1 2 | | 1356552 | perampanel |  | | 217 222 | 0.3% 0.3% | | 0.811 | 0.001 |
|  |  | 1 2 | | 2045371 | cannabidiol |  | | 183 194 | 0.3% 0.3% | | 0.571 | 0.003 |
|  |  | 1 2 | | 1482502 | eslicarbazepine |  | | 119 118 | 0.2% 0.2% | | 0.948 | <0.001 |
|  |  | 1 2 | | 8691 | primidone |  | | 110 120 | 0.2% 0.2% | | 0.509 | 0.004 |
|  |  | 1 2 | | 2265690 | cenobamate |  | | 67 64 | 0.1% 0.1% | | 0.793 | 0.001 |
|  |  | 1 2 | | 31914 | tiagabine |  | | 30 27 | 0.0% 0.0% | | 0.691 | 0.002 |
|  |  | 1 2 | | 4328 | fenfluramine |  | | 11 10 | 0.0% 0.0% | | 0.827 | 0.001 |
|  |  | 1 2 | | 47858 | methsuximide |  | | 10 10 | 0.0% 0.0% | | 1 | <0.001 |
|  |  | 1 2 | | 2054968 | stiripentol |  | | 10 10 | 0.0% 0.0% | | 1 | <0.001 |
|  |  | 1 2 | | CN302 | BENZODIAZEPINE DERIVATIVE SEDATIVES/HYPNOTICS |  | | 35,721 37,123 | 50.7% 52.7% | | <0.001 | 0.040 |
|  |  | 1 2 | | CN309 | SEDATIVES/HYPNOTICS,OTHER |  | | 7,068 7,342 | 10.0% 10.4% | | 0.016 | 0.013 |
|  |  | 1 2 | | CN301 | BARBITURIC ACID DERIVATIVE SEDATIVES/HYPNOTICS |  | | 2,218 2,270 | 3.1% 3.2% | | 0.430 | 0.004 |
|  |  | 1 2 | | CN101 | OPIOID ANALGESICS |  | | 19,727 21,562 | 28.0% 30.6% | | <0.001 | 0.057 |
|  |  | 1 2 | | HS051 | GLUCOCORTICOIDS |  | | 16,020 17,207 | 22.7% 24.4% | | <0.001 | 0.040 |
|  | **Laboratory** | | | | | | | | | | | |
|  |  | Cohort | | |  | Mean ± SD | | Patients | % of Cohort | | P-Value | SMD |
|  |  | 1 2 | | 9083 | BMI | 25.8 +/- 7.0 26.1 +/- 7.3 | | 35,201 37,542 | 50.0% 53.3% | | <0.001 | 0.044 |
|  |  | 1 2 | |  | 0 - 0 kg/m2 |  | | 35,271 37,626 | 50.1% 53.4% | | <0.001 | 0.067 |

**Abbreviations:** BMI = Body Mass Index; SD = Standard deviation; SMD = Standardised mean difference

**N.B.:** Uncorrected p-values were calculated using two-sided t-tests for continuous covariates and two-sided Z-tests for categorical covariates. These p-values reflect descriptive comparisons of baseline characteristics during propensity score matching. Correction for multiple comparisons is not appropriate in this context, as the aim is to assess covariate balance (reflected by a standardised mean difference of <0.1) rather than to test hypotheses. Exact p-values beyond three decimal places are not provided in the propensity score matching outputs generated by the TriNetX platform. Where p-values fall below this threshold, they are reported as <0.001.

# Supplementary Table 11: Propensity score matching men with epilepsy exposed (cohort 1) and unexposed (cohort 2) to valproate

Propensity score matching was performed on all listed characteristics. Characteristics of the cohorts before and after matching are summarized in the table below.

| **Cohort 1 and cohort 2 patient count before and after propensity score matching** | | | | | | | | | | | | |
| --- | --- | --- | --- | --- | --- | --- | --- | --- | --- | --- | --- | --- |
|  | | | Cohort | | | Patient count before matching | | | | Patient count after matching | | |
|  | | | 1 - Men with epilepsy, exposed to valproate_v9 | | | 60,860 | | | | 50,102 | | |
|  | | | 2 - Men with epilepsy, not exposed to valproate_v9 | | | 399,984 | | | | 50,102 | | |
| **Propensity score density function - Before and after matching (cohort 1 - purple, cohort 2 - green)** | | | | | | | | | | | | |
|  |  | | 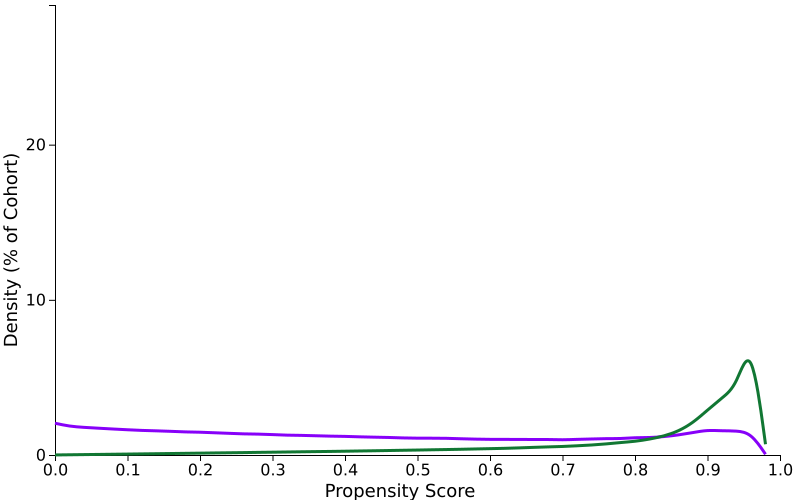 | | | | 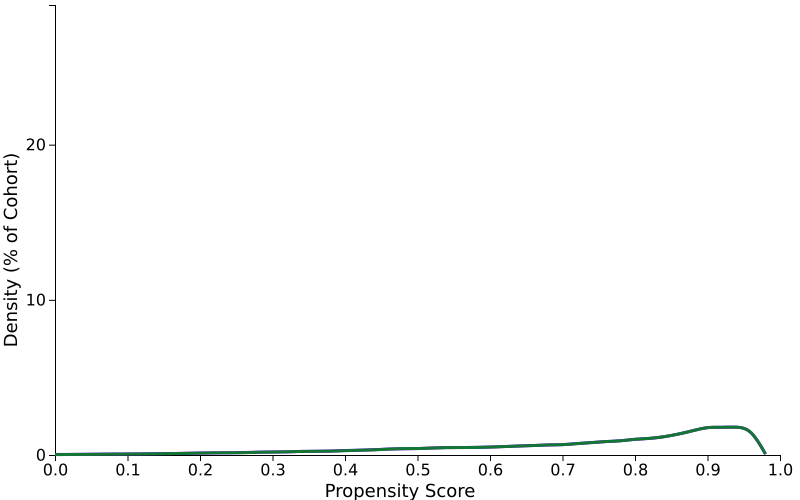 | | | | | |
| **Cohort 1 (N = 60,860) and cohort 2 (N = 399,984) characteristics before propensity score matching** | | | | | | | | | | | | |
|  | **Demographics** | | | | | | | | | | | |
|  |  | Cohort | | |  | Mean ± SD | | Patients | % of Cohort | | P-Value | SMD |
|  |  | 1 2 | | AI | Age at Index | 22.4 +/- 13.3 18.3 +/- 15.0 | | 59,364 383,085 | 100% 100% | | <0.001 | 0.289 |
|  |  | 1 2 | | 2106-3 | White |  | | 33,644 205,908 | 56.7% 53.7% | | <0.001 | 0.059 |
|  |  | 1 2 | | 1002-5 | American Indian or Alaska Native |  | | 244 1,301 | 0.4% 0.3% | | 0.006 | 0.012 |
|  |  | 1 2 | | UNK | Unknown Race |  | | 8,888 68,740 | 15.0% 17.9% | | <0.001 | 0.080 |
|  |  | 1 2 | | 2076-8 | Native Hawaiian or Other Pacific Islander |  | | 200 1,807 | 0.3% 0.5% | | <0.001 | 0.021 |
|  |  | 1 2 | | 2054-5 | Black or African American |  | | 10,035 65,736 | 16.9% 17.2% | | 0.124 | 0.007 |
|  |  | 1 2 | | 2131-1 | Other Race |  | | 3,664 26,024 | 6.2% 6.8% | | <0.001 | 0.025 |
|  |  | 1 2 | | 2028-9 | Asian |  | | 2,689 13,569 | 4.5% 3.5% | | <0.001 | 0.050 |
|  | **Diagnosis** | | | | | | | | | | | |
|  |  | Cohort | | |  | Mean ± SD | | Patients | % of Cohort | | P-Value | SMD |
|  |  | 1 2 | | F80-F89 | Pervasive and specific developmental disorders |  | | 11,803 40,694 | 19.9% 10.6% | | <0.001 | 0.260 |
|  |  | 1 2 | | F40-F48 | Anxiety, dissociative, stress-related, somatoform and other nonpsychotic mental disorders |  | | 11,248 38,517 | 18.9% 10.1% | | <0.001 | 0.255 |
|  |  | 1 2 | | F90-F98 | Behavioral and emotional disorders with onset usually occurring in childhood and adolescence |  | | 8,873 26,437 | 14.9% 6.9% | | <0.001 | 0.260 |
|  |  | 1 2 | | F20-F29 | Schizophrenia, schizotypal, delusional, and other non-mood psychotic disorders |  | | 4,133 6,279 | 7.0% 1.6% | | <0.001 | 0.265 |
|  |  | 1 2 | | F60-F69 | Disorders of adult personality and behavior |  | | 3,229 5,329 | 5.4% 1.4% | | <0.001 | 0.224 |
|  |  | 1 2 | | F70-F79 | Intellectual Disabilities |  | | 5,274 8,250 | 8.9% 2.2% | | <0.001 | 0.298 |
|  |  | 1 2 | | F01-F09 | Mental disorders due to known physiological conditions |  | | 3,189 6,101 | 5.4% 1.6% | | <0.001 | 0.207 |
|  |  | 1 2 | | F99-F99 | Unspecified mental disorder (F99) |  | | 1,001 1,692 | 1.7% 0.4% | | <0.001 | 0.122 |
|  |  | 1 2 | | E08-E13 | Diabetes mellitus |  | | 2,457 11,424 | 4.1% 3.0% | | <0.001 | 0.062 |
|  |  | 1 2 | | Q00-Q07 | Congenital malformations of the nervous system |  | | 3,063 9,650 | 5.2% 2.5% | | <0.001 | 0.138 |
|  |  | 1 2 | | Q60-Q64 | Congenital malformations of the urinary system |  | | 441 2,661 | 0.7% 0.7% | | 0.190 | 0.006 |
|  |  | 1 2 | | N45 | Orchitis and epididymitis |  | | 246 1,082 | 0.4% 0.3% | | <0.001 | 0.022 |
|  |  | 1 2 | | N43 | Hydrocele and spermatocele |  | | 377 2,165 | 0.6% 0.6% | | 0.036 | 0.009 |
|  |  | 1 2 | | N50.1 | Vascular disorders of male genital organs |  | | 26 100 | 0.0% 0.0% | | 0.017 | 0.009 |
|  |  | 1 2 | | N49 | Inflammatory disorders of male genital organs, not elsewhere classified |  | | 81 345 | 0.1% 0.1% | | 0.001 | 0.014 |
|  |  | 1 2 | | I86.1 | Scrotal varices |  | | 105 595 | 0.2% 0.2% | | 0.219 | 0.005 |
|  |  | 1 2 | | Q53 | Undescended and ectopic testicle |  | | 520 2,641 | 0.9% 0.7% | | <0.001 | 0.021 |
|  |  | 1 2 | | Q55 | Other congenital malformations of male genital organs |  | | 329 2,350 | 0.6% 0.6% | | 0.083 | 0.008 |
|  |  | 1 2 | | Q54 | Hypospadias |  | | 188 1,451 | 0.3% 0.4% | | 0.021 | 0.011 |
|  |  | 1 2 | | C60-C63 | Malignant neoplasms of male genital organs |  | | 65 418 | 0.1% 0.1% | | 0.979 | <0.001 |
|  |  | 1 2 | | N44.8 | Other noninflammatory disorders of the testis |  | | 138 419 | 0.2% 0.1% | | <0.001 | 0.030 |
|  |  | 1 2 | | N44.0 | Torsion of testis |  | | 40 237 | 0.1% 0.1% | | 0.617 | 0.002 |
|  |  | 1 2 | | N30-N39 | Other diseases of the urinary system |  | | 3,474 12,648 | 5.9% 3.3% | | <0.001 | 0.122 |
|  |  | 1 2 | | K40 | Inguinal hernia |  | | 621 3,871 | 1.0% 1.0% | | 0.421 | 0.004 |
|  |  | 1 2 | | E84 | Cystic fibrosis |  | | 44 254 | 0.1% 0.1% | | 0.495 | 0.003 |
|  |  | 1 2 | | S30-S39 | Injuries to the abdomen, lower back, lumbar spine, pelvis and external genitals |  | | 3,137 12,442 | 5.3% 3.2% | | <0.001 | 0.101 |
|  |  | 1 2 | | E23.0 | Hypopituitarism |  | | 326 1,336 | 0.5% 0.3% | | <0.001 | 0.030 |
|  |  | 1 2 | | R56 | Convulsions, not elsewhere classified |  | | 31,064 245,567 | 52.3% 64.1% | | <0.001 | 0.240 |
|  |  | 1 2 | | E34.5 | Androgen insensitivity syndrome |  | | 10 10 | 0.0% 0.0% | | <0.001 | 0.014 |
|  |  | 1 2 | | Q87.1 | Congenital malformation syndromes predominantly associated with short stature |  | | 77 336 | 0.1% 0.1% | | 0.002 | 0.013 |
|  |  | 1 2 | | F31 | Bipolar disorder |  | | 4,122 6,543 | 6.9% 1.7% | | <0.001 | 0.260 |
|  |  | 1 2 | | F32 | Depressive episode |  | | 6,737 21,830 | 11.3% 5.7% | | <0.001 | 0.203 |
|  |  | 1 2 | | F39 | Unspecified mood [affective] disorder |  | | 2,542 3,811 | 4.3% 1.0% | | <0.001 | 0.206 |
|  |  | 1 2 | | F33 | Major depressive disorder, recurrent |  | | 1,613 4,542 | 2.7% 1.2% | | <0.001 | 0.111 |
|  |  | 1 2 | | F34 | Persistent mood [affective] disorders |  | | 931 2,050 | 1.6% 0.5% | | <0.001 | 0.101 |
|  |  | 1 2 | | F30 | Manic episode |  | | 251 251 | 0.4% 0.1% | | <0.001 | 0.072 |
|  |  | 1 2 | | F50-F59 | Behavioral syndromes associated with physiological disturbances and physical factors |  | | 1,544 4,740 | 2.6% 1.2% | | <0.001 | 0.100 |
|  |  | 1 2 | | J00-J99 | Diseases of the respiratory system |  | | 19,978 112,754 | 33.7% 29.4% | | <0.001 | 0.091 |
|  |  | 1 2 | | I00-I99 | Diseases of the circulatory system |  | | 14,105 66,316 | 23.8% 17.3% | | <0.001 | 0.160 |
|  |  | 1 2 | | K00-K95 | Diseases of the digestive system |  | | 17,173 86,431 | 28.9% 22.6% | | <0.001 | 0.146 |
|  |  | 1 2 | | Q65-Q79 | Congenital malformations and deformations of the musculoskeletal system |  | | 2,486 13,663 | 4.2% 3.6% | | <0.001 | 0.032 |
|  |  | 1 2 | | N17-N19 | Acute kidney failure and chronic kidney disease |  | | 2,967 13,680 | 5.0% 3.6% | | <0.001 | 0.071 |
|  |  | 1 2 | | A50-A64 | Infections with a predominantly sexual mode of transmission |  | | 384 1,850 | 0.6% 0.5% | | <0.001 | 0.022 |
|  |  | 1 2 | | E03 | Other hypothyroidism |  | | 1,582 5,098 | 2.7% 1.3% | | <0.001 | 0.095 |
|  |  | 1 2 | | E05 | Thyrotoxicosis [hyperthyroidism] |  | | 190 648 | 0.3% 0.2% | | <0.001 | 0.031 |
|  |  | 1 2 | | E83.1 | Disorders of iron metabolism |  | | 75 432 | 0.1% 0.1% | | 0.363 | 0.004 |
|  |  | 1 2 | | Q99 | Other chromosome abnormalities, not elsewhere classified |  | | 1,023 2,791 | 1.7% 0.7% | | <0.001 | 0.090 |
|  |  | 1 2 | | Q93 | Monosomies and deletions from the autosomes, not elsewhere classified |  | | 468 1,568 | 0.8% 0.4% | | <0.001 | 0.049 |
|  |  | 1 2 | | Q90 | Down syndrome |  | | 324 1,686 | 0.5% 0.4% | | <0.001 | 0.015 |
|  |  | 1 2 | | Q92 | Other trisomies and partial trisomies of the autosomes, not elsewhere classified |  | | 188 522 | 0.3% 0.1% | | <0.001 | 0.038 |
|  |  | 1 2 | | Q98.4 | Klinefelter syndrome, unspecified |  | | 59 163 | 0.1% 0.0% | | <0.001 | 0.021 |
|  |  | 1 2 | | Q98.5 | Karyotype 47, XYY |  | | 26 85 | 0.0% 0.0% | | 0.002 | 0.012 |
|  |  | 1 2 | | Q98.7 | Male with sex chromosome mosaicism |  | | 26 54 | 0.0% 0.0% | | <0.001 | 0.017 |
|  |  | 1 2 | | Q98.8 | Other specified sex chromosome abnormalities, male phenotype |  | | 25 61 | 0.0% 0.0% | | <0.001 | 0.015 |
|  |  | 1 2 | | Q98.0 | Klinefelter syndrome karyotype 47, XXY |  | | 16 48 | 0.0% 0.0% | | 0.007 | 0.010 |
|  |  | 1 2 | | Q98.1 | Klinefelter syndrome, male with more than two X chromosomes |  | | 10 15 | 0.0% 0.0% | | <0.001 | 0.013 |
|  |  | 1 2 | | Q98.6 | Male with structurally abnormal sex chromosome |  | | 10 10 | 0.0% 0.0% | | <0.001 | 0.014 |
|  |  | 1 2 | | Q98.9 | Sex chromosome abnormality, male phenotype, unspecified |  | | 10 10 | 0.0% 0.0% | | <0.001 | 0.014 |
|  |  | 1 2 | | D35.2 | Benign neoplasm of pituitary gland |  | | 73 298 | 0.1% 0.1% | | <0.001 | 0.014 |
|  |  | 1 2 | | D35.3 | Benign neoplasm of craniopharyngeal duct |  | | 26 115 | 0.0% 0.0% | | 0.080 | 0.007 |
|  |  | 1 2 | | E40-E46 | Malnutrition |  | | 1,836 6,488 | 3.1% 1.7% | | <0.001 | 0.092 |
|  |  | 1 2 | | G40.9 | Epilepsy, unspecified |  | | 30,249 97,915 | 51.0% 25.6% | | <0.001 | 0.541 |
|  |  | 1 2 | | G40.3 | Generalized idiopathic epilepsy and epileptic syndromes |  | | 10,540 14,893 | 17.8% 3.9% | | <0.001 | 0.458 |
|  |  | 1 2 | | G40.4 | Other generalized epilepsy and epileptic syndromes |  | | 8,841 13,035 | 14.9% 3.4% | | <0.001 | 0.407 |
|  |  | 1 2 | | G40.2 | Localization-related (focal) (partial) symptomatic epilepsy and epileptic syndromes with complex partial seizures |  | | 7,998 19,247 | 13.5% 5.0% | | <0.001 | 0.295 |
|  |  | 1 2 | | G40.8 | Other epilepsy and recurrent seizures |  | | 6,441 17,325 | 10.9% 4.5% | | <0.001 | 0.239 |
|  |  | 1 2 | | G40.1 | Localization-related (focal) (partial) symptomatic epilepsy and epileptic syndromes with simple partial seizures |  | | 6,225 14,547 | 10.5% 3.8% | | <0.001 | 0.262 |
|  |  | 1 2 | | G40.A | Absence epileptic syndrome |  | | 3,207 5,072 | 5.4% 1.3% | | <0.001 | 0.228 |
|  |  | 1 2 | | G40.0 | Localization-related (focal) (partial) idiopathic epilepsy and epileptic syndromes with seizures of localized onset |  | | 2,043 4,794 | 3.4% 1.3% | | <0.001 | 0.145 |
|  |  | 1 2 | | G40.5 | Epileptic seizures related to external causes |  | | 1,222 2,605 | 2.1% 0.7% | | <0.001 | 0.119 |
|  |  | 1 2 | | G40.B | Juvenile myoclonic epilepsy [impulsive petit mal] |  | | 906 753 | 1.5% 0.2% | | <0.001 | 0.144 |
|  |  | 1 2 | | F17 | Nicotine dependence |  | | 7,118 31,233 | 12.0% 8.2% | | <0.001 | 0.128 |
|  |  | 1 2 | | F12 | Cannabis related disorders |  | | 3,821 12,009 | 6.4% 3.1% | | <0.001 | 0.155 |
|  |  | 1 2 | | F10 | Alcohol related disorders |  | | 3,730 21,576 | 6.3% 5.6% | | <0.001 | 0.028 |
|  |  | 1 2 | | F19 | Other psychoactive substance related disorders |  | | 2,812 9,482 | 4.7% 2.5% | | <0.001 | 0.122 |
|  |  | 1 2 | | F11 | Opioid related disorders |  | | 1,807 7,358 | 3.0% 1.9% | | <0.001 | 0.072 |
|  |  | 1 2 | | F14 | Cocaine related disorders |  | | 1,288 4,174 | 2.2% 1.1% | | <0.001 | 0.085 |
|  |  | 1 2 | | F15 | Other stimulant related disorders |  | | 1,218 3,561 | 2.1% 0.9% | | <0.001 | 0.093 |
|  |  | 1 2 | | F13 | Sedative, hypnotic, or anxiolytic related disorders |  | | 872 3,063 | 1.5% 0.8% | | <0.001 | 0.063 |
|  |  | 1 2 | | F18 | Inhalant related disorders |  | | 648 1,937 | 1.1% 0.5% | | <0.001 | 0.066 |
|  |  | 1 2 | | F16 | Hallucinogen related disorders |  | | 272 686 | 0.5% 0.2% | | <0.001 | 0.050 |
|  | **Procedure** | | | | | | | | | | | |
|  |  | Cohort | | |  | Mean ± SD | | Patients | % of Cohort | | P-Value | SMD |
|  |  | 1 2 | | 1010843 | Radiation Oncology Treatment |  | | 169 762 | 0.3% 0.2% | | <0.001 | 0.017 |
|  |  | 1 2 | | 1008061 | Surgical Procedures on the Urinary System |  | | 1,905 7,370 | 3.2% 1.9% | | <0.001 | 0.081 |
|  |  | 1 2 | | 1008011 | Repair initial inguinal hernia, age 5 years or older |  | | 79 301 | 0.1% 0.1% | | <0.001 | 0.017 |
|  |  | 1 2 | | 1008470 | Surgical Procedures on the Male Genital System |  | | 636 4,403 | 1.1% 1.1% | | 0.096 | 0.007 |
|  | **Medication** | | | | | | | | | | | |
|  |  | Cohort | | |  | Mean ± SD | | Patients | % of Cohort | | P-Value | SMD |
|  |  | 1 2 | | 25025 | finasteride |  | | 108 451 | 0.2% 0.1% | | <0.001 | 0.017 |
|  |  | 1 2 | | CN750 | LITHIUM SALTS |  | | 684 885 | 1.2% 0.2% | | <0.001 | 0.111 |
|  |  | 1 2 | | 6135 | ketoconazole |  | | 773 3,380 | 1.3% 0.9% | | <0.001 | 0.040 |
|  |  | 1 2 | | 10829 | trimethoprim |  | | 3,254 14,487 | 5.5% 3.8% | | <0.001 | 0.081 |
|  |  | 1 2 | | 7454 | nitrofurantoin |  | | 310 979 | 0.5% 0.3% | | <0.001 | 0.043 |
|  |  | 1 2 | | AM200 | ERYTHROMYCINS/MACROLIDES |  | | 5,063 21,907 | 8.5% 5.7% | | <0.001 | 0.109 |
|  |  | 1 2 | | AM300 | AMINOGLYCOSIDES |  | | 3,077 11,241 | 5.2% 2.9% | | <0.001 | 0.114 |
|  |  | 1 2 | | AN000 | ANTINEOPLASTICS |  | | 1,059 4,985 | 1.8% 1.3% | | <0.001 | 0.039 |
|  |  | 1 2 | | CN709 | ANTIPSYCHOTICS,OTHER |  | | 13,513 19,657 | 22.8% 5.1% | | <0.001 | 0.526 |
|  |  | 1 2 | | CN701 | PHENOTHIAZINE/RELATED ANTIPSYCHOTICS |  | | 1,024 1,006 | 1.7% 0.3% | | <0.001 | 0.148 |
|  |  | 1 2 | | CN609 | ANTIDEPRESSANTS,OTHER |  | | 12,039 29,887 | 20.3% 7.8% | | <0.001 | 0.365 |
|  |  | 1 2 | | CN601 | TRICYCLIC ANTIDEPRESSANTS |  | | 1,760 4,385 | 3.0% 1.1% | | <0.001 | 0.129 |
|  |  | 1 2 | | CV100 | BETA BLOCKERS/RELATED |  | | 6,099 19,384 | 10.3% 5.1% | | <0.001 | 0.197 |
|  |  | 1 2 | | CV200 | CALCIUM CHANNEL BLOCKERS |  | | 3,244 11,729 | 5.5% 3.1% | | <0.001 | 0.119 |
|  |  | 1 2 | | CV800 | ACE INHIBITORS |  | | 2,607 10,009 | 4.4% 2.6% | | <0.001 | 0.097 |
|  |  | 1 2 | | CV150 | ALPHA BLOCKERS/RELATED |  | | 1,945 4,522 | 3.3% 1.2% | | <0.001 | 0.142 |
|  |  | 1 2 | | CV490 | ANTIHYPERTENSIVES,OTHER |  | | 7,959 21,303 | 13.4% 5.6% | | <0.001 | 0.270 |
|  |  | 1 2 | | HS100 | ANDROGENS/ANABOLICS |  | | 292 1,330 | 0.5% 0.3% | | <0.001 | 0.022 |
|  |  | 1 2 | | HS800 | PROGESTINS |  | | 181 510 | 0.3% 0.1% | | <0.001 | 0.037 |
|  |  | 1 2 | | HS300 | ESTROGENS |  | | 82 260 | 0.1% 0.1% | | <0.001 | 0.022 |
|  |  | 1 2 | | 9997 | spironolactone |  | | 443 2,548 | 0.7% 0.7% | | 0.025 | 0.010 |
|  |  | 1 2 | | 3014 | cyproterone |  | | 10 10 | 0.0% 0.0% | | <0.001 | 0.014 |
|  |  | 1 2 | | 114477 | levetiracetam |  | | 20,424 62,390 | 34.4% 16.3% | | <0.001 | 0.426 |
|  |  | 1 2 | | 28439 | lamotrigine |  | | 5,835 11,651 | 9.8% 3.0% | | <0.001 | 0.279 |
|  |  | 1 2 | | 25480 | gabapentin |  | | 4,460 13,608 | 7.5% 3.6% | | <0.001 | 0.174 |
|  |  | 1 2 | | 38404 | topiramate |  | | 4,754 6,382 | 8.0% 1.7% | | <0.001 | 0.299 |
|  |  | 1 2 | | 32624 | oxcarbazepine |  | | 4,952 9,757 | 8.3% 2.5% | | <0.001 | 0.258 |
|  |  | 1 2 | | 623400 | lacosamide |  | | 4,236 5,073 | 7.1% 1.3% | | <0.001 | 0.292 |
|  |  | 1 2 | | 8183 | phenytoin |  | | 3,448 8,076 | 5.8% 2.1% | | <0.001 | 0.191 |
|  |  | 1 2 | | 21241 | clobazam |  | | 3,476 2,552 | 5.9% 0.7% | | <0.001 | 0.295 |
|  |  | 1 2 | | 2002 | carbamazepine |  | | 2,771 6,721 | 4.7% 1.8% | | <0.001 | 0.166 |
|  |  | 1 2 | | 39998 | zonisamide |  | | 2,990 3,478 | 5.0% 0.9% | | <0.001 | 0.245 |
|  |  | 1 2 | | 72236 | fosphenytoin |  | | 2,303 3,773 | 3.9% 1.0% | | <0.001 | 0.189 |
|  |  | 1 2 | | 4135 | ethosuximide |  | | 1,366 1,297 | 2.3% 0.3% | | <0.001 | 0.173 |
|  |  | 1 2 | | 69036 | rufinamide |  | | 628 362 | 1.1% 0.1% | | <0.001 | 0.128 |
|  |  | 1 2 | | 1739745 | brivaracetam |  | | 492 506 | 0.8% 0.1% | | <0.001 | 0.101 |
|  |  | 1 2 | | 14851 | vigabatrin |  | | 609 306 | 1.0% 0.1% | | <0.001 | 0.128 |
|  |  | 1 2 | | 24812 | felbamate |  | | 360 277 | 0.6% 0.1% | | <0.001 | 0.092 |
|  |  | 1 2 | | 1356552 | perampanel |  | | 426 374 | 0.7% 0.1% | | <0.001 | 0.097 |
|  |  | 1 2 | | 2045371 | cannabidiol |  | | 419 309 | 0.7% 0.1% | | <0.001 | 0.100 |
|  |  | 1 2 | | 1482502 | eslicarbazepine |  | | 206 367 | 0.3% 0.1% | | <0.001 | 0.053 |
|  |  | 1 2 | | 8691 | primidone |  | | 141 265 | 0.2% 0.1% | | <0.001 | 0.043 |
|  |  | 1 2 | | 2265690 | cenobamate |  | | 114 188 | 0.2% 0.0% | | <0.001 | 0.041 |
|  |  | 1 2 | | 31914 | tiagabine |  | | 35 45 | 0.1% 0.0% | | <0.001 | 0.025 |
|  |  | 1 2 | | 4328 | fenfluramine |  | | 32 12 | 0.1% 0.0% | | <0.001 | 0.030 |
|  |  | 1 2 | | 47858 | methsuximide |  | | 10 10 | 0.0% 0.0% | | <0.001 | 0.014 |
|  |  | 1 2 | | 2054968 | stiripentol |  | | 13 10 | 0.0% 0.0% | | <0.001 | 0.017 |
|  |  | 1 2 | | CN302 | BENZODIAZEPINE DERIVATIVE SEDATIVES/HYPNOTICS |  | | 31,926 95,706 | 53.8% 25.0% | | <0.001 | 0.617 |
|  |  | 1 2 | | CN309 | SEDATIVES/HYPNOTICS,OTHER |  | | 6,368 17,136 | 10.7% 4.5% | | <0.001 | 0.238 |
|  |  | 1 2 | | CN301 | BARBITURIC ACID DERIVATIVE SEDATIVES/HYPNOTICS |  | | 3,293 8,284 | 5.5% 2.2% | | <0.001 | 0.176 |
|  |  | 1 2 | | CN101 | OPIOID ANALGESICS |  | | 17,825 74,263 | 30.0% 19.4% | | <0.001 | 0.249 |
|  |  | 1 2 | | HS051 | GLUCOCORTICOIDS |  | | 15,538 72,305 | 26.2% 18.9% | | <0.001 | 0.175 |
|  | **Laboratory** | | | | | | | | | | | |
|  |  | Cohort | | |  | Mean ± SD | | Patients | % of Cohort | | P-Value | SMD |
|  |  | 1 2 | | 9083 | BMI | 24.6 +/- 7.0 24.2 +/- 7.3 | | 29,800 140,320 | 50.2% 36.6% | | <0.001 | 0.055 |
|  |  | 1 2 | |  | 0 - 0 kg/m2 |  | | 29,851 140,722 | 50.3% 36.7% | | <0.001 | 0.276 |
| **Cohort 1 (N = 50,102) and cohort 2 (N = 50,102) characteristics after propensity score matching** | | | | | | | | | | | | |
|  | **Demographics** | | | | | | | | | | | |
|  |  | Cohort | | |  | Mean ± SD | | Patients | % of Cohort | | P-Value | SMD |
|  |  | 1 2 | | AI | Age at Index | 22.6 +/- 13.3 23.9 +/- 14.1 | | 50,102 50,102 | 100% 100% | | <0.001 | 0.096 |
|  |  | 1 2 | | 2106-3 | White |  | | 28,015 28,567 | 55.9% 57.0% | | <0.001 | 0.022 |
|  |  | 1 2 | | 1002-5 | American Indian or Alaska Native |  | | 202 213 | 0.4% 0.4% | | 0.588 | 0.003 |
|  |  | 1 2 | | UNK | Unknown Race |  | | 7,900 7,482 | 15.8% 14.9% | | <0.001 | 0.023 |
|  |  | 1 2 | | 2076-8 | Native Hawaiian or Other Pacific Islander |  | | 177 197 | 0.4% 0.4% | | 0.300 | 0.007 |
|  |  | 1 2 | | 2054-5 | Black or African American |  | | 8,386 8,288 | 16.7% 16.5% | | 0.406 | 0.005 |
|  |  | 1 2 | | 2131-1 | Other Race |  | | 3,160 3,145 | 6.3% 6.3% | | 0.845 | 0.001 |
|  |  | 1 2 | | 2028-9 | Asian |  | | 2,262 2,210 | 4.5% 4.4% | | 0.426 | 0.005 |
|  | **Diagnosis** | | | | | | | | | | | |
|  |  | Cohort | | |  | Mean ± SD | | Patients | % of Cohort | | P-Value | SMD |
|  |  | 1 2 | | F80-F89 | Pervasive and specific developmental disorders |  | | 7,975 7,806 | 15.9% 15.6% | | 0.143 | 0.009 |
|  |  | 1 2 | | F40-F48 | Anxiety, dissociative, stress-related, somatoform and other nonpsychotic mental disorders |  | | 8,426 9,324 | 16.8% 18.6% | | <0.001 | 0.047 |
|  |  | 1 2 | | F90-F98 | Behavioral and emotional disorders with onset usually occurring in childhood and adolescence |  | | 6,134 6,186 | 12.2% 12.3% | | 0.617 | 0.003 |
|  |  | 1 2 | | F20-F29 | Schizophrenia, schizotypal, delusional, and other non-mood psychotic disorders |  | | 2,881 2,875 | 5.8% 5.7% | | 0.935 | 0.001 |
|  |  | 1 2 | | F60-F69 | Disorders of adult personality and behavior |  | | 2,043 2,034 | 4.1% 4.1% | | 0.886 | 0.001 |
|  |  | 1 2 | | F70-F79 | Intellectual Disabilities |  | | 3,195 3,090 | 6.4% 6.2% | | 0.171 | 0.009 |
|  |  | 1 2 | | F01-F09 | Mental disorders due to known physiological conditions |  | | 2,005 2,063 | 4.0% 4.1% | | 0.353 | 0.006 |
|  |  | 1 2 | | F99-F99 | Unspecified mental disorder (F99) |  | | 653 655 | 1.3% 1.3% | | 0.956 | <0.001 |
|  |  | 1 2 | | E08-E13 | Diabetes mellitus |  | | 2,033 2,276 | 4.1% 4.5% | | <0.001 | 0.024 |
|  |  | 1 2 | | Q00-Q07 | Congenital malformations of the nervous system |  | | 1,859 1,824 | 3.7% 3.6% | | 0.557 | 0.004 |
|  |  | 1 2 | | Q60-Q64 | Congenital malformations of the urinary system |  | | 327 317 | 0.7% 0.6% | | 0.693 | 0.002 |
|  |  | 1 2 | | N45 | Orchitis and epididymitis |  | | 198 212 | 0.4% 0.4% | | 0.488 | 0.004 |
|  |  | 1 2 | | N43 | Hydrocele and spermatocele |  | | 277 283 | 0.6% 0.6% | | 0.799 | 0.002 |
|  |  | 1 2 | | N50.1 | Vascular disorders of male genital organs |  | | 20 12 | 0.0% 0.0% | | 0.157 | 0.009 |
|  |  | 1 2 | | N49 | Inflammatory disorders of male genital organs, not elsewhere classified |  | | 63 56 | 0.1% 0.1% | | 0.521 | 0.004 |
|  |  | 1 2 | | I86.1 | Scrotal varices |  | | 82 98 | 0.2% 0.2% | | 0.233 | 0.008 |
|  |  | 1 2 | | Q53 | Undescended and ectopic testicle |  | | 332 331 | 0.7% 0.7% | | 0.969 | <0.001 |
|  |  | 1 2 | | Q55 | Other congenital malformations of male genital organs |  | | 211 228 | 0.4% 0.5% | | 0.416 | 0.005 |
|  |  | 1 2 | | Q54 | Hypospadias |  | | 138 133 | 0.3% 0.3% | | 0.761 | 0.002 |
|  |  | 1 2 | | C60-C63 | Malignant neoplasms of male genital organs |  | | 58 56 | 0.1% 0.1% | | 0.851 | 0.001 |
|  |  | 1 2 | | N44.8 | Other noninflammatory disorders of the testis |  | | 98 100 | 0.2% 0.2% | | 0.887 | 0.001 |
|  |  | 1 2 | | N44.0 | Torsion of testis |  | | 31 35 | 0.1% 0.1% | | 0.622 | 0.003 |
|  |  | 1 2 | | N30-N39 | Other diseases of the urinary system |  | | 2,500 2,601 | 5.0% 5.2% | | 0.147 | 0.009 |
|  |  | 1 2 | | K40 | Inguinal hernia |  | | 465 506 | 0.9% 1.0% | | 0.186 | 0.008 |
|  |  | 1 2 | | E84 | Cystic fibrosis |  | | 32 41 | 0.1% 0.1% | | 0.292 | 0.007 |
|  |  | 1 2 | | S30-S39 | Injuries to the abdomen, lower back, lumbar spine, pelvis and external genitals |  | | 2,378 2,459 | 4.7% 4.9% | | 0.233 | 0.008 |
|  |  | 1 2 | | E23.0 | Hypopituitarism |  | | 246 245 | 0.5% 0.5% | | 0.964 | <0.001 |
|  |  | 1 2 | | R56 | Convulsions, not elsewhere classified |  | | 23,997 22,812 | 47.9% 45.5% | | <0.001 | 0.047 |
|  |  | 1 2 | | E34.5 | Androgen insensitivity syndrome |  | | 10 10 | 0.0% 0.0% | | 1 | <0.001 |
|  |  | 1 2 | | Q87.1 | Congenital malformation syndromes predominantly associated with short stature |  | | 56 54 | 0.1% 0.1% | | 0.849 | 0.001 |
|  |  | 1 2 | | F31 | Bipolar disorder |  | | 3,030 3,112 | 6.0% 6.2% | | 0.280 | 0.007 |
|  |  | 1 2 | | F32 | Depressive episode |  | | 5,145 5,508 | 10.3% 11.0% | | <0.001 | 0.024 |
|  |  | 1 2 | | F39 | Unspecified mood [affective] disorder |  | | 1,680 1,719 | 3.4% 3.4% | | 0.496 | 0.004 |
|  |  | 1 2 | | F33 | Major depressive disorder, recurrent |  | | 1,244 1,308 | 2.5% 2.6% | | 0.199 | 0.008 |
|  |  | 1 2 | | F34 | Persistent mood [affective] disorders |  | | 644 682 | 1.3% 1.4% | | 0.293 | 0.007 |
|  |  | 1 2 | | F30 | Manic episode |  | | 154 161 | 0.3% 0.3% | | 0.693 | 0.002 |
|  |  | 1 2 | | F50-F59 | Behavioral syndromes associated with physiological disturbances and physical factors |  | | 1,047 1,097 | 2.1% 2.2% | | 0.275 | 0.007 |
|  |  | 1 2 | | J00-J99 | Diseases of the respiratory system |  | | 15,278 15,360 | 30.5% 30.7% | | 0.574 | 0.004 |
|  |  | 1 2 | | I00-I99 | Diseases of the circulatory system |  | | 10,876 11,496 | 21.7% 22.9% | | <0.001 | 0.030 |
|  |  | 1 2 | | K00-K95 | Diseases of the digestive system |  | | 12,977 13,510 | 25.9% 27.0% | | <0.001 | 0.024 |
|  |  | 1 2 | | Q65-Q79 | Congenital malformations and deformations of the musculoskeletal system |  | | 1,627 1,657 | 3.2% 3.3% | | 0.595 | 0.003 |
|  |  | 1 2 | | N17-N19 | Acute kidney failure and chronic kidney disease |  | | 2,281 2,429 | 4.6% 4.8% | | 0.027 | 0.014 |
|  |  | 1 2 | | A50-A64 | Infections with a predominantly sexual mode of transmission |  | | 306 326 | 0.6% 0.7% | | 0.425 | 0.005 |
|  |  | 1 2 | | E03 | Other hypothyroidism |  | | 1,144 1,162 | 2.3% 2.3% | | 0.705 | 0.002 |
|  |  | 1 2 | | E05 | Thyrotoxicosis [hyperthyroidism] |  | | 131 139 | 0.3% 0.3% | | 0.626 | 0.003 |
|  |  | 1 2 | | E83.1 | Disorders of iron metabolism |  | | 58 70 | 0.1% 0.1% | | 0.289 | 0.007 |
|  |  | 1 2 | | Q99 | Other chromosome abnormalities, not elsewhere classified |  | | 579 544 | 1.2% 1.1% | | 0.294 | 0.007 |
|  |  | 1 2 | | Q93 | Monosomies and deletions from the autosomes, not elsewhere classified |  | | 297 302 | 0.6% 0.6% | | 0.838 | 0.001 |
|  |  | 1 2 | | Q90 | Down syndrome |  | | 251 266 | 0.5% 0.5% | | 0.508 | 0.004 |
|  |  | 1 2 | | Q92 | Other trisomies and partial trisomies of the autosomes, not elsewhere classified |  | | 96 96 | 0.2% 0.2% | | 1 | <0.001 |
|  |  | 1 2 | | Q98.4 | Klinefelter syndrome, unspecified |  | | 41 37 | 0.1% 0.1% | | 0.650 | 0.003 |
|  |  | 1 2 | | Q98.5 | Karyotype 47, XYY |  | | 20 22 | 0.0% 0.0% | | 0.758 | 0.002 |
|  |  | 1 2 | | Q98.7 | Male with sex chromosome mosaicism |  | | 20 18 | 0.0% 0.0% | | 0.746 | 0.002 |
|  |  | 1 2 | | Q98.8 | Other specified sex chromosome abnormalities, male phenotype |  | | 19 21 | 0.0% 0.0% | | 0.752 | 0.002 |
|  |  | 1 2 | | Q98.0 | Klinefelter syndrome karyotype 47, XXY |  | | 10 10 | 0.0% 0.0% | | 1 | <0.001 |
|  |  | 1 2 | | Q98.1 | Klinefelter syndrome, male with more than two X chromosomes |  | | 10 10 | 0.0% 0.0% | | 1 | <0.001 |
|  |  | 1 2 | | Q98.6 | Male with structurally abnormal sex chromosome |  | | 10 10 | 0.0% 0.0% | | 1 | <0.001 |
|  |  | 1 2 | | Q98.9 | Sex chromosome abnormality, male phenotype, unspecified |  | | 10 10 | 0.0% 0.0% | | 1 | <0.001 |
|  |  | 1 2 | | D35.2 | Benign neoplasm of pituitary gland |  | | 54 49 | 0.1% 0.1% | | 0.622 | 0.003 |
|  |  | 1 2 | | D35.3 | Benign neoplasm of craniopharyngeal duct |  | | 22 21 | 0.0% 0.0% | | 0.879 | 0.001 |
|  |  | 1 2 | | E40-E46 | Malnutrition |  | | 1,283 1,338 | 2.6% 2.7% | | 0.276 | 0.007 |
|  |  | 1 2 | | G40.9 | Epilepsy, unspecified |  | | 22,328 21,219 | 44.6% 42.4% | | <0.001 | 0.045 |
|  |  | 1 2 | | G40.3 | Generalized idiopathic epilepsy and epileptic syndromes |  | | 6,026 5,888 | 12.0% 11.8% | | 0.178 | 0.009 |
|  |  | 1 2 | | G40.4 | Other generalized epilepsy and epileptic syndromes |  | | 4,661 4,533 | 9.3% 9.0% | | 0.161 | 0.009 |
|  |  | 1 2 | | G40.2 | Localization-related (focal) (partial) symptomatic epilepsy and epileptic syndromes with complex partial seizures |  | | 4,643 4,161 | 9.3% 8.3% | | <0.001 | 0.034 |
|  |  | 1 2 | | G40.8 | Other epilepsy and recurrent seizures |  | | 3,571 3,378 | 7.1% 6.7% | | 0.016 | 0.015 |
|  |  | 1 2 | | G40.1 | Localization-related (focal) (partial) symptomatic epilepsy and epileptic syndromes with simple partial seizures |  | | 3,282 3,044 | 6.6% 6.1% | | 0.002 | 0.020 |
|  |  | 1 2 | | G40.A | Absence epileptic syndrome |  | | 1,778 1,743 | 3.5% 3.5% | | 0.548 | 0.004 |
|  |  | 1 2 | | G40.0 | Localization-related (focal) (partial) idiopathic epilepsy and epileptic syndromes with seizures of localized onset |  | | 1,064 1,001 | 2.1% 2.0% | | 0.161 | 0.009 |
|  |  | 1 2 | | G40.5 | Epileptic seizures related to external causes |  | | 618 616 | 1.2% 1.2% | | 0.954 | <0.001 |
|  |  | 1 2 | | G40.B | Juvenile myoclonic epilepsy [impulsive petit mal] |  | | 509 530 | 1.0% 1.1% | | 0.513 | 0.004 |
|  |  | 1 2 | | F17 | Nicotine dependence |  | | 5,790 6,426 | 11.6% 12.8% | | <0.001 | 0.039 |
|  |  | 1 2 | | F12 | Cannabis related disorders |  | | 2,879 2,918 | 5.7% 5.8% | | 0.598 | 0.003 |
|  |  | 1 2 | | F10 | Alcohol related disorders |  | | 3,159 3,332 | 6.3% 6.7% | | 0.026 | 0.014 |
|  |  | 1 2 | | F19 | Other psychoactive substance related disorders |  | | 2,226 2,277 | 4.4% 4.5% | | 0.437 | 0.005 |
|  |  | 1 2 | | F11 | Opioid related disorders |  | | 1,488 1,589 | 3.0% 3.2% | | 0.064 | 0.012 |
|  |  | 1 2 | | F14 | Cocaine related disorders |  | | 1,020 1,097 | 2.0% 2.2% | | 0.091 | 0.011 |
|  |  | 1 2 | | F15 | Other stimulant related disorders |  | | 964 986 | 1.9% 2.0% | | 0.615 | 0.003 |
|  |  | 1 2 | | F13 | Sedative, hypnotic, or anxiolytic related disorders |  | | 676 688 | 1.3% 1.4% | | 0.744 | 0.002 |
|  |  | 1 2 | | F18 | Inhalant related disorders |  | | 493 522 | 1.0% 1.0% | | 0.360 | 0.006 |
|  |  | 1 2 | | F16 | Hallucinogen related disorders |  | | 208 218 | 0.4% 0.4% | | 0.627 | 0.003 |
|  | **Procedure** | | | | | | | | | | | |
|  |  | Cohort | | |  | Mean ± SD | | Patients | % of Cohort | | P-Value | SMD |
|  |  | 1 2 | | 1010843 | Radiation Oncology Treatment |  | | 131 132 | 0.3% 0.3% | | 0.951 | <0.001 |
|  |  | 1 2 | | 1008061 | Surgical Procedures on the Urinary System |  | | 1,338 1,385 | 2.7% 2.8% | | 0.361 | 0.006 |
|  |  | 1 2 | | 1008011 | Repair initial inguinal hernia, age 5 years or older |  | | 57 61 | 0.1% 0.1% | | 0.713 | 0.002 |
|  |  | 1 2 | | 1008470 | Surgical Procedures on the Male Genital System |  | | 441 459 | 0.9% 0.9% | | 0.547 | 0.004 |
|  | **Medication** | | | | | | | | | | | |
|  |  | Cohort | | |  | Mean ± SD | | Patients | % of Cohort | | P-Value | SMD |
|  |  | 1 2 | | 25025 | finasteride |  | | 92 118 | 0.2% 0.2% | | 0.072 | 0.011 |
|  |  | 1 2 | | CN750 | LITHIUM SALTS |  | | 503 545 | 1.0% 1.1% | | 0.192 | 0.008 |
|  |  | 1 2 | | 6135 | ketoconazole |  | | 570 621 | 1.1% 1.2% | | 0.137 | 0.009 |
|  |  | 1 2 | | 10829 | trimethoprim |  | | 2,419 2,546 | 4.8% 5.1% | | 0.064 | 0.012 |
|  |  | 1 2 | | 7454 | nitrofurantoin |  | | 218 249 | 0.4% 0.5% | | 0.150 | 0.009 |
|  |  | 1 2 | | AM200 | ERYTHROMYCINS/MACROLIDES |  | | 3,728 4,067 | 7.4% 8.1% | | <0.001 | 0.025 |
|  |  | 1 2 | | AM300 | AMINOGLYCOSIDES |  | | 2,219 2,361 | 4.4% 4.7% | | 0.032 | 0.014 |
|  |  | 1 2 | | AN000 | ANTINEOPLASTICS |  | | 819 925 | 1.6% 1.8% | | 0.010 | 0.016 |
|  |  | 1 2 | | CN709 | ANTIPSYCHOTICS,OTHER |  | | 9,899 10,373 | 19.8% 20.7% | | <0.001 | 0.024 |
|  |  | 1 2 | | CN701 | PHENOTHIAZINE/RELATED ANTIPSYCHOTICS |  | | 629 617 | 1.3% 1.2% | | 0.732 | 0.002 |
|  |  | 1 2 | | CN609 | ANTIDEPRESSANTS,OTHER |  | | 9,226 9,991 | 18.4% 19.9% | | <0.001 | 0.039 |
|  |  | 1 2 | | CN601 | TRICYCLIC ANTIDEPRESSANTS |  | | 1,344 1,501 | 2.7% 3.0% | | 0.003 | 0.019 |
|  |  | 1 2 | | CV100 | BETA BLOCKERS/RELATED |  | | 4,814 5,150 | 9.6% 10.3% | | <0.001 | 0.022 |
|  |  | 1 2 | | CV200 | CALCIUM CHANNEL BLOCKERS |  | | 2,638 2,804 | 5.3% 5.6% | | 0.021 | 0.015 |
|  |  | 1 2 | | CV800 | ACE INHIBITORS |  | | 2,069 2,253 | 4.1% 4.5% | | 0.004 | 0.018 |
|  |  | 1 2 | | CV150 | ALPHA BLOCKERS/RELATED |  | | 1,487 1,599 | 3.0% 3.2% | | 0.041 | 0.013 |
|  |  | 1 2 | | CV490 | ANTIHYPERTENSIVES,OTHER |  | | 5,880 6,183 | 11.7% 12.3% | | 0.003 | 0.019 |
|  |  | 1 2 | | HS100 | ANDROGENS/ANABOLICS |  | | 231 273 | 0.5% 0.5% | | 0.061 | 0.012 |
|  |  | 1 2 | | HS800 | PROGESTINS |  | | 141 159 | 0.3% 0.3% | | 0.298 | 0.007 |
|  |  | 1 2 | | HS300 | ESTROGENS |  | | 66 78 | 0.1% 0.2% | | 0.317 | 0.006 |
|  |  | 1 2 | | 9997 | spironolactone |  | | 362 405 | 0.7% 0.8% | | 0.119 | 0.010 |
|  |  | 1 2 | | 3014 | cyproterone |  | | 10 10 | 0.0% 0.0% | | 1 | <0.001 |
|  |  | 1 2 | | 114477 | levetiracetam |  | | 15,004 15,325 | 29.9% 30.6% | | 0.027 | 0.014 |
|  |  | 1 2 | | 28439 | lamotrigine |  | | 3,955 4,206 | 7.9% 8.4% | | 0.004 | 0.018 |
|  |  | 1 2 | | 25480 | gabapentin |  | | 3,537 3,838 | 7.1% 7.7% | | <0.001 | 0.023 |
|  |  | 1 2 | | 38404 | topiramate |  | | 2,832 2,993 | 5.7% 6.0% | | 0.030 | 0.014 |
|  |  | 1 2 | | 32624 | oxcarbazepine |  | | 3,132 3,189 | 6.3% 6.4% | | 0.459 | 0.005 |
|  |  | 1 2 | | 623400 | lacosamide |  | | 2,391 2,518 | 4.8% 5.0% | | 0.063 | 0.012 |
|  |  | 1 2 | | 8183 | phenytoin |  | | 2,403 2,527 | 4.8% 5.0% | | 0.070 | 0.011 |
|  |  | 1 2 | | 21241 | clobazam |  | | 1,663 1,706 | 3.3% 3.4% | | 0.451 | 0.005 |
|  |  | 1 2 | | 2002 | carbamazepine |  | | 1,971 2,160 | 3.9% 4.3% | | 0.003 | 0.019 |
|  |  | 1 2 | | 39998 | zonisamide |  | | 1,580 1,643 | 3.2% 3.3% | | 0.259 | 0.007 |
|  |  | 1 2 | | 72236 | fosphenytoin |  | | 1,199 1,157 | 2.4% 2.3% | | 0.381 | 0.006 |
|  |  | 1 2 | | 4135 | ethosuximide |  | | 800 778 | 1.6% 1.6% | | 0.577 | 0.004 |
|  |  | 1 2 | | 69036 | rufinamide |  | | 273 270 | 0.5% 0.5% | | 0.897 | 0.001 |
|  |  | 1 2 | | 1739745 | brivaracetam |  | | 238 249 | 0.5% 0.5% | | 0.617 | 0.003 |
|  |  | 1 2 | | 14851 | vigabatrin |  | | 240 228 | 0.5% 0.5% | | 0.578 | 0.004 |
|  |  | 1 2 | | 24812 | felbamate |  | | 180 194 | 0.4% 0.4% | | 0.468 | 0.005 |
|  |  | 1 2 | | 1356552 | perampanel |  | | 206 232 | 0.4% 0.5% | | 0.213 | 0.008 |
|  |  | 1 2 | | 2045371 | cannabidiol |  | | 168 184 | 0.3% 0.4% | | 0.393 | 0.005 |
|  |  | 1 2 | | 1482502 | eslicarbazepine |  | | 121 125 | 0.2% 0.2% | | 0.798 | 0.002 |
|  |  | 1 2 | | 8691 | primidone |  | | 101 102 | 0.2% 0.2% | | 0.944 | <0.001 |
|  |  | 1 2 | | 2265690 | cenobamate |  | | 59 69 | 0.1% 0.1% | | 0.376 | 0.006 |
|  |  | 1 2 | | 31914 | tiagabine |  | | 23 25 | 0.0% 0.0% | | 0.773 | 0.002 |
|  |  | 1 2 | | 4328 | fenfluramine |  | | 10 10 | 0.0% 0.0% | | 1 | <0.001 |
|  |  | 1 2 | | 47858 | methsuximide |  | | 10 10 | 0.0% 0.0% | | 1 | <0.001 |
|  |  | 1 2 | | 2054968 | stiripentol |  | | 10 10 | 0.0% 0.0% | | 1 | <0.001 |
|  |  | 1 2 | | CN302 | BENZODIAZEPINE DERIVATIVE SEDATIVES/HYPNOTICS |  | | 24,039 24,817 | 48.0% 49.5% | | <0.001 | 0.031 |
|  |  | 1 2 | | CN309 | SEDATIVES/HYPNOTICS,OTHER |  | | 4,484 4,642 | 8.9% 9.3% | | 0.083 | 0.011 |
|  |  | 1 2 | | CN301 | BARBITURIC ACID DERIVATIVE SEDATIVES/HYPNOTICS |  | | 2,087 2,126 | 4.2% 4.2% | | 0.539 | 0.004 |
|  |  | 1 2 | | CN101 | OPIOID ANALGESICS |  | | 13,868 14,952 | 27.7% 29.8% | | <0.001 | 0.048 |
|  |  | 1 2 | | HS051 | GLUCOCORTICOIDS |  | | 11,754 12,413 | 23.5% 24.8% | | <0.001 | 0.031 |
|  | **Laboratory** | | | | | | | | | | | |
|  |  | Cohort | | |  | Mean ± SD | | Patients | % of Cohort | | P-Value | SMD |
|  |  | 1 2 | | 9083 | BMI | 24.8 +/- 6.9 25.2 +/- 7.2 | | 23,866 25,479 | 47.6% 50.9% | | <0.001 | 0.067 |
|  |  | 1 2 | |  | 0 - 0 kg/m2 |  | | 23,911 25,549 | 47.7% 51.0% | | <0.001 | 0.065 |

**Abbreviations:** BMI = Body Mass Index; SD = Standard deviation; SMD = Standardised mean difference

**N.B.:** Uncorrected p-values were calculated using two-sided t-tests for continuous covariates and two-sided Z-tests for categorical covariates. These p-values reflect descriptive comparisons of baseline characteristics during propensity score matching. Correction for multiple comparisons is not appropriate in this context, as the aim is to assess covariate balance (reflected by a standardised mean difference of <0.1) rather than to test hypotheses. Exact p-values beyond three decimal places are not provided in the propensity score matching outputs generated by the TriNetX platform. Where p-values fall below this threshold, they are reported as <0.001.

#

# Supplementary Table 12: Propensity score matching men with bipolar disorder exposed (cohort 1) and unexposed (cohort 2) to valproate

Propensity score matching was performed on all listed characteristics. Characteristics of the cohorts before and after matching are summarized in the table below.

| **Cohort 1 and cohort 2 patient count before and after propensity score matching** | | | | | | | | | | | | |
| --- | --- | --- | --- | --- | --- | --- | --- | --- | --- | --- | --- | --- |
|  | | | Cohort | | | Patient count before matching | | | | Patient count after matching | | |
|  | | | 1 - Men with bipolar disorder exposed to valproate_v9 | | | 33,450 | | | | 22,826 | | |
|  | | | 2 - Men with bipolar disorder not exposed to valproate_v9 | | | 123,881 | | | | 22,826 | | |
| **Propensity score density function - Before and after matching (cohort 1 - purple, cohort 2 - green)** | | | | | | | | | | | | |
|  |  | | 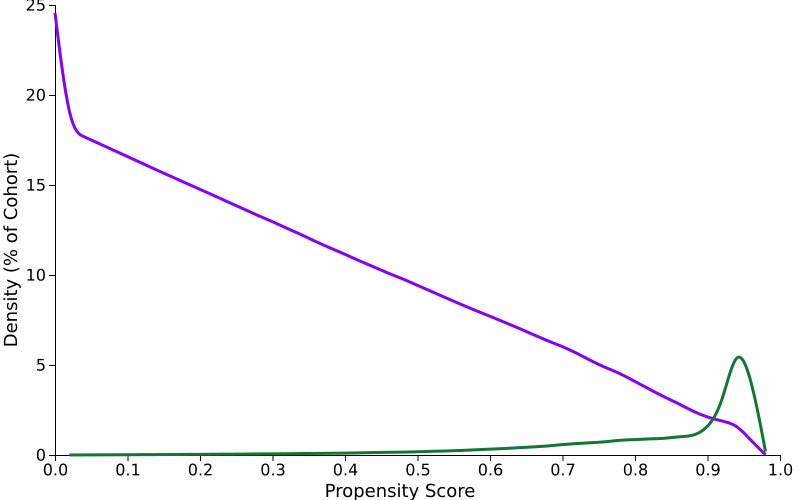 | | | | 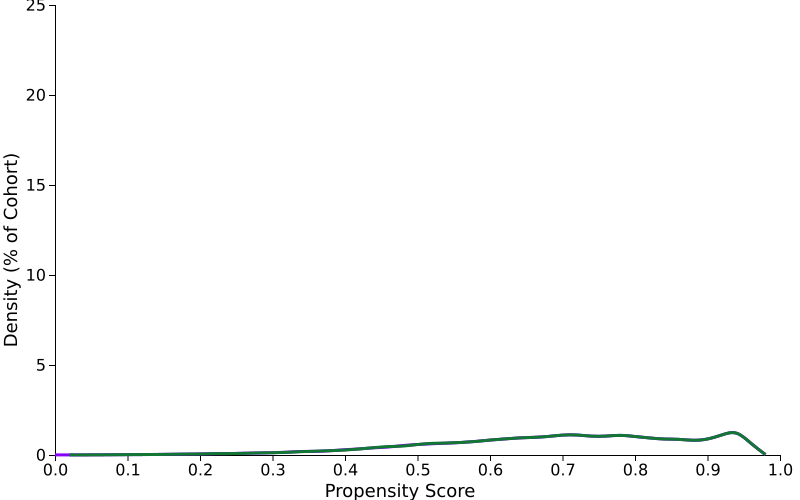 | | | | | |
| **Cohort 1 (N = 33,450) and cohort 2 (N = 123,881) characteristics before propensity score matching** | | | | | | | | | | | | |
|  | **Demographics** | | | | | | | | | | | |
|  |  | Cohort | | |  | Mean ± SD | | Patients | % of Cohort | | P-Value | SMD |
|  |  | 1 2 | | AI | Age at Index | 30.7 +/- 9.8 30.8 +/- 9.9 | | 32,494 120,983 | 100% 100% | | 0.327 | 0.006 |
|  |  | 1 2 | | 2106-3 | White |  | | 20,015 81,140 | 61.6% 67.1% | | <0.001 | 0.114 |
|  |  | 1 2 | | 1002-5 | American Indian or Alaska Native |  | | 127 436 | 0.4% 0.4% | | 0.420 | 0.005 |
|  |  | 1 2 | | UNK | Unknown Race |  | | 3,086 11,666 | 9.5% 9.6% | | 0.429 | 0.005 |
|  |  | 1 2 | | 2076-8 | Native Hawaiian or Other Pacific Islander |  | | 176 604 | 0.5% 0.5% | | 0.340 | 0.006 |
|  |  | 1 2 | | 2054-5 | Black or African American |  | | 6,809 19,861 | 21.0% 16.4% | | <0.001 | 0.117 |
|  |  | 1 2 | | 2131-1 | Other Race |  | | 1,266 4,688 | 3.9% 3.9% | | 0.861 | 0.001 |
|  |  | 1 2 | | 2028-9 | Asian |  | | 1,015 2,588 | 3.1% 2.1% | | <0.001 | 0.062 |
|  | **Diagnosis** | | | | | | | | | | | |
|  |  | Cohort | | |  | Mean ± SD | | Patients | % of Cohort | | P-Value | SMD |
|  |  | 1 2 | | F80-F89 | Pervasive and specific developmental disorders |  | | 2,796 5,552 | 8.6% 4.6% | | <0.001 | 0.162 |
|  |  | 1 2 | | F40-F48 | Anxiety, dissociative, stress-related, somatoform and other nonpsychotic mental disorders |  | | 15,459 51,152 | 47.6% 42.3% | | <0.001 | 0.107 |
|  |  | 1 2 | | F90-F98 | Behavioral and emotional disorders with onset usually occurring in childhood and adolescence |  | | 7,821 24,386 | 24.1% 20.2% | | <0.001 | 0.094 |
|  |  | 1 2 | | F20-F29 | Schizophrenia, schizotypal, delusional, and other non-mood psychotic disorders |  | | 11,092 21,599 | 34.1% 17.9% | | <0.001 | 0.378 |
|  |  | 1 2 | | F60-F69 | Disorders of adult personality and behavior |  | | 4,859 8,957 | 15.0% 7.4% | | <0.001 | 0.241 |
|  |  | 1 2 | | F70-F79 | Intellectual Disabilities |  | | 1,741 1,716 | 5.4% 1.4% | | <0.001 | 0.219 |
|  |  | 1 2 | | F01-F09 | Mental disorders due to known physiological conditions |  | | 1,829 2,708 | 5.6% 2.2% | | <0.001 | 0.175 |
|  |  | 1 2 | | F99-F99 | Unspecified mental disorder (F99) |  | | 1,828 3,480 | 5.6% 2.9% | | <0.001 | 0.137 |
|  |  | 1 2 | | E08-E13 | Diabetes mellitus |  | | 2,423 7,557 | 7.5% 6.2% | | <0.001 | 0.048 |
|  |  | 1 2 | | Q00-Q07 | Congenital malformations of the nervous system |  | | 193 316 | 0.6% 0.3% | | <0.001 | 0.051 |
|  |  | 1 2 | | Q60-Q64 | Congenital malformations of the urinary system |  | | 125 322 | 0.4% 0.3% | | <0.001 | 0.021 |
|  |  | 1 2 | | N45 | Orchitis and epididymitis |  | | 276 866 | 0.8% 0.7% | | 0.013 | 0.015 |
|  |  | 1 2 | | N43 | Hydrocele and spermatocele |  | | 228 648 | 0.7% 0.5% | | <0.001 | 0.021 |
|  |  | 1 2 | | N50.1 | Vascular disorders of male genital organs |  | | 22 48 | 0.1% 0.0% | | 0.036 | 0.012 |
|  |  | 1 2 | | N49 | Inflammatory disorders of male genital organs, not elsewhere classified |  | | 69 237 | 0.2% 0.2% | | 0.555 | 0.004 |
|  |  | 1 2 | | I86.1 | Scrotal varices |  | | 140 517 | 0.4% 0.4% | | 0.931 | 0.001 |
|  |  | 1 2 | | Q53 | Undescended and ectopic testicle |  | | 51 128 | 0.2% 0.1% | | 0.016 | 0.014 |
|  |  | 1 2 | | Q55 | Other congenital malformations of male genital organs |  | | 63 180 | 0.2% 0.1% | | 0.069 | 0.011 |
|  |  | 1 2 | | Q54 | Hypospadias |  | | 36 62 | 0.1% 0.1% | | <0.001 | 0.021 |
|  |  | 1 2 | | C60-C63 | Malignant neoplasms of male genital organs |  | | 35 184 | 0.1% 0.2% | | 0.060 | 0.012 |
|  |  | 1 2 | | N44.8 | Other noninflammatory disorders of the testis |  | | 138 340 | 0.4% 0.3% | | <0.001 | 0.024 |
|  |  | 1 2 | | N44.0 | Torsion of testis |  | | 24 87 | 0.1% 0.1% | | 0.908 | 0.001 |
|  |  | 1 2 | | N30-N39 | Other diseases of the urinary system |  | | 1,731 4,223 | 5.3% 3.5% | | <0.001 | 0.090 |
|  |  | 1 2 | | K40 | Inguinal hernia |  | | 314 1,135 | 1.0% 0.9% | | 0.641 | 0.003 |
|  |  | 1 2 | | E84 | Cystic fibrosis |  | | 23 80 | 0.1% 0.1% | | 0.773 | 0.002 |
|  |  | 1 2 | | S30-S39 | Injuries to the abdomen, lower back, lumbar spine, pelvis and external genitals |  | | 2,667 7,508 | 8.2% 6.2% | | <0.001 | 0.077 |
|  |  | 1 2 | | E23.0 | Hypopituitarism |  | | 66 181 | 0.2% 0.1% | | 0.033 | 0.013 |
|  |  | 1 2 | | R56 | Convulsions, not elsewhere classified |  | | 3,111 4,953 | 9.6% 4.1% | | <0.001 | 0.218 |
|  |  | 1 2 | | E34.5 | Androgen insensitivity syndrome |  | | 10 10 | 0.0% 0.0% | | 0.002 | 0.016 |
|  |  | 1 2 | | Q87.1 | Congenital malformation syndromes predominantly associated with short stature |  | | 19 34 | 0.1% 0.0% | | 0.009 | 0.015 |
|  |  | 1 2 | | F31 | Bipolar disorder |  | | 24,280 120,983 | 74.7% 100% | | <0.001 | 0.823 |
|  |  | 1 2 | | F32 | Depressive episode |  | | 9,827 30,158 | 30.2% 24.9% | | <0.001 | 0.119 |
|  |  | 1 2 | | F39 | Unspecified mood [affective] disorder |  | | 5,203 10,008 | 16.0% 8.3% | | <0.001 | 0.239 |
|  |  | 1 2 | | F33 | Major depressive disorder, recurrent |  | | 3,321 10,290 | 10.2% 8.5% | | <0.001 | 0.059 |
|  |  | 1 2 | | F34 | Persistent mood [affective] disorders |  | | 1,460 3,550 | 4.5% 2.9% | | <0.001 | 0.083 |
|  |  | 1 2 | | F30 | Manic episode |  | | 2,140 2,635 | 6.6% 2.2% | | <0.001 | 0.217 |
|  |  | 1 2 | | F50-F59 | Behavioral syndromes associated with physiological disturbances and physical factors |  | | 1,421 3,900 | 4.4% 3.2% | | <0.001 | 0.060 |
|  |  | 1 2 | | J00-J99 | Diseases of the respiratory system |  | | 10,856 35,997 | 33.4% 29.8% | | <0.001 | 0.079 |
|  |  | 1 2 | | I00-I99 | Diseases of the circulatory system |  | | 9,670 29,123 | 29.8% 24.1% | | <0.001 | 0.128 |
|  |  | 1 2 | | K00-K95 | Diseases of the digestive system |  | | 10,690 34,836 | 32.9% 28.8% | | <0.001 | 0.089 |
|  |  | 1 2 | | Q65-Q79 | Congenital malformations and deformations of the musculoskeletal system |  | | 481 1,251 | 1.5% 1.0% | | <0.001 | 0.040 |
|  |  | 1 2 | | N17-N19 | Acute kidney failure and chronic kidney disease |  | | 1,740 4,407 | 5.4% 3.6% | | <0.001 | 0.083 |
|  |  | 1 2 | | A50-A64 | Infections with a predominantly sexual mode of transmission |  | | 684 2,480 | 2.1% 2.0% | | 0.535 | 0.004 |
|  |  | 1 2 | | E03 | Other hypothyroidism |  | | 1,341 3,189 | 4.1% 2.6% | | <0.001 | 0.083 |
|  |  | 1 2 | | E05 | Thyrotoxicosis [hyperthyroidism] |  | | 220 513 | 0.7% 0.4% | | <0.001 | 0.034 |
|  |  | 1 2 | | E83.1 | Disorders of iron metabolism |  | | 33 151 | 0.1% 0.1% | | 0.282 | 0.007 |
|  |  | 1 2 | | Q99 | Other chromosome abnormalities, not elsewhere classified |  | | 68 106 | 0.2% 0.1% | | <0.001 | 0.032 |
|  |  | 1 2 | | Q93 | Monosomies and deletions from the autosomes, not elsewhere classified |  | | 21 31 | 0.1% 0.0% | | 0.001 | 0.018 |
|  |  | 1 2 | | Q90 | Down syndrome |  | | 21 66 | 0.1% 0.1% | | 0.498 | 0.004 |
|  |  | 1 2 | | Q92 | Other trisomies and partial trisomies of the autosomes, not elsewhere classified |  | | 10 15 | 0.0% 0.0% | | 0.021 | 0.013 |
|  |  | 1 2 | | Q98.4 | Klinefelter syndrome, unspecified |  | | 30 67 | 0.1% 0.1% | | 0.019 | 0.014 |
|  |  | 1 2 | | Q98.5 | Karyotype 47, XYY |  | | 10 11 | 0.0% 0.0% | | 0.003 | 0.015 |
|  |  | 1 2 | | Q98.7 | Male with sex chromosome mosaicism |  | | 10 10 | 0.0% 0.0% | | 0.002 | 0.016 |
|  |  | 1 2 | | Q98.8 | Other specified sex chromosome abnormalities, male phenotype |  | | 10 10 | 0.0% 0.0% | | 0.002 | 0.016 |
|  |  | 1 2 | | Q98.0 | Klinefelter syndrome karyotype 47, XXY |  | | 10 10 | 0.0% 0.0% | | 0.002 | 0.016 |
|  |  | 1 2 | | Q98.1 | Klinefelter syndrome, male with more than two X chromosomes |  | | 10 0 | 0.0% 0% | | <0.001 | 0.025 |
|  |  | 1 2 | | Q98.6 | Male with structurally abnormal sex chromosome |  | | 0 0 | 0% 0% | | -- | -- |
|  |  | 1 2 | | Q98.9 | Sex chromosome abnormality, male phenotype, unspecified |  | | 0 0 | 0% 0% | | -- | -- |
|  |  | 1 2 | | D35.2 | Benign neoplasm of pituitary gland |  | | 37 111 | 0.1% 0.1% | | 0.254 | 0.007 |
|  |  | 1 2 | | D35.3 | Benign neoplasm of craniopharyngeal duct |  | | 16 49 | 0.0% 0.0% | | 0.497 | 0.004 |
|  |  | 1 2 | | E40-E46 | Malnutrition |  | | 563 1,323 | 1.7% 1.1% | | <0.001 | 0.054 |
|  |  | 1 2 | | G40.9 | Epilepsy, unspecified |  | | 2,515 3,502 | 7.7% 2.9% | | <0.001 | 0.217 |
|  |  | 1 2 | | G40.3 | Generalized idiopathic epilepsy and epileptic syndromes |  | | 351 408 | 1.1% 0.3% | | <0.001 | 0.089 |
|  |  | 1 2 | | G40.4 | Other generalized epilepsy and epileptic syndromes |  | | 368 412 | 1.1% 0.3% | | <0.001 | 0.093 |
|  |  | 1 2 | | G40.2 | Localization-related (focal) (partial) symptomatic epilepsy and epileptic syndromes with complex partial seizures |  | | 310 388 | 1.0% 0.3% | | <0.001 | 0.080 |
|  |  | 1 2 | | G40.8 | Other epilepsy and recurrent seizures |  | | 385 440 | 1.2% 0.4% | | <0.001 | 0.094 |
|  |  | 1 2 | | G40.1 | Localization-related (focal) (partial) symptomatic epilepsy and epileptic syndromes with simple partial seizures |  | | 229 298 | 0.7% 0.2% | | <0.001 | 0.067 |
|  |  | 1 2 | | G40.A | Absence epileptic syndrome |  | | 69 93 | 0.2% 0.1% | | <0.001 | 0.036 |
|  |  | 1 2 | | G40.0 | Localization-related (focal) (partial) idiopathic epilepsy and epileptic syndromes with seizures of localized onset |  | | 52 87 | 0.2% 0.1% | | <0.001 | 0.026 |
|  |  | 1 2 | | G40.5 | Epileptic seizures related to external causes |  | | 116 123 | 0.4% 0.1% | | <0.001 | 0.053 |
|  |  | 1 2 | | G40.B | Juvenile myoclonic epilepsy [impulsive petit mal] |  | | 17 11 | 0.1% 0.0% | | <0.001 | 0.025 |
|  |  | 1 2 | | F17 | Nicotine dependence |  | | 11,567 35,418 | 35.6% 29.3% | | <0.001 | 0.135 |
|  |  | 1 2 | | F12 | Cannabis related disorders |  | | 8,416 18,451 | 25.9% 15.3% | | <0.001 | 0.266 |
|  |  | 1 2 | | F10 | Alcohol related disorders |  | | 6,274 17,788 | 19.3% 14.7% | | <0.001 | 0.123 |
|  |  | 1 2 | | F19 | Other psychoactive substance related disorders |  | | 5,725 14,067 | 17.6% 11.6% | | <0.001 | 0.170 |
|  |  | 1 2 | | F11 | Opioid related disorders |  | | 3,179 10,005 | 9.8% 8.3% | | <0.001 | 0.053 |
|  |  | 1 2 | | F14 | Cocaine related disorders |  | | 3,029 7,095 | 9.3% 5.9% | | <0.001 | 0.131 |
|  |  | 1 2 | | F15 | Other stimulant related disorders |  | | 2,998 7,527 | 9.2% 6.2% | | <0.001 | 0.113 |
|  |  | 1 2 | | F13 | Sedative, hypnotic, or anxiolytic related disorders |  | | 1,411 2,875 | 4.3% 2.4% | | <0.001 | 0.109 |
|  |  | 1 2 | | F18 | Inhalant related disorders |  | | 1,241 2,900 | 3.8% 2.4% | | <0.001 | 0.082 |
|  |  | 1 2 | | F16 | Hallucinogen related disorders |  | | 698 1,429 | 2.1% 1.2% | | <0.001 | 0.076 |
|  | **Procedure** | | | | | | | | | | | |
|  |  | Cohort | | |  | Mean ± SD | | Patients | % of Cohort | | P-Value | SMD |
|  |  | 1 2 | | 1010843 | Radiation Oncology Treatment |  | | 25 59 | 0.1% 0.0% | | 0.054 | 0.011 |
|  |  | 1 2 | | 1008061 | Surgical Procedures on the Urinary System |  | | 875 1,856 | 2.7% 1.5% | | <0.001 | 0.081 |
|  |  | 1 2 | | 1008011 | Repair initial inguinal hernia, age 5 years or older |  | | 56 164 | 0.2% 0.1% | | 0.120 | 0.009 |
|  |  | 1 2 | | 1008470 | Surgical Procedures on the Male Genital System |  | | 299 927 | 0.9% 0.8% | | 0.006 | 0.017 |
|  | **Medication** | | | | | | | | | | | |
|  |  | Cohort | | |  | Mean ± SD | | Patients | % of Cohort | | P-Value | SMD |
|  |  | 1 2 | | 25025 | finasteride |  | | 119 454 | 0.4% 0.4% | | 0.813 | 0.001 |
|  |  | 1 2 | | CN750 | LITHIUM SALTS |  | | 3,622 7,067 | 11.1% 5.8% | | <0.001 | 0.191 |
|  |  | 1 2 | | 6135 | ketoconazole |  | | 648 1,514 | 2.0% 1.3% | | <0.001 | 0.059 |
|  |  | 1 2 | | 10829 | trimethoprim |  | | 2,372 6,347 | 7.3% 5.2% | | <0.001 | 0.085 |
|  |  | 1 2 | | 7454 | nitrofurantoin |  | | 168 289 | 0.5% 0.2% | | <0.001 | 0.045 |
|  |  | 1 2 | | AM200 | ERYTHROMYCINS/MACROLIDES |  | | 3,520 9,899 | 10.8% 8.2% | | <0.001 | 0.090 |
|  |  | 1 2 | | AM300 | AMINOGLYCOSIDES |  | | 1,508 3,349 | 4.6% 2.8% | | <0.001 | 0.099 |
[truncated: 108,606 more chars]
