# Supplementary material for: A retrospective cohort study of valproate and infertility in men with epilepsy or bipolar disorder using international health data
Source: Nat Commun. 2025 Sep 15;16:8221. doi: 10.1038/s41467-025-63469-0 (PMC12436621; doi:10.1038/s41467-025-63469-0)
Supplement: Supplementary file 2 — Reporting Summary [file 41467_2025_63469_MOESM2_ESM.pdf]

nature portfolio

Corresponding author(s): Dr Gashirai Mbizvo

Last updated by author(s): Apr 9, 2024

# Reporting Summary

Nature Portfolio wishes to improve the reproducibility of the work that we publish. This form provides structure for consistency and transparency in reporting. For further information on Nature Portfolio policies, see our Editorial Policies and the Editorial Policy Checklist.

## Statistics

For all statistical analyses, confirm that the following items are present in the figure legend, table legend, main text, or Methods section.

| n/a | Confirmed | |
|---|---|---|
| ☐ | ☒ | The exact sample size (*n*) for each experimental group/condition, given as a discrete number and unit of measurement |
| ☒ | ☐ | A statement on whether measurements were taken from distinct samples or whether the same sample was measured repeatedly |
| ☐ | ☒ | The statistical test(s) used AND whether they are one- or two-sided<br>*Only common tests should be described solely by name; describe more complex techniques in the Methods section.* |
| ☐ | ☒ | A description of all covariates tested |
| ☒ | ☐ | A description of any assumptions or corrections, such as tests of normality and adjustment for multiple comparisons |
| ☐ | ☒ | A full description of the statistical parameters including central tendency (e.g. means) or other basic estimates (e.g. regression coefficient) AND variation (e.g. standard deviation) or associated estimates of uncertainty (e.g. confidence intervals) |
| ☒ | ☐ | For null hypothesis testing, the test statistic (e.g. $F$, $t$, $r$) with confidence intervals, effect sizes, degrees of freedom and $P$ value noted<br>*Give P values as exact values whenever suitable.* |
| ☒ | ☐ | For Bayesian analysis, information on the choice of priors and Markov chain Monte Carlo settings |
| ☒ | ☐ | For hierarchical and complex designs, identification of the appropriate level for tests and full reporting of outcomes |
| ☒ | ☐ | Estimates of effect sizes (e.g. Cohen's $d$, Pearson's $r$), indicating how they were calculated |

*Our web collection on statistics for biologists contains articles on many of the points above.*

## Software and code

Policy information about availability of computer code

| | |
|---|---|
| Data collection | Undertaken in the TriNetX platform using R4.0.2, Python 3.7, and Java 11.0.16. |
| Data analysis | Undertaken in the TriNetX platform using R4.0.2, Python 3.7, and Java 11.0.16. |

For manuscripts utilizing custom algorithms or software that are central to the research but not yet described in published literature, software must be made available to editors and reviewers. We strongly encourage code deposition in a community repository (e.g. GitHub). See the Nature Portfolio guidelines for submitting code & software for further information.

## Data

Policy information about availability of data

All manuscripts must include a data availability statement. This statement should provide the following information, where applicable:

- Accession codes, unique identifiers, or web links for publicly available datasets
- A description of any restrictions on data availability
- For clinical datasets or third party data, please ensure that the statement adheres to our policy

All analyses were conducted within the TriNetX LIVE platform, and results were exported as aggregate data outputs and summary statistics, which we have made publicly available via FigShare (https://doi.org/10.6084/m9.figshare.28728839). These are not the Source Data required to reproduce the figures and tables in our manuscript. The Source Data required to reproduce the figures and tables in our manuscript are real-world clinical records from patients and so are held securely within the TriNetX LIVE platform; they cannot be publicly deposited under TriNetX policy and the U.S. HIPAA Privacy Rule de-identification requirements (§164.514).

Other researchers can request access to the TriNetX LIVE platform (www.trinetx.com/about-trinetx/contact) via their institution, follow the methods described in this manuscript within the TriNetX LIVE platform, and in doing so, reproduce the figures and tables, with small variations in counts expected due to real-time data updates from participating healthcare organisations over time. Custom code was not generated for this study.

## Research involving human participants, their data, or biological material

Policy information about studies with human participants or human data. See also policy information about sex, gender (identity/presentation), and sexual orientation and race, ethnicity and racism.

| | |
|---|---|
| Reporting on sex and gender | In referring to men in this study, we report the biological sex of the patients (male), as recorded by healthcare providers and captured subsequently by TriNetX. |
| Reporting on race, ethnicity, or other socially relevant groupings | We report the ethnic heritage of patients (including the subgroups of: White, Black or African American, Unknown Race, Other Race, Asian, American Indian or Alaska Native, Native Hawaiian or Other Pacific Islander), as recorded by healthcare providers and captured subsequently by TriNetX. |
| Population characteristics | Other characteristics reported include age, diagnoses, medications, medical procedures, and laboratory results, as recorded by healthcare providers and captured subsequently by TriNetX. |
| Recruitment | Not applicable - this was a retrospective cohort study of fully anonymised healthcare data |
| Ethics oversight | The University of Liverpool Research Ethics decision tool was used to determine that ethical approval was not required as the study was a secondary analysis of data that were anonymised by an external party (TriNetX) and provided to the research team in the fully anonymised format. Data were de-identified per the de-identification standard defined in Section 164.514(a) of the HIPAA Privacy Rule. TriNetX data are attenuated to ensure participating HCOs remain anonymised. This includes withholding the names of participating countries with less than three HCOs contributing data, and shielding frequencies to ≤10 when ten or less patients experience an outcome, in order to protect their anonymity. No personal data were used or patient recruitment undertaken as part of this study |

Note that full information on the approval of the study protocol must also be provided in the manuscript.

# Field-specific reporting

Please select the one below that is the best fit for your research. If you are not sure, read the appropriate sections before making your selection.

☒ Life sciences ☐ Behavioural & social sciences ☐ Ecological, evolutionary & environmental sciences

For a reference copy of the document with all sections, see nature.com/documents/nr-reporting-summary-flat.pdf

# Life sciences study design

All studies must disclose on these points even when the disclosure is negative.

| | |
|---|---|
| Sample size | As this was a retrospective study of routinely collected healthcare data from the TriNetX platform, no a priori sample size calculation was performed. Instead, all eligible patients were included from the platform, which has a denominator of 275 million patients, offering a sufficient size to support robust comparisons for most clinical conditions. 627,720 patients with epilepsy or bipolar disorder were studied. |
| Data exclusions | Men aged 55 years or older, women at any age |
| Replication | We acknowledge that the dynamic nature of data acquisition onto the TriNetX platform serves as both a strength and a limitation. It is a strength that new patients and HCOs are added daily, meaning sample sizes increase with time and results are representative of real-time frontline clinical decisions and progress. Conversely, this growth can make it challenging to replicate exact counts over time. Nonetheless, we expect that trends with a strong biological basis will retain their direction of effect despite the evolving nature of the dataset, and our findings support this expectation as there were no changes in the direction of effect or conclusions drawn between our original search at submission (conducted on 09/06/2024, capturing 606,785 men with epilepsy or bipolar disorder from 120 HCOs; logged here: https://doi.org/10.6084/m9.figshare.27310551.v1) and our updated search after the first peer review (conducted on 24/10/2024, capturing an expanded 633,405 men from 131 HCOs; logged here: https://doi.org/10.6084/m9.figshare.27310593.v1). The results included in the current version of the manuscript are drawn from updated searches undertaken on 24/03/2025 following a further round of peer review. Conclusions remain unchanged. |
| Randomization | Propensity score matching was used to balance baseline covariates - emulating randomisation |
| Blinding | Blinding is not possible in a retrospective cohort study of real-world healthcare data, as the data reflect treatment decisions that were made by clinicians and patients during the course of routine clinical care, which would not be blinded. |

# Reporting for specific materials, systems and methods

We require information from authors about some types of materials, experimental systems and methods used in many studies. Here, indicate whether each material, system or method listed is relevant to your study. If you are not sure if a list item applies to your research, read the appropriate section before selecting a response.

## Materials & experimental systems

| n/a | Involved in the study |
| --- | --- |
| ☒ | ☐ Antibodies |
| ☒ | ☐ Eukaryotic cell lines |
| ☒ | ☐ Palaeontology and archaeology |
| ☒ | ☐ Animals and other organisms |
| ☒ | ☐ Clinical data |
| ☒ | ☐ Dual use research of concern |
| ☒ | ☐ Plants |

## Methods

| n/a | Involved in the study |
| --- | --- |
| ☒ | ☐ ChIP-seq |
| ☒ | ☐ Flow cytometry |
| ☒ | ☐ MRI-based neuroimaging |

# Plants

| | |
| --- | --- |
| Seed stocks | n/a |
| Novel plant genotypes | n/a |
| Authentication | n/a |
